# Evolutionary history of the poly(ADP-ribose) polymerase gene family in eukaryotes

**DOI:** 10.1186/1471-2148-10-308

**Published:** 2010-10-13

**Authors:** Matteo Citarelli, Sachin Teotia, Rebecca S Lamb

**Affiliations:** 1 Plant Cellular and Molecular Biology Department, Ohio State University, 500 Aronoff Laboratory, 318 W. 12th Ave., Columbus, OH 43210 USA; 2 Molcular, Cellular and Developmental Biology Program, Ohio State University, Columbus, OH 43210 USA

## Abstract

**Background:**

The Poly(ADP-ribose)polymerase (PARP) superfamily was originally identified as enzymes that catalyze the attachment of ADP-ribose subunits to target proteins using NAD^+ ^as a substrate. The family is characterized by the catalytic site, termed the PARP signature. While these proteins can be found in a range of eukaryotes, they have been best studied in mammals. In these organisms, PARPs have key functions in DNA repair, genome integrity and epigenetic regulation. More recently it has been found that proteins within the PARP superfamily have altered catalytic sites, and have mono(ADP-ribose) transferase (mART) activity or are enzymatically inactive. These findings suggest that the PARP signature has a broader range of functions that initially predicted. In this study, we investigate the evolutionary history of PARP genes across the eukaryotes.

**Results:**

We identified *in silico *236 PARP proteins from 77 species across five of the six eukaryotic supergroups. We performed extensive phylogenetic analyses of the identified PARPs. They are found in all eukaryotic supergroups for which sequence is available, but some individual lineages within supergroups have independently lost these genes. The PARP superfamily can be subdivided into six clades. Two of these clades were likely found in the last common eukaryotic ancestor. In addition, we have identified PARPs in organisms in which they have not previously been described.

**Conclusions:**

Three main conclusions can be drawn from our study. First, the broad distribution and pattern of representation of PARP genes indicates that the ancestor of all extant eukaryotes encoded proteins of this type. Second, the ancestral PARP proteins had different functions and activities. One of these proteins was similar to human PARP1 and likely functioned in DNA damage response. The second of the ancestral PARPs had already evolved differences in its catalytic domain that suggest that these proteins may not have possessed poly(ADP-ribosyl)ation activity. Third, the diversity of the PARP superfamily is larger than previously documented, suggesting as more eukaryotic genomes become available, this gene family will grow in both number and type.

## Background

Poly(ADP-ribosyl)ation activity was originally identified in the 1960s [1-5]; it is the rapid and reversible posttranslational covalent attachment of ADP-ribose subunits onto glutamate, aspartate, and lysine residues of target proteins. The ADP-ribose polymer is formed by sequential attachment of ADP-ribosyl moieties from NAD^+^; the polymers can reach a length of over 200 units and can have multiple branching points. Overall, the ADP-ribose polymer is highly negatively charged and has large physiological consequences on functional and biochemical properties of the proteins modified.

Poly(ADP-ribosyl)ation is done by enzymes called poly(ADP-ribose)polymerases (PARPs). The so-called PARP signature, a catalytic ß-alpha-loop-B-alpha NAD^+ ^fold [6,7], characterizes these enzymes. PARPs are found in diverse groups of eukaryotes [8,9], but are best studied in animals. PARPs have been shown to be involved in DNA damage repair, cell death pathways, transcription and chromatin modification/remodelling (reviewed in [10-13]). PARPs have been implicated in a wide range of human diseases (reviewed in [14]) and are important targets for anti-cancer therapies [15]. A polymorphism in human PARP1, which causes decreased enzymatic activity, has been reported to be associated with an increased cancer risk and a decreased risk of asthma [16,17], further underlining the importance of this class of enzymes and their complex roles in disease.

The first PARP purified and cloned, PARP1 from human, remains the best studied. PARP1 was long thought to be the only enzyme with poly(ADP-ribosyl)ation activity until two PARP isoforms were identified in plants [18] and, simultaneously, tankyrase was identified as a PARP localized at the telomere in humans [19]. Subsequently, studies on PARP1 knock out mice demonstrated that the mutant mice still possessed poly(ADP-ribosyl)ation capacity and developed normally [20,21], suggesting other enzymes existed. Since these studies, a number of genes containing the PARP signature have been identified, although a minority of them have been functionally characterized.

The PARP-like family has been best characterized in humans, where there are seventeen family members that share the PARP catalytic domain, but vary widely in other parts of the proteins [8,9]. It is postulated that different PARPs subfamilies participate in diverse events mediated by their variable domain structures. However, only some of the family members have been shown to have PARP activity, mostly in humans (PARP1 [22] and its orthologs from other species (for example, [23,24]), PARP2 [25,26], tankyrase1 [19,27], tankyrase2 [28,29], and vPARP [30]). Most of these enzymes contain an evolutionarily conserved catalytic glutamate residue in an "HYE" catalytic triad. This residue was shown to be essential for poly(ADP-ribose) chain elongation in human PARP1 [31]. It is clear that some proteins with PARP signatures missing the catalytic glutamate residue or other residues known to be important for chain elongation do not act in poly(ADP-ribosyl)ation. For example, human PARP10 has transferase activity rather than polymerase activity, adding one ADP-ribose subunit to target proteins [32]. It is thought that other PARP-like proteins may actually function in mono(ADP-ribosyl)ation [32-34] or even have non-enzymatic functions; human PARP9 appears to not have enzymatic activity [35]. Even enzymes that retain the catalytically important residues that have been identified may not act as PARPs. For example, conflicting reports about the catalytic activity of human PARP3 exist; it has been reported act in poly(ADP-ribosyl)ation [36] and mono(ADP-ribosyl)ation [37].

Our knowledge of the PARP gene family is principally based on animals, in particular mammals. This taxon is a member of the Opisthokonts, one of the six eukaryotic "supergroups" [38,39] and therefore represents only a portion of the evolutionary history and diversity of known eukaryotes. For the other five eukaryotic supergroups, studies on PARPs have been limited or non-existent. A previous study on PARPs indentified new members in more basal animals, amoebas, fungi and plants [40]. However, no representatives from Excavates or Chromalveolates were included in the analysis and only one member of Plantae (*Arabidopsis thaliana*). Here we use comparative genomics and phylogenetic analysis to investigate the distribution of PARP genes across almost the entire breadth of eukaryotes, to reconstruct the evolutionary history of this protein family and to gain insights into its functional diversification. Our results indicate that the last common ancestor of extant eukaryotes encoded at least two PARP proteins, one similar to human PARP1 and functioning in DNA repair and damage response, the other likely acting in mono(ADP-ribosyl)ation; the cellular role of the last group is not known.

## Results

### Identification of PARP genes from eukaryotic genomes

We used the information obtained from the Pfam database [41-43] and Uniprot [44,45] along with BLAST searches [46] of sequenced eukaryotic genomes at the DOE Joint Genome Institute (JGI), the Broad Institute, the J. Craig Venter Institute, ToxoDB [47], NCBI, dictyBase [48] and the Arabidopsis Information Resource (TAIR) [49] to compile the sequences of over 300 PARP proteins. After preliminary alignment and phylogenetic analysis, we reduced the number of species representing animals; specifically we choose representative species of vertebrates since the genes from this group are shared by all and kept *Drosophila melanogaster *or *Anopheles gambiae *to represent insects, since all of our sequences were from Diptera. This left us with 236 sequences from 77 eukaryotic species (Additional file 1). In addition, another 46 sequences contained regions with high similarity to the PARP catalytic domain (Additional file 2); however, these sequences were incomplete and not included in the alignment. Nonetheless, these sequences likely represent *bona fide *members of the PARP catalytic domain. The PARP catalytic domain was extracted from the proteins sequences and aligned using MUSCLE [50]. This alignment can be found in Additional file 3.

### Phylogenetic analysis of the PARP family suggests that the ancestral eukaryote had at least two PARP enzymes

**Figure 1:**
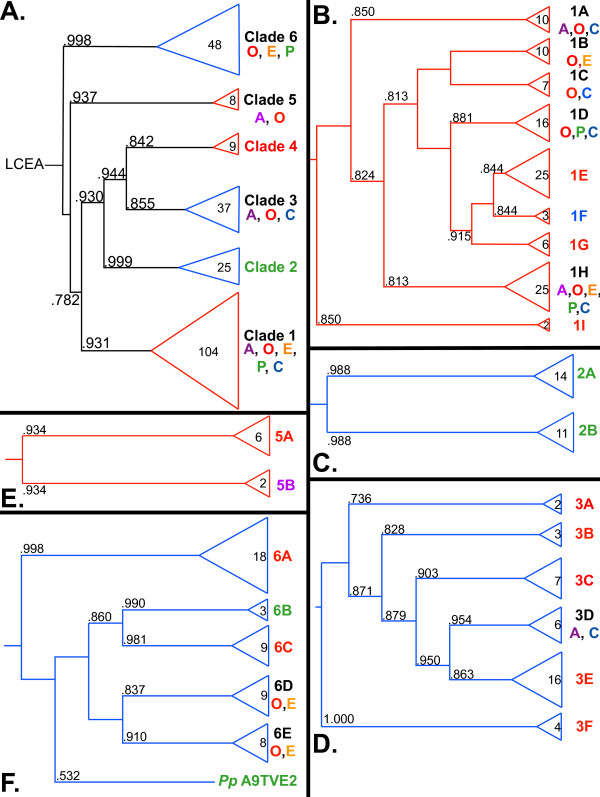
**The PARP gene family forms six clades**. A. Graphical representation of the maximum-likelihood (ML) phylogenetic tree of all identified eukaryotic PARPs indicating the relationships between the six clades as defined in the text. The full tree can be found in Additional file 4. The tree was based on an alignment of the PARP catalytic domains (Additional file 3). B. Graphical representation of the ML phylogenetic tree of Clade 1 PARPs indicating the relationships between nine subclades as defined in the text. C. Graphical representation of the ML phylogenetic tree of Clade 2 indicating the relationships between the two subclades as defined in the text. D. Graphical representation of the ML phylogenetic tree of Clade 3 PARPs indicating the relationships between the six subclades as defined in the text. E. Graphical representation of the ML phylogenetic tree of Clade 5 PARPs indicting the relationship between the two subclades as defined in the text. F. Graphical representation of the maximum-likelihood (ML) phylogenetic tree of Clade 6 PARPs indicating the relationships between the six subclades as defined in the text. Numbers in the clades or subclades indicate the number of proteins in each. Colors and letters indicate the eukaryotic supergroup or groups represented. A, Amoebozoa; O, Opithokonts; E, Excavata; P, Plantae; C, Chromalveolates. Purple, Amoebozoa; red, Opithokonts; orange, Excavata; green, Plantae; blue, chromalveolates. Branch support values are indicated at the nodes as computed in PhyML using an aLRT non-parametric Shimodaira-Hasegawa-like (SH) procedure and a midpoint rooting method. Triangle and branch colors indicate either the presence of the HYE (red) or variant (blue) catalytic triad in each group. Branch lengths do not indicate genetic distance.

**Figure 2:**
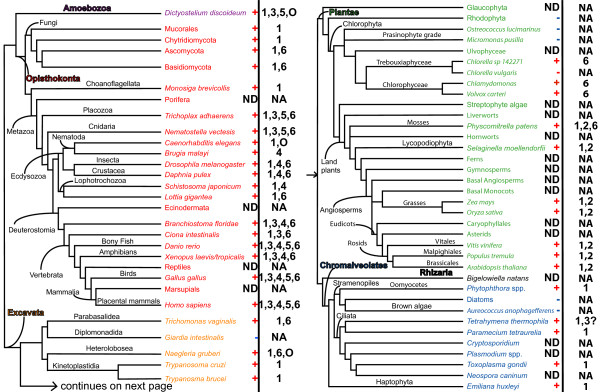
**Phylogenetic distribution of the PARP family across eukaryotes**. The topology of the schematic tree is based on recent evidence from single- and multi-gene phylogenetic analyses of eukaryotes or subgroups thereof. Some nodes, especially the deepest ones (e.g. monophyly of Excavates, Plantae or Chromalveolates + Rhizarians), remain controversial; these uncertainties do not affect the conclusions concerning the evolutionary history of the PARP family. Branch lengths do not reflect genetic distance. Presence or absence of PARP proteins are indicated by a red + or a blue -, respectively. For each species or group, PARP family members are listed with the clade numbers introduced in Figure 1. For an expanded phylogeny of the fungi, please see Figure 11. Accession numbers of the genes and details on the source of data for individual taxa is provided in Additional files 1 and 2. The six eukaryotic supergroups are indicated as follows: Amoebozoa, purple; Opisthokonta, red; Excavata, orange; Plantae, green; Rhizaria, black; Chromalveolates, blue. ND, no data; NA, not applicable; O, orphan PARPs, as discussed in the text; 3?, reflects the ambiguity of placement of the Tetrahymena proteins into this clade, as discussed in the text.

We first analyzed all the PARP-like genes we identified in the eukaryotic lineage. We used the multiple sequence alignment of the PARP catalytic domain generated above (Additional file 3) to generate a maximum-likelihood phylogenetic tree of the PARP family (Additional file 4). We defined six clades of PARPs based on our maximum-likelihood tree, an examination of domains found outside of the PARP catalytic domain used to generate that tree and the evolutionary relationships of organisms within clades (Clades 1-6; Figure 1). Clades were defined as having a bootstrap value of at least .8, one or more shared domains outside of the PARP catalytic domain, and having subbranches consisting of proteins from closely related species. Within each major clade one or more subclades were defined by similar reasoning; however, the branch supports for subclades were less stringent. Clade 5 contains proteins with almost the exact same domain structures all from closely related species; therefore, subclades were not defined for this clade. Four proteins (*Dictyostelium discoideum *DDB0232241, *Naegleria gruberi *72525, *Naegleria gruberi *80603 and *Caenorhabditis elegans *PME5) did not fall clearly into any clades; rather they fell between clades or next to proteins from widely divergent species (Additional file 4). Therefore, they have not been included in any of the defined clades. Dictyostelium DDB0232241 contains two WWE domains and a Cwf15/Cwc15 domain. WWE domains are postulated to be protein-protein interaction domains and are found in proteins involved in the ubiquitin/proteosome pathway and in PARPs [51]. Cwf15/Cwc15 domains are of unknown function and found in splicing factors [52]. *Naegleria gruberi *is a member of the Heterolobosea within the eukaryotic group Excavates (Figure 2). Heterolobosea are protozoa, many of which, including *Naegleria gruberi*, can transform between amoeboid, flagellate, and encysted stages. *Naegleria gruberi *is the only member of this group of organisms with a completed genome, making it impossible to determine if these genes are representative of ones found in a wide range of heterolobosea species or are more specific to *Naegleria *and its relatives. The two Naegleria PARP-like proteins are relatively short proteins with the PARP catalytic domain at their very C termini. Their N termini contain no known functional domains. The function of these proteins remains obscure, although they retain the "HYE" catalytic triad (Additional File 3), and may act as *bona fide *PARPs. *C. elegans *PME5 has been characterized as a tankyrase [53,54] and does share ankyrin repeats in its N terminus with those proteins, which are found in Clade 4. The placement of this protein outside of the defined clades likely reflects the large changes found in *C. elegans *PARPs (see below).

**Figure 3:**
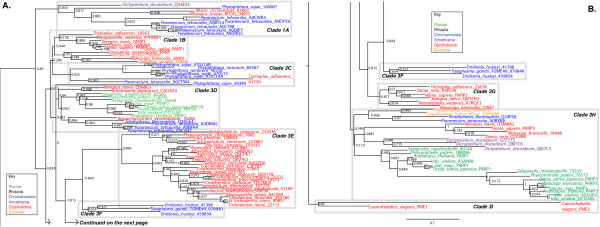
**Phylogenetic analysis of Clade 1 PARP genes**. The represented tree is a ML tree. This tree is based on a multiple alignment that includes the PARP catalytic domain (Additional file 5). Clade 1 proteins can be divided into nine subclades A-I, as indicated. Branch supports as in Figure 1. Scale bar indicates genetic distance reflected in branch length.

The PARP lineages (which will be detailed below) include one clade, Clade 1, which contains representatives from five of the six so-called eukaryotic supergroups: Plantae, Opisthokonts, Chromalveolates, Excavates, and Amoebozoa (Figures 1, 2 and 3; [38,39]). There is no completely sequenced species available from the sixth supergroup, Rhizaria. This broad distribution suggests that the last common ancestor of all extant eukaryotes encoded a gene similar to those of Clade 1. Clade 6 is only found in three of the eukaryotic supergroups; however, the position of this clade as sister group to all other members of the PARP superfamily and the placement of these groups within eukaryotes supports the hypothesis that the last common eukaryote also encoded such a gene (Figure 2).

### Clade 1: the PARP1 clade

**Figure 4:**
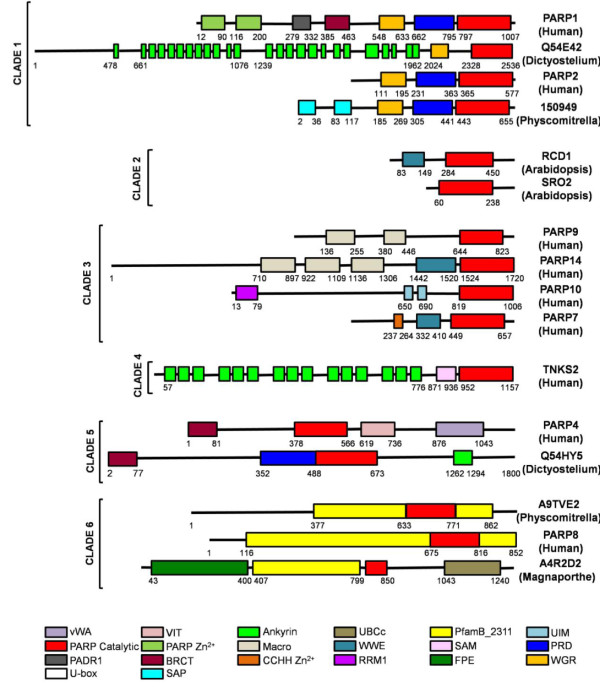
**Schematic representation of domains found in PARP proteins**. Proteins are arranged by clade as defined in Figure 1 and in the text (indicated on the left). The protein name is given on the right, with species in parenthesis. Numbers indicate amino acids. Protein domains are illustrated by coloured boxes and were defined according to Pfam 23.0. Although each protein is represented in scale, the proteins are not in scale between each other. vWA, von Willebrand factor type A; VIT, vault inter-alpha-trypsin domain; Ankyrin, ankyrin repeats; UBCc, ubiquitin conjugating enzyme catalytic domain; PfamB_2311, domain of unknown function found in Clade 6 PARPs; UIM, ubiquitin interaction motif; PARP catalytic, catalytic domain of PARPs, PARP Zn^2+^, DNA binding zinc finger; Macro, macro domain; WWE, WWE domain; SAM, sterile alpha domain; PRD, PARP regulatory domain; PADR1, domain of unknown function found in PARPs; BRCT, BRCA-1 C terminus domain; CCHH Zn^2+^, DNA binding zinc finger; RRM1, RNA-binding motif; FPE, Fungal PARP E2-like domain; WGR, domain defined by conserved Trp, Gly, and Arg residues; U-box, modified ring finger found in E3 ubiquitin ligases; SAP, DNA binding domain. Human, *Homo sapiens*; Dictyostelium, *Dictyostelium discoideum*; Arabidopsis, *Arabidopsis thaliana*; Magnaporthe, *Magnaporthe grisea*; Physcomitrella, *Physcomitrella patens*.

Clade 1 is the most broadly distributed PARP clade among eukaryotes (Figures 1, 2 and 3 and [40]). The distribution of Clade1 proteins among eukaryotic species suggests that there was at least one Clade 1-like PARP protein encoded in the genome of their last common ancestor. This group of PARPs can be subdivided into nine subclades (A-H; Figures 1 and 3). Almost all members of Clade 1 are characterized by the presence of WGR and PARP regulatory domains (PRD) in addition to the PARP catalytic domains, one of the reasons we placed these proteins together (Figure 4). The WGR domain is found in PARPs as well an *Escherichia coli *molybdate metabolism regulator and other proteins of unknown function. Its exact function is unclear, but it is proposed to be a nucleic acid binding domain. The PRD domain is found only in Clade 1 PARP proteins and has been shown to increase the poly(ADP-ribosyl)ation activity of proteins that contain it. Consistent with the presence of PRD domains, many members of Clade 1 have been demonstrated to have poly(ADP-ribosyl)ation activity, making it likely that most if not all members have this activity; this is also supported by the finding that the so-called HYE catalytic triad is conserved in almost all of these proteins (Additional files 5 and 6). Another commonality between members of Clade 1 is that many of them have been shown to have roles in DNA repair. Other common domains found in Clade 1 proteins are zinc finger DNA binding domains, BRCT domains and PADR1 domains. The BRCT domain, originally identified in the C terminus of the BRCA-1 protein, is usually found in proteins involved in cell cycle regulation and/or DNA repair [55]. The PADR1 domain is found only in PARPs (specifically Clade 1 PARPs) and is of unknown function [56].

Clade 1A is found in Amoebozoa (Dictyostelium), Opisthokonta (fungi) and Chromalveolates (the ciliate *Paramecium tetraurelia*) and is the sister group to most of the other Clade 1 subclades (with the exception of Clade 1I; Figure 3). This subclade is unique within Clade 1 in containing proteins with ankyrin repeats, in addition to WGR, PRD and PARP catalytic domains. Clade 1B contains members from both the Opisthokonta (animals and Choanoflagellata) and the Excavata (the Heterolobosea member Naegleria). This subclade is typified by human PARP1, the founding member of the superfamily. This protein has three N terminal zinc fingers that contribute to DNA binding, a BRCT domain and a PADR1 domain in addition to WGR, PRD, and the catalytic domain (Figure 4; [22,57,58]).

Both Clade 1C and 1D both contain proteins that have in common WGR, PRD and PARP catalytic domains and mostly do not contain other functional domains. Clade 1C is confined to several Oomyocete Phytophtora species (within the Excavata) and one basal animal. Clade 1D contains members from Opisthokonta (the animals *Xenopus laevis *(Q566G1) and *Schistosoma japonicum *(Q5DAZ0) and the fungus *Batrachochytrium dendrobatidis*) and Plantae (land plants) as well as ciliate members of the Chromalveolates. Some of the land plant members of Clade 1D have acquired SAP domains DNA binding domains [59] N terminal to the other domains (Figure 4). In addition, the land plant members of this group have altered their catalytic triad, alone among Clade 1 members (Additional files 5 and 6). All the plant proteins have a cysteine in place of the histidine while all except for the moss protein have a valine instead of the tyrosine in the second position. However, the plant Clade 1D proteins have retained the glutamic acid in the third position. It is unclear what effect these changes might have on the catalytic activity of these proteins.

Clade 1E contains most of the fungal members of Clade 1 and is characterized by proteins with BRCT domains N terminal to WGR, PRD and PARP catalytic domains. Clade 1F is specific to the Excavata. The *Toxoplasma gondii *representative (TGME49_070840) has a similar domain structure to human PARP1, found in Clade 1B. Clade 1G is confined to the Opisthokonta (both animals and the Choanoflagellate *Monosiga brevicollis*), contains proteins with only WGR, PRD and PARP catalytic domains and includes human PARP2.

All five eukaryotic supergroups that contain sequenced species are represented in Clade 1H (Figures 1 and 3). This clade includes human PARP3. Interestingly, land plants have duplicated one of their Clade 1H genes; one duplicate lineage appears to be changing rapidly, based on the long-branch length in the phylogenetic tree (Figure 3). These proteins may have acquired a novel function or the original function may have been split between the two copies in these species (neofunctionalization or subfunctionalization), as these processes are hypothesized to increase the probability of retention of duplicate genes [60].

The final subclade in Clade 1, Clade 1I, consists of two *Caenorhabditis elegans (C. elegans) *proteins, PME1 and PME2, which have been characterized previously [61]. PME1 contains zinc fingers and PADR1, WGR, PRD and PARP domains, while PME2 only has WGR, PRD and PARP domains. As will be discussed further below, many of the nematode proteins are anomalous.

### Clade 2: the RCD1 clade

**Figure 5:**
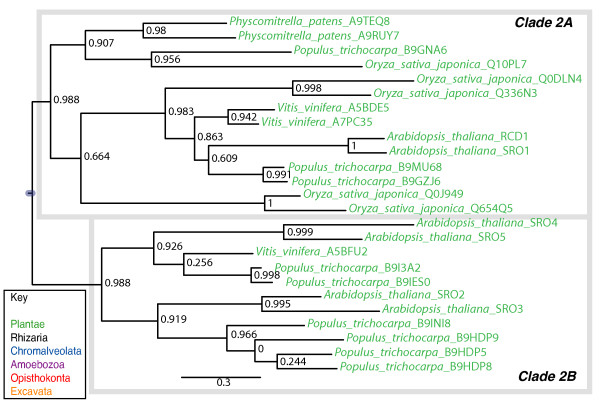
**Phylogenetic analysis of Clade 2 PARP genes**. The represented tree is a ML tree. This tree is based on a multiple alignment that includes the PARP catalytic domain (Additional file 7). Clade 2 proteins can be divided into two subclades as indicated. Branch supports as in Figure 1. Scale bar indicates genetic distance reflected in branch length.

Clade 2 of PARP-like genes consists of proteins identified only in land plants, with representatives found from bryophytes to angiosperms (Figures 2 and 5), a finding that has also been made by another group [62]. However, there is no genomic information available for any member of the streptophyte algae, the sister group to land plants within Plantae, leaving open the possibility that members of this clade may be found in these organisms (Figure 2). All groups of land plants also contain members of Clade 1 PARPs, while the moss *Physcomitrella patens *contains Clade 6 proteins in addition (Figure 2).

The founding member of this type of PARP-like protein, RADICAL-INDUCED CELL DEATH 1 (RCD1), was identified in a genetic screen in the model plant *Arabidopsis thaliana *for genes involved in cell death in response to ozone [63] and has been shown to be involved in response to a number of abiotic stresses [64]. Other members of this clade have subsequently been identified based on sequence similarity and several are also involved in stress response [62,65,66]. Clade 2 is made up two subclades (Figure 5). Clade 2A consists of proteins that have, in common with RCD1, an N terminal WWE domain, the PARP signature and a C terminal extension (Figure 4) and are found throughout the breadth of the land plants (Figure 5). Clade 2B is apparently eudicot specific (Figure 5) and consists of relatively short proteins with only the PARP signature and the C terminal extension (Figure 4). Although Clade 2A proteins contain WWE domains, they do not group with another group of WWE containing PARPs, which fall into Clade 3, a clade with no plant representatives (see below). RCD1 has recently been shown to be enzymatically inactive, a result consistent with the lack of conservation of many of the catalytic residues within the PARP domain (Additional file 7; [62]).

One interesting observation we made concerning Clade 2 was the large number of independent gene duplications that have occurred within this gene lineage (Figure 5). While this is likely due to the propensity of plant genomes to undergo whole genome duplications (reviewed in [67]), the retention of many of the gene pairs suggests that Clade 2 proteins are undergoing neofunctionalization and/or subfunctionalization at a high rate [60,68]. This supposition is supported for a pair of Clade 2A paralogs in *Arabidopsis thaliana*, *RCD1 *and *SIMILAR TO RCD ONE 1 (SRO1)*, which have been shown to be only partially redundant despite a relatively recent evolutionary origin [65,69].

### Clade 3

**Figure 6:**
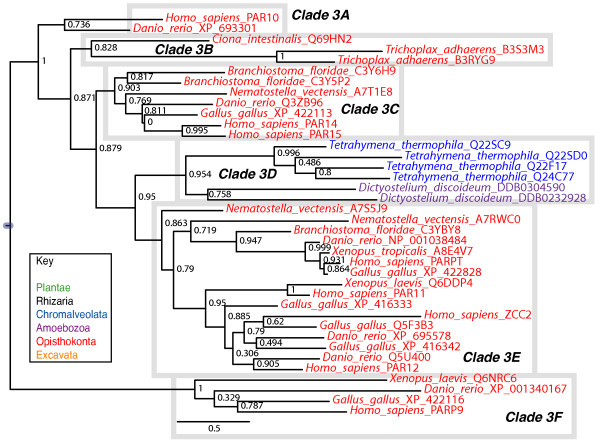
**Phylogenetic analysis of Clade 3 PARP genes**. The represented tree is a ML tree. This tree is based on a multiple alignment that includes the PARP catalytic domain (Additional file 8). Clade 3 proteins can be divided into six subclades A-F as indicated. Branch supports as in Figure 1. Scale bar indicates genetic distance reflected in branch length.

**Figure 7:**
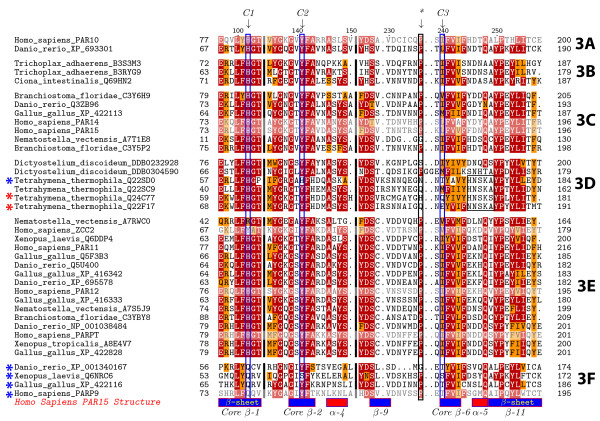
**Clade 3 PARPs have divergent catalytic domains**. Alignment of the deduced amino acid sequences of part of the PARP catalytic domains from Clade 3 proteins. Species names and protein accession numbers are shown at left. Numbers indicate amino acid position within each PARP catalytic domain while the labels on the right indicate the subclade to which the sequences belong. Dots indicate gaps introduced to maximize the alignment; the black thick lines indicate missing amino acids introduced to allow representation of all three residues of the catalytic triad, indicated by the blue boxes (C1 = H; C2 = Y; C3 = E). Only one Clade 3 protein contains a glutamic acid residue at the third position, while another has a glutamine (both indicated with red asterisks). Most Clade 3 proteins have substituted aliphatic amino acids (no asterisk), while five have serine or threonine at the position of the glutamic acid (blue asterisks). The black box surrounds a short motif characteristic of *Tetrahymena thermophila *Clade 3 proteins. The black box labelled with an asterisk indicates a proline residue that is found in most of the Clade 3 proteins. Shaded sequences indicate proteins for which a 3D structure is available.

Clade 3 contains proteins from three of the six eukaryotic supergroups: Opisthokonts (animals), Amoebozoa (*Dictyostelium discoideum*) and Chromalveolates (*Tetrahymena thermophila*) (Figures 1 and 6). This clade is likely to be somewhat artificial; the domain structures outside of the PARP catalytic domain are heterogeneous among Clade 3 proteins and the presence of *Tetrahymena thermophila *sequences within a group that otherwise contains Opisthokonts and Amoebozoa (which are sister groups) is unlikely to be real. These proteins do share certain characteristics in their catalytic domains suggestive of a switch from PARP activity to mART activity. PARP family members have catalytic domains containing the "HYE" catalytic triad conserved throughout the ADPr transferase superfamily [32]. The third residue, normally a glutamic acid, is not conserved in most Clade 3 members (Figure 7 and Additional file 8), with only one of its members retaining this residue (*Tetrahymena thermophila *Q22F17), while a second has a glutamine (*Tetrahymena thermophila *Q24C77). Most members of the clade have substituted the aliphatic amino acids isoleucine, valine, methionine or leucine for the glutamic acid, while one Tetrahymena protein (Q22SD0) as well as human PARP9 and its vertebrate orthologs have threonine or serine at this position. These substitutions have consequences for the catalytic activity of these proteins; these proteins likely do not have poly(ADP-ribosyl)ation activity [32]. It is likely that the grouping of at least the Tetrahymena proteins into this clade is a result of convergent evolution of mART activity.

Given the heterogeneous composition of Clade 3, it is difficult to divide into subclades; however, we classified the proteins into six subclades as outlined below, partially for the purpose of discussion, and partially based on common domain structures and features of the catalytic domains (Figures 6 and 7 and Additional file 8). Clade 3A is composed of two proteins, including human PARP10, containing an RRM RNA binding domain [70], a glycine-rich region (GRD), and a UIM domain, known to bind monoubiquitin and polyubiquitin chains [71]. The proteins found in Clade 3B and 3C contain at least one Macro domain N terminal to their C terminal catalytic domain (Figure 4). Macro domains have been shown to bind to poly(ADP-ribose) (PAR) [72]. Clade 3B includes representatives from the most basal animal in our study *Trichoplax adhaerens*, while 3C includes two human proteins, PARP14 and PARP15. PARP10, PARP14 and PARP15 have been demonstrated to have mART activity [32].

Clade 3D consists of the two *Dictyostelium discoideum *and four *Tetrahymena thermophila *proteins. Unlike the majority of animal proteins in Clade 3, only one of these proteins have a proline located one amino acid away from the third residue of the catalytic triad (Figure 7). The four proteins from the ciliate *Tetrahymena thermophilia *have no known functional domains outside of their C terminal PARP catalytic domains and are only similar to one another in this region (data not shown), again supporting the idea that these proteins are not closely evolutionarily related to the other proteins in Clade 3. One of the Tetrahymena proteins has retained the glutamic acid of the "HYE" (Figure 7), again supporting this interpretation. All four proteins also share a H/NNSK motif just past the last amino acid of the putative catalytic triad not found in other members of Clade 3 (Figure 7). The Dictyostelium proteins in 3D do not show high similarity outside of the PARP domain. DDB0304590 is a relatively short protein with only the PARP catalytic domain and a short C terminal extension. DDB0232928 has a Macro domain and, at its very N terminus, a U-box (Figure 4). The U-box is a modified RING finger [73] found in E3 ubiquitin ligases known to bind ubiquitin E2 enzymes [74]. As Amoebozoa is the sister group to Opisthokonts within eukaryotes and given that DDB0232928 contains a Macro domain as do some other members of Clade 3, it is possible that these proteins are orthologous to at least some of the animal Clade 3 proteins.

Clade 3E is confined to animals, but is not represented in Placozoa (Figure 6). Members of this subclade contain one to two WWE domains, alone or in combination with zinc fingers (either CCCH or CCCH types) in front of their PARP catalytic domains (Figure 4). All members of 3E have replaced the glutamic acid characteristic of PARPs with an isoleucine except for two (human ZCC2/PARP13 and *Nematostella vectensis *A7RWC0) that contain valines at that site (Figure 7). This subclade also contains human PARP12 and human PARPT/PARP7.

Clade 3F, which is sister group to all other Clade 3 subclades, contains human PARP9 and orthologs from vertebrates. These proteins contain two Macro domains N terminal to their PARP catalytic domains (Figure 4) and have a more divergent catalytic triad than the rest of Clade 3, having Q-Y/S-T/S instead of HYE (Figure 7 and Additional file 8). Human PARP9 has been shown to be inactive [35], suggesting that no Clade 3F proteins act as enzymes. PARP9 was originally identified as a gene conferring risk for diffuse large B-cell lymphoma and named BAL1 (B-aggressive lymphoma 1) [75]. Interestingly, two proteins identified by their similarity to BAL1, PARP14/BAL2 and PARP15/BAL3, although their domain structures resemble that of PARP9/BAL1, group in subclade 3C (Figures 6 and 7), and act as mARTs [35].

### Clade 4: the tankyrase clade

**Figure 8:**
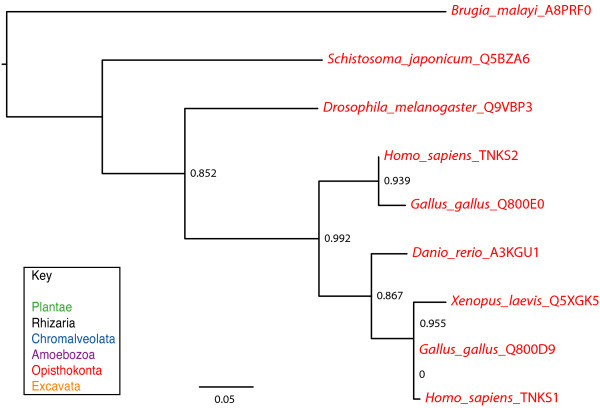
**Phylogenetic analysis of Clade 4 PARP genes**. The represented tree is a ML tree. This tree is based on a multiple alignment that includes the PARP catalytic domain (Additional file 9). Branch supports as in Figure 1. Scale bar indicates genetic distance reflected in branch length.

Clade 4 proteins are characterized by fifteen to eighteen ankyrin repeats followed by a sterile alpha motif (SAM), most likely a protein-protein interaction domain [76], and the PARP catalytic domain (Figure 4). These proteins are so similar to one another that we have not further subdivided them (Figure 8 and Additional file 9). The two human members of this clade, tankyrase1 and tankyrase2, have been shown to have poly(ADP-ribosyl)ation activity [19,27,77]. All proteins grouped in this clade retain the "HYE" catalytic triad (Figure 1 and Additional file 8), suggesting that they are likely to be active enzymes.

Our analysis indicates true tankyrases are confined to animals, and in fact do not appear to be found outside of the bilateria (Figures 2 and 8). A duplication event that generated two tankyrase-encoding genes appears to have occurred within the vertebrates, sometime after the separation of the amphibians. The absence of tankyrase orthologs outside of the animals contradicts the report of such proteins in protozoa such as *Dictyostelium discoideum *and *Tetrahymena thermophila *[78]. However, these protozoan proteins differ from the canonical tankyrases in structure; although they have ankyrin repeats in their N terminal region, these are followed by WGR and PRD domains rather than a SAM motif (Figure 4). Consistent with the presence of the WGR and PRD domains and the low similarity between their PARP catalytic domain and that of tankyrases, these proteins fall into Clade 1A (Figure 3). This suggests that PARP proteins independently acquired ankyrin repeats at least twice.

### Clade 5: The vPARP clade

**Figure 9:**
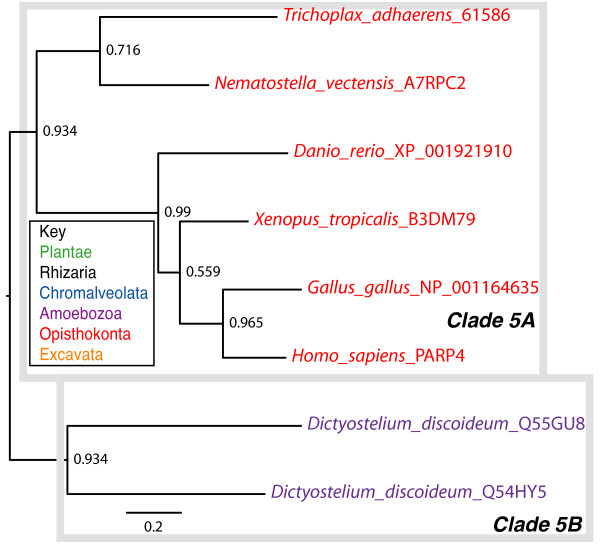
**Phylogenetic analysis of Clade 5 PARP genes**. The represented tree is a ML tree. This tree is based on a multiple alignment that includes the PARP catalytic domain (Additional file 10). Clade 5 proteins can be divided into two subclades, A-B, as indicated. Branch supports as in Figure 1. Scale bar indicates genetic distance reflected in branch length.

Clade 5 is found only in the Opishthokonts (animals) and Amboezoa (Figure 9 and Additional file 10) and is characterized by the position of the PARP catalytic domain. In this group, the PARP signature is found in the middle of the protein, rather than at the C terminus and is typified by human vPARP/PARP4. vPARP has the catalytic domain preceded by a BRCT domain and followed by a vault protein inter-alpha-trypsin (VIT) domain, and a von Willebrand factor type A domain (vWA) (Figure 4; [30]). Both VIT and vWA domains are commonly found in proteins of multiprotein complexes and are structurally related to each other [79]. Clade 5 is further subdivided into two subclades (Figure 9). Clade 5A contains animal proteins while Clade 5B contains two proteins from the amoeba *Dictyostelium discoideum *(Q54HY5 and Q55GU8). The amoeba proteins have a different protein structure than the animal members of this clade; they too have BRCT domains N terminal to their PARP catalytic domains and long C terminal extensions. However, there are no VIT or vWA domains found in these proteins.

vPARP is associated with vaults, very large cytoplasmic ribonucleoprotein particles first described in the 1980s whose function is unclear [80]. Vaults have a patchy taxonomic distribution within eukaryotes. Our analysis suggests that the phylogenetic distribution of vPARP is also limited (Figures 2 and 9); members of Clade 5A with the vPARP domain structure are found only in animals that have been shown to contain vaults, while Clade 5B proteins are found in Dictyostelium, which also contains vaults [81]. However, although vaults have been identified in trypanosomes [82], no evidence of proteins sharing the domain structure of vPARP can be found in this group of organisms, although such proteins may be present in species with currently unsequenced genomes.

### mART activity may be ancient

**Figure 10:**
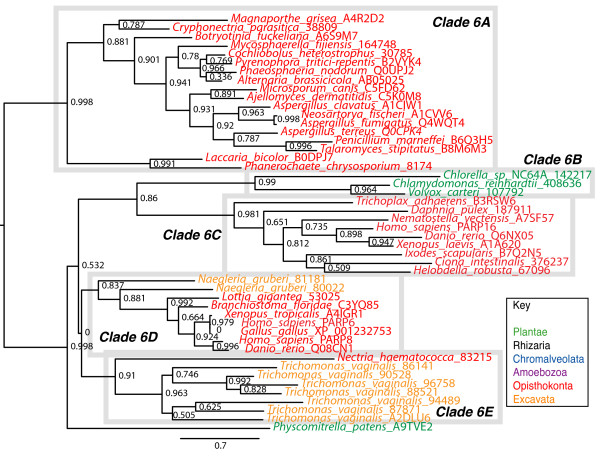
**Phylogenetic analysis of Clade 6 PARP genes**. The represented tree is a ML tree. This tree is based on a multiple alignment that includes the PARP catalytic domain (Additional file 11). Clade 6 can be divided into five subclades, A-E, as indicated. Branch supports as in Figure 1. Scale bar indicates genetic distance reflected in branch length.

Clade 6 proteins are found in Opisthokonts (animals and fungi), Excavates (Parabasalids and Heterolobosa), and Plantae (chlorophyta and bryophytes) (Figures 1, 2 and 10 and Additional file 11). Based on its position as sister group to all other clades of PARPs (Figure 1) and the distribution of species containing Clade 6 PARPs within the eukaryotes (Figure 2), it is likely that the last common eukaryotic ancestor had at least one Clade 6-like protein encoded in its genome. This clade is characterized by N termini with no known functional domains and C terminal extensions beyond the PARP catalytic domain of varying lengths. Almost all of these proteins contain a PfamB_2311 domain immediately before their PARP catalytic domain (Figure 4), although the function or significance of this domain is unknown, supporting the placement of these proteins in a single clade. Another characteristic of Clade 6 members is changes within the PARP catalytic domain. None of the Clade 6 proteins we identified contain the final glutamic acid of the HYE catalytic triad, although they mostly retain the histidine and tyrosine (Additional file 11). This might lead to an inability to catalyze poly(ADP-ribosyl)ation. In fact, the human proteins in this clade (PARP6, 8, and 16) have been predicted to have mono(ADP-ribosyl)ation activity based on structural models [32], although this awaits experimental confirmation. None of the Clade 6 PARPs have been functionally characterized.

Clade 6 can be subdivided into five groups (6A-E; Figure 10). Clade 6A contains fungal proteins exclusively (Figure 10; [40,83]). These proteins consist of a long N terminal region containing no known functional domains, a PfamB_2311 domain, the PARP catalytic domain, and a C terminal extension containing an UBCc (Figure 4 and Additional files 12 and 13). The UBCc domain is the catalytic domain contained in E2 Ub-conjugating enzymes (UBCs) [84]. These enzymes carry Ub and transfer it either directly to a substrate in cooperation with an E3 enzyme or to the E3 Ub-ligase. An active cysteine residue [84] characterizes the UBCc domain and is found in Clade 6A proteins (Additional files 12 and 13A-B). In addition, these proteins also share a number of residues conserved across a range of UBCc and UBCc-like domains (Additional files 12 and 13A-B). These include the residues making up the proline-hydrophobic side chain interaction at the top of the so-called E2 fold flap, and a chain of interacting residues at the bottom of the flap (see bolded residues in Additional file 13A-B). These residues have been implicated in the mechanical structure of the E2 fold [85]. Although it is unusual for E2 enzymes to have multiple functional domains, there is at least one other family of such enzymes, the BRUCE-like family, which has multiple domains. These proteins are large (between four and five thousand amino acids) and contain Baculovirus Inhibitor of apoptosis Repeats (BIR; [86,87]) in their N termini, followed by a large region of unknown function, and a UBCc domain at their C termini [88].

No other known functional domains can be identified in Clade 6A proteins; however, most of these proteins do share another PfamB domain, 30617, at their very N termini [41]. This domain is confined to fungal species and appears to only occur in Clade 6A family members with the exception of a protein from the fungus *Uncinocarpus reesii *(EEP82442.1) that consists only of this domain (Additional file 14). Pfam-B_30617 averages 360 amino acids in length and has some secondary structure similarity to the RWD domain when modelled using the Protein Homology/Analogy Recognition Engine (Phyre; [89]), and is predicted to form an alpha helix/beta strand/alpha helix/beta strand/alpha helix structure (Additional file 13C). The RWA domain has some structural similarity to the UBCc domain [90], further providing a link between the Clade 6A proteins and Ub. The RWA domain is thought to mediate non-catalytic protein-protein interactions. We propose renaming the Pfam-B_30617 domain FPE, for Fungal PARP E2-associated.

Clade 6B proteins are found in a subset of green algae (Figure 10). These proteins have no other domains of known function but do contain PfamB_2311 domains as well as the PARP catalytic domain. Green algae have not previously been shown to have any PARP-like proteins encoded in their genomes. Clade 6C proteins are animal specific and are found in species from across this group, including human (PARP16; Figure 10). Again, other than a PfamB_2311 domain and a PARP catalytic domain, no other obvious protein motifs are present. Clade 6D is confined to Deuterostomes with the exception of the mollusc *Lottia gigantea*. These proteins consist of no identifiable domains other than a PfamB_2311 domain and the PARP catalytic domain (Figure 4). Human PARP6 and PARP8 are found within this group of proteins.

Clade 6E consist of seven proteins encoded by *Trichomonas vaginalis*, the only member of the Parabasalids (Excavata) with a fully sequenced genome and one fungal protein (*Nectria haematocca *83215). Trichomonas is the causative agent of the sexually transmitted disease trichomoniasis in humans; without other completed genomes available for the parabasalids, it is impossible to determine if members of Clade 6E are found elsewhere in this group. Besides the PARP catalytic domain, the only other identified domain in these proteins is a PfamB_2311 domain. The *Nectria haematocca *protein does not have a PfamB_2311 domain or any known functional domain.

### Phylogenetic analysis suggest multiple independent losses of PARP genes across the eukaryotes

Although the five supergroups of eukaryotes with genome information contain organisms with PARP-encoding genes in their genome, some lineages appear to have lost all PARP genes (Figure 2 and Table 1). For example, in Plantae the sequenced genomes available for three red algae and a subset of green algae do not encode any PARP genes (Table 1), although it is possible that such genes may be present in other species not yet sequenced. The complement of PARP proteins present can differ even between closely related species; for example, the green algae *Chlorella sp. NC64A *contains a Clade 6 PARP representative while *Chlorella vulgaris *does not (Figure 2 and Table 1). Diatoms and brown algae (members of the Chromalveolates) do not appear to have PARPs, nor do the sequenced members of the Excavates group Diplomonads. While the sequenced species represent only a small amount of the diversity in these groups of organisms, the lack of PARP genes suggests that these lineages have lost PARPs and, further, demonstrate that these genes are not absolutely essential for eukaryotic life.

**Table 1:** Eukaryotic organisms with no identifiable PARP genes in their nuclear genomes.

Eukaryotic Supergroup	Rank	Species	Citation or sequencing group
Plantae			
	Chlorophyta		
		Chlorella vulgaris	JGI
		Ostreococcus lucimarinus	[162]
		Micromonas pusilla	JGI
	Red algae		
		Guillardia theta	JGI
		Hemiselmis andersenii	NCBI
		Cyanidioschyzon merolae	[163]
Opisthokonts			
	Fungi		
		Saccharomyces cerevisiae	[164]
		Schizosaccharomyces pombe	[165]
		Pichia stipitis	[166]
		Heterobasidion annosum	JGI
		Melampsora laricis-populina	JGI
		Phycomyces blakesleeanus	JGI
		Candida albicans	[167]
		Sporobolomyces roseus	JGI
Chromalveolates			
	Diatoms		
		Phaeodactylum tricornutum	[168]
		Thalassiosira pseudonana	[169]
	Brown algae		
		Aureococcus anophagefferens	JGI
Excavates			
	Diplomonads		
		Giardia intestinalis	[170]
		Spironucleus vortens	JGI
			

Species listed have had their genomes individually searched by BLAST to determine if any PARP proteins were present. NCBI, National Center for Biotechnology Information; JGI, Department of Energy Joint Genome Institute.

**Figure 11:**
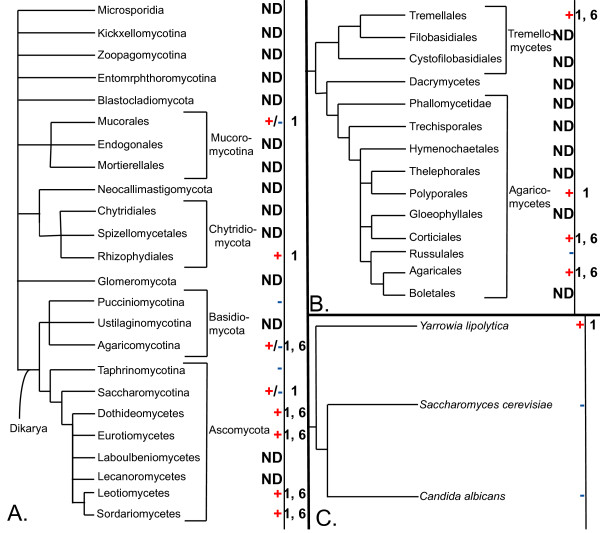
**Multiple independent losses of PARP genes have taken place in the fungal lineage**. A. Phylogeny of the fungi. The topology of the schematic tree is based on a recent higher order examination of the fungi [159]. B. Simplified phylogeny of the Agarimycotina. This tree is based on that of [160]. C. Simplified phylogeny of the Saccharomycotina, based on that of [161]. Branch lengths are not proportional to genetic distance in any of the phylogenetic trees. Presence or absence of PARP proteins are indicated by a red + or a blue -, respectively. Groups in which some species have lost PARPs while others have retained them are indicated by a +/- symbol. For each species or group, PARP family members are listed with the clade numbers introduced in Figure 1. Accession numbers of the genes and details on the source of data for individual taxa is provided in Additional file 1 and Table 1. ND, no data.

The fungal lineages within the Opisthokonts provide a particularly interesting pattern of gene loss. This group of organisms contain Clade 1 and 6 PARP proteins, and based on the phylogenetic distribution of these genes, the fungal ancestor contained proteins representing both clades (Figure 2). However, not all current fungal groups or species have both types of PARPs and some do not encode PARP genes at all (Figure 11A and Table 1). For example, the two major model fungal species, *Saccharomyces cerevisiae *and *Schizosaccharomyces pombe*, do not have PARPs. It appears that there have been at least five independent losses of PARPs within the fungi. The basal fungi are not well represented by sequenced genomes, however within the Mucorales the genomes of three species have been sequenced and two have Clade 1 PARPs (*Rhizopus oryzae *and *Mucor circinelloides*) while the other has none (*Phycomyces blakesleeanus*). The Basidiomycota has had at least two losses of PARPs; one loss has occurred within the Pucciniomycotina and one within the Agaricomycotina. Only two species within the Pucciniomycotina are represented in our analysis and neither encodes PARP proteins (Table 1). Within the Agaricomycotina, there appear to have been two losses of PARPs. Both Clade 1 and 6 PARPs are found in some species within this group of Basidiomycota; however, *Postia placenta *(Polyporales) has retained only a Clade 1 PARP while *Heterobasidion annosum *(Russulales) has lost both types of PARPs (Figure 11B). The Ascomycota are the fungal group including the most species with sequenced genomes and have both Clade 1 and 6 PARPs (Figure 11A). This group has seen at least two independent losses of PARPs. The Taphrinomycotina (represented by *Schizosaccharomyces pombe*) contain no PARP genes while none of the Saccharomycotina has Clade 6 proteins and only a basal member of this group, *Yarrowia lipolytica, *retains Clade 1 proteins (Figure 11C). Interestingly, as previously noted by other groups [91], PARPs or PARP-like proteins are mostly retained in fungi that have multicellular hyphae and/or elaborate developmental programs, but not in yeasts (Figure 11).

## Discussion

### Evolutionary history of the PARP family

**Figure 12:**
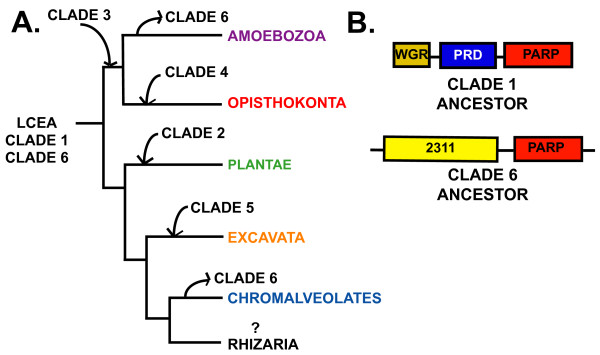
**General summary of the evolutionary hypothesis of the PARP enzyme family**. A. Simplified phylogeny of the eukaryotes. The last common eukaryotic ancestor (LCEA) contained Clades 1 and 6 PARPs. Subsequently, Clade 6 PARPs were lost in two supergroups (Amoebozoa and Chromalveolates), while individual supergroups gained novel clades of PARPs. Branch lengths do not reflect genetic distance. B. Schematic representation of the domain structure of ancestral PARP proteins present in the LCEA. WGR, WGR domain; PRD, PARP Regulatory Domain; PARP, PARP catalytic domain; 2311, PfamB_2311.

The broad distribution of PARPs across the eukaryotes indicates that the last common eukaryotic ancestor (LCEA) had genes encoding members of this protein family. Clade 1 PARPs are found in all five eukaryotic supergroups for which sequence information is available; this implies that the LCEA encoded at least one enzyme of this type, and may have had multiple members (Figures 2 and 12A). Based on the domain structure of modern Clade 1 proteins, we hypothesize that the Clade 1 enzyme or enzymes found in the LCEA consisted of WGR, PRD, and PARP catalytic domains (Figure 12B).

Members of Clade 1 have been characterized in a range of organisms, encompassing three of the six eukaryotic supergroups. While a wide range of functions has been described for these PARPs, most characterized members of Clade 1 have been implicated in or demonstrated to have roles in DNA damage response and repair. In Plantae, two of the *Arabidopsis thaliana *Clade 1 members, *AtPARP1 *and *AtPARP2*, have been shown to be induced by DNA damage and be involved in the response to it [92,93]. In the Opisthokonts, several animal Clade 1 members have been investigated and shown to be involved in DNA repair. This is a well-known function for the human Clade 1 members, PARP1, PARP2, and PARP3 [26,94,95]. In addition, a fungal protein, PrpA from *Aspergillus nidulans*, has been shown to act early in the DNA damage response [91], while loss of its ortholog from *Neurospora crassa*, NPO, causes sensitivity to DNA damage and acceleration of replicative aging [83]. Within the Excavates, a *Trypanosoma cruzi *Clade 1 member, TcPARP, has been shown to be induced in response by DNA damage, be enzymatically activated by nicked DNA and to require DNA for catalytic activity [96]. Clade 1 members in the Chromalveolates and the Amoebozoa have not been functionally characterized, but are also likely to function in DNA damage response. *Dictyostelium discoideum *in the Amoebozoa has at least four Clade 1 proteins encoded in its genome (Figure 3). Drug studies have implicated PARP activity in oxidative stress response and DNA damage in this organism [97], but no direct evidence of which PARP or PARPs is involved has been published. The ubiquitous distribution of Clade 1 members and the consistent association of the proteins with DNA damage response suggests that this gene lineage is ancient and that the original function of this family was in DNA repair and genome integrity.

While Clade 6 is found in only three of the five eukaryotic supergroups with available genome information (Opisthokonta, Excavata, and Plantae), the phylogenetic relationship of these groups within eukaryotes suggests that a Clade 6-like protein was found in the LCEA (Figures 2 and 12A). Subsequently, during the eukaryotic radiation, Amoebozoa (or at least *Dictyostelium discoideum*) and Chromalveolates lost Clade 6 PARPs. The ancestral Clade 6 protein was likely to consist of a PfamB_2311 domain N terminal to the PARP catalytic domain (Figure 12B). Members of Clade 6 were more difficult to identify than other PARPs; it was necessary to do supplemental BLAST searches with the human PARP6 catalytic domain to find most of these proteins (see Methods). This is consistent with the positioning of Clade 6 as sister group to the rest of the PARP superfamily. The fact that Clade 6 PARPs represent an ancient lineage further suggests that changes in the PARP catalytic domain likely to eliminate or change enzymatic activity evolved early in this protein family or, alternatively, PARP activity evolved from mART activity. It is difficult to speculate on the possible function of the Clade 6 ancestral protein, as none of the extant Clade 6 members have been functionally characterized.

One group of PARPs defined in our study has an unusual distribution. Clade 3 is found in animals (Opisthokonta), *Dictylostelium discoideum *(Amoebozoa) and the ciliate *Tetrahymena thermophila *(Chromalveolates), but no other species in our analysis, including the ciliate *Paramecium tetraurelia*. Our phylogenetic tree is based on the PARP catalytic domain. Clade 3 proteins have evolved to become either mARTs or non-enzymatic (Figure 7; [32]). We propose that the grouping of the Tetrahymena proteins in Clade 3 is an artefact caused by this group of proteins independently beginning to evolve similar changes in the PARP catalytic domain. Clades 3 and 6 independently acquired somewhat similar changes, supporting the idea that changes within the PARP catalytic domain may be constrained in order to preserve overall structure. The hypothesis that the Tetrahymena proteins are not closely related to the other Clade 3 proteins is supported by the fact that one of them (Q22F17) retains the glutamic acid of the PARP catalytic triad, while another (Q24C77) has a conservative substitution of a glutamine at that position and that they do not share any domains outside of the catalytic domain with other members of Clade 3. When more sequences within the ciliates become available, it may become possible to determine if this hypothesis is correct. The Dictyostelium proteins found in Clade 3 may be orthologous to the animal proteins, since one of them has a Macro domain, a domain found in other members of this clade (Figure 4).

In extant eukaryotes, the animal lineage within Opisthokonta appears to have the most diverse collection of PARPs. Most animal genomes encode representatives of at least two clades of PARPs. In addition, a PARP clade has been acquired in this lineage, Clade 4 (Figure 12A). Vertebrates contain the highest number and type of PARPs of any group examined within the eukaryotes, containing members of Clades 1, 3, 4, 5 and 6; additionally they often encode more than one representative of each clade. However, within animals the nematodes are unusual. *C. elegans*, within the order Rhabditida, only encodes two Clade 1I proteins, PME1 and PME2 (Figure 3), and a protein (PME5) that did not clearly fall into any clade (Additional file 4). Within Clade 1, the nematode 1I PARPs do not group with other animal PARPs but rather are found as the sister group to all of the Clade 1 proteins. PME5 somewhat resembles tankyrases in domain structure but does not group with them. However, the branches leading to the *C. elegans *proteins are long. The length of these branches likely results in long-branch effects, causing misplacement of these proteins within the tree. Such long-branch effects can be caused by the independent acquisition of identical character states [98], phylogenetic signal erosion ("long branch repulsion") [99], or by symplesiomorphy (retention of an old conserved character state) [100]. In contrast to the situation in *C. elegans*, we were unable to identify any Clade 1 PARPs in the nematode *Brugia malayi*, in the order Spirudida, but did identify a clear tankyrase (Figures 2, 3 and 8). The nematodes are clearly outliers within the animal lineage and a closer examination of the PARP family across a greater number of such species would be interesting.

Although PARPs are found throughout the eukaryotes, these proteins are not essential for eukaryotic life. This is illustrated most clearly in the fungal lineage within the Opisthokonta. In contrast to their fellow Opisthokont lineage the animals, fungi encode members of only Clades 1 and 6 PARPs (Figures 2 and 11). Lineages within the fungi have independently lost PARPs at least five times, illustrating that eukaryotic organisms do not absolutely require this family of proteins. In addition, it should be noted that none of the fungal species examined retained Clade 6 PARPs in the absence of Clade 1 PARPs. This underscores the relative importance of the so-called "classical" Clade 1 PARPs in these organisms. Interestingly, many of the fungi that have lost all PARPs, including the model fungal systems *Saccharomyces cerevisiae *and *Schizosaccharomyces pombe*, are yeasts. This suggests fungi with more complex life cycles may retain PARPs more readily than yeasts do. It is possible that a selective advantage is found in organisms with relatively rapid generation times in dispensing with this class of proteins. This is supported by the retention of Clade 1 PARPs in the basal Saccharomycia fungus *Yarrowia lipolytica *while the two other sequenced members of this fungal group have lost all PARPs (Figure 11C). Yarrowia can grow in three forms: as yeast, hyphae and pseudohyphae. *Candida albicans*, also a Saccharomyces member, is trimorphic but lacks PARPs; however, this diploid organism lacks a known sexual cycle, suggesting a simplification of its life cycle. *Sacchromyces cerevisiae *is only dimorphic, growing only as yeast or pseudohyphae (reviewed in [101]). Other groups have noted the association of retention of PARPs with filamentous growth [91]. This correlation is also found in the dimorphic human pathogen *Histoplasma capsulatum*, the cause of histoplasmosis, which grows as either yeast or hyphae. In this organism, we have found that its Clade 6A PARP gene is expressed only during the filamentous growth stage and not when the fungus is growing in the yeast form (Lee and Lamb, data not shown).

Our conclusions about the function and distribution of PARP proteins in the eukaryotes are limited by the availability of species with sequenced genomes. Currently, there is a dearth of sequences available in many groups of eukaryotes while animals, particularly vertebrates, and fungi are relatively well represented. A number of phylogenetically important groups such as streptophyte algae, glaucophytes, phaeophytes, dinoflagellates, and archamoebe have no sequenced genomes. The eukaryotic supergroup Amoebozoa is represented by only one species, *Dictyostelium discoideum*, while there are no representatives of Rhizaria sequenced. Despite the limitations of the available sequences, we have identified unique types of PARPs in *Naegleria gruberi*, *Trichomonas vaginalis *and green algae and clarified the phylogenetic distribution of tankyrases. There are likely to be additional variations of PARPs discovered as more eukaryotic genomes are sequenced and a further advancement of our understanding of evolution of this important proteins superfamily.

### Clade 5 and vaults

The Clade 5 PARPs have a limited phylogenetic distribution, found only in a subset of animals and amoeba (Figure 9). vPARP was originally identified in a two-hybrid screen using the major vault protein (MVP) protein as bait and shown to act as a *bona fide *PARP [30]. vPARP associates not only with the ribonucleoprotein vault complex, but also can be found in the nucleus, associated with the telomere and the mitotic spindle. The function of vPARP at any of its locations is unclear. Vaults have been best studied in mammals and in these organisms are composed of three proteins, MVP, TEL1 (also found at telomeres), and vPARP. In addition, several vault specific RNAs (vRNAs) are found. The function or functions of vaults are still unclear; they are associated with drug resistance and several signalling pathways (reviewed in [102]), as well as the nuclear pore complex [103,104]. vPARP-deficient mice are normal and fertile with no defects in telomeres or vaults [105]. More recently these mice have been found to develop more tumours in response to carcinogens, suggesting a role in chemically induced cancers [106].

Vaults have been identified in diverse animals and in other eukaryotes such as the amoeba *Dictyostelium discoideum*, flatworms, and trypanosomatides [81,82]. However, vaults appear to be missing from fungi, a number of model animals (*C. elegans *and *Drosophila melanogaster*) and in plants [107-109].

The fact that vPARP does not appear essential for normal development or vault structure in mouse [105] suggests that this protein is not essential for vault function. This may explain why organisms that have been demonstrated to contain vaults in their cells do not always encode proteins that look like vPARP.

### Clade 2 plant-specific PARPs are involved in stress responses

In addition to containing three Clade 1 PARPs throughout and Clade 6 PARPs only in the bryophytes, the land plants contain a unique clade of PARP-like proteins. This clade can be subdivided into two subclades, one of which contains proteins with an N terminal WWE domain. Clade 2 is distinct from Clade 3, which also contains proteins with WWE domains. A group within Clade 2, confined to the eudicots within the angiosperms, consists of truncated proteins lacking the N terminal WWE domain. Examination of the phylogeny of Clade 2 clearly illustrates the importance of genome duplication during plant evolution [110-112]; plant species tend to encode gene pairs (Figure 5).

The plant Clade 2 proteins have only been investigated in the model angiosperm *Arabidopsis thaliana*. Arabidopsis has two genes, *RCD1 *and *SRO1, *which encode full-length members of Clade 2A [64,113]. *RCD1 *was originally identified as a stress response gene [63]. It is involved in the response to several abiotic stresses and shows altered hormone accumulation and gene expression [64,114,115]. *rcd1 *mutants also display pleiotropic developmental defects including reduced stature, malformed leaves, and early flowering [64]. Loss of *SRO1 *causes only minor defects; however *rcd1; sro1 *double mutants are severely affected with a majority of individuals dying during embryogenesis [65,69], indicating that this clade of PARP proteins has essential functions in land plants. RCD1 has been shown to bind to a number of transcription factors, suggesting that Clade 2 PARPs may function in transcriptional regulation [69,113]. RCD1 does not appear to have catalytic activity, consistent with the absence of the HYE catalytic triad in this protein (Figure 1 and Additional file 7); however, other members of this clade do contain variant HYE motifs that may confer activity (Additional file 7). Therefore, it will be necessary to test individual members of this clade for activity.

Four genes in Arabidopsis, *SRO2-5*, encode proteins within Clade 2 that lack the N terminal WWE domain [64,113] and consist of two gene pairs: *SRO2/SRO3 *and *SRO4/SRO5 *(Figure 5). These genes may be involved in stress signalling; *SRO5 *is necessary for response to both salt and oxidative stress [66] and can bind transcription factors [62] and *SRO2 *is up regulated in chloroplastic ascorbic peroxidase mutants [116].

### Multiple independent acquisitions of mART activity within the PARP superfamily

Although not closely evolutionarily related (Figure 1), the proteins belonging to Clades 3 and 6 have modified their catalytic domains, replacing the glutamic acid of the "HYE" catalytic triad with various other amino acids (Figure 7 and Additional files 8 and 11). The catalytic activity of several human members of Clade 3 has been experimentally investigated. PARP10, which falls into Clade 3A and has an isoleucine instead of a glutamic acid in its catalytic site, has been reported to have auto(ADP-ribosyl)ation activity and modify core histones [33,34]. More recently it was shown to have mono(ADP-ribosyl)ation activity, not poly(ADP-ribosyl)ation activity, and therefore function as a mono(ADP-ribosyl) transferase (mART) rather than a PARP [32]. Molecular modelling suggested that this enzyme uses substrate-assisted catalysis in order to activate the NAD^+ ^substrate. This group further demonstrated that PARP14/BAL2, a Clade 3C member with a leucine in place of the glutamic acid, also has mART activity, consistent with an earlier paper demonstrating auto(ADP-ribosyl)ation activity [35]. A human member of Clade 3F, PARP9/BAL1, has not only replaced the glutamic acid within the catalytic PARP signature but have also replaced the histidine (with a glutamic acid). This enzyme has been shown to be inactive [32,35]. Almost all of the proteins comprising both Clade 3 and Clade 6 have replaced at least the glutamic acid of the "HYE" triad. It is likely that none of these proteins function as bone fide PARPs but rather are either mARTs or are no longer enzymatically active. Clade 3 has a limited taxonomic distribution (Figures 2 and 6); Clade 6, on the other hand, is found in at least three of the six eukaryotic supergroups and was likely present in the LCEA (Figure 12A). This suggests that the evolution of mART activity within the PARP gene family occurred before the full complement of crown groups had formed. In addition, the changes in the catalytic domain of the Clade 2 proteins also suggest that these proteins have altered enzymatic activities (Additional file 6). Therefore, it is likely that mART activity and/or loss of enzymatic activity has evolved at least twice from PARP activity (in Clades 3 and 2) and that mART activity in extant Clade 6 proteins represents an even earlier acquisition of this enzymatic activity.

What functions do PARP-like/mART proteins play? While no members of Clade 6 have been characterized, several members of Clade 3 have, all in mammalian systems. PARP9/BAL1, PARP14/BAL2, and PARP15/BAL3 have been shown to interact with transcription factors and mediate transcriptional repression or activation [35,75,117,118]. PARP13/ZCC2/ZAP has been shown to bind to viral RNA through its zinc fingers and promote degradation of the RNA by the exosome [119-124]. PARP12 shares significant similarity to PARP13 and is thought to function similarly. PARP10 interacts with MYC and inhibits transformation; its overexpression leads to a loss of cell viability [33,34]. To date, no clear consensus about the function of Clade 3 proteins can be formulated.

### True tankyrases are confined to animals

Human tankyrase1 was originally identified as a telomeric protein interacting with TRF1, a negative regulator of telomere length. It was shown to act as a PARP and automodify itself as well as TRF1 [19]. A second human tankyrase, tankyrase2 (Figure 4), was identified shortly after the initial discovery of tankyrase1 [28,29,125]. Human tankyrases can be found both in the nucleus [19], at the nuclear pore and centrosome [126], and in the cytoplasm associated with the Golgi or vesicles [127] or the plasma membrane [128]. Since their initial discovery, the known functions of these proteins have expanded to include spindle assembly and vesicle trafficking (reviewed in [78]), sister chromatid segregation [129], and regulation of the WNT pathway [130-132]. Tankyrases have been identified in a number of animal species, including mouse. In this model organism, it appears tankyrase may not function in telomere length control [133], but its other functions are conserved and its function is essential [134]. Consistent with functions outside of the telomere, a tankyrase is found in *Drosophila melanogaster *(Figure 8; [78]), an organism with a highly divergent telomere consisting of transposons rather than the short repeats found in other eukaryotes [135].

Our phylogenetic tree places a number of proteins previously reported as tankyrases in Clade 1, rather than within Clade 4 (Figures 3 and 8). These proteins do have a different domain structure than tankyrases, sharing ankyrin repeats with tankyrases but having WGR and PRD domains rather than SAM motifs (Figure 4). It is likely that the Clade 1 ankyrin repeat proteins do not share functions with tankyrases.

PME5 from *C. elegans *was reported as a tankyrase and has been functionally characterized. As mentioned above, this protein does not clearly group with any clade, including Clade 4 (Additional file 4). In the original paper describing PME5, it was shown to be more closely related to a *Dictyostelium discoideum *protein we have placed in Clade 1A (Q54E42) and to have a higher similarity within the catalytic domain to human PARP1 than human tankyrase [54]. In addition, the induction of *PME5 *expression by DNA damaging agents, the increased apoptosis in *pme5(RNAi) *lines after DNA-damage, and the constitutively nuclear chromatin-associated localization of PME5 [53,136] is more consistent with a role in DNA damage. However, the difficulty in placing *C. elegans *PARPs into clades complicates the issue. Further work will need to be done to determine the function of PME5.

### Connections between ubiquitination, SUMOylation and poly(ADP-ribosyl)ation

The attachment of ubiquitin to proteins is an important mechanism in regulating many cellular processes. Similarly to ADP-ribosylation, one to many ubiquitin units can be added to proteins, although only on lysine resides. A chain consisting of at least four ubiquitin linked together by Lys48 residues causes destruction of the protein via the 26S proteasome [137,138], while either monubiquitination or polyubiquitination with chains linked at Lys63 serve as nonproteolytic signals in such processes as trafficking, DNA repair, and signal transduction [139,140]. Ubiquitination of proteins involves an enzymatic cascade involving ubiqutin-activating (E1), ubiquitin-conjugating (E2), and ubiquitin-ligating (E3) enzymes.

A number of connections between PARP proteins and ubiquitination have emerged. One connection involves the fact that both attachment of ubiquitin and ADP-ribose can be made at lysine residues, suggesting that these post-translational modifications could compete for substrates. In addition, several protein domains found in PARP proteins can also be found in proteins associated with the ubiquitin system (Figure 4). For example, many Clade 1 proteins have BRCT domains; these domains were originally identified in the BRCA1 protein. BRCA1 functions as an E3 ligase in a multi-protein complex in response to DNA damage [141-143]. Within Clade 6, Clade 6A proteins have a UBCc domain, similar to that found in ubiquitin E2s [144], at their C termini, as well as FPE domains at their N termini (Additional Figures 12, 13 and 14). This novel domain has some similarity to the RWD domain, which in turn is related to the UBCc domain, although thought to be non-catalytic. WWE domains are found in Clade 2 and 3 proteins and also in certain ubiquitin E3 ligases [51]. Some Clade 3 proteins have UIM domains, which can bind ubiquitin and polyubiquitin chains [145]; this domain is also found in the BRCA1-interacting protein Rap80 [141]. The *Dictyostelium discoideum *protein DDB0393590 contains a U-box (Figure 4), found in E3 ubiqutin ligases and known to bind E2 enzymes [74].

In addition to the structural similarities found between PARPs and classes of Ub enzymes, some functional connections are also known. Human PARP14/BAL2, a Clade 3E member, has been shown to bind to the multifunctional phosphoglucose isomerase/autocrine motility factor (PGI/AMF). This binding inhibits polyubiquitination of PGI/AMF, stabilizing the protein [146]. PARP1 in humans is regulated by ubiquitination [147] and has been shown to bind to the E2 enzyme hUBC9 [148]. Proteasome-mediated proteolysis of ubiquitinated tankyrase has also been documented; this is promoted by the auto-poly(ADP-ribosyl)ation of tankyrase, which releases the protein into the cytoplasm [128]. This is similar to the mechanism whereby tankyrase poly(ADP-ribosyl)ates the telomeric protein TRF1, releasing it from the telomere, allowing its ubiquitination and degradation [149] and the regulation of axin by tankyrase [130]. There are likely to be more connections found in the future between post-translational ADP-ribosylation and ubiquitination.

Recently, a connection between poly(ADP-ribosyl)ation and SUMOylation has also been demonstrated. PARP1 itself is SUMOylated [150,151], and this takes place within its automodification domain and does not regulate poly(ADP-ribosyl)ation activity [150]. Rather, PARP1's transcriptional co-activator activity is modified [150,151]. PARP1 can also form higher order complexes and influence SUMOylation of other proteins. In response to both heat shock and DNA damage, human PARP1 associates with the SUMO E3 ligase PIASy [151,152] and this requires a PAR-binding motif in this protein [152]. Upon DNA damage, PIASy associates with PAR on PARP1 and subsequently its target NEMO binds and is SUMOylated by PIASy, leading to NF-kappaB activation [152]. Clearly, the interplay between poly(ADP-ribosyl)ation and other post-translational modifications is just beginning to be explored.

## Conclusions

We present here a large-scale phylogenetic analysis of the PARP gene family that extends previous examination of this family. Several main conclusions can be drawn from our study. First, the phylogenetic distribution of the PARP protein family is tremendously broad across the eukaryotes, consistent with the last common ancestor of modern eukaryotes containing at least two PARP-encoding genes. Second, two types of PARP-like proteins were present in the LCEA; one likely functioned in DNA repair and genomic maintenance and resembled modern members of Clade 1. The second probably had mART activity. Third, increasing numbers and types of PARP-like protein are likely to be found as more eukaryotic organisms have their genomes sequenced.

## Methods

### Retrieval of the PARP gene sequences

The initial sequence set was selected from the Pfam database (http://pfam.sanger.ac.uk/; [41-43]), using the sequences identified as members of the PARP family (PF00644). The full sequences of the proteins were retrieved from UniProt [44,45], using the links provided by Pfam. Additional sequences were retrieved from other eukaryotic organisms at the DOE Joint Genome Institute (JGI; http://www.jgi.doe.gov/), the Broad Institute http://www.broadinstitute.org, the J. Craig Venter Institute http://www.jcvi.org/, ToxoDB (http://toxodb.org/toxo/; [47]), and the Arabidopsis Information Resource (TAIR; http://www.arabidopsis.org/) using BLAST searches [46] based on human or *Arabidopsis thaliana *PARP catalytic domain sequences as search queries. Specific phylogenetically interesting genomes were also individually searched by BLAST to confirm the absence of PARP proteins (see Table 1). The catalytic domains of most retrieved sequences were delineated using Pfam. Sequences in Clade 6 have lower similarity to the classical PARPs (i.e. Clade 1) used to generate the Pfam HMM, so the PARP catalytic domains for these sequences were identified using BLAST searches based on human PARP6 catalytic domain as the query and identifying the region of retrieved sequences that had similarity to this PARP signature. In addition, many sequences whose catalytic domain was incompletely identified by Pfam were completed by BLAST searches using closely related complete PARP catalytic domains from other closely related species, in order to provide as much sequence information as possible for the alignment and phylogeny inference. The identified PARP catalytic domains were extracted using the extract.pl tool in the Wildcat Toolbox set of Perl utilities (http://proteomics.arizona.edu/wildcat_toolbox; [153]). Sequences of less than 100 amino acids in length and many that were missing important structural elements of the PARP domain were discarded to allow better alignment and phylogenetic signal recovery. Many of these sequences were obtained from shotgun sequencing and are presumably incomplete.

### Phylogenetic analyses

The collected PARP catalytic domains were aligned using the MUSCLE3.8.31 multiple alignment tool, using default settings [50]. The multiple alignment was subjected to a maximum-likelihood (ML) analysis using PhyML3.0 [154] using the computer facilities at the Ohio Supercomputer Center http://www.osc.edu. The substitution model parameters using for the PhyML analysis were the WAG substitution matrix, Γ8+I correction to model site rate heterogeneity and empirical equilibrium frequencies. These parameters were selected as the optimal substitution model based on analysis by ProtTest v2.4 [155]. A parsimony-based starting tree was used. Branch supports were computed in PhyML using an aLRT non-parametric Shimodaira-Hasegawa-like (SH) procedure [156]. Once a tree with all PARP domains had been generated, it was used to identify the six clades referred to in the text in combination with examination of domains outside of the PARP catalytic domain. After the six clades were defined, sequences from each clade were aligned separately using MUSCLE. These alignments were used to generate individual clade trees using PhyML with identical parameters. The phylogenetic trees were generated for figures using FigTree http://tree.bio.ed.ac.uk/software/figtree. Alignment figures were generated using TEXshade [157] and Jalview [158].

### Prediction of protein domains

After sequences of PARP family members were retrieved and placed into clades, the sequences were checked for other domains at the Pfam website [41]. Domains identified are shown in Figure 4. PfamB_30617 was identified in Clade 6A fungal proteins and extracted aligned as above. This domain was further analyzed using the Protein homology/analogy recognition engine (Phyre) [89] and renamed FPE (Fungal PARP E2-associated). Subsequently, a consensus FPE sequence was used in BLAST searches to find other proteins containing this region. The UBCc domains from Clade 6A proteins were similarly processed.

## Authors' contributions

MC retrieved the sequences, made the sequence alignments, and performed the phylogenetic analyses. RSL performed the domain analysis using Pfam and Phyre. ST, MC and RSL participated in data analysis and figure preparation. MC and RSL participated in the design and coordination of the study. RSL drafted the manuscript and all the authors participated in the editing of the manuscript. All the authors read and approved the final manuscript.

## Supplementary Material

Additional file 1 **List of all protein sequences used in our study**. The protein name is given as species followed by accession number. The eukaryotic supergroup, phylum, class and order are also indicated. The source of the sequence is given as well as the link to the sequence. The proteins are arranged alphabetically by species. Click here for file

Additional file 2 **Alignment of protein sequences removed from analysis due to incomplete PARP catalytic domains**. Click here for file

Additional file 3 **Multiple alignment of the PARP catalytic domain**. These alignments only show the conserved PARP catalytic domain. Only sequences containing complete domains were used to generate the alignment. Click here for file

Additional file 4 **Phylogenetic analysis of PARP genes in eukaryotes**. The represented tree is a ML tree, based on an alignment of the PARP catalytic domain. Branch supports as in Figure 1. Scale bar indicates genetic distance reflected in branch length. Click here for file

Additional file 5 **Multiple alignment of the PARP catalytic domains of Clade 1 PARP proteins**. These alignments only show the conserved PARP catalytic domain and the numbers indicate amino acids within the catalytic domain. Due to the large number of proteins in Clade 1, some needed to be removed in order to annotate the sequence. Dots indicate gaps introduced to optimize the alignment and identical amino acids indicated by red shading and similar amino acids indicated by orange shading. The structural elements present in *Gallus gallus *PARP1 are shown at the bottom of the alignment, with the six "core" ß strands indicated [6]. Subclades are separated from one another by spaces with Clade 1A at the top. The amino acids of the HYE catalytic triad are boxed in blue and labelled C1 (H), C2 (Y) and C3 (E). Click here for file

Additional file 6 **Multiple alignment of the PARP catalytic domains of Clade 1 PARP proteins**. Click here for file

Additional file 7 **Multiple alignment of the PARP catalytic domains of Clade 2 PARP proteins annotated with structural predictions**. These alignments only show the conserved PARP catalytic domain. The structural elements predicted to be present in *Arabidopsis thaliana *RCD1 by Phyre are shown at the bottom of the alignment [89]. Annotations as in Additional file 5. Click here for file

Additional file 8 **Multiple alignment of the PARP catalytic domains of Clade 3 proteins annotated with structural information**. These alignments only show the conserved PARP catalytic domain. The structural elements present in *Homo sapiens *PARP15 are shown at the bottom of the alignment. The crystal structure of PARP15 is available in the Protein Data Bank (PDB; http://www.rcsb.org/pdb/explore/explore.do?structureId=3GEY; [171]). Annotations as in Additional file 5. Click here for file

Additional file 9 **Multiple alignment of the PARP catalytic domain from Clade 4 PARP proteins annotated with structural information**. These alignments only show the conserved PARP catalytic domain. The structural elements present in *Homo sapiens *TNK1are shown at the bottom of the alignment [172]. Annotations as in Additional file 5. Click here for file

Additional file 10 **Multiple alignment of the PARP catalytic domain from Clade 5 PARP proteins**. These alignments only show the conserved PARP catalytic domain. The structural elements present in *Homo sapiens *vPARP are shown at the bottom of the alignment. Annotations as in Additional file 5. Click here for file

Additional file 11 **Multiple alignment of the PARP catalytic domain from Clade 6 PARP proteins annotated with structural information**. These alignments only show the conserved PARP catalytic domain. The structural elements predicted to be present in *Homo sapiens *PARP8 by Phyre are shown at the bottom of the alignment [89]. Annotations as in Additional file 6. Click here for file

Additional file 12 **Multiple alignment of the UBCc domain of Clade 6A PARPs**. The entire UBCc domains as defined by Pfam from Clade 6A proteins are shown. Click here for file

Additional file 13 **Clade 6A PARP proteins contain FPE and UBCc domains**. A. Clade 6A PARPs contain UBCc domains in their C termini. An alignment of the HMM consensus sequence of the UBCc domain from Pfam (UBCc) and the UBCc domain from *Phaeosphaeria nodorum *QOUPJ2 (Pn6F). The sequence similarity between the UBCc and UBCc-like domains is shown in red (CONS). +, similar amino acids; -, gaps introduced to maximize the alignment; ., any amino acid. Residues in bold have been shown to be diagnostic of UBCc domains as discussed in the text and [85]. B. Alignment of a region of the Clade 6A PARP UBCc-like domains, containing the catalytic cysteine. The names of the proteins and the amino acid positions (within the UBCc domain) are indicated at left. The blue asterisk marks a histidine and the red asterisk marks the catalytic cysteine, both shared with typical UBCc domains. C. The FPE domain consists of alpha helices and beta strands. The sequence of the FPE domain from the *Phaeosphaeria nodorum *Clade 6A member (QOUPJ2) is shown. Secondary structural characteristics as detected by Phyre are shown above the sequence. h, alpha helices; e, beta strands. Click here for file

Additional file 14 **Multiple alignment of the FPE from Clade 6A PARP proteins**. The entire PfamB_30617/FPE domains as defined by Pfam from Clade 6A proteins are shown. Click here for file
