# Supplementary figures and images for: Evolutionary history of the poly(ADP-ribose) polymerase gene family in eukaryotes
Source: BMC Evol Biol. 2010 Oct 13;10:308. doi: 10.1186/1471-2148-10-308 (PMC2964712; doi:10.1186/1471-2148-10-308)

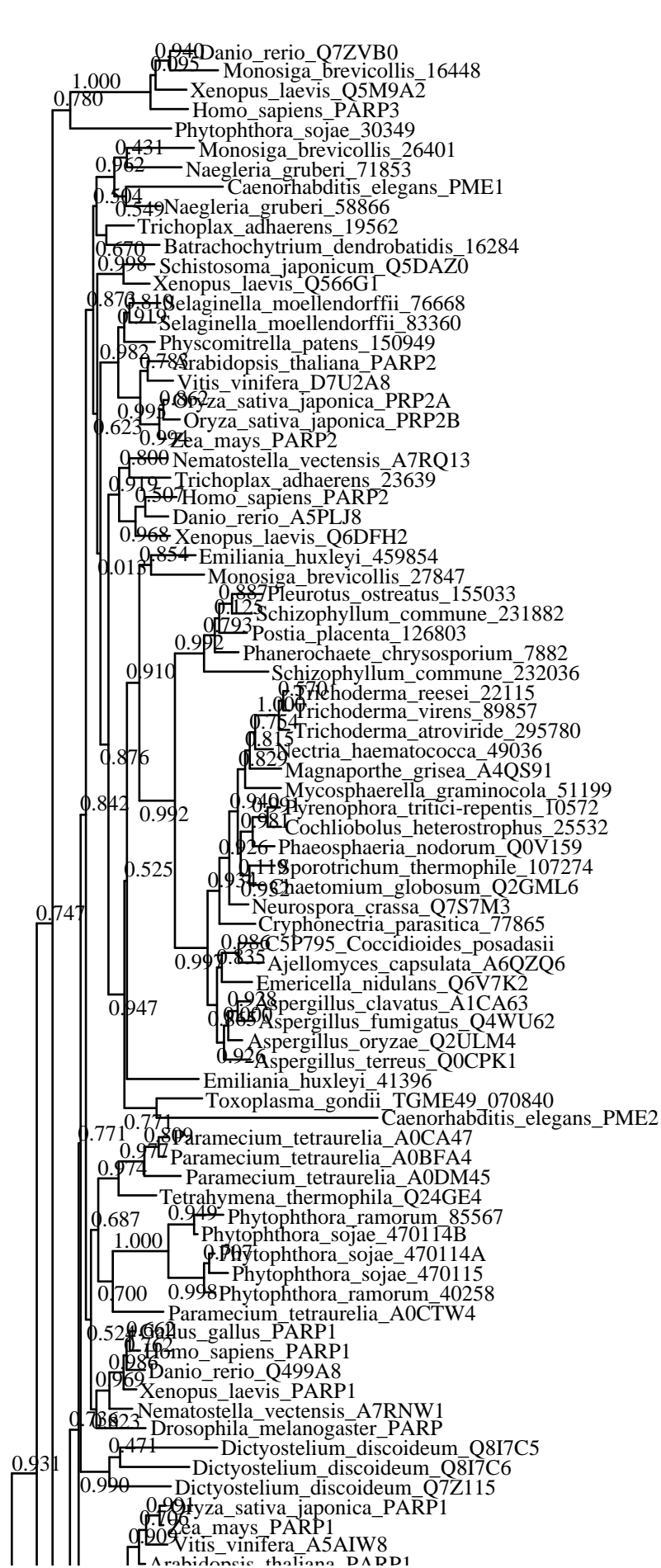

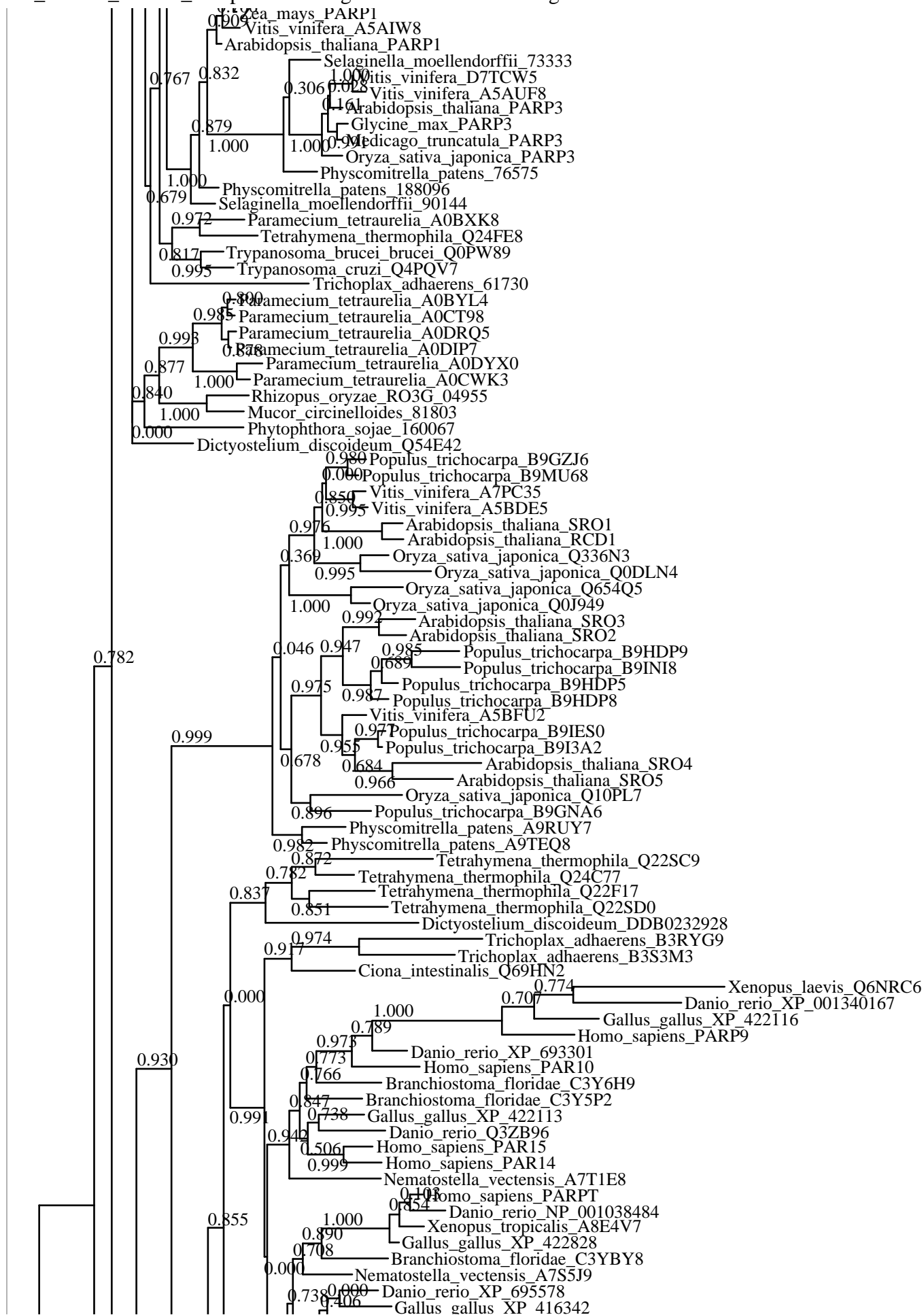

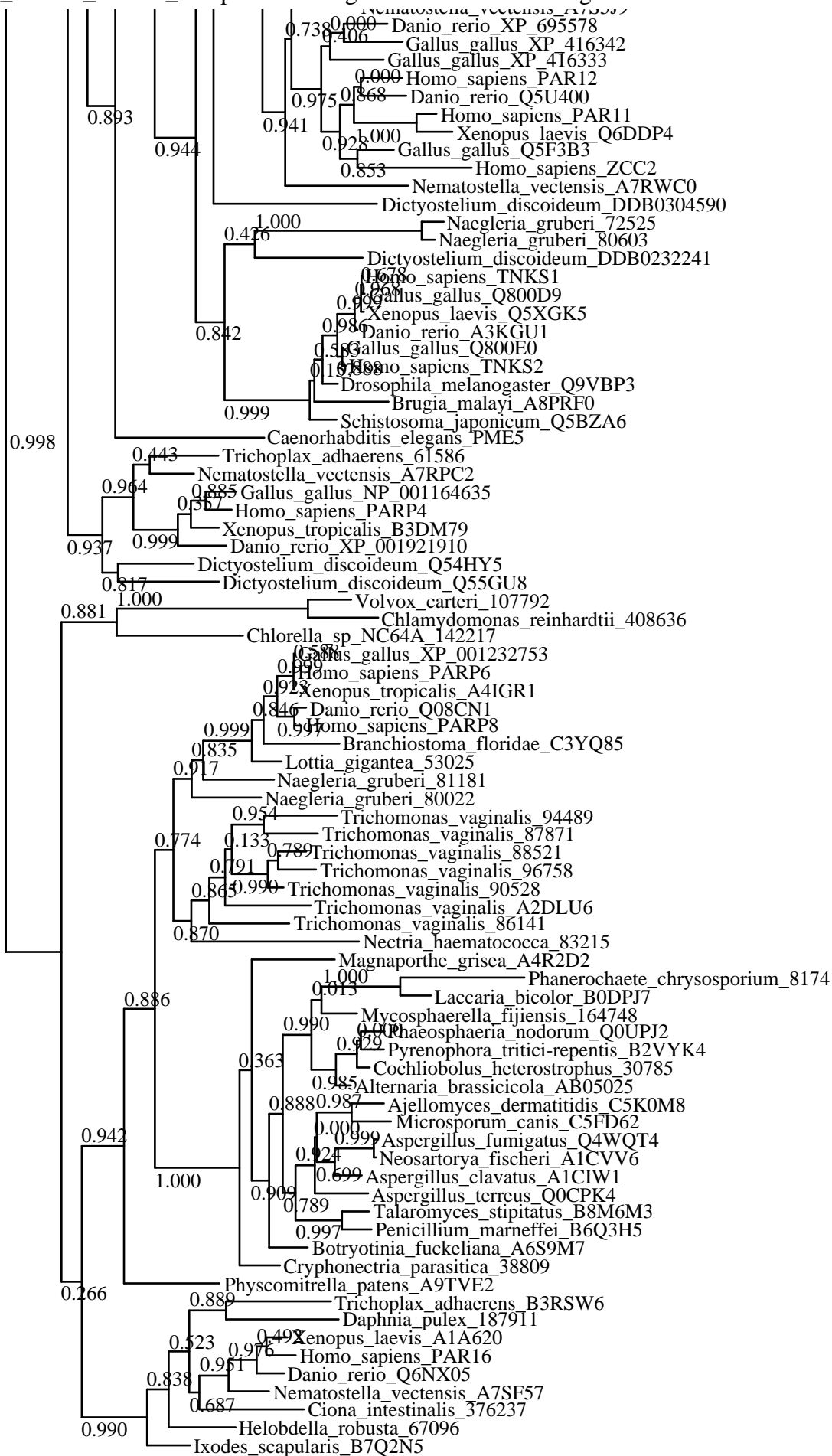

Supplement: Additional file 4 — Phylogenetic analysis of PARP genes in eukaryotes. The represented tree is a ML tree, based on an alignment of the PARP catalytic domain. Branch supports as in Figure 1. Scale bar indicates genetic distance reflected in branch length. [file 1471-2148-10-308-S4.PDF]
