# Supplementary material for: Evolutionary history of the poly(ADP-ribose) polymerase gene family in eukaryotes
Source: BMC Evol Biol. 2010 Oct 13;10:308. doi: 10.1186/1471-2148-10-308 (PMC2964712; doi:10.1186/1471-2148-10-308)
Supplement: Additional file 5 — Multiple alignment of the PARP catalytic domains of Clade 1 PARP proteins. These alignments only show the conserved PARP catalytic domain and the numbers indicate amino acids within the catalytic domain. Due to the large number of proteins in Clade 1, some needed to be removed in order to annotate the sequence. Dots indicate gaps introduced to optimize the alignment and identical amino acids indicated by red shading and similar amino acids indicated by orange shading. The structural elements present in Gallus gallus PARP1 are shown at the bottom of the alignment, with the six "core" ß strands indicated [6]. Subclades are separated from one another by spaces with Clade 1A at the top. The amino acids of the HYE catalytic triad are boxed in blue and labelled C1 (H), C2 (Y) and C3 (E). [file 1471-2148-10-308-S5.PDF]

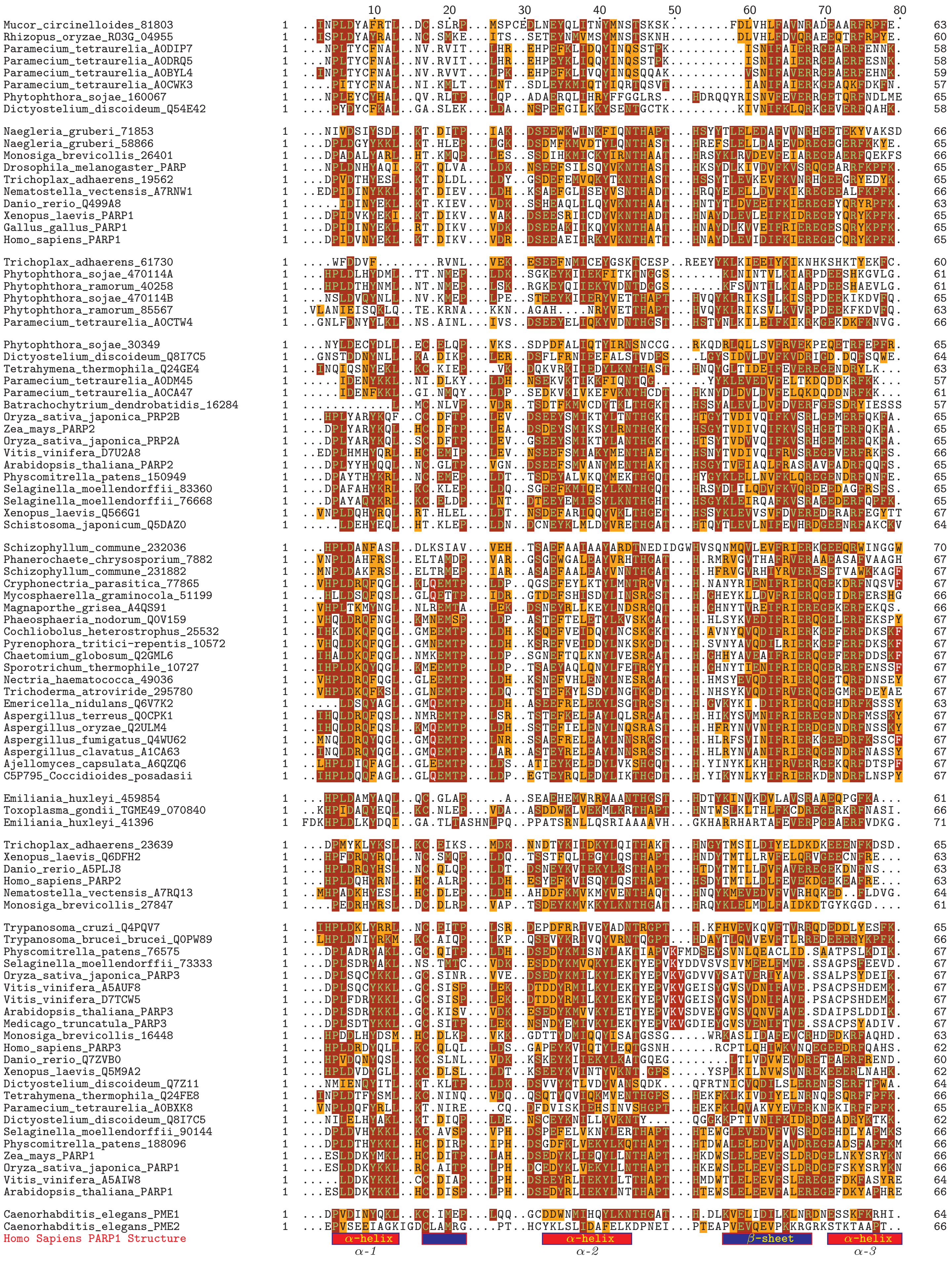
10 20 30 40 50 60 70 80
Mucor_circinelloides_81803
Rhizopus_oryzae_RO3G_04955
Paramecium_tetraurelia_A0DIP7
Paramecium_tetraurelia_A0DRQ5
Paramecium_tetraurelia_A0BYL4
Paramecium_tetraurelia_A0CWK3
Phytophthora_sojae_160067
Dictyostelium_discoideum_Q54E42
Naegleria_gruberi_71853
Naegleria_gruberi_58866
Monosiga_brevicollis_26401
Drosophila_melanogaster_PARP
Trichoplax_adhaerens_19562
Nematostella_vectensis_A7RNW1
Danio_rerio_Q499A8
Xenopus_laevis_PARP1
Gallus_gallus_PARP1
Homo_sapiens_PARP1
Trichoplax_adhaerens_61730
Phytophthora_sojae_470114A
Phytophthora_ramorum_40258
Phytophthora_sojae_470114B
Phytophthora_ramorum_85567
Paramecium_tetraurelia_A0CTW4
Phytophthora_sojae_30349
Dictyostelium_discoideum_Q8I7C5
Tetrahymena_thermophila_Q24GE4
Paramecium_tetraurelia_A0DM45
Paramecium_tetraurelia_A0CA47
Batrachochytrium_dendrobatidis_16284
Oryza_sativa_japonica_PRP2B
Zea_mays_PARP2
Oryza_sativa_japonica_PRP2A
Vitis_vinifera_D7U2A8
Arabidopsis_thaliana_PARP2
Physcomitrella_patens_150949
Selaginella_moellendorffii_83360
Selaginella_moellendorffii_76668
Xenopus_laevis_Q566G1
Schistosoma_japonicum_Q5DAZ0
Schizophyllum_commune_232036
Phanerochaete_chrysosporium_7882
Schizophyllum_commune_231882
Cryphonectria_parasitica_77865
Mycosphaerella_graminocola_51199
Magnaporthe_grisea_A4QS91
Phaeosphaeria_nodorum_Q0V159
Cochliobolus_heterostrophus_25532
Pyrenophora_tritici-repentis_10572
Chaetomium_globosum_Q2GML6
Sporotrichum_thermophile_10727
Nectria_haematococca_49036
Trichoderma_atroviride_295780
Emericella_nidulans_Q6K2
Aspergillus_terreus_Q0CPK1
Aspergillus_oryzae_Q2ULM4
Aspergillus_fumigatus_Q4WU62
Aspergillus_clavatus_A1CA63
Ajellomyces_capsulata_A6QZQ6
C5P795_Coccidioides_posadasii
Emiliania_huxleyi_459854
Toxoplasma_gondii_TGME49_070840
Emiliania_huxleyi_41396
Trichoplax_adhaerens_23639
Xenopus_laevis_Q6DFH2
Danio_rerio_A5PLJ8
Homo_sapiens_PARP2
Nematostella_vectensis_A7RQ13
Monosiga_brevicollis_27847
Trypanosoma_cruzi_Q4PQV7
Trypanosoma_brucei_brucei_Q0PW89
Physcomitrella_patens_76575
Selaginella_moellendorffii_73333
Oryza_sativa_japonica_PARP3
Vitis_vinifera_A5AUF8
Vitis_vinifera_D7TCW5
Arabidopsis_thaliana_PARP3
Medicago_truncatula_PARP3
Monosiga_brevicollis_16448
Homo_sapiens_PARP3
Danio_rerio_Q7ZVB0
Xenopus_laevis_Q5M9A2
Dictyostelium_discoideum_Q7Z11
Tetrahymena_thermophila_Q24FE8
Paramecium_tetraurelia_A0BXK8
Dictyostelium_discoideum_Q8I7C5
Selaginella_moellendorffii_90144
Physcomitrella_patens_188096
Zea_mays_PARP1
Oryza_sativa_japonica_PARP1
Vitis_vinifera_A5AIW8
Arabidopsis_thaliana_PARP1
Caenorhabditis_elegans_PME1
Caenorhabditis_elegans_PME2
Homo Sapiens PARP1 Structure
α-helix
α-1
α-helix
α-2
β-sheet
α-helix
α-3

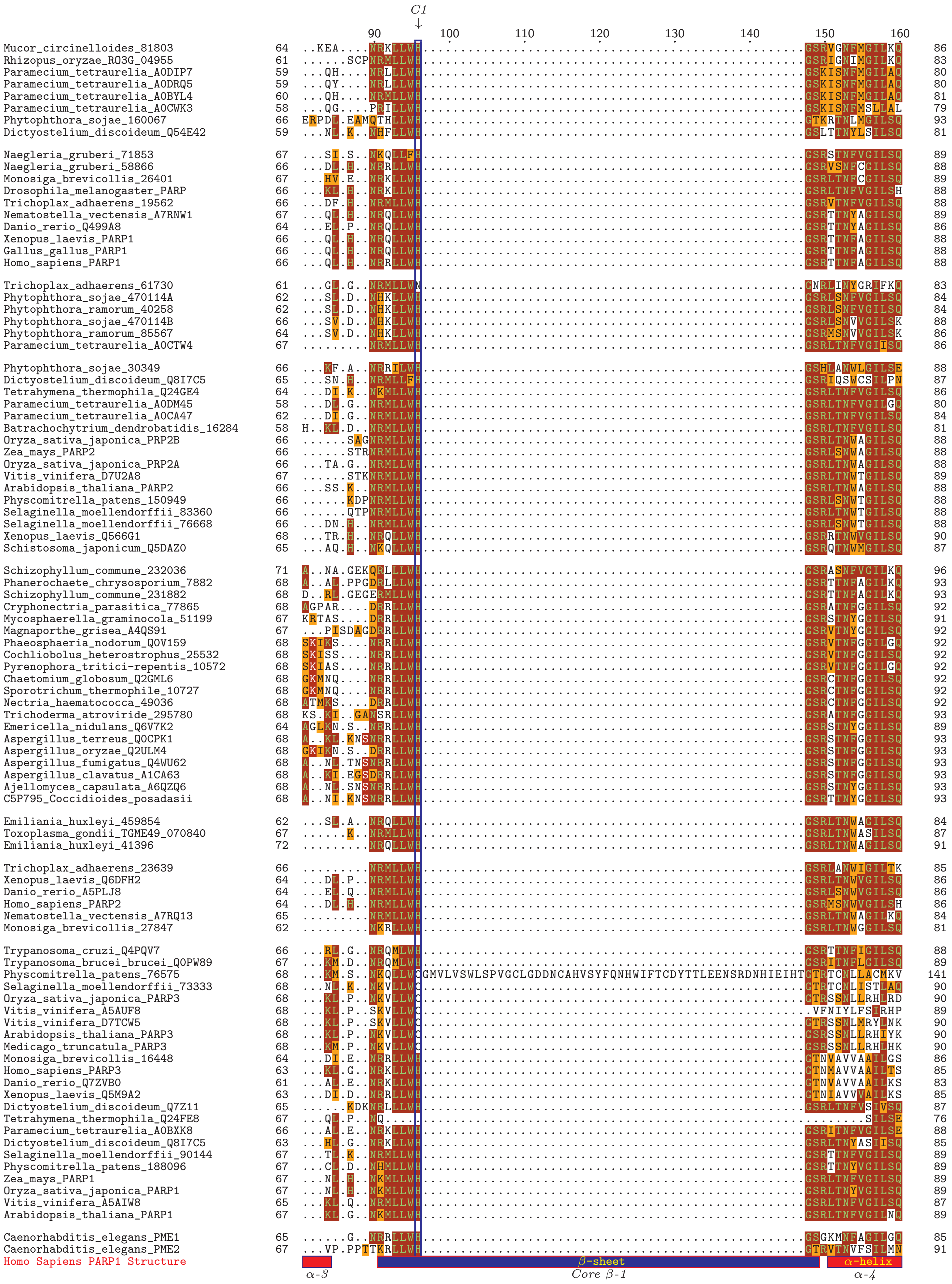
C1
↓
90 100 110 120 130 140 150 160
Mucor_circinelloides_81803 64 ..KEA....NRKLLWH..GSRVGNFMGILKQ 86
Rhizopus_oryzae_RO3G_04955 61 ......SCPNRMLLWH..GSRIGNIMGILKQ 83
Paramecium_tetraurelia_A0DIP7 59 ...QH....NRLLLWH..GSKISNFMGILAQ 80
Paramecium_tetraurelia_A0DRQ5 59 ...QY....NRLLLWH..GSKISNFMGILAQ 80
Paramecium_tetraurelia_A0BYL4 60 ...QH....NRMLLWH..GSKISNFMGILAQ 81
Paramecium_tetraurelia_A0CWK3 58 ...QG....PRILLWH..GSKISNFMSLLAL 79
Phytophthora_sojae_160067 66 ERPDL.EAMQTHLLWH..GTKRTNLMGILSQ 93
Dictyostelium_discoideum_Q54E42 59 ...NL.K..NHFLLWH..GSLTTNYLSILSQ 81
Naegleria_gruberi_71853 67 ...SI.S..NKQLLFH..GSRSTNFVGILSQ 89
Naegleria_gruberi_58866 66 ...DL.H..NRRLLWH..GSRVSNFCGILSQ 88
Monosiga_brevicollis_26401 67 ...HV.E..NRKLLWH..GSRLTNFCGILSQ 89
Drosophila_melanogaster_PARP 66 ...KL.H..NRKLLWH..GSRLTNFVGILSH 88
Trichoplax_adhaerens_19562 66 ...DF.H..NRMLLWH..GSRVTNFVGILSQ 88
Nematostella_vectensis_A7RNW1 67 ...QL.H..NRQLLWH..GSRTTNYAGILSQ 89
Danio_rerio_Q499A8 64 ...EL.P..NRQLLWH..GSRTTNYAGILSQ 86
Xenopus_laevis_PARP1 66 ...QL.H..NRQLLWH..GSRTTNFAGILSQ 88
Gallus_gallus_PARP1 66 ...QL.H..NRQLLWH..GSRTTNFAGILSQ 88
Homo_sapiens_PARP1 66 ...QL.H..NRRLLWH..GSRTTNFAGILSQ 88
Trichoplax_adhaerens_61730 61 ...GL.G..NRMLLWN..GNRLINYGRIFKQ 83
Phytophthora_sojae_470114A 62 ...SL.D..NHKLLWH..GSRLSNFVGILSQ 84
Phytophthora_ramorum_40258 62 ...SL.D..NHKLLWH..GSRLSNFVGILSQ 84
Phytophthora_sojae_470114B 66 ...SV.D..NHKLLWH..GSRLSNVVGILSK 88
Phytophthora_ramorum_85567 64 ...SV.D..NHKLLWH..GSRMSNVVGILSK 86
Paramecium_tetraurelia_A0CTW4 67 ....NRMLLWH..GSRLTNFVGIISQ 86
Phytophthora_sojae_30349 66 ...KF.A..NRRILWH..GSHLANWLGILSE 88
Dictyostelium_discoideum_Q8I7C5 65 ...SN.H..NRMLLFH..GSRIQSWCSILPN 87
Tetrahymena_thermophila_Q24GE4 64 ...DI.K..NKMLLWH..GSRLTNFVGILSQ 86
Paramecium_tetraurelia_A0DM45 58 ...DL.G..NRMLLWH..GSRLTNFVGILGQ 80
Paramecium_tetraurelia_A0CA47 62 ...DI.G..NRMLLWH..GSRLTNFVGILSQ 84
Batrachochytrium_dendrobatidis_16284 58 H..KL.D..NRMLLWH..GSRLTNFVGILSQ 81
Oryza_sativa_japonica_PRP2B 66 ......SAGNRMLLWH..GSRLTNWAGILSQ 88
Zea_mays_PARP2 66 ......STRNRMLLWH..GSRLSNWAGILSQ 88
Oryza_sativa_japonica_PRP2A 66 ...TA.G..NRMLLWH..GSRLTNWAGILSQ 88
Vitis_vinifera_D7U2A8 67 ......STKNRMLLWH..GSRLTNWTGILSQ 89
Arabidopsis_thaliana_PARP2 66 ...SS.K..NRMLLWH..GSRLTNWAGILSQ 88
Physcomitrella_patens_150949 66 ......KDPNRMLLWH..GSRLSNWTGILSQ 88
Selaginella_moellendorffii_83360 66 ......QTPNRMLLWH..GSRLTNWTGILSQ 88
Selaginella_moellendorffii_76668 66 ...DN.H..NRMLLWH..GSRLSNWTGILSQ 88
Xenopus_laevis_Q566G1 68 ...TR.H..NRQLLWH..GSRRTNWVGILSQ 90
Schistosoma_japonicum_Q5DAZ0 65 ...AQ.H..NKQLLWH..GSRQTNWMGILSQ 87
Schizophyllum_commune_232036 71 A..NA.GEKQRLLLWH..GSRASNFVGILKQ 96
Phanerochaete_chrysosporium_7882 68 A..AL.PPGDRLLLWH..GSRTTNFAGILKQ 93
Schizophyllum_commune_231882 68 D..RL.GEGERMLLWH..GSRTTNFAGILKQ 93
Cryphonectria_parasitica_77865 68 AGPAR....DRRLLWH..GSRATNFGGILSQ 92
Mycosphaerella_graminocola_51199 67 KRTAS....DRRLLWH..GSRSTNYGGILSQ 91
Magnaporthe_grisea_A4QS91 67 ...PISDAGDRRLLWH..GSRVTNYGGILSQ 92
Phaeosphaeria_nodorum_Q0V159 68 SKIKS....NRRLLWH..GSRVTNFGGILGQ 92
Cochliobolus_heterostrophus_25532 68 SKISS....NRRLLWH..GSRVTNFGGILSQ 92
Pyrenophora_tritici-repentis_10572 68 SKIAS....NRRLLWH..GSRVTNFGGILGQ 92
Chaetomium_globosum_Q2GML6 68 GKMNQ....NRRLLWH..GSRCTNFGGILSQ 92
Sporotrichum_thermophile_10727 68 GKMNQ....NRRLLWH..GSRCTNFGGILSQ 92
Nectria_haematococca_49036 68 ATMKS....DRRLLWH..GSRCTNFGGILSQ 92
Trichoderma_atroviride_295780 68 KS.KI..GANSRLLWH..GSRATNFGGILSQ 93
Emericella_nidulans_Q6V7K2 64 AGLKN.S..NRRLLWH..GSRSTNYGGILSQ 89
Aspergillus_terreus_Q0CPK1 68 A..KL.KNSNRRLLWH..GSRSTNFGGILSQ 93
Aspergillus_oryzae_Q2ULM4 68 GKIKN.S..DRRLLWH..GSRSTNFGGILSQ 93
Aspergillus_fumigatus_Q4WU62 68 A..NL.TNSNRRLLWH..GSRSTNFGGILSQ 93
Aspergillus_clavatus_A1CA63 68 A..KI.EGSDRRLLWH..GSRSTNFGGILSQ 93
Ajellomyces_capsulata_A6QZQ6 68 A..NL.SNSNRRLLWH..GSRSTNYGGILSQ 93
C5P795_Coccidioides_posadasii 68 A..NI.KNSNRRLLWH..GSRTTNYGGILSQ 93
Emiliania_huxleyi_459854 62 ...SL.A..NRQLLWH..GSRLTNWAGILSQ 84
Toxoplasma_gondii_TGME49_070840 67 ......K..NRMLLWH..GSRLTNWASILSQ 87
Emiliania_huxleyi_41396 72 ....NRQLLWH..GSRLTNWAGILSQ 91
Trichoplax_adhaerens_23639 66 ....NRMLLWH..GSRLANWIGILTK 85
Xenopus_laevis_Q6DFH2 64 ...DL.P..NRMLLWH..GSRLTNWVGILSQ 86
Danio_rerio_A5PLJ8 64 ...EL.Q..NRMLLWH..GSRLSNWVGILSQ 86
Homo_sapiens_PARP2 64 ...DL.H..NRMLLWH..GSRMSNWVGILSH 86
Nematostella_vectensis_A7RQ13 65 ....NRMLLWH..GSRLTNWAGILKQ 84
Monosiga_brevicollis_27847 62 ....NKRLLWH..GSRLTNWGGILSQ 81
Trypanosoma_cruzi_Q4PQV7 66 ...RL.G..NRQMLWH..GSRTTNFIGILSQ 88
Trypanosoma_brucei_brucei_Q0PW89 67 ...KM.D..NRQMLWH..GSRITNFLGILSQ 89
Physcomitrella_patens_76575 68 ...KM.S..NKQLLWQGMVLVSWLSPVGCLGDDNCAHVSYFQNHWIFTCDYTTLEENSRDNHIEIHTGTRTCNLLACMKV 141
Selaginella_moellendorffii_73333 68 ...NL.K..NKVLLWQ..GTRTCNLISTLAQ 90
Oryza_sativa_japonica_PARP3 68 ...KL.P..NKVLLWQ..GTRSSNLLRHLRD 90
Vitis_vinifera_A5AUF8 68 ...KL.P..SKVLLWQ..VFNIYLFSIRHP 89
Vitis_vinifera_D7TCW5 68 ...KL.P..SKVLLWQ..GTRSSNLMRYLNK 90
Arabidopsis_thaliana_PARP3 68 ...KL.P..NKVLLWQ..GSRSSNLLRHIYK 90
Medicago_truncatula_PARP3 68 ...KM.P..NKVLLWQ..GSRSSNLLRHLHK 90
Monosiga_brevicollis_16448 64 ...DI.E..NRRLLWH..GTNVAVVAAILGS 86
Homo_sapiens_PARP3 63 ...KL.G..NRKLLWH..GTNMAVVAAILTS 85
Danio_rerio_Q7ZVB0 61 ...AL.E..NRKLLWH..GTNVAVVAAILKS 83
Xenopus_laevis_Q5M9A2 63 ...DI.D..NRRLLWH..GTNIAVVVAILKS 85
Dictyostelium_discoideum_Q7Z11 65 ......KDKNRLLLWH..GSRLTNFVSIVSQ 87
Tetrahymena_thermophila_Q24FE8 67 ...QL.P..NQ...SILSE 76
Paramecium_tetraurelia_A0BXK8 66 ...AL.E..NRKLLWH..GSRITNFVGILSE 88
Dictyostelium_discoideum_Q8I7C5 63 ...HL.G..NRKLLWH..GSRLTNYASIISQ 85
Selaginella_moellendorffii_90144 67 ...TL.K..NRMLLWH..GSRTTNFVGILSQ 89
Physcomitrella_patens_188096 67 ...CL.D..NHMLLWH..GSRTTNYVGILSQ 89
Zea_mays_PARP1 67 ...NL.H..NKMLLWH..GSRLTNFVGILSQ 89
Oryza_sativa_japonica_PARP1 67 ...NL.H..NKMLLWH..GSRLTNYVGILSQ 89
Vitis_vinifera_A5AIW8 65 ...KL.Q..NRMLLWH..GSRLTNFVGILSQ 87
Arabidopsis_thaliana_PARP1 67 ...KL.G..NKMLLWH..GSRLTNFVGILNQ 89
Caenorhabditis_elegans_PME1 65 ......G..NRRLLWH..GSGKMNFAGILGQ 85
Caenorhabditis_elegans_PME2 67 ...VP.PPTTKRLLWH..GTRVTNVFSILMN 91
Homo Sapiens PARP1 Structure
β-sheet
α-helix
α-3
Core β-1
α-4

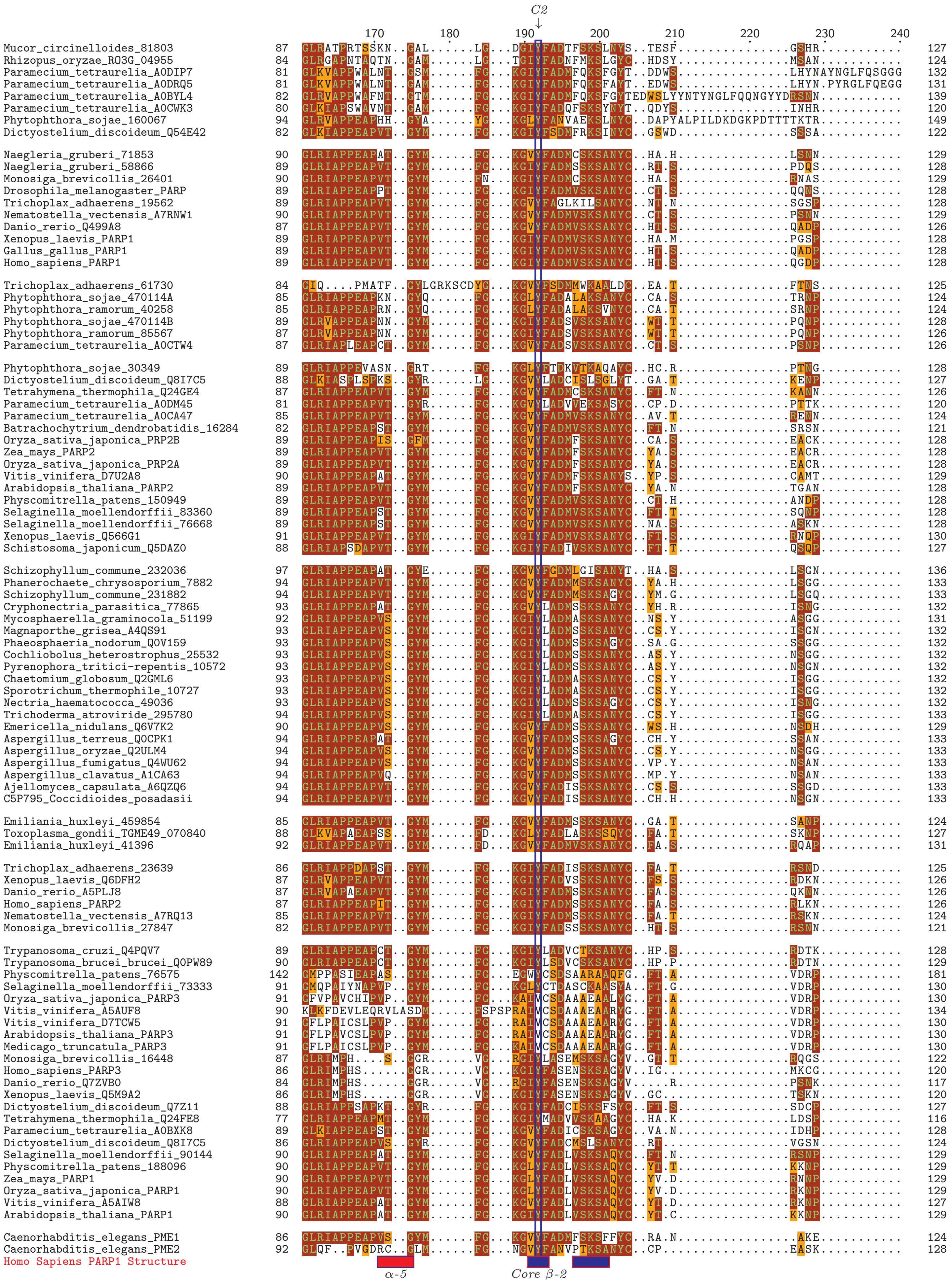

C2
170 180 190 200 210 220 230 240
Mucor_circinelloides_81803 87 GLRATPRTSSKN..GAL......LG...DGIYFADTFSKSLNYS..TESF...............GSHR.......... 127
Rhizopus_oryzae_RO3G_04955 84 GLRGAPNTAQTN..GAM......LG...TGIYFADNFMKSLGYC..HDSY...............MSAN.......... 124
Paramecium_tetraurelia_A0DIP7 81 GLKVAPPWALNT..GSM......FG...KGIYFADMFQKSFGYT..DDWS...............LHYNAYNGLFQSGGG 132
Paramecium_tetraurelia_A0DRQ5 81 GLKVAPPWALNT..GAM......FG...KGIYFADMFQKSFAYT..EDWS...............LHYN.PYRGLFQEGG 131
Paramecium_tetraurelia_A0BYL4 82 GLRVAPPWAFNT..GTM......FG...KGIYFADMFQKSFGYTEDWSLYYNTYNGLFQQNGYYDRSNN.......... 139
Paramecium_tetraurelia_A0CWK3 80 GLKIAPSWAVNT..GAM......FG...KGIYFADQFSKSYNYT..QDYS...............INHR.......... 120
Phytophthora_sojae_160067 94 GLRVAPPEAPHH..GYA......YG...KGLYFANVAEKSLNYC..DAPYALPILDKDGKPDTTTTKTR.......... 149
Dictyostelium_discoideum_Q54E42 82 GLKIAPPEAPVT..GYM......FG...KGIYFSDMFRKSINYC..GSWD...............SSSA.......... 122
Naegleria_gruberi_71853 90 GLRIAPPEAPAT..GYM......FG...KGVYFADMCSKSANYC..HA.H...............LSNN.......... 129
Naegleria_gruberi_58866 89 GLRIAPPEAPVT..GYM......FG...KGIYFADMSSKSANYC..RT.S...............PDQS.......... 128
Monosiga_brevicollis_26401 90 GLRIAPPEAPVT..GYM......FN...KGIYFADMCSKSANYC..HA.S...............RNAS.......... 129
Drosophila_melanogaster_PARP 89 GLRIAPPEAPPT..GYM......FG...KGIYFADMVSKSANYC..CT.S...............QQNS.......... 128
Trichoplax_adhaerens_19562 89 GLRIAPPEAPVT..GYM......FG...KGVYFAGLKILSANYC..NT.N...............SGSP.......... 128
Nematostella_vectensis_A7RNW1 90 GLRIAPPEAPVT..GYM......FG...KGVYFADMVSKSANYC..CT.S...............PSNN.......... 129
Danio_rerio_Q499A8 87 GLRIAPPEAPVT..GYM......FG...KGVYFADMVSKSANYC..HT.S...............QADP.......... 126
Xenopus_laevis_PARP1 89 GLRIAPPEAPVT..GYM......FG...KGIYFADMVSKSANYC..HA.M...............PGSP.......... 128
Gallus_gallus_PARP1 89 GLRIAPPEAPVT..GYM......FG...KGIYFADMVSKSANYC..HT.S...............QADP.......... 128
Homo_sapiens_PARP1 89 GLRIAPPEAPVT..GYM......FG...KGIYFADMVSKSANYC..HT.S...............QGDP.......... 128
Trichoplax_adhaerens_61730 84 GIQ....PMATF..GYLGRKSCDYG...KGVYFSDMMWKAALDC..EA.T...............FTNS.......... 125
Phytophthora_sojae_470114A 85 GLRIAPPEAPKN..GYQ......FG...KGLYFADALAKSANYC..CA.S...............TRNP.......... 124
Phytophthora_ramorum_40258 85 GLRIAPPEAPRN..GYQ......FG...KGLYFADALAKSVNYC..CA.T...............SRNP.......... 124
Phytophthora_sojae_470114B 89 GLRVAPPEAPNN..GYM......FG...KGIYFADSVSKSANYC..WT.T...............PQNP.......... 128
Phytophthora_ramorum_85567 87 GLRVAPPEAPNN..GYM......FG...KGIYFADSVSKSANYC..WT.T...............PQNP.......... 126
Paramecium_tetraurelia_A0CTW4 87 GLRIAPLEAPCT..GYM......FG...KGVYFADSVSKSANYC..CT.S...............PSNP.......... 126
Phytophthora_sojae_30349 89 GLRIAPPEVASN..GRT......FG...KGLYFTDKVTKAQAYC..HC.R...............PTNG.......... 128
Dictyostelium_discoideum_Q8I7C5 88 GLKIASPLSPKS..GYR......LG...KGVYLADCISLSGLYT..GA.T...............KENP.......... 127
Tetrahymena_thermophila_Q24GE4 87 GLRIAPPEAPVT..GYM......FG...KGVYFADMCSKSANYC..FT.N...............KANN.......... 126
Paramecium_tetraurelia_A0DM45 81 GLRIAPPEAPVT..GYR......FG...KGVYLADVVEKSASYC..CP.D...............PTTK.......... 120
Paramecium_tetraurelia_A0CA47 85 GLRIAPPEAPVT..GYM......FG...KGVYFADMVSKSANYC..AV.T...............RENN.......... 124
Batrachochytrium_dendrobatidis_16284 82 GLRIAPPEAPST..GYM......FG...KGVYFADMVSKSANYC..FT.N...............SRSN.......... 121
Oryza_sativa_japonica_PRP2B 89 GLRIAPPEAPIS..GFM......FG...KGVYFADMFSKSANYC..CA.S...............EACK.......... 128
Zea_mays_PARP2 89 GLRIAPPEAPVT..GYM......FG...KGVYFADMFSKSANYC..YA.S...............EACR.......... 128
Oryza_sativa_japonica_PRP2A 89 GLRIAPPEAPVT..GYM......FG...KGVYFADMFSKSANYC..YA.S...............EACR.......... 128
Vitis_vinifera_D7U2A8 90 GLRIAPPEAPAT..GYM......FG...KGVYFADMFSKSANYS..YP.S...............CAMT.......... 129
Arabidopsis_thaliana_PARP2 89 GLRIAPPEAPVT..GYM......FG...KGVYFADMFSKSANYC..YA.N...............TGAN.......... 128
Physcomitrella_patens_150949 89 GLRIAPPEAPVT..GYM......FG...KGVYFADMVSKSANYC..CT.H...............ANDP.......... 128
Selaginella_moellendorffii_83360 89 GLRIAPPEAPST..GYM......FG...KGVYFADMVSKSANYC..FT.T...............SQNP.......... 128
Selaginella_moellendorffii_76668 89 GLRIAPPEAPST..GYM......FG...KGVYFADMVSKSANYC..NA.S...............ASKN.......... 128
Xenopus_laevis_Q566G1 91 GLRIAPPEAPVT..GYM......FG...KGIYFADMVSKSANYC..FT.S...............RNQP.......... 130
Schistosoma_japonicum_Q5DAZ0 88 GLRIAPSDAPVT..GYM......FG...KGIYFADIVSKSANYC..FT.T...............QSQP.......... 127
Schizophyllum_commune_232036 97 GLRIAPPEAPAT..GYE......FG...KGVYFGDMLGISANYT..HA.S...............LSGN.......... 136
Phanerochaete_chrysosporium_7882 94 GLRIAPPEAPVT..GYM......FG...KGVYFADMMSKSANYC..YA.H...............LSGG.......... 133
Schizophyllum_commune_231882 94 GLRIAPPEAPVT..GYM......FG...KGVYFADMMSKSAGYC..YM.G...............LSGQ.......... 133
Cryphonectria_parasitica_77865 93 GLRIAPPEAPAT..GYM......FG...KGVYLADMSSKSANYC..YH.R...............ISNG.......... 132
Mycosphaerella_graminocola_51199 92 GLRIAPPEAPVS..GYM......FG...KGIYLADMASKSANYC..NS.Y...............ASGG.......... 131
Magnaporthe_grisea_A4QS91 93 GLRIAPPEAPVS..GYM......FG...KGIYLADMSSKSANYC..CS.Y...............ISGN.......... 132
Phaeosphaeria_nodorum_Q0V159 93 GLRIAPPEAPVS..GYM......FG...KGIYLADMSSKSAGYC..SA.G...............SSGG.......... 132
Cochliobolus_heterostrophus_25532 93 GLRIAPPEAPVS..GYM......FG...KGIYLADMSSKSANYC..AS.Y...............NSGG.......... 132
Pyrenophora_tritici-repentis_10572 93 GLRIAPPEAPVS..GYM......FG...KGIYLADMSSKSANYC..AS.Y...............NSGN.......... 132
Chaetomium_globosum_Q2GML6 93 GLRIAPPEAPVS..GYM......FG...KGIYLADMASKSANYC..CS.Y...............ISGG.......... 132
Sporotrichum_thermophile_10727 93 GLRIAPPEAPVS..GYM......FG...KGIYLADMASKSANYC..CS.Y...............ISGG.......... 132
Nectria_haematococca_49036 93 GLRIAPPEAPVS..GYM......FG...KGIYLADMSSKSAGYC..CS.Y...............ISNG.......... 132
Trichoderma_atroviride_295780 94 GLRIAPPEAPVS..GYM......FG...KAIYLADMASKSANYC..CS.Y...............ISGG.......... 133
Emericella_nidulans_Q6V7K2 90 GLRIAPPEAPVS..GYM......FG...KGVYFADMSSKSANYC..WS.H...............NSDH.......... 129
Aspergillus_terreus_Q0CPK1 94 GLRIAPPEAPAT..GYM......FG...KGVYFADMSSKSAGYC..CH.Y...............SSAN.......... 133
Aspergillus_oryzae_Q2ULM4 94 GLRIAPPEAPVS..GYM......FG...KGVYFADMSSKSANYC..CS.Y...............NSGG.......... 133
Aspergillus_fumigatus_Q4WU62 94 GLRIAPPEAPVS..GYM......FG...KGVYFADMSSKSANYC..VP.Y...............NSAN.......... 133
Aspergillus_clavatus_A1CA63 94 GLRIAPPEAPVQ..GYM......FG...KGVYFADMSSKSANYC..MP.Y...............NSAN.......... 133
Ajellomyces_capsulata_A6QZQ6 94 GLRIAPPEAPVT..GYM......FG...KGVYFADISSKSANYC..CS.S...............SSGD.......... 133
C5P795_Coccidioides_posadasii 94 GLRIAPPEAPVT..GYM......FG...KGVYFADISSKSANYC..CH.H...............NSGN.......... 133
Emiliania_huxleyi_459854 85 GLRIAPPEAPVT..GYM......FG...KGVYFADMSSKSANYC..GA.T...............SANP.......... 124
Toxoplasma_gondii_TGME49_070840 88 GLKVAPAEAPSS..GYM......FD...KGIYFADLASKSQYC..FA.T...............SKNP.......... 127
Emiliania_huxleyi_41396 92 GLRIAPPEAPVT..GYM......FD...KGVYFADMVSKSANYC..FA.S...............RQAP.......... 131
Trichoplax_adhaerens_23639 86 GLRIAPPDAPST..GYM......FG...KGIYFADISSKSANYC..FA.T...............RSND.......... 125
Xenopus_laevis_Q6DFH2 87 GLRVAPPEAPVT..GYM......FG...KGIYFADVSSKSANYC..FS.S...............RDKN.......... 126
Danio_rerio_A5PLJ8 87 GLRVAPAEAPVT..GYM......FG...KGIYFADMSSKSANYC..FA.S...............QKNN.......... 126
Homo_sapiens_PARP2 87 GLRIAPPEAPIT..GYM......FG...KGIYFADMSSKSANYC..FA.S...............RLKN.......... 126
Nematostella_vectensis_A7RQ13 85 GLRIAPPEAPVT..GYM......FG...KGIYFADMSSKSANYC..FA.T...............RSKN.......... 124
Monosiga_brevicollis_27847 82 GLRIAPPEAPVT..GYM......FG...KGIYFADSSSKSANYC..HT.S...............RSNN.......... 121
Trypanosoma_cruzi_Q4PQV7 89 GLRIAPPEAPCT..GYM......FG...KGIYLADVCTKSANYC..HP.S...............RDTK.......... 128
Trypanosoma_brucei_brucei_Q0PW89 90 GLRIAPPEAPCT..GYM......FG...KGIYLSDVCSKSANYC..HP.P...............RDTN.......... 129
Physcomitrella_patens_76575 142 GMPPASIEAPAS..GYM......FG...EGWVCSDSAARAQFG..FT.A...............VDRP.......... 181
Selaginella_moellendorffii_73333 91 GMQPAIYNAPVP..GYM......FG...KGLYCTDASCKAASYA..FT.G...............VDRP.......... 130
Oryza_sativa_japonica_PARP3 91 GFVPAVCHIPVP..GYM......FG...KAIVCSDAAAEAALYG..FT.A...............VDRP.......... 130
Vitis_vinifera_A5AUF8 90 KLKFDEVLEQRVLASDM......FSPSPRAIVCSDAAAEAARYG..FT.A...............VDRP.......... 134
Vitis_vinifera_D7TCW5 91 GFLPAICSLPVP..GYM......FG...RAIVCSDAAAEAARYG..FT.A...............VDRP.......... 130
Arabidopsis_thaliana_PARP3 91 GFLPAVCSLPVP..GYM......FG...RAIVCSDAAAEAARYG..FT.A...............VDRP.......... 130
Medicago_truncatula_PARP3 91 GFLPAICSLPVP..GYM......FG...KAIVCSDAAAEAARYG..FT.A...............VDRP.......... 130
Monosiga_brevicollis_16448 87 GLRIMPH....S..GGR......VG...RGIYLASEMSKSAGYV..GT.T...............RQGS.......... 122
Homo_sapiens_PARP3 86 GLRIMPHS......GGR......VG...KGIYFASENSKSAGYV..IG.................MKCG.......... 120
Danio_rerio_Q7ZVB0 84 GLRIMPHS......GGR......VG...RGIYFASENSKSAGYV.....R...............PSNK.......... 117
Xenopus_laevis_Q5M9A2 86 GLRIMPHS......GGR......VG...KGIYFASENSKSAGYV..GC.................TSKN.......... 120
Dictyostelium_discoideum_Q7Z11 88 GLRIAPPSAPKT..GYR......FG...KGIYFADCISKSFSYC..FT.S...............SDCP.......... 127
Tetrahymena_thermophila_Q24FE8 77 GLRIAPPEAPMT..GYM......FG...KGIYMADVVSKAAGYC..HA.K...............LDSP.......... 116
Paramecium_tetraurelia_A0BXK8 89 GLKIAPPEAPST..GYM......FG...KGVYFADICSKSAGYC..VA.N...............IDHP.......... 128
Dictyostelium_discoideum_Q8I7C5 86 GLRIAPPEAPVS..GYR......FG...KGVYFADCMSLSANYC..RT.................VGSN.......... 124
Selaginella_moellendorffii_90144 90 GLRIAPPEAPAT..GYM......FG...KGVYFADLVSKSAQYC..FT.N...............RSNP.......... 129
Physcomitrella_patens_188096 90 GLRIAPPEAPVT..GYM......FG...KGLYFADLVSKSAQYC..YT.T...............KKNP.......... 129
Zea_mays_PARP1 90 GLRIAPPEAPVT..GYM......FG...KGLYFADLVSKSAQYC..YV.D...............RNNP.......... 129
Oryza_sativa_japonica_PARP1 90 GLRIAPPEAPVT..GYM......FG...KGLYFADLVSKSAQYC..YV.D...............RKNP.......... 129
Vitis_vinifera_A5AIW8 88 GLRIAPPEAPAT..GYM......FG...KGVYFADLVSKSAQYC..YT.D...............RKNP.......... 127
Arabidopsis_thaliana_PARP1 90 GLRIAPPEAPAT..GYM......FG...KGIYFADLVSKSAQYC..YT.C...............KKNP.......... 129
Caenorhabditis_elegans_PME1 86 GLRIAPPEAPVS..GYM......FG...KGVYFADMFSKSFFYC..RA.N................AKE.......... 124
Caenorhabditis_elegans_PME2 92 GLQF..PVGDRC..GLM......FG...NGVYFANVPTKSANYC..CP.................EASK.......... 128
Homo Sapiens PARP1 Structure
α-5
Core β-2

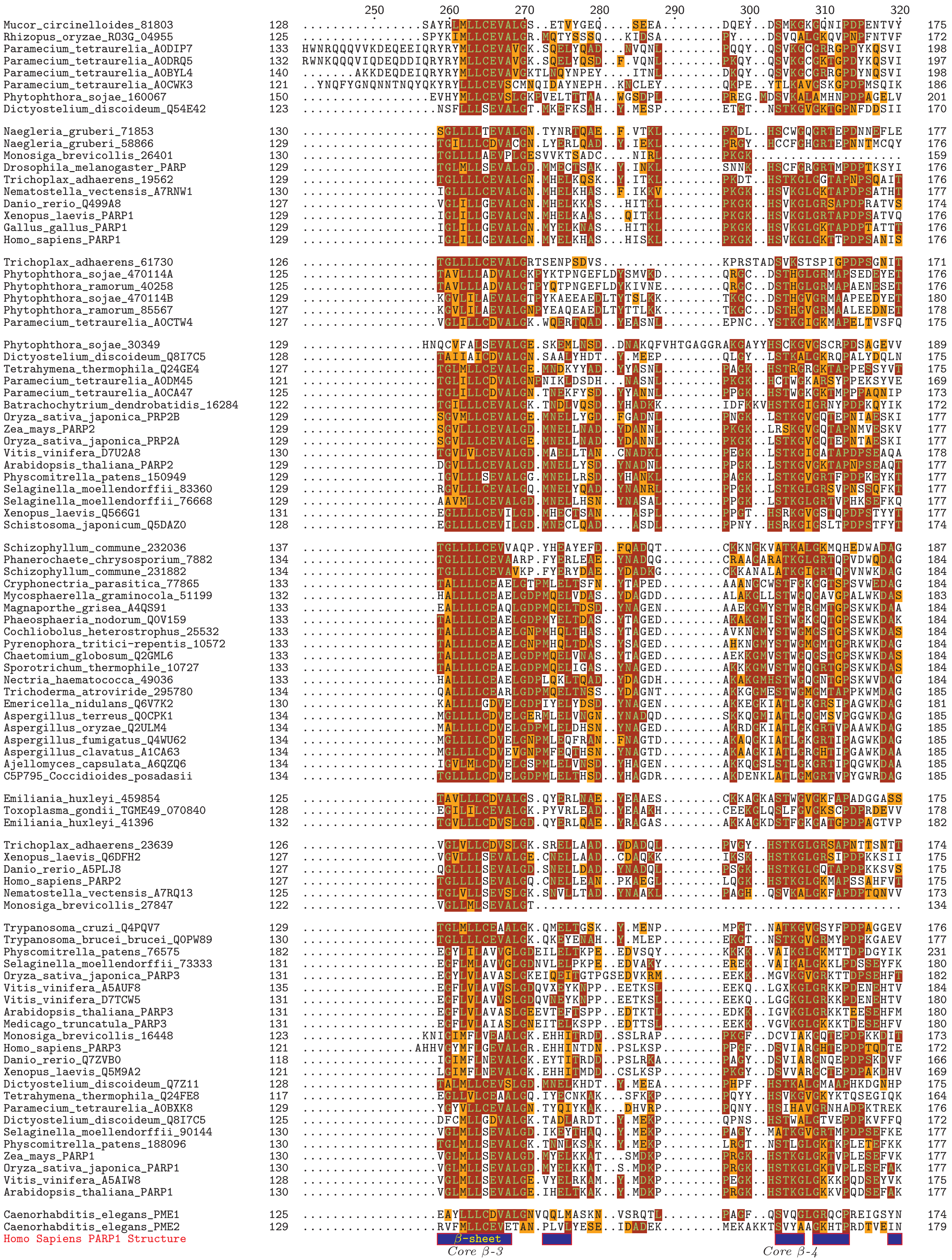

250
260
270
280
290
300
310
320
Homo Sapiens PARP1 Structure
β-sheet
Core β-3
Core β-4

```
                                                   330       340       350       360       370       380       390       400
Mucor_circinelloides_81803             176  ......DESG.LCVP...IGPCIPN......TQ........PTTQNATHNQMFGYSPPVTL...................  212
Rhizopus_oryzae_RO3G_04955             173  ......DKNG.TRIT...FGPCVEN..TFTEQT........NDIV...............L...................  198
Paramecium_tetraurelia_A0DIP7          199  ......LSNG.CKVP...VGQCIDYPDPKKKDK........DGNP...........IQYYL...................  230
Paramecium_tetraurelia_A0DRQ5          198  ......LSNG.CKVP...VGQCIEYPEPKKKDK........EGNP...........IHYCL...................  229
Paramecium_tetraurelia_A0BYL4          199  ......LSNG.CKVP...VGQCIDYPQPKKKDK........EGNP...........VRFQL...................  230
Paramecium_tetraurelia_A0CWK3          187  ......MPNG.CVVP...IGQIVSNSIPHQIQE........RTKN...............I...................  214
Phytophthora_sojae_160067              202  .....SPKCG.AKLH...LGKVKQV......GRELPFDRVWAKTDPNPAPMGWYERNPKFMPETQDYLSKLLEDKSFTVG  266
Dictyostelium_discoideum_Q54E42        171  ......LNNG.VAIP...LSPPEQV....KGDT........SDYH...............L...................  194

Naegleria_gruberi_71853                178  ..IDSSCSGQKIKVP...LGKPTQV.......Q........RSGS...............L...................  203
Naegleria_gruberi_58866                177  ......ILDGKVMVP...LGTPSAS....LLYN........RHTS...............L...................  201
Monosiga_brevicollis_26401             160  ........DGRVTVP...LGKATSA......DV........SRTD...............L...................  180
Drosophila_melanogaster_PARP           177  ......RSDG.VEIP...YGETITD......EH........LKSS...............L...................  198
Trichoplax_adhaerens_19562             177  ......LENG.TVVP...LGKSSKS......KV........TNSS...............L...................  198
Nematostella_vectensis_A7RNW1          178  ......FEDG.TIVP...KGKGCPA......PV........KDSS...............L...................  199
Danio_rerio_Q499A8                     175  .......LNG.VDIP...LGKGMNT......NI........DDTS...............L...................  195
Xenopus_laevis_PARP1                   177  .......LDG.VDVP...LGKGTSA......NI........SDTS...............L...................  197
Gallus_gallus_PARP1                    177  .......LDG.VEVP...LGNGIST......GI........NDTC...............L...................  197
Homo_sapiens_PARP1                     177  .......LDG.VDVP...LGTGISS......GV........NDTS...............L...................  197

Trichoplax_adhaerens_61730             172  ......LEND.VIVP...IGDINKA...................................E...................  187
Phytophthora_sojae_470114A             177  ......LPDG.IVVP...AGTLKPV.......A........GDQY...............L...................  197
Phytophthora_ramorum_40258             177  ......LPDG.VVVP...AGTLKPV.......A........GNQY...............L...................  197
Phytophthora_sojae_470114B             181  ......MDDG.VVVP...VGEFMPS.......D........GNGS...............L...................  201
Phytophthora_ramorum_85567             179  ......MADG.VVVP...IGEFTPS.......D........GSGS...............L...................  199
Paramecium_tetraurelia_A0CTW4          176  .........D.LKVP...IGKLEDQ.......Q........IKSD...............L...................  193

Phytophthora_sojae_30349               190  ......DIHG.AVWP...VGKPVQP.......E........ERTG...............L...................  210
Dictyostelium_discoideum_Q8I7C5        176  ..GCYQDKDE.FIPSSVLKGPIIET.....GS.........IVTS...............F...................  204
Tetrahymena_thermophila_Q24GE4         176  ......IYDD.VQVP...VGKGEPQ...VFPNG........QYGS...............L...................  200
Paramecium_tetraurelia_A0DM45          170  ....MPGLPG.VKVP...IGKPEPS......DV.......EKKSG...............L...................  194
Paramecium_tetraurelia_A0CA47          174  ....FPGMPE.VQVP...IGKGAPS......GV........ANTS...............L...................  197
Batrachochytrium_dendrobatidis_16284   173  ......LEDG.VVVP...AGSNLET......KG........SNGY...............L...................  194
Oryza_sativa_japonica_PRP2B            178  ......TDDG.MVIP...LGKPEKV......PS........RRGS...............L...................  199
Zea_mays_PARP2                         178  ......ADDG.VVVP...LGEPKQE......PS........KRGG...............L...................  199
Oryza_sativa_japonica_PRP2A            178  ......TDDG.VVVP...LGKPKAE......PS........KRGS...............L...................  199
Vitis_vinifera_D7U2A8                  179  ......FENG.IVVP...LGKPKLR......SD........PKGG...............L...................  200
Arabidopsis_thaliana_PARP2             178  ......LEDG.VVVP...LGKPVER......SC........SKGM...............L...................  199
Physcomitrella_patens_150949           178  ......LENG.VVVP...VGQPISS......PL........SMGC...............L...................  199
Selaginella_moellendorffii_83360       178  ......LPDG.VVVP...LGKPVKS......PN........SNTS...............L...................  199
Selaginella_moellendorffii_76668       178  ......LSDG.LIVP...MGKPLTS.....GAY........HQGS...............L...................  200
Xenopus_laevis_Q566G1                  178  ......SPDG.VVYP...IGKPQAS......GQ........KGTS...............L...................  199
Schistosoma_japonicum_Q5DAZ0           175  ......NKDG.VVYP...IGKPIDS......NV........PNTT...............L...................  196

Schizophyllum_commune_232036           188  EKLGRDDLRG.VLMP...GGHAKDL......SS......TFPRPS..........VKCED....................  221
Phanerochaete_chrysosporium_7882       185  EALDYPDLRG.VHMP...KGPAADVSLDPTTGN........PHIG...............L...................  218
Schizophyllum_commune_231882           185  EVLGNDALKG.CHMP...DGPCDNN......AD........PKAY...............L...................  212
Cryphonectria_parasitica_77865         185  ..CIHPSLQG.VKVPDTKAAPPKRN......DS........IASS...............L...................  213
Mycosphaerella_graminocola_51199       184  ..CVHPSLAG.VQMPDTVTKPPGDT......TV........LDAC...............L...................  212
Magnaporthe_grisea_A4QS91              185  ..GVHDSLKG.VKMPDT.SVKPGNT......NY........PNAW...............L...................  212
Phaeosphaeria_nodorum_Q0V159           185  ..CVNKSLAG.CIMPDM.EFNPGPT......NV........SGAY...............L...................  212
Cochliobolus_heterostrophus_25532      185  ..CINPALKG.CTMPDV.STPAGKT......NV........SGAT...............L...................  212
Pyrenophora_tritici-repentis_10572     185  ..CLNPTLAG.CMMPDV.SMPPGPT......MI........PNAY...............L...................  212
Chaetomium_globosum_Q2GML6             185  ..CVHPNLAG.VKMPDT.SVLPGAT......NV........PGAG...............L...................  212
Sporotrichum_thermophile_10727         185  ..CVHPSLVG.VKMPDT.SVPPGPT......DV........KGAG...............L...................  212
Nectria_haematococca_49036             185  ..IVHESLKG.IKMPDT.KVKPGPT......NV........KNAS...............L...................  212
Trichoderma_atroviride_295780          186  ..VVHESLKG.IQMPDI.TEKPCAT......NV........DGAY...............L...................  213
Emericella_nidulans_Q6V7K2             182  ..CIHPHLKG.IQVPDADSGTTDHK.......T........DQGY...............L...................  209
Aspergillus_terreus_Q0CPK1             186  ..CVHPSLQG.VSMPD..METPLDR.......N........TGKC...............L...................  211
Aspergillus_oryzae_Q2ULM4              186  ..SVNPQLQG.VKMPDV.SASSKSA................NAQS...............L...................  211
Aspergillus_fumigatus_Q4WU62           186  ..CLHPALSG.VQMPD..VSKSTMS................DNGS...............L...................  210
Aspergillus_clavatus_A1CA63            186  ..CVNPALAG.VQMPD..VAQPATS.......A........NAPS...............L...................  211
Ajellomyces_capsulata_A6QZQ6           186  ..CVHSDLSG.VLMPDV.STPPKLQ......DD........TNAW...............L...................  213
C5P795_Coccidioides_posadasii          186  ..SVHEDLAG.VLMPDT.TEAPRSC.......D......DGQNRS...............L...................  214

Emiliania_huxleyi_459854               176  ......IEGGAIRVP...MGEAGDS......EY........MKANFARLQKAEGGKARAAL...................  213
Toxoplasma_gondii_TGME49_070840        179  ..LPSVVDDEKVIAR...TGSCTAN..KTALEEAKKDEAAGTDAA...............L...................  217
Emiliania_huxleyi_41396                183  ....LPSDPA.VLVP...KGKAKKT......AV........AGSS...............L...................  206

Trichoplax_adhaerens_23639             175  ......LSNG.VVVP...LGKVMNT...GVNNP........GGYT...............L...................  199
Xenopus_laevis_Q6DFH2                  176  .......HEG.AVVP...LGPLMDT...GLTND........GGYT...............L...................  199
Danio_rerio_A5PLJ8                     176  .......LNG.VTVP...LGPSVKT...GVGQK........GGYS...............L...................  199
Homo_sapiens_PARP2                     176  .......LNG.STVP...LGPASDT...GILNP........DGYT...............L...................  199
Nematostella_vectensis_A7RQ13          174  ......RPDG.LTIP...CGKGIAT......GV.....TSPNGYT...............L...................  198
Monosiga_brevicollis_27847             135  ..................CDERADA.......................................................  141

Trypanosoma_cruzi_Q4PQV7               177  .......VDG.VLWP...KGRVRES.......R........ISSS...............L...................  196
Trypanosoma_brucei_brucei_Q0PW89       178  .......VKG.VLWP...KGCIKED.......S........RPTS...............L...................  197
Physcomitrella_patens_76575            232  ......FEED.ITVP...CGPLIPG.......K........EDSP...............L...................  252
Selaginella_moellendorffii_73333       181  ..W....KNN.ITVP...CGALKSS......GR........DDCT...............L...................  202
Oryza_sativa_japonica_PARP3            183  ..W....RDG.VTVP...CGKLVPS......TN........KDGP...............L...................  204
Vitis_vinifera_A5AUF8                  185  ..W....EDD.VKVP...CGHLIXS......EY........KDSP...............L...................  206
Vitis_vinifera_D7TCW5                  181  ..W....EDD.VKVP...CGHLIPS......EY........KDSP...............L...................  202
Arabidopsis_thaliana_PARP3             181  ..W....RDD.IKVP...CGRLVPS......EH........KDSP...............L...................  202
Medicago_truncatula_PARP3              181  ..W....KDD.IKVP...CGSIIAS......EH........EDSP...............L...................  202
Monosiga_brevicollis_16448             174  ....VEFDGKPVKVP...QGAPLDQ.....PKY........AQSS...............F...................  199
Homo_sapiens_PARP3                     173  ....LELDGQQVVVP...QGQPVPC.....PEF........SSST...............F...................  198
Danio_rerio_Q7ZVB0                     167  ....IELDGKKVVVP...QGKVIKQ.....QQY........EGSH...............F...................  192
Xenopus_laevis_Q5M9A2                  170  ....LALDGRKITVP...QGPPIKM.....EKY........NCTS...............F...................  195
Dictyostelium_discoideum_Q7Z11         176  ....LSDSDG.LVVP...LGKISKT......GL........STSC...................................  198
Tetrahymena_thermophila_Q24FE8         165  ...........IGTTQCFAGKVVES......DE........NGDG...........QPKDL...................  189
Paramecium_tetraurelia_A0BXK8          177  .......INN.LDFE...SGKVIPNEELKIHNI.........ESS...............L...................  202
Dictyostelium_discoideum_Q8I7C5        173  ......DTEG.CITP...YGQMIPS......QH........KGVS...............C...................  194
Selaginella_moellendorffii_90144       178  ......FDDG.VVVP...CGRPVPS.......Q........VRSD...............L...................  198
Physcomitrella_patens_188096           178  ......FEGDVTVP...CGQPVPS......GI.........RSD...............L...................  198
Zea_mays_PARP1                         178  ..W....RDD.VVVP...CGKPVPS......SI........RSSE...............L...................  199
Oryza_sativa_japonica_PARP1            178  ..W....RDD.VVVP...CGKPVPA......SI........KTSE...............L...................  199
Vitis_vinifera_A5AIW8                  176  ..W....RDE.VVVP...CGKPVPS......NV........KSTE...............L...................  197
Arabidopsis_thaliana_PARP1             178  ..W....RGD.VTVP...CGKPVSS......KV........KASE...............L...................  199

Caenorhabditis_elegans_PME1            175  ......HPDG.YTIP...LGLTYMQ..LQGKQD........VDYH...............L...................  200
Caenorhabditis_elegans_PME2            180  .........G...IPA..FKSNLET......IE........EETR...............L...................  197
Homo Sapiens PARP1 Structure                 β-sheet                                                   β-sheet
```

		410 420 430 440 450 460 470 480	
Mucor_circinelloides_81803	213	....	212
Rhizopus_oryzae_RO3G_04955	199	....	198
Paramecium_tetraurelia_A0DIP7	231	....	230
Paramecium_tetraurelia_A0DRQ5	230	....	229
Paramecium_tetraurelia_A0BYL4	231	....	230
Paramecium_tetraurelia_A0CWK3	215	....	214
Phytophthora_sojae_160067	267	STHTVSSTGNAREHFVQYQYEQRTIVIEVVNREIFDTGDDDDEPDAGSAVPHKAGSGAWCEATLKVTIRPDDGTAYSYLA	346
Dictyostelium_discoideum_Q54E42	195	....	194
Naegleria_gruberi_71853	204	....	203
Naegleria_gruberi_58866	202	....	201
Monosiga_brevicollis_26401	181	....	180
Drosophila_melanogaster_PARP	199	....	198
Trichoplax_adhaerens_19562	199	....	198
Nematostella_vectensis_A7RNW1	200	....	199
Danio_rerio_Q499A8	196	....	195
Xenopus_laevis_PARP1	198	....	197
Gallus_gallus_PARP1	198	....	197
Homo_sapiens_PARP1	198	....	197
Trichoplax_adhaerens_61730	188	....	187
Phytophthora_sojae_470114A	198	....	197
Phytophthora_ramorum_40258	198	....	197
Phytophthora_sojae_470114B	202	....	201
Phytophthora_ramorum_85567	200	....	199
Paramecium_tetraurelia_A0CTW4	194	....	193
Phytophthora_sojae_30349	211	....	210
Dictyostelium_discoideum_Q8I7C5	205	....	204
Tetrahymena_thermophila_Q24GE4	201	....	200
Paramecium_tetraurelia_A0DM45	195	....	194
Paramecium_tetraurelia_A0CA47	198	....	197
Batrachochytrium_dendrobatidis_16284	195	....	194
Oryza_sativa_japonica_PRP2B	200	....	199
Zea_mays_PARP2	200	....	199
Oryza_sativa_japonica_PRP2A	200	....	199
Vitis_vinifera_D7U2A8	201	....	200
Arabidopsis_thaliana_PARP2	200	....	199
Physcomitrella_patens_150949	200	....	199
Selaginella_moellendorffii_83360	200	....	199
Selaginella_moellendorffii_76668	201	....	200
Xenopus_laevis_Q566G1	200	....	199
Schistosoma_japonicum_Q5DAZ0	197	....	196
Schizophyllum_commune_232036	222	....	221
Phanerochaete_chrysosporium_7882	219	....	218
Schizophyllum_commune_231882	213	....	212
Cryphonectria_parasitica_77865	214	....	213
Mycosphaerella_graminocola_51199	213	....	212
Magnaporthe_grisea_A4QS91	213	....	212
Phaeosphaeria_nodorum_Q0V159	213	....	212
Cochliobolus_heterostrophus_25532	213	....	212
Pyrenophora_tritici-repentis_10572	213	....	212
Chaetomium_globosum_Q2GML6	213	....	212
Sporotrichum_thermophile_10727	213	....	212
Nectria_haematococca_49036	213	....	212
Trichoderma_atroviride_295780	214	....	213
Emericella_nidulans_Q6V7K2	210	....	209
Aspergillus_terreus_Q0CPK1	212	....	211
Aspergillus_oryzae_Q2ULM4	212	....	211
Aspergillus_fumigatus_Q4WU62	211	....	210
Aspergillus_clavatus_A1CA63	212	....	211
Ajellomyces_capsulata_A6QZQ6	214	....	213
C5P795_Coccidioides_posadasii	215	....	214
Emiliania_huxleyi_459854	214	....	213
Toxoplasma_gondii_TGME49_070840	218	....	217
Emiliania_huxleyi_41396	207	....	206
Trichoplax_adhaerens_23639	200	....	199
Xenopus_laevis_Q6DFH2	200	....	199
Danio_rerio_A5PLJ8	200	....	199
Homo_sapiens_PARP2	200	....	199
Nematostella_vectensis_A7RQ13	199	....	198
Monosiga_brevicollis_27847	141	....	141
Trypanosoma_cruzi_Q4PQV7	197	....	196
Trypanosoma_brucei_brucei_Q0PW89	198	....	197
Physcomitrella_patens_76575	253	....	252
Selaginella_moellendorffii_73333	203	....	202
Oryza_sativa_japonica_PARP3	205	....	204
Vitis_vinifera_A5AUF8	207	....	206
Vitis_vinifera_D7TCW5	203	....	202
Arabidopsis_thaliana_PARP3	203	....	202
Medicago_truncatula_PARP3	203	....	202
Monosiga_brevicollis_16448	200	....	199
Homo_sapiens_PARP3	199	....	198
Danio_rerio_Q7ZVB0	193	....	192
Xenopus_laevis_Q5M9A2	196	....	195
Dictyostelium_discoideum_Q7Z11	199	....	198
Tetrahymena_thermophila_Q24FE8	190	....	189
Paramecium_tetraurelia_A0BXK8	203	....	202
Dictyostelium_discoideum_Q8I7C5	195	....	194
Selaginella_moellendorffii_90144	199	....	198
Physcomitrella_patens_188096	199	....	198
Zea_mays_PARP1	200	....	199
Oryza_sativa_japonica_PARP1	200	....	199
Vitis_vinifera_A5AIW8	198	....	197
Arabidopsis_thaliana_PARP1	200	....	199
Caenorhabditis_elegans_PME1	201	....	200
Caenorhabditis_elegans_PME2	198	....	197
Homo Sapiens PARP1 Structure		β-sheet	

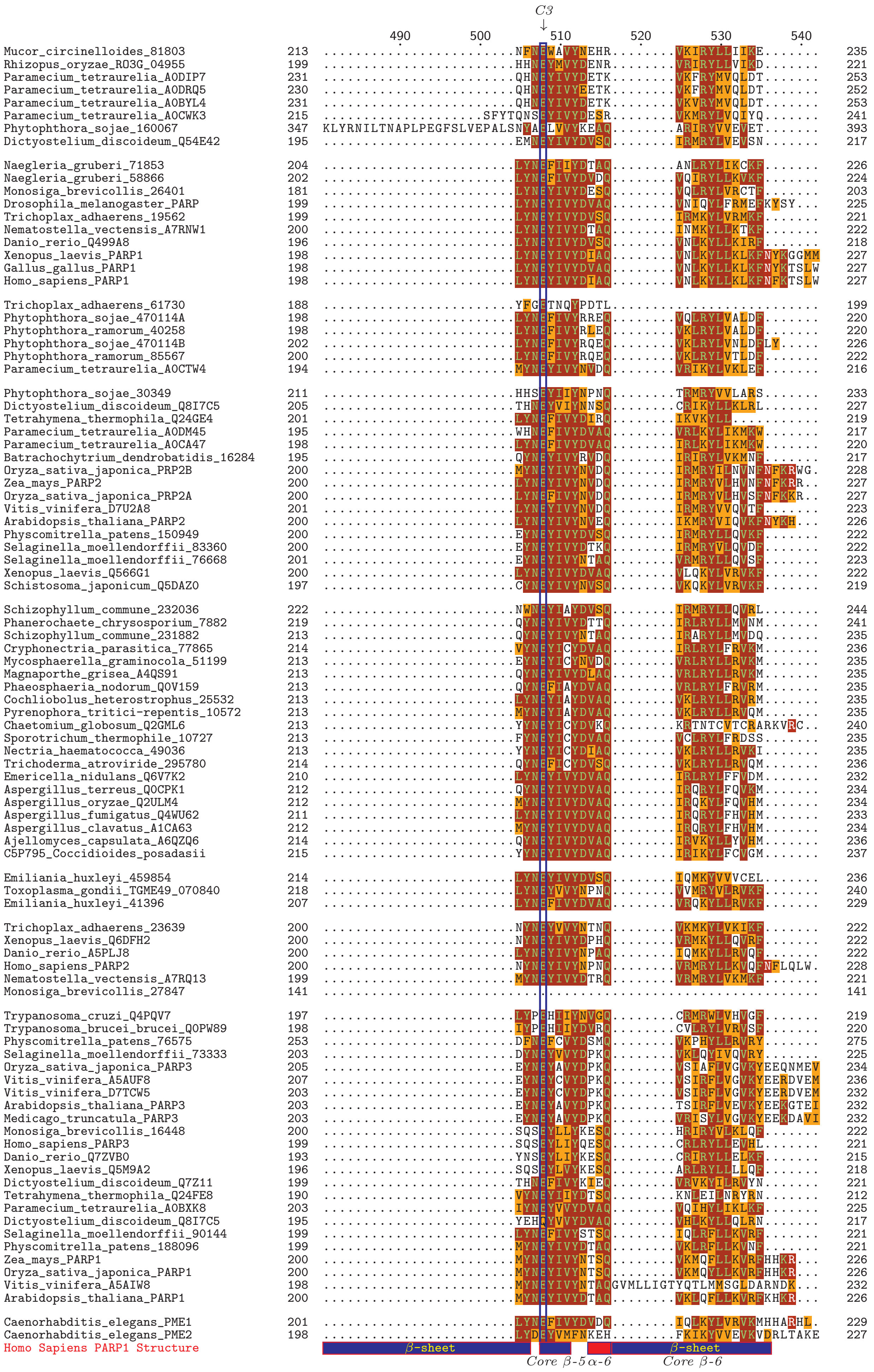
C3
490 500 510 520 530 540
Mucor_circinelloides_81803 213 ........................NFNEWAVYNEHR.......VKIRYLLIIKE....... 235
Rhizopus_oryzae_RO3G_04955 199 ........................HHNEYMVYDENR.......VRIRYLLVIKD....... 221
Paramecium_tetraurelia_A0DIP7 231 ........................QHNEYIVYDETK.......VKFRYMVQLDT....... 253
Paramecium_tetraurelia_A0DRQ5 230 ........................QHNEYIVYEETK.......VKFRYMVQLDT....... 252
Paramecium_tetraurelia_A0BYL4 231 ........................QHNEYIVYDETK.......VKVRYMVQLDT....... 253
Paramecium_tetraurelia_A0CWK3 215 ...................SFYTQNSEYIVYDESR.......VKMRYLVQIYQ....... 241
Phytophthora_sojae_160067 347 KLYRNILTNAPLPEGFSLVEPALSNYAELVVYKEAQ.......ARIRYVVEVET....... 393
Dictyostelium_discoideum_Q54E42 195 ........................EMNEYIVYDVSQ.......IRMRYLVEVSN....... 217
Naegleria_gruberi_71853 204 ........................LYNEFIIYDTAQ.......ANLRYLIKCKF....... 226
Naegleria_gruberi_58866 202 ........................LYNEFIVYDVDQ.......VQIRYLLKVKF....... 224
Monosiga_brevicollis_26401 181 ........................LYNEYIVYDESQ.......VQLRYLVRCTF....... 203
Drosophila_melanogaster_PARP 199 ........................LYNEYIVYDVAQ.......VNIQYLFRMEFKYSY... 225
Trichoplax_adhaerens_19562 199 ........................LYNEYIVYDVSQ.......IRMKYLVRMKF....... 221
Nematostella_vectensis_A7RNW1 200 ........................LYNEYIVYDTAQ.......INMKYLLKTKF....... 222
Danio_rerio_Q499A8 196 ........................LYNEYIVYDVSQ.......VNLKYLLKIRF....... 218
Xenopus_laevis_PARP1 198 ........................LYNEYIVYDIAQ.......VNLKYLLKLKFNYKGGMM 227
Gallus_gallus_PARP1 198 ........................LYNEYIVYDVAQ.......VNLKYLLKLKFNYKTSLW 227
Homo_sapiens_PARP1 198 ........................LYNEYIVYDIAQ.......VNLKYLLKLKFNFKTSLW 227
Trichoplax_adhaerens_61730 188 ........................YFGETNQYPDTL.............................. 199
Phytophthora_sojae_470114A 198 ........................LYNEFIVYRREQ.......VQLRYLVALDF....... 220
Phytophthora_ramorum_40258 198 ........................LYNEFIVYRLEQ.......VKLRYLVALDF....... 220
Phytophthora_sojae_470114B 202 ........................LYNEFIVYRQEQ.......VKLRYLVNLDFLY..... 226
Phytophthora_ramorum_85567 200 ........................LYNEFIVYRQEQ.......VKLRYLVTLDF....... 222
Paramecium_tetraurelia_A0CTW4 194 ........................MYNEYIVYNVDQ.......VRIKYLVKLEF....... 216
Phytophthora_sojae_30349 211 ........................HHSEYIIYNPNQ.......TRMRYVVLARS....... 233
Dictyostelium_discoideum_Q8I7C5 205 ........................THNEYVIYNNSQ.......CRIKYLLKLRL....... 227
Tetrahymena_thermophila_Q24GE4 201 ........................LYNEFIVYDIRQ.......IKVKYLL.......... 219
Paramecium_tetraurelia_A0DM45 195 ........................WHNEFIVYDVAQ.......VRLKYLIKMKW....... 217
Paramecium_tetraurelia_A0CA47 198 ........................LYNEFIVYDVAQ.......IRLKYLIKMKW....... 220
Batrachochytrium_dendrobatidis_16284 195 ........................QYNEYIVYRVDQ.......IRIRYLVKMNF....... 217
Oryza_sativa_japonica_PRP2B 200 ........................MYNEYIVYNVDQ.......IRMRYILNVNFNFKRWG. 228
Zea_mays_PARP2 200 ........................LYNEYIVYNVDQ.......IRMRYVLHVNFNFKRR.. 227
Oryza_sativa_japonica_PRP2A 200 ........................LYNEFIVYNVDQ.......IRMRYVLHVSFNFKKR.. 227
Vitis_vinifera_D7U2A8 201 ........................LYNEYIVYNVDQ.......IRMRYVVQVTF....... 223
Arabidopsis_thaliana_PARP2 200 ........................LYNEYIVYNVEQ.......IKMRYVIQVKFNYKH... 226
Physcomitrella_patens_150949 200 ........................EYNEYIVYDVSQ.......IRMRYLLQVKF....... 222
Selaginella_moellendorffii_83360 200 ........................EYNEYIVYDTKQ.......IRMRYVLQVDF....... 222
Selaginella_moellendorffii_76668 201 ........................EYNEYIVYNTAQ.......VRMRYLLQVSF....... 223
Xenopus_laevis_Q566G1 200 ........................LYNEYIVYDVAQ.......VLQKYLVRVKF....... 222
Schistosoma_japonicum_Q5DAZ0 197 ........................CYNEYIVYNVSQ.......VKQKYLVRVKF....... 219
Schizophyllum_commune_232036 222 ........................NWNEYIAYDVSQ.......IRMRYLLQVRL....... 244
Phanerochaete_chrysosporium_7882 219 ........................QYNEYIVYDTTQ.......IRLRYLLMVNM....... 241
Schizophyllum_commune_231882 213 ........................QYNEYIVYNTAQ.......IRARYLLMVDQ....... 235
Cryphonectria_parasitica_77865 214 ........................VYNEYICYDVAQ.......IRLRYLFRVKM....... 236
Mycosphaerella_graminocola_51199 213 ........................EYNEYICYNVDQ.......VRLRYLLRVKM....... 235
Magnaporthe_grisea_A4QS91 213 ........................QYNEYIVYDLAQ.......VRLRYLLRVKM....... 235
Phaeosphaeria_nodorum_Q0V159 213 ........................QYNEFIAYDVAQ.......VRLRYLFRVRM....... 235
Cochliobolus_heterostrophus_25532 213 ........................LYNEYIAYDVAQ.......VKLRYLLRVRM....... 235
Pyrenophora_tritici-repentis_10572 213 ........................MYNEYIAYDVAQ.......VKLRYLLRVQM....... 235
Chaetomium_globosum_Q2GML6 213 ........................YYNEYICYDVKQ.......KRTNTCVTCRARKVRC.. 240
Sporotrichum_thermophile_10727 213 ........................FYNEYICYDVAQ.......VCLRYLFRDSS....... 235
Nectria_haematococca_49036 213 ........................YYNEYICYDIAQ.......VKLRYLLRVKI....... 235
Trichoderma_atroviride_295780 214 ........................QYNEFICYDVSQ.......VKLRYLLRVQM....... 236
Emericella_nidulans_Q6V7K2 210 ........................LYNEYIVYDVAQ.......IRLRYLFFVDM....... 232
Aspergillus_terreus_Q0CPK1 212 ........................QYNEYIVYDVAQ.......IRQRYLFQVKM....... 234
Aspergillus_oryzae_Q2ULM4 212 ........................MYNEYIVYDVAQ.......IRQKYLFQVHM....... 234
Aspergillus_fumigatus_Q4WU62 211 ........................LYNEYIVYDVAQ.......IRQRYLFHVHM....... 233
Aspergillus_clavatus_A1CA63 212 ........................MYNEYIVYDVAQ.......IRQRYLFHVHM....... 234
Ajellomyces_capsulata_A6QZQ6 214 ........................QYNEYIVYDVAQ.......IRVKYLLYVHM....... 236
C5P795_Coccidioides_posadasii 215 ........................YYNEYIVYDVAQ.......IRIKYLFCVGM....... 237
Emiliania_huxleyi_459854 214 ........................LYNEYIVYDVSQ.......IQMKYVVVCEL....... 236
Toxoplasma_gondii_TGME49_070840 218 ........................LYNEYVVYNPNQ.......VVMRYVLRVKF....... 240
Emiliania_huxleyi_41396 207 ........................LYNEFIVYDVAQ.......VRQKYLLRVKF....... 229
Trichoplax_adhaerens_23639 200 ........................NYNEYVVYNTNQ.......VKMKYLVKIKF....... 222
Xenopus_laevis_Q6DFH2 200 ........................NYNEYIVYDPHQ.......VRMKYLLQVRF....... 222
Danio_rerio_A5PLJ8 200 ........................LYNEYIVYNPAQ.......IQMKYLLRVQF....... 222
Homo_sapiens_PARP2 200 ........................NYNEYIVYNPNQ.......VRMRYLLKVQFNFLQLW. 228
Nematostella_vectensis_A7RQ13 199 ........................MYNEYIVYDTRQ.......VRMRYLVKMKF....... 221
Monosiga_brevicollis_27847 141 141
Trypanosoma_cruzi_Q4PQV7 197 ........................LYPEHIIYNVGQ.......CRMRWLVHVGF....... 219
Trypanosoma_brucei_brucei_Q0PW89 198 ........................IYPEHIIYDVRQ.......CVLRYLVRVSF....... 220
Physcomitrella_patens_76575 253 ........................DFNEFCVYDSMQ.......VKPHYLLRVRY....... 275
Selaginella_moellendorffii_73333 203 ........................DYNEYVVYDPKQ.......VKLQYIVQVRY....... 225
Oryza_sativa_japonica_PARP3 205 ........................EYNEYAVYDPKQ.......VSIAFLVGVKYEEQNMEV 234
Vitis_vinifera_A5AUF8 207 ........................EYNEYCVYDPKQ.......VSIRFLVGVKYEERDVEM 236
Vitis_vinifera_D7TCW5 203 ........................EYNEYCVYDPKQ.......VSIRFLVGVKYEERDVEM 232
Arabidopsis_thaliana_PARP3 203 ........................EYNEYAVYDPKQ.......TSIRFLVEVKYEEKGTEI 232
Medicago_truncatula_PARP3 203 ........................EYNEYAVYDPKQ.......VRISYLVGVKYEEKDAVI 232
Monosiga_brevicollis_16448 200 ........................SQSEYLLYKESQ.......HRIRYVLKLQF....... 222
Homo_sapiens_PARP3 199 ........................SQSEYLIYQESQ.......CRLRYLLEVHL....... 221
Danio_rerio_Q7ZVB0 193 ........................YNSEYLIYKESQ.......CRIRYLLELKF....... 215
Xenopus_laevis_Q5M9A2 196 ........................SQSEYLVYKESQ.......ARLRYLLLQF........ 218
Dictyostelium_discoideum_Q7Z11 199 ........................THNEFIVYKIEQ.......VRVKYILRVYN....... 221
Tetrahymena_thermophila_Q24FE8 190 ........................VYNEYIIYDTSQ.......KNLEILNRYRN....... 212
Paramecium_tetraurelia_A0BXK8 203 ........................IYNEYVVYDVAQ.......VQIHYLIKLKF....... 225
Dictyostelium_discoideum_Q8I7C5 195 ........................YEHQYVVYDVAQ.......VHLKYLLQLRN....... 217
Selaginella_moellendorffii_90144 199 ........................LYNEFIVYSTSQ.......IQLRFLLKVRF....... 221
Physcomitrella_patens_188096 199 ........................MYNEYIVYDTAQ.......VKLRFLLKVNF....... 221
Zea_mays_PARP1 200 ........................MYNEYIVYNTSQ.......VKMQFLLKVRFHHKR... 226
Oryza_sativa_japonica_PARP1 200 ........................MYNEYIVYNTSQ.......VKMQYLLKVRFHHKR... 226
Vitis_vinifera_A5AIW8 198 ........................MYNEYIVYNTAQGVMLLIGTYQTLMMSGLDARNDK... 232
Arabidopsis_thaliana_PARP1 200 ........................MYNEYIVYDTAQ.......VKLQFLLKVRFKHKR... 226
Caenorhabditis_elegans_PME1 201 ........................LYNEFIVYDVDQ.......IQLKYLVRVKMHHARHL. 229
Caenorhabditis_elegans_PME2 198 ........................LYDEYVMFNKEH.......FKIKYVVEVKVDRLTAKE 227
Homo Sapiens PARP1 Structure
β-sheet
β-sheet
Core β-5 α-6
Core β-6
