# Supplementary material for: Evolutionary history of the poly(ADP-ribose) polymerase gene family in eukaryotes
Source: BMC Evol Biol. 2010 Oct 13;10:308. doi: 10.1186/1471-2148-10-308 (PMC2964712; doi:10.1186/1471-2148-10-308)
Supplement: Additional file 8 — Multiple alignment of the PARP catalytic domains of Clade 3 proteins annotated with structural information. These alignments only show the conserved PARP catalytic domain. The structural elements present in Homo sapiens PARP15 are shown at the bottom of the alignment. The crystal structure of PARP15 is available in the Protein Data Bank (PDB; http://www.rcsb.org/pdb/explore/explore.do?structureId=3GEY; [171]). Annotations as in Additional file 5. [file 1471-2148-10-308-S8.PDF]

```
                                                    10        20        30        40        50        60        70        80
Homo_sapiens_PAR10                      1   ....PTLAGQTLKGPWNNLERLAENTGEFQEVVRAFYDTLDAARSSIRVVRVERVSHPLLQQQYELYRERLLQRC.....   71
Danio_rerio_XP_693301                   1   .............SMPEVSTVDVNSDEFQDVVKEFYDTLQDNHNKIRIIKVEKLMNKLLHDQYRLKKASIEQST.....   61

Trichoplax_adhaerens_B3S3M3             1   .......DSIDEVKYGPPRIMDVVNEKERKELFDLIGSRGIK......VTKFQRVQNWNLYYHFQQRKKEIEASVIKYK.   66
Trichoplax_adhaerens_B3RYG9             1   ................YYHAVDVTNQHEISEITSLLGFT......GLRVRNITRIQNWNLYRRYQIMKKDVENAIRKYK.   57
Ciona_intestinalis_Q69HN2               1   .....HWGNV..SRGTTEVKQLTPDSSEFNDVVAAFKKSNPPVN...EILQIERIQNPTLYKQYEGKREAIVDKV.....   65

Branchiostoma_floridae_C3Y6H9           1   ...PDHWEQMT.EGETVKVVDLQPTSEEYKKVHDPFKSTLPSSASGAQVLKIQRIQNPRLWRQYQVRKDQMEFDN.....   71
Danio_rerio_Q3ZB96                      1   ...PQHWDTMP.PNDLNKKFNLQPTNKEYQDVLGRFKATCPNQN....VLKIERVQNPGMWKNYQNNKSVMEKKN.....   67
Gallus_gallus_XP_422113                 1   ...............QLKIVELKPDTKDYRQVKERFLNTSPSLN..LKIEKIERVQNPSLWKAYQIKKCQMDDKN.....   58
Homo_sapiens_PAR14                      1   ..IPAHWSDM..KQQNFCVVELLPSDPEYNTVASKFNQTCSHFR....IEKIERIQNPDLWNSYQAKKKTMDAKN.....   67
Homo_sapiens_PAR15                      1   ..LPEHWTDM..NHQLFCMVQLEPGQSEYNTIKDKFTRTCSSYA....IEKIERIQNAFLWQSYQVKKRQMDIKN.....   67
Nematostella_vectensis_A7T1E8           1   ...........................................................................MDKDN.....    5
Branchiostoma_floridae_C3Y5P2           1   ..LPEFWDPQ..GDDEVKVVELSEGSLEFQETYMYFTKTIGDMP..SKVVKIERIQNPALWRQYQVKKEKMDRTN.....   69

Dictyostelium_discoideum_DDB0232928     1   ...............ECCLVEIEKGGKEWLEIRTRMSETLPGVI....INKVEFVQNRSSYEDYYYKKNKIEAHNNGKSV   61
Dictyostelium_discoideum_DDB0304590     1   ...............QVFLIKINKGSSEYIIVSERFNETMSNSF...EIIKIERIQNKSLWRNFDESRKRLNEKYQ....   58
Tetrahymena_thermophila_Q22SD0          1   .................LLFSLSLNSVEAVKCINMFNQSMSNI.....VRKVYRIQNMNLWKNYQFEKETLLQ.......   51
Tetrahymena_thermophila_Q22SC9          1   ................LLLVNLDLSSPEAMDCITAMKKTLPNSK....VSRIQR..........................   34
Tetrahymena_thermophila_Q24C77          1   ................LKLVTINLNSDEAKKVIQLFQNSMQNMP....ITKIERIQNISLMKNYLFEKQKLKE.......   53
Tetrahymena_thermophila_Q22F17          1   ....DDWES...QDQDVEVFDVKLDSDYAKNIMKIVKKSIPNAK....FHKLERIQNLKLWKNFCFERKKLEEKG.....   64

Nematostella_vectensis_A7RWC0           1   ..................................MRQHPGKVK....VQAISRVQNPELWENFIRKKSQMSRKT.....   36
Homo_sapiens_ZCC2                       1   ....PQEDFCFLSSKKYKLSEIHHLHPEYVRVSEHFKASMKNFK....IEKIKKIENSELLDKFTWKKSQMKEE......   66
Xenopus_laevis_Q6DDP4                   1   ...............PYQLVPLLSVSNEYSEVVGRFGKTLDRSC....IISVHRVQNLDLWEFYCRKKAQLKNKKG....   57
Homo_sapiens_PAR11                      1   IPMPPHWENVN.TQVPYQLIPLHNQTHEYNEVANLFGKTMDRNR....IKRIQRIQNLDLWEFFCRKKAQLKKKRG....   71
Gallus_gallus_Q5F3B3                    1   ............PDFGYKEVEISTISSEYQEIKKQFEQTMKSCN....IIKILRIQNPSLWKVFQWQKEQMKRQSG....   60
Danio_rerio_Q5U400                      1   ..............TGCKRVQLTKTTAEFIKIQELFNRTMRGFT....IQNIERIQNKALWEVFQWQKDCMKKNSR....   58
Gallus_gallus_XP_416342                 1   ..............IGYKLVDVGNTSSEYRRIESLFQKTMKDYS....ICRLQRIQNPTLWQIFQWQKEQMKKLHK....   58
Danio_rerio_XP_695578                   1   ..............TYKIVSLQKTCKEYERVSSMFNRTLPGSI....IHRIDRVQNPSLWKVFEWQKEQMNLKSG....   57
Homo_sapiens_PAR12                      1   ..IPDYWDSSALPDPGFQKITLSSSSEEYQKVWNLFNRTLPFYF....VQKIERVQNLALWEVYQWQKGQMQKQNG....   70
Gallus_gallus_XP_416333                 1   ..............LGFKLIELDSSSEEYNKVKGDFQRTMPKTH....IKRICRIQNPSLWELYQWQKEQMQKSN.....   57
Nematostella_vectensis_A7S5J9           1   ...PKHWDPIP.DDKDFICVQLSSTSPEYKKIEAQFLSSLDTSR...RIVKIERIQNPDLWMQFAQKKERMEKKS.....   68
Branchiostoma_floridae_C3YBY8           1   ...PSTWTPMQ.AGQDFVRRPLSRLCPEFRVVEMLFRQTMGEEK...VIVRIERVQNPFLWEKYNRKKEYMRPRLHTQSS   73
Danio_rerio_NP_001038484                1   ..YPETWLPMN.NCQDFMRVPVSRDDRSYRTVYSLFHKTVSETK..FRILKILRVQNPFLWEKYKRKKEYMSRRMTDM..   73
Homo_sapiens_PARPT                      1   ..YPETWVYMH.PSQDFIQVPVSAEDKSYRIIYNLFHKTVPEFK..YRILQILRVQNQFLWEKYKRKKEYMNRKMFGR..   73
Xenopus_tropicalis_A8E4V7               1   ...PETWLSMD.ASQEFFQVPMSKEDKSYRTVYTLFHKTVPETR..FRILKIIRVQNLFLWEKYKRKKEFMTRKMTGL..   72
Gallus_gallus_XP_422828                 1   ..YPETWISMD.PSQDFIQVPVLKEDKSYRTIYNLFHKTVPETK..YKILKILRVQNQFLWEKYKRKKEYMSKKMTGL..   73

Danio_rerio_XP_001340167                1   .............NSFYARNPVT..KNYFLEIEGRKASTAFRLS....IIKVEKLENIVLKQLFDKNRQRIKCQ......   55
Xenopus_laevis_Q6NRC6                   1   ................LSELRLVEKLDLFSYCERELKKSKLE......IVKVEQIYNHVLFAVFNSKKNSIGDK......   52
Gallus_gallus_XP_422116                 1   ..........TNTTKQFQMLQVDSHLQAFKDRQKQFERTGLH......ILKIEKIHNPLLSAAFQQTKKNLEQKG.....   59
Homo_sapiens_PARP9                      1   ..IQQQKTQDEMKENIIFLKCPVPPTQELLDQKKQFEKC......GLQVLKVEKIDNEVLMAAFQRKKKMMEEKL.....   67
```

Homo Sapiens PAR15 Structure: α-helix (α-1), β-sheet, α-helix (α-2)

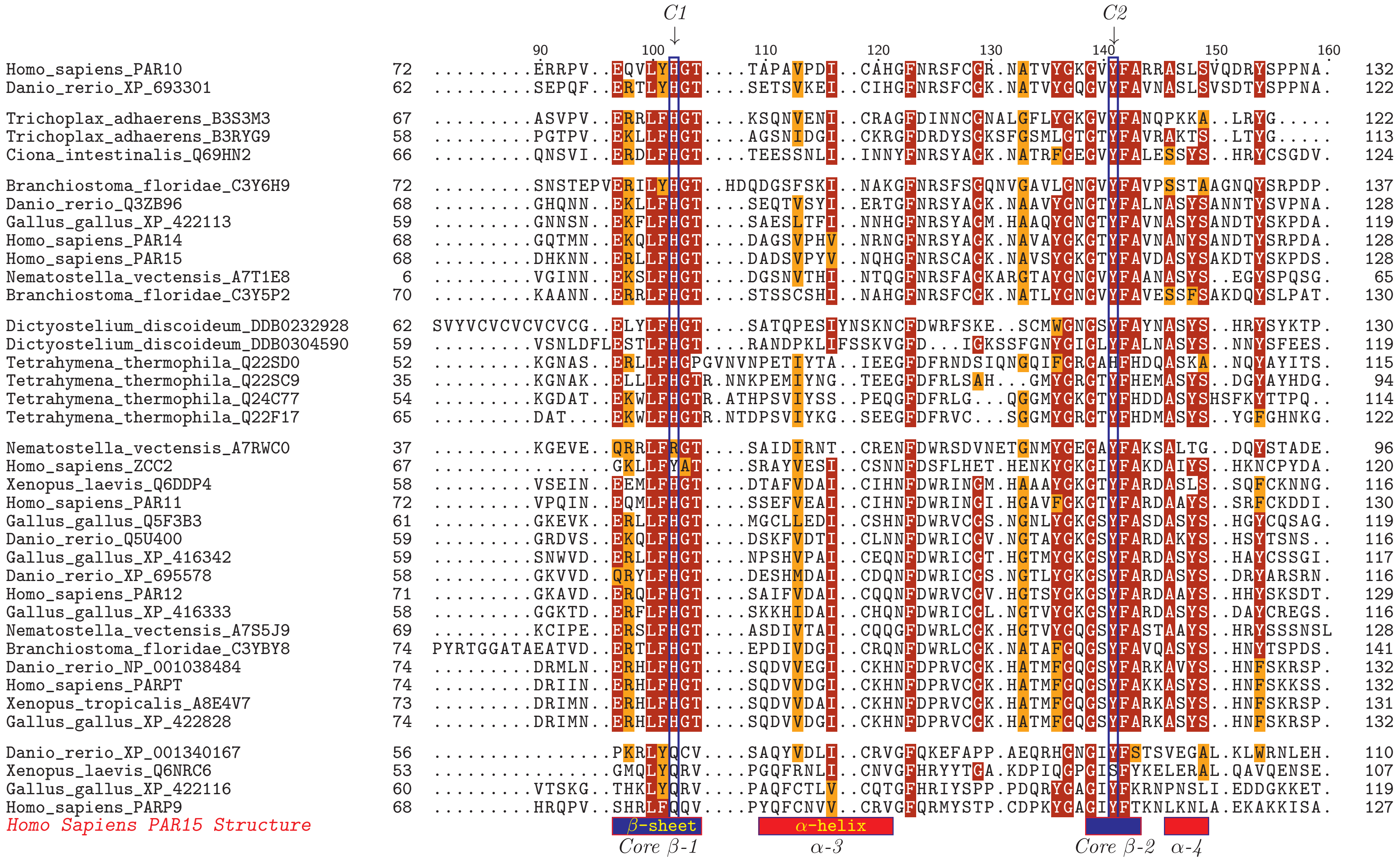
C1
C2
90 100 110 120 130 140 150 160
Homo_sapiens_PAR10 72 .........ERRPV..EQVLYHGT....TAPAVPDI..CAHGFNRSFCGR.NATVYGKGVYFARRASLSVQDRYSPPNA. 132
Danio_rerio_XP_693301 62 .........SEPQF..ERTLYHGT....SETSVKEI..CIHGFNRSFCGK.NATVYGQGVYFAVNASLSVSDTYSPPNA. 122
Trichoplax_adhaerens_B3S3M3 67 .........ASVPV..ERRLFHGT....KSQNVENI..CRAGFDINNCGNALGFLYGKGVYFANQPKKA..LRYG..... 122
Trichoplax_adhaerens_B3RYG9 58 .........PGTPV..EKLLFHGT....AGSNIDGI..CKRGFDRDYSGKSFGSMLGTGTYFAVRAKTS..LTYG..... 113
Ciona_intestinalis_Q69HN2 66 .........QNSVI..ERDLFHGT....TEESSNLI..INNYFNRSYAGK.NATRFGEGVYFALESSYS..HRYCSGDV. 124
Branchiostoma_floridae_C3Y6H9 72 .........SNSTEPVERILYHGT..HDQDGSFSKI..NAKGFNRSFSGQNVGAVLGNGVYFAVPSSTAAGNQYSRPDP. 137
Danio_rerio_Q3ZB96 68 .........GHQNN..EKLLFHGT....SEQTVSYI..ERTGFNRSYAGK.NAAVYGNGTYFALNASYSANNTYSVPNA. 128
Gallus_gallus_XP_422113 59 .........GNNSN..EKFLFHGT....SAESLTFI..NNHGFNRSYAGM.HAAQYGNGTYFAVNASYSANDTYSKPDA. 119
Homo_sapiens_PAR14 68 .........GQTMN..EKQLFHGT....DAGSVPHV..NRNGFNRSYAGK.NAVAYGKGTYFAVNANYSANDTYSRPDA. 128
Homo_sapiens_PAR15 68 .........DHKNN..ERLLFHGT....DADSVPYV..NQHGFNRSCAGK.NAVSYGKGTYFAVDASYSAKDTYSKPDS. 128
Nematostella_vectensis_A7T1E8 6 .........VGINN..EKSLFHGT....DGSNVTHI..NTQGFNRSFAGKARGTAYGNGVYFAANASYS..EGYSPQSG. 65
Branchiostoma_floridae_C3Y5P2 70 .........KAANN..ERRLFHGT....STSSCSHI..NAHGFNRSFCGK.NATLYGNGVYFAVESSFSAKDQYSLPAT. 130
Dictyostelium_discoideum_DDB0232928 62 SVYVCVCVCVCVCG..ELYLFHGT....SATQPESIYNSKNCFDWRFSKE..SCMWGNGSYFAYNASYS..HRYSYKTP. 130
Dictyostelium_discoideum_DDB0304590 59 .........VSNLDFLESTLFHGT....RANDPKLIFSSKVGFD...IGKSSFGNYGIGLYFALNASYS..NNYSFEES. 119
Tetrahymena_thermophila_Q22SD0 52 .........KGNAS..ERLLFHGPGVNVNPETIYTA..IEEGFDFRNDSIQNGQIFGRGAHFHDQASKA..NQYAYITS. 115
Tetrahymena_thermophila_Q22SC9 35 .........KGNAK..ELLLFHGTR.NNKPEMIYNG..TEEGFDFRLSAH...GMYGRGTYFHEMASYS..DGYAYHDG. 94
Tetrahymena_thermophila_Q24C77 54 .........KGDAT..EKWLFHGTR.ATHPSVIYSS..PEQGFDFRLG...QGGMYGKGTYFHDDASYSHSFKYTTPQ.. 114
Tetrahymena_thermophila_Q22F17 65 .........DAT....EKWLFHGTR.NTDPSVIYKG..SEEGFDFRVC...SGGMYGRGTYFHDMASYS..YGFGHNKG. 122
Nematostella_vectensis_A7RWC0 37 .........KGEVE..QRRLFRGT....SAIDIRNT..CRENFDWRSDVNETGNMYGEGAYFAKSALTG..DQYSTADE. 96
Homo_sapiens_ZCC2 67 ..............GKLLFYAT....SRAYVESI..CSNNFDSFLHET.HENKYGKGIYFAKDAIYS..HKNCPYDA. 120
Xenopus_laevis_Q6DDP4 58 .........VSEIN..EEMLFHGT....DTAFVDAI..CIHNFDWRINGM.HAAAYGKGTYFARDASLS..SQFCKNNG. 116
Homo_sapiens_PAR11 72 .........VPQIN..EQMLFHGT....SSEFVEAI..CIHNFDWRINGI.HGAVFGKGTYFARDAAYS..SRFCKDDI. 130
Gallus_gallus_Q5F3B3 61 .........GKEVK..ERLLFHGT....MGCLLEDI..CSHNFDWRVCGS.NGNLYGKGSYFASDASYS..HGYCQSAG. 119
Danio_rerio_Q5U400 59 .........GRDVS..EKQLFHGT....DSKFVDTI..CLNNFDWRICGV.NGTAYGKGSYFARDAKYS..HSYTSNS.. 116
Gallus_gallus_XP_416342 59 .........SNWVD..ERLLFHGT....NPSHVPAI..CEQNFDWRICGT.HGTMYGKGSYFARDASYS..HAYCSSGI. 117
Danio_rerio_XP_695578 58 .........GKVVD..QRYLFHGT....DESHMDAI..CDQNFDWRICGS.NGTLYGKGSYFARDASYS..DRYARSRN. 116
Homo_sapiens_PAR12 71 .........GKAVD..ERQLFHGT....SAIFVDAI..CQQNFDWRVCGV.HGTSYGKGSYFARDAAYS..HHYSKSDT. 129
Gallus_gallus_XP_416333 58 .........GGKTD..ERFLFHGT....SKKHIDAI..CHQNFDWRICGL.NGTVYGKGSYFARDASYS..DAYCREGS. 116
Nematostella_vectensis_A7S5J9 69 .........KCIPE..ERSLFHGT....ASDIVTAI..CQQGFDWRLCGK.HGTVYGQGSYFASTAAYS..HRYSSSNSL 128
Branchiostoma_floridae_C3YBY8 74 PYRTGGATAEATVD..ERTLFHGT....EPDIVDGI..CRQNFDWRLCGK.NATAFGQGSYFAVQASYS..HNYTSPDS. 141
Danio_rerio_NP_001038484 74 .........DRMLN..ERHLFHGT....SQDVVEGI..CKHNFDPRVCGK.HATMFGQGSYFARKAVYS..HNFSKRSP. 132
Homo_sapiens_PARPT 74 .........DRIIN..ERHLFHGT....SQDVVDGI..CKHNFDPRVCGK.HATMFGQGSYFAKKASYS..HNFSKKSS. 132
Xenopus_tropicalis_A8E4V7 73 .........DRIMN..ERHLFHGT....SQDVVDAI..CKHNFDPRVCGK.HATMFGQGSYFARKASYS..HNFSKRSP. 131
Gallus_gallus_XP_422828 74 .........DRIMN..ERHLFHGT....SQDVVDGI..CKHNFDPRVCGK.HATMFGQGSYFARKASYS..HNFSKRSP. 132
Danio_rerio_XP_001340167 56 ..............PKRLYQCV....SAQYVDLI..CRVGFQKEFAPP.AEQRHGNGIYFSTSVEGAL.KLWRNLEH. 110
Xenopus_laevis_Q6NRC6 53 ..............GMQLYQRV....PGQFRNLI..CNVGFHRYYTGA.KDPIQGPGISFYKELERAL.QAVQENSE. 107
Gallus_gallus_XP_422116 60 .........VTSKG..THKLYQRV....PAQFCTLV..CQTGFHRIYSPP.PDQRYGAGIYFKRNPNSLI.EDDGKKET. 119
Homo_sapiens_PARP9 68 .........HRQPV..SHRLFQQV....PYQFCNVV..CRVGFQRMYSTP.CDPKYGAGIYFTKNLKNLA.EKAKKISA. 127
Homo Sapiens PAR15 Structure
β-sheet
α-helix
Core β-1
α-3
Core β-2
α-4

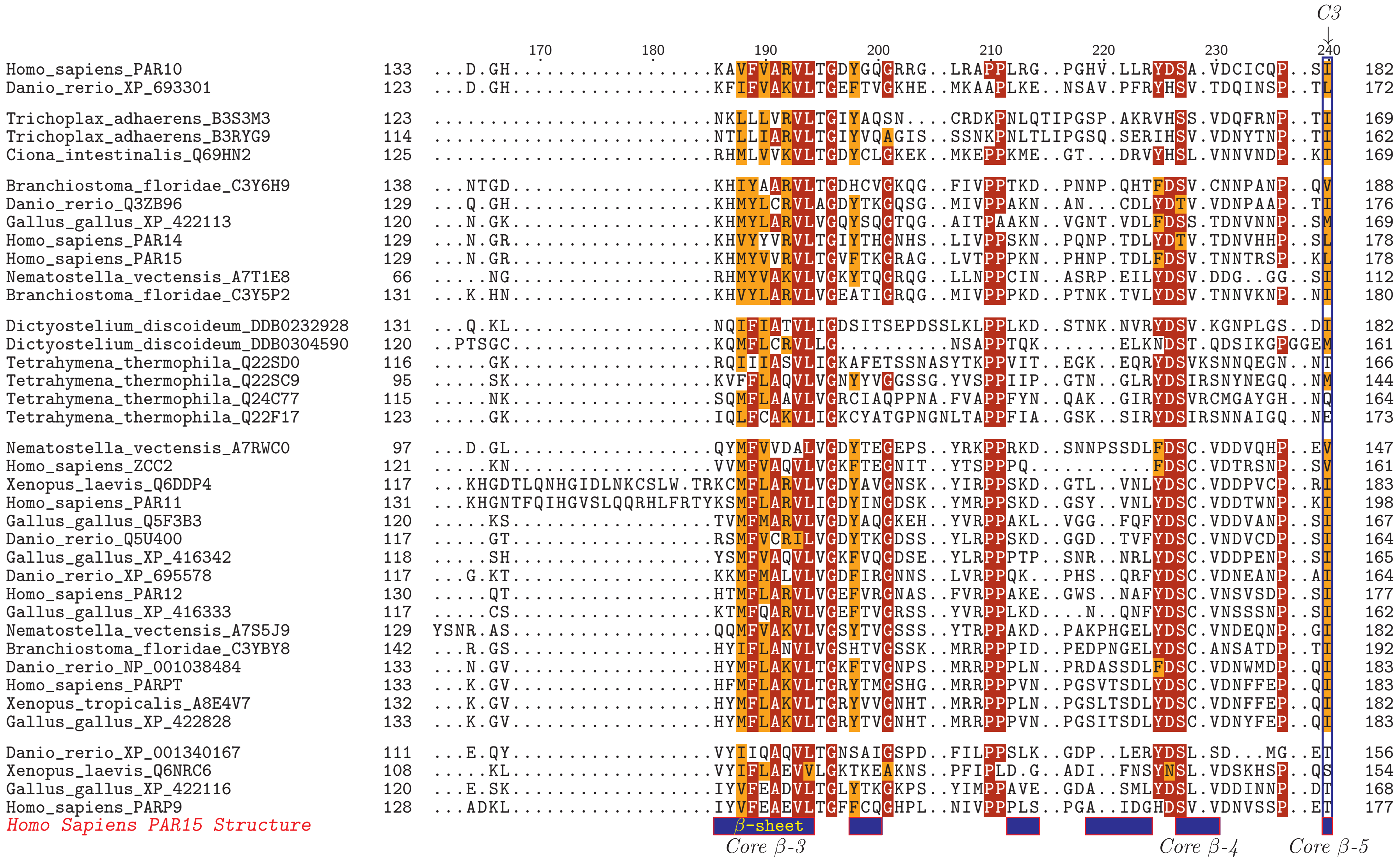
C3
170 180 190 200 210 220 230 240
Homo_sapiens_PAR10 133 ...D.GH.................KAVFVARVLTGDYGQGRRG..LRAPPLRG..PGHV.LLRYDSA.VDCICQP..SI 182
Danio_rerio_XP_693301 123 ...D.GH.................KFIFVAKVLTGEFTVGKHE..MKAAPLKE..NSAV.PFRYHSV.TDQINSP..TL 172
Trichoplax_adhaerens_B3S3M3 123 ........................NKLLLVRVLTGIYAQSN....CRDKPNLQTIPGSP.AKRVHSS.VDQFRNP..TI 169
Trichoplax_adhaerens_B3RYG9 114 ........................NTLLIARVLTGIYVQAGIS..SSNKPNLTLIPGSQ.SERIHSV.VDNYTNP..TI 162
Ciona_intestinalis_Q69HN2 125 ........................RHMLVVKVLTGDYCLGKEK..MKEPPKME..GT...DRVYHSL.VNNVNDP..KI 169
Branchiostoma_floridae_C3Y6H9 138 ...NTGD.................KHIYAARVLTGDHCVGKQG..FIVPPTKD..PNNP.QHTFDSV.CNNPANP..QV 188
Danio_rerio_Q3ZB96 129 ...Q.GH.................KHMYLCRVLAGDYTKGQSG..MIVPPAKN..AN...CDLYDTV.VDNPAAP..TI 176
Gallus_gallus_XP_422113 120 ...N.GK.................KHMYLARVLVGQYSQGTQG..AITPAAKN..VGNT.VDLFDSS.TDNVNNP..SM 169
Homo_sapiens_PAR14 129 ...N.GR.................KHVYYVRVLTGIYTHGNHS..LIVPPSKN..PQNP.TDLYDTV.TDNVHHP..SL 178
Homo_sapiens_PAR15 129 ...N.GR.................KHMYVVRVLTGVFTKGRAG..LVTPPPKN..PHNP.TDLFDSV.TNNTRSP..KL 178
Nematostella_vectensis_A7T1E8 66 ....NG..................RHMYVAKVLVGKYTQGRQG..LLNPPCIN..ASRP.EILYDSV.DDG..GG..SI 112
Branchiostoma_floridae_C3Y5P2 131 ...K.HN.................KHVYLARVLVGEATIGRQG..MIVPPPKD..PTNK.TVLYDSV.TNNVKNP..NI 180
Dictyostelium_discoideum_DDB0232928 131 ...Q.KL.................NQIFIATVLIGDSITSEPDSSLKLPPLKD..STNK.NVRYDSV.KGNPLGS..DI 182
Dictyostelium_discoideum_DDB0304590 120 ..PTSGC.................KQMFLCRVLLG...........NSAPTQK.......ELKNDST.QDSIKGPGGEM 161
Tetrahymena_thermophila_Q22SD0 116 ....GK..................RQIIIASVLIGKAFETSSNASYTKPPVIT..EGK..EQRYDSVKSNNQEGN..NT 166
Tetrahymena_thermophila_Q22SC9 95 ....SK..................KVFFLAQVLVGNYYVGGSSG.YVSPPIIP..GTN..GLRYDSIRSNYNEGQ..NM 144
Tetrahymena_thermophila_Q24C77 115 ....NK..................SQMFLAAVLVGRCIAQPPNA.FVAPPFYN..QAK..GIRYDSVRCMGAYGH..NQ 164
Tetrahymena_thermophila_Q22F17 123 ....GK..................IQLFCAKVLIGKCYATGPNGNLTAPPFIA..GSK..SIRYDSIRSNNAIGG..NE 173
Nematostella_vectensis_A7RWC0 97 ...D.GL.................QYMFVVDALVGDYTEGEPS..YRKPPRKD..SNNPSSDLFDSC.VDDVQHP..EV 147
Homo_sapiens_ZCC2 121 ....KN..................VVMFVAQVLVGKFTEGNIT..YTSPPPQ...........FDSC.VDTRSNP..SV 161
Xenopus_laevis_Q6DDP4 117 ...KHGDTLQNHGIDLNKCSLW.TRKCMFLARVLVGDYAVGNSK..YIRPPSKD..GTL..VNLYDSC.VDDPVCP..RI 183
Homo_sapiens_PAR11 131 ...KHGNTFQIHGVSLQQRHLFRTYKSMFLARVLIGDYINGDSK..YMRPPSKD..GSY..VNLYDSC.VDDTWNP..KI 198
Gallus_gallus_Q5F3B3 120 ....KS..................TVMFMARVLVGDYAQGKEH..YVRPPAKL..VGG..FQFYDSC.VDDVANP..SI 167
Danio_rerio_Q5U400 117 ....GT..................RSMFVCRILVGDYTKGDSS..YLRPPSKD..GGD..TVFYDSC.VNDVCDP..SI 164
Gallus_gallus_XP_416342 118 ....SH..................YSMFVAQVLVGKFVQGDSE..YLRPPPTP..SNR..NRLYDSC.VDDPENP..SI 165
Danio_rerio_XP_695578 117 ...G.KT.................KKMFMALVLVGDFIRGNNS..LVRPPQK...PHS..QRFYDSC.VDNEANP..AI 164
Homo_sapiens_PAR12 130 ....QT..................HTMFLARVLVGEFVRGNAS..FVRPPAKE..GWS..NAFYDSC.VNSVSDP..SI 177
Gallus_gallus_XP_416333 117 ....CS..................KTMFQARVLVGEFTVGRSS..YVRPPLKD....N..QNFYDSC.VNSSSNP..SI 162
Nematostella_vectensis_A7S5J9 129 YSNR.AS.................QQMFVAKVLVGSYTVGSSS..YTRPPAKD..PAKPHGELYDSC.VNDEQNP..GI 182
Branchiostoma_floridae_C3YBY8 142 ...R.GS.................HYIFLANVLVGSHTVGSSK..MRRPPPID..PEDPNGELYDSC.ANSATDP..TI 192
Danio_rerio_NP_001038484 133 ...N.GV.................HYMFLAKVLTGKFTVGNPS..MRRPPPLN..PRDASSDLFDSC.VDNWMDP..QI 183
Homo_sapiens_PARPT 133 ...K.GV.................HFMFLAKVLTGRYTMGSHG..MRRPPPVN..PGSVTSDLYDSC.VDNFFEP..QI 183
Xenopus_tropicalis_A8E4V7 132 ...K.GV.................HYMFLAKVLTGRYVVGNHT..MRRPPPLN..PGSLTSDLYDSC.VDNFFEP..QI 182
Gallus_gallus_XP_422828 133 ...K.GV.................HYMFLAKVLTGRYTVGNHT..MRRPPPVN..PGSITSDLYDSC.VDNYFEP..QI 183
Danio_rerio_XP_001340167 111 ...E.QY.................VYIIQAQVLTGNSAIGSPD..FILPPSLK..GDP..LERYDSL.SD...MG..ET 156
Xenopus_laevis_Q6NRC6 108 ....KL..................VYIFLAEVVLGKTKEAKNS..PFIPLD.G..ADI..FNSYNSL.VDSKHSP..QS 154
Gallus_gallus_XP_422116 120 ...E.SK.................IYVFEADVLTGLYTKGKPS..YIMPPAVE..GDA..SMLYDSL.VDDINNP..DT 168
Homo_sapiens_PARP9 128 ...ADKL.................IYVFEAEVLTGFFCQGHPL..NIVPPPLS..PGA..IDGHDSV.VDNVSSP..ET 177
Homo Sapiens PAR15 Structure
β-sheet
Core β-3
Core β-4
Core β-5

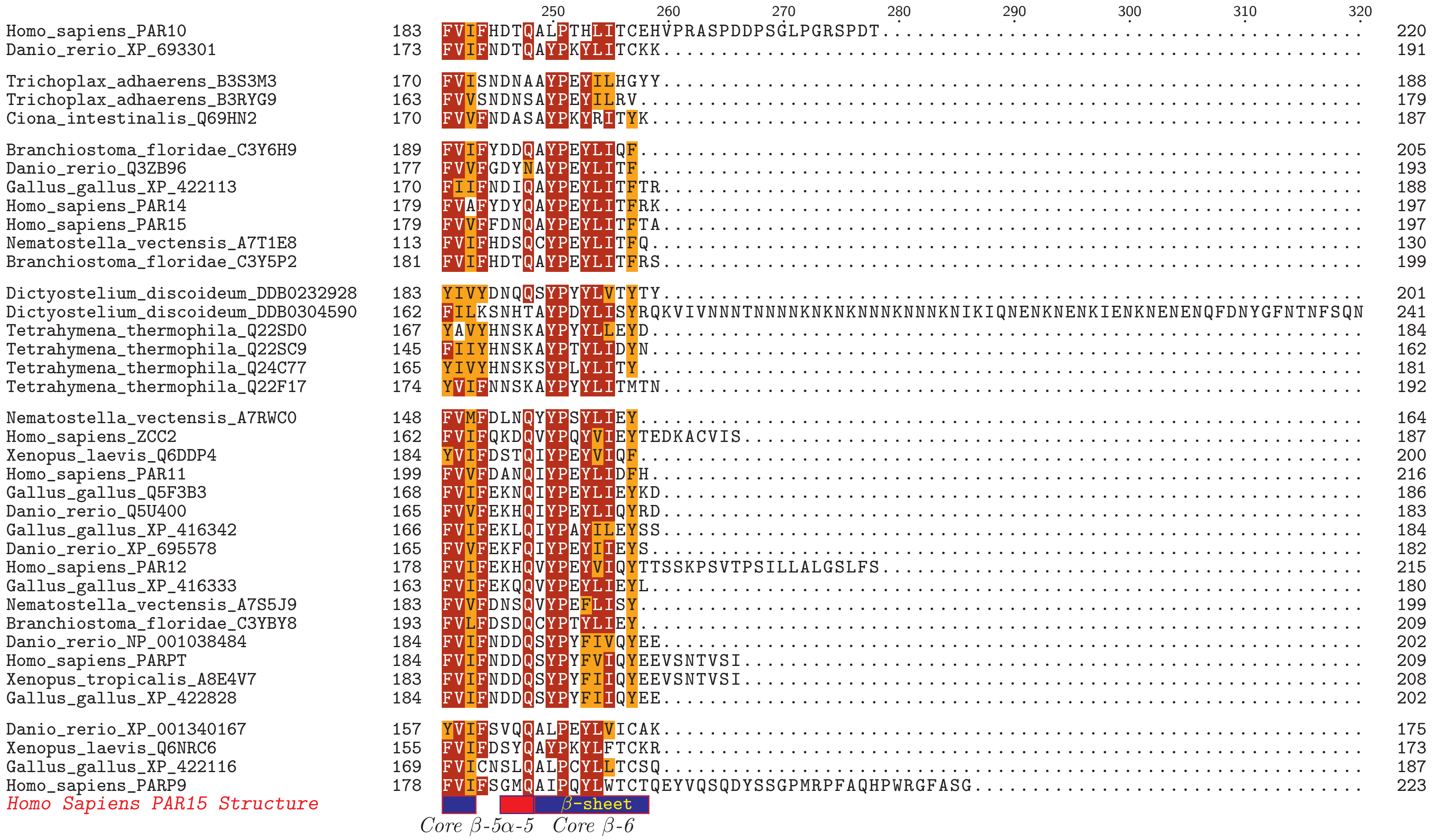

250 260 270 280 290 300 310 320
Homo_sapiens_PAR10 183 FVIFHDTQALPTHLITCEHVPRASPDDPSGLPGRSPDT 220
Danio_rerio_XP_693301 173 FVIFNDTQAYPKYLITCKK 191
Trichoplax_adhaerens_B3S3M3 170 FVISNDNAAYPEYILHGYY 188
Trichoplax_adhaerens_B3RYG9 163 FVVSNDNSAYPEYILRV 179
Ciona_intestinalis_Q69HN2 170 FVVFNDASAYPKYRITYK 187
Branchiostoma_floridae_C3Y6H9 189 FVIFYDDQAYPEYLIQF 205
Danio_rerio_Q3ZB96 177 FVVFGDYNAYPEYLITF 193
Gallus_gallus_XP_422113 170 FIIFNDIQAYPEYLITFTR 188
Homo_sapiens_PAR14 179 FVAFYDYQAYPEYLITFRK 197
Homo_sapiens_PAR15 179 FVVFFDNQAYPEYLITFTA 197
Nematostella_vectensis_A7T1E8 113 FVIFHDSQCYPEYLITFQ 130
Branchiostoma_floridae_C3Y5P2 181 FVIFHDTQAYPEYLITFRS 199
Dictyostelium_discoideum_DDB0232928 183 YIVYDNQQSYPYYLVTYTY 201
Dictyostelium_discoideum_DDB0304590 162 FILKSNHTAYPDYLISYRQKVIVNNNTNNNNKNKNKNNNKNNNKNIKIQNENKNENKIENKNENENQFDNYGFNTNFSQN 241
Tetrahymena_thermophila_Q22SD0 167 YAVYHNSKAYPYYLLEYD 184
Tetrahymena_thermophila_Q22SC9 145 FIIYHNSKAYPTYLIDYN 162
Tetrahymena_thermophila_Q24C77 165 YIVYHNSKSYPLYLITY 181
Tetrahymena_thermophila_Q22F17 174 YVIFNNSKAYPYYLITMTN 192
Nematostella_vectensis_A7RWC0 148 FVMFDLNQYYPSYLIEY 164
Homo_sapiens_ZCC2 162 FVIFQKDQVYPQYVIEYTEDKACVIS 187
Xenopus_laevis_Q6DDP4 184 YVIFDSTQIYPEYVIQF 200
Homo_sapiens_PAR11 199 FVVFDANQIYPEYLIDFH 216
Gallus_gallus_Q5F3B3 168 FVIFEKNQIYPEYLIEYKD 186
Danio_rerio_Q5U400 165 FVVFEKHQIYPEYLIQYRD 183
Gallus_gallus_XP_416342 166 FVIFEKLQIYPAYILEYSS 184
Danio_rerio_XP_695578 165 FVVFEKFQIYPEYIIEYS 182
Homo_sapiens_PAR12 178 FVIFEKHQVYPEYVIQYTTSSKPSVTPSILLALGSLFS 215
Gallus_gallus_XP_416333 163 FVIFEKQQVYPEYLIEYL 180
Nematostella_vectensis_A7S5J9 183 FVVFDNSQVYPEFLISY 199
Branchiostoma_floridae_C3YBY8 193 FVLFDSDQCYPTYLIEY 209
Danio_rerio_NP_001038484 184 FVIFNDDQSYPYFIVQYEE 202
Homo_sapiens_PARPT 184 FVIFNDDQSYPYFVIQYEEVSNTVSI 209
Xenopus_tropicalis_A8E4V7 183 FVIFNDDQSYPYFIIQYEEVSNTVSI 208
Gallus_gallus_XP_422828 184 FVIFNDDQSYPYFIIQYEE 202
Danio_rerio_XP_001340167 157 YVIFSVQQALPEYLVICAK 175
Xenopus_laevis_Q6NRC6 155 FVIFDSYQAYPKYLFTCKR 173
Gallus_gallus_XP_422116 169 FVICNSLQALPCYLLTCSQ 187
Homo_sapiens_PARP9 178 FVIFSGMQAIPQYLWTCTQEYVQSQDYSSGPMRPFAQHPWRGFASG 223
Homo Sapiens PAR15 Structure
β-sheet
Core β-5α-5 Core β-6

Homo_sapiens_PAR10	220	..	220
Danio_rerio_XP_693301	191	..	191
Trichoplax_adhaerens_B3S3M3	188	..	188
Trichoplax_adhaerens_B3RYG9	179	..	179
Ciona_intestinalis_Q69HN2	187	..	187
Branchiostoma_floridae_C3Y6H9	205	..	205
Danio_rerio_Q3ZB96	193	..	193
Gallus_gallus_XP_422113	188	..	188
Homo_sapiens_PAR14	197	..	197
Homo_sapiens_PAR15	197	..	197
Nematostella_vectensis_A7T1E8	130	..	130
Branchiostoma_floridae_C3Y5P2	199	..	199
Dictyostelium_discoideum_DDB0232928	201	..	201
Dictyostelium_discoideum_DDB0304590	242	LY	243
Tetrahymena_thermophila_Q22SD0	184	..	184
Tetrahymena_thermophila_Q22SC9	162	..	162
Tetrahymena_thermophila_Q24C77	181	..	181
Tetrahymena_thermophila_Q22F17	192	..	192
Nematostella_vectensis_A7RWC0	164	..	164
Homo_sapiens_ZCC2	187	..	187
Xenopus_laevis_Q6DDP4	200	..	200
Homo_sapiens_PAR11	216	..	216
Gallus_gallus_Q5F3B3	186	..	186
Danio_rerio_Q5U400	183	..	183
Gallus_gallus_XP_416342	184	..	184
Danio_rerio_XP_695578	182	..	182
Homo_sapiens_PAR12	215	..	215
Gallus_gallus_XP_416333	180	..	180
Nematostella_vectensis_A7S5J9	199	..	199
Branchiostoma_floridae_C3YBY8	209	..	209
Danio_rerio_NP_001038484	202	..	202
Homo_sapiens_PARPT	209	..	209
Xenopus_tropicalis_A8E4V7	208	..	208
Gallus_gallus_XP_422828	202	..	202
Danio_rerio_XP_001340167	175	..	175
Xenopus_laevis_Q6NRC6	173	..	173
Gallus_gallus_XP_422116	187	..	187
Homo_sapiens_PARP9	223	..	223
