# Supplementary material for: Evolutionary history of the poly(ADP-ribose) polymerase gene family in eukaryotes
Source: BMC Evol Biol. 2010 Oct 13;10:308. doi: 10.1186/1471-2148-10-308 (PMC2964712; doi:10.1186/1471-2148-10-308)
Supplement: Additional file 10 — Multiple alignment of the PARP catalytic domain from Clade 5 PARP proteins. These alignments only show the conserved PARP catalytic domain. The structural elements present in Homo sapiens vPARP are shown at the bottom of the alignment. Annotations as in Additional file 5. [file 1471-2148-10-308-S10.PDF]

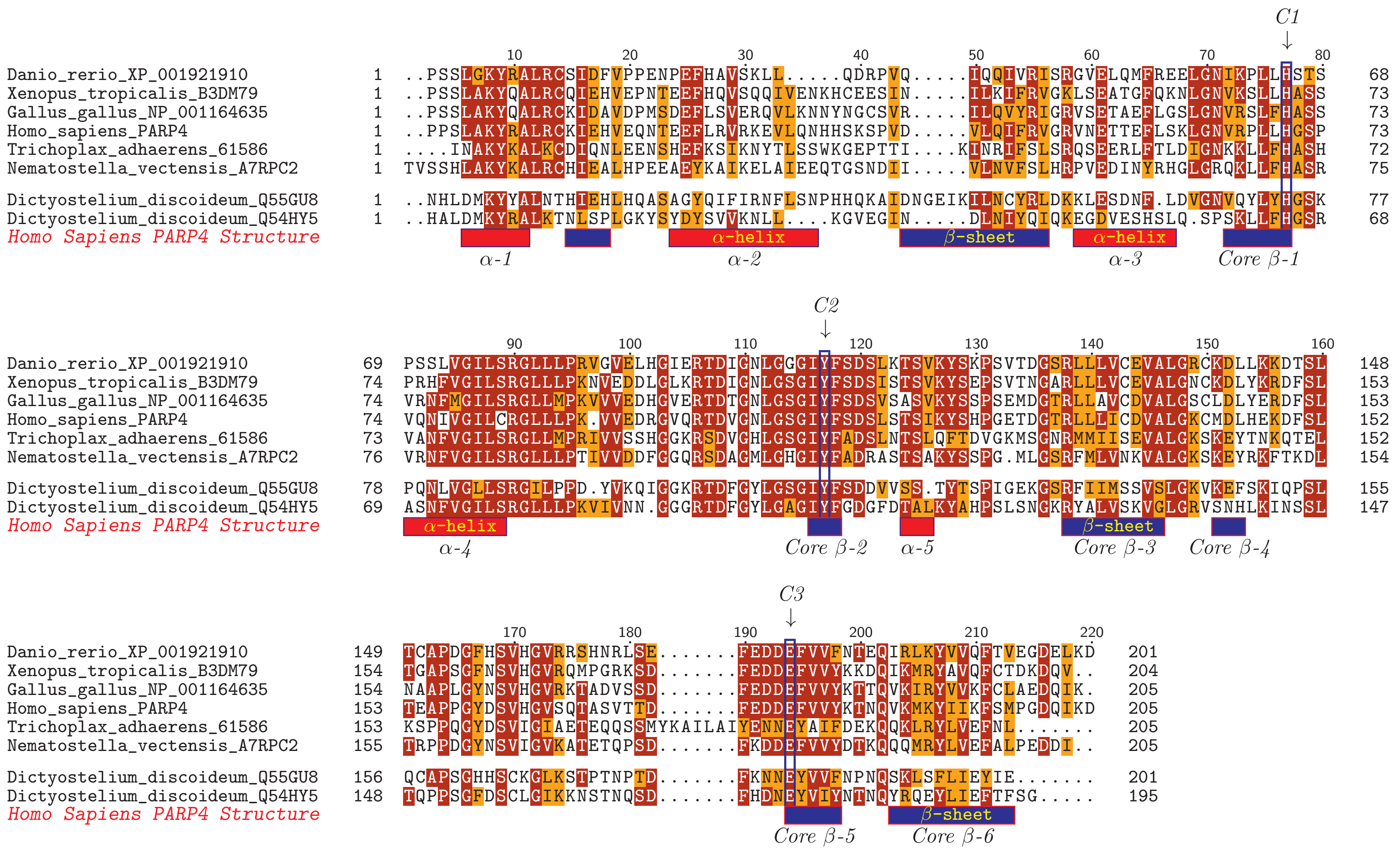
C1
10 20 30 40 50 60 70 80
Danio_rerio_XP_001921910 1 ..PSSLGKYRALRCSIDFVPPENPEFHAVSKLL.....QDRPVQ.....IQQIVRISRGVELQMFREELGNIKPLLHSTS 68
Xenopus_tropicalis_B3DM79 1 ..PSSLAKYQALRCQIEHVEPNTEEFHQVSQQIVENKHCEESIN.....ILKIFRVGKLSEATGFQKNLGNVKSLLHASS 73
Gallus_gallus_NP_001164635 1 ..PSSLAKYQALRCKIDAVDPMSDEFLSVERQVLKNNYNGCSVR.....ILQVYRIGRVSETAEFLGSLGNVRSLFHASS 73
Homo_sapiens_PARP4 1 ..PPSLAKYRALRCKIEHVEQNTEEFLRVRKEVLQNHHSKSPVD.....VLQIFRVGRVNETTEFLSKLGNVRPLLHGSP 73
Trichoplax_adhaerens_61586 1 ....INAKYKALKCDIQNLEENSHEFKSIKNYTLSSWKGEPTTI....KINRIFSLSRQSEERLFTLDIGNKKLLFHASH 72
Nematostella_vectensis_A7RPC2 1 TVSSHLAKYKALRCHIEALHPEEAEYKAIKELAIEEQTGSNDII.....VLNVFSLHRPVEDINYRHGLGRQKLLFHASR 75
Dictyostelium_discoideum_Q55GU8 1 ..NHLDMKYYALNTHIEHLHQASAGYQIFIRNFLSNPHHQKAIDNGEIKILNCYRLDKKLESDNF.LDVGNVQYLYHGSK 77
Dictyostelium_discoideum_Q54HY5 1 ..HALDMKYRALKTNLSPLGKYSYDYSVVKNLL....KGVEGIN.....DLNIYQIQKEGDVESHSLQ.SPSKLLFHGSR 68
Homo Sapiens PARP4 Structure
α-helix β-sheet α-helix
α-1 α-2 α-3 Core β-1
C2
90 100 110 120 130 140 150 160
Danio_rerio_XP_001921910 69 PSSLVGILSRGLLLPRVGVELHGIERTDIGNLGGGIYFSDSLKTSVKYSKPSVTDGSRLLLVCEVALGRCKDLLKKDTSL 148
Xenopus_tropicalis_B3DM79 74 PRHFVGILSRGLLLPKNVEDDLGLKRTDIGNLGSGIYFSDSISTSVKYSEPSVTNGARLLLVCEVALGNCKDLYKRDFSL 153
Gallus_gallus_NP_001164635 74 VRNFMGILSRGLLMPKVVVEDHGVERTDTGNLGSGIYFSDSVSASVKYSSPSEMDGTRLLAVCDVALGSCLDLYERDFSL 153
Homo_sapiens_PARP4 74 VQNIVGILCRGLLLPK.VVEDRGVQRTDVGNLGSGIYFSDSLSTSIKYSHPGETDGTRLLLICDVALGKCMDLHEKDFSL 152
Trichoplax_adhaerens_61586 73 VANFVGILSRGLLMPRIVVSSHGGKRSDVGHLGSGIYFADSLNTSLQFTDVGKMSGNRMMIISEVALGKSKEYTNKQTEL 152
Nematostella_vectensis_A7RPC2 76 VRNFVGILSRGLLLPTIVVDDFGGQRSDAGMLGHGIYFADRASTSAKYSSPG.MLGSRFMLVNKVALGKSKEYRKFTKDL 154
Dictyostelium_discoideum_Q55GU8 78 PQNLVGLLSRGILPPD.YVKQIGGKRTDFGYLGSGIYFSDDVVSS.TYTSPIGEKGSRFIIMSSVSLGKVKEFSKIQPSL 155
Dictyostelium_discoideum_Q54HY5 69 ASNFVGILSRGLLLPKVIVNN.GGGRTDFGYLGAGIYFGDGFDTALKYAHPSLSNGKRYALVSKVGLGRVSNHLKINSSL 147
Homo Sapiens PARP4 Structure
α-helix β-sheet
α-4 Core β-2 α-5 Core β-3 Core β-4
C3
170 180 190 200 210 220
Danio_rerio_XP_001921910 149 TCAPDGFHSVHGVRRSHNRLSE.......FEDDEFVVFNTEQIRLKYVVQFTVEGDELKD 201
Xenopus_tropicalis_B3DM79 154 TGAPSGFNSVHGVRQMPGRKSD.......FEDDEFVVYKKDQIKMRYAVQFCTDKDQV.. 204
Gallus_gallus_NP_001164635 154 NAAPLGYNSVHGVRKTADVSSD.......FEDDEFVVYKTTQVKIRYVVKFCLAEDQIK. 205
Homo_sapiens_PARP4 153 TEAPPGYDSVHGVSQTASVTTD.......FEDDEFVVYKTNQVKMKYIIKFSMPGDQIKD 205
Trichoplax_adhaerens_61586 153 KSPPQGYDSVIGIAETEQQSSMYKAILAIYENNEYAIFDEKQQKLRYLVEFNL....... 205
Nematostella_vectensis_A7RPC2 155 TRPPDGYNSVIGVKATETQPSD.......FKDDEFVVYDTKQQQMRYLVEFALPEDDI.. 205
Dictyostelium_discoideum_Q55GU8 156 QCAPSGHHSCKGLKSTPTNPTD.......FKNNEYVVFNPNQSKLSFLIEYIE....... 201
Dictyostelium_discoideum_Q54HY5 148 TQPPSGFDSCLGIKKNSTNQSD.......FHDNEYVIYNTNQYRQEYLIEFTFSG..... 195
Homo Sapiens PARP4 Structure
β-sheet
Core β-5 Core β-6
