# Supplementary material for: Evolutionary history of the poly(ADP-ribose) polymerase gene family in eukaryotes
Source: BMC Evol Biol. 2010 Oct 13;10:308. doi: 10.1186/1471-2148-10-308 (PMC2964712; doi:10.1186/1471-2148-10-308)
Supplement: Additional file 11 — Multiple alignment of the PARP catalytic domain from Clade 6 PARP proteins annotated with structural information. These alignments only show the conserved PARP catalytic domain. The structural elements predicted to be present in Homo sapiens PARP8 by Phyre are shown at the bottom of the alignment [89]. Annotations as in Additional file 6. [file 1471-2148-10-308-S11.PDF]

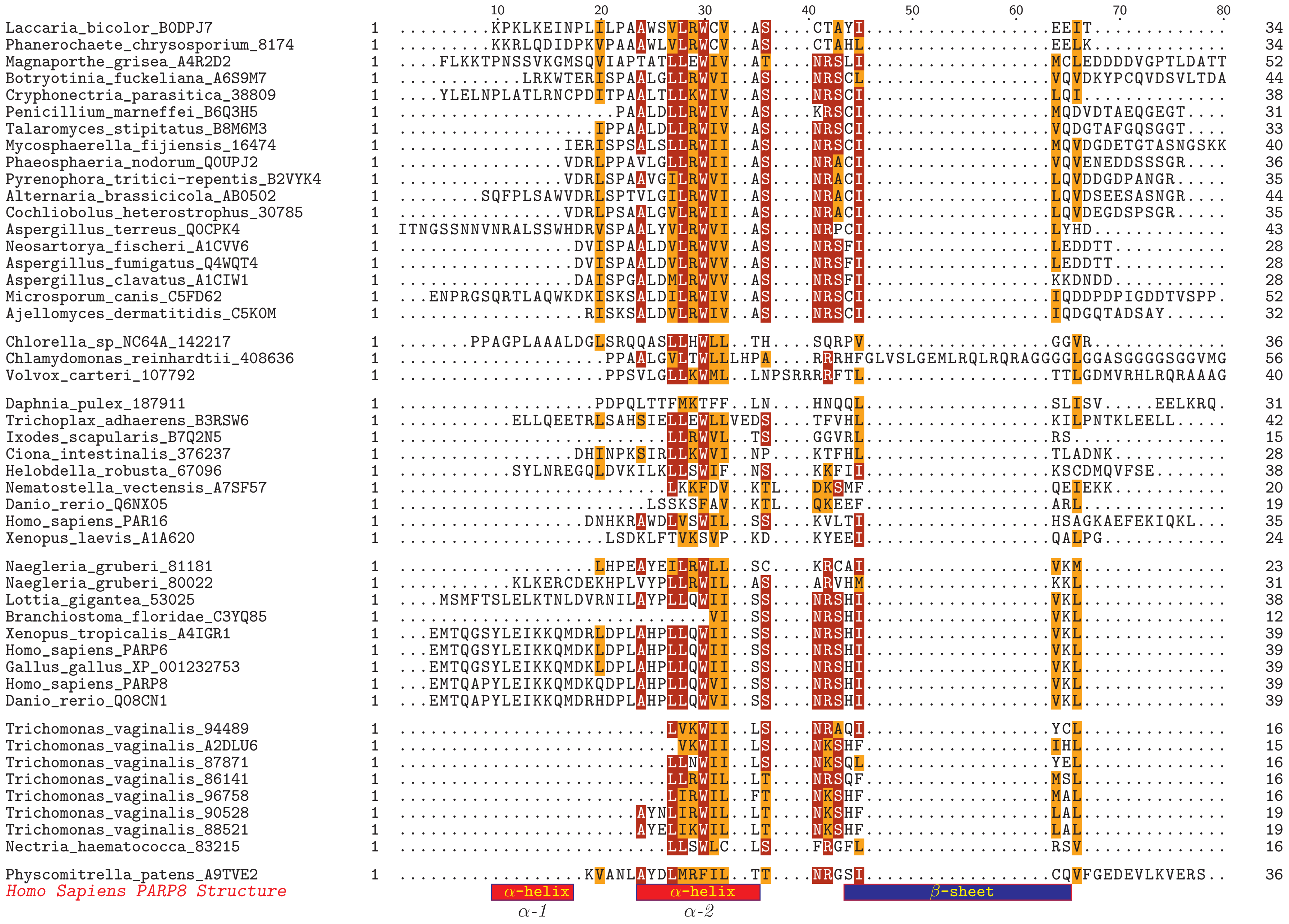

10 20 30 40 50 60 70 80
Laccaria_bicolor_B0DPJ7 1 .........KPKLKEINPLILPAAWSVLRWCV..AS....CTAYI..................EEIT............. 34
Phanerochaete_chrysosporium_8174 1 .........KKRLQDIDPKVPAAAWLVLRWCV..AS....CTAHL..................EELK............. 34
Magnaporthe_grisea_A4R2D2 1 ....FLKKTPNSSVKGMSQVIAPTATLLEWIV..AT....NRSLI..................MCLEDDDDVGPTLDATT 52
Botryotinia_fuckeliana_A6S9M7 1 ............LRKWTERISPAALGLLRWVI..AS....NRSCL..................VQVDKYPCQVDSVLTDA 44
Cryphonectria_parasitica_38809 1 ....YLELNPLATLRNCPDITPAALTLLKWIV..AS....NRSCI..................LQI.............. 38
Penicillium_marneffei_B6Q3H5 1 .....................PAALDLLRWIV..AS....KRSCI..................MQDVDTAEQGEGT.... 31
Talaromyces_stipitatus_B8M6M3 1 ...................IPPAALDLLRWIV..AS....NRSCI..................VQDGTAFGQSGGT.... 33
Mycosphaerella_fijiensis_16474 1 ................IERISPSALSLLRWII..AS....NRSCI..................MQVDGDETGTASNGSKK 40
Phaeosphaeria_nodorum_Q0UPJ2 1 ................VDRLPPAVLGLLRWII..AS....NRACI..................VQVENEDDSSSGR.... 36
Pyrenophora_tritici-repentis_B2VYK4 1 ................VDRLSPAAVGILRWVI..AS....NRACI..................LQVDDGDPANGR..... 35
Alternaria_brassicicola_AB0502 1 ........SQFPLSAWVDRLSPTVLGILRWVI..AS....NRACI..................LQVDSEESASNGR.... 44
Cochliobolus_heterostrophus_30785 1 ................VDRLPSAALGVLRWII..AS....NRACI..................LQVDEGDSPSGR..... 35
Aspergillus_terreus_Q0CPK4 1 ITNGSSNNVNRALSSWHDRVSPAALYVLRWVI..AS....NRPCI..................LYHD............. 43
Neosartorya_fischeri_A1CVV6 1 .................DVISPAALDVLRWVV..AS....NRSFI..................LEDDTT........... 28
Aspergillus_fumigatus_Q4WQT4 1 .................DVISPAALDVLRWVV..AS....NRSFI..................LEDDTT........... 28
Aspergillus_clavatus_A1CIW1 1 .................DAISPGALDMLRWVV..AS....NRSFI..................KKDNDD........... 28
Microsporum_canis_C5FD62 1 ...ENPRGSQRTLAQWKDKISKSALDILRWIV..AS....NRSCI..................IQDDPDPIGDDTVSPP. 52
Ajellomyces_dermatitidis_C5K0M 1 ..................RISKSALDVLRWIV..AS....NRSCI..................IQDGQTADSAY...... 32
Chlorella_sp_NC64A_142217 1 .......PPAGPLAAALDGLSRQQASLLHWLL..TH....SQRPV..................GGVR............. 36
Chlamydomonas_reinhardtii_408636 1 ....................PPAALGVLTWLLLHPA....RRRHFGLVSLGEMLRQLRQRAGGGGLGGASGGGGSGGVMG 56
Volvox_carteri_107792 1 ....................PPSVLGLLKWML..LNPSRRRRFTL..................TTLGDMVRHLRQRAAAG 40
Daphnia_pulex_187911 1 ...................PDPQLTTFMKTFF..LN....HNQQL..................SLISV.....EELKRQ. 31
Trichoplax_adhaerens_B3RSW6 1 ...........ELLQEETRLSAHSIELLEWLLVEDS....TFVHL..................KILPNTKLEELL..... 42
Ixodes_scapularis_B7Q2N5 1 ..........................LLRWVL..TS....GGVRL..................RS............... 15
Ciona_intestinalis_376237 1 .................DHINPKSIRLLKWVI..NP....KTFHL..................TLADNK........... 28
Helobdella_robusta_67096 1 ..........SYLNREGGQLDVKILKLLSWIF..NS....KKFII..................KSCDMQVFSE....... 38
Nematostella_vectensis_A7SF57 1 ..........................LKKFDV..KTL...DKSMF..................QEIEKK........... 20
Danio_rerio_Q6NX05 1 ........................LSSKSFAV..KTL...QKEEF..................ARL.............. 19
Homo_sapiens_PAR16 1 ..................DNHKRAWDLVSWIL..SS....KVLTI..................HSAGKAEFEKIQKL... 35
Xenopus_laevis_A1A620 1 ....................LSDKLFTVKSVP..KD....KYEEI..................QALPG............ 24
Naegleria_gruberi_81181 1 ...................LHPEAYEILRWLL..SC....KRCAI..................VKM.............. 23
Naegleria_gruberi_80022 1 ...........KLKERCDEKHPLVYPLLRWIL..AS....ARVHM..................KKL.............. 31
Lottia_gigantea_53025 1 ....MSMFTSLELKTNLDVRNILAYPLLQWII..SS....NRSHI..................VKL.............. 38
Branchiostoma_floridae_C3YQ85 1 ..............................VI..SS....NRSHI..................VKL.............. 12
Xenopus_tropicalis_A4IGR1 1 ...EMTQGSYLEIKKQMDRLDPLAHPLLQWII..SS....NRSHI..................VKL.............. 39
Homo_sapiens_PARP6 1 ...EMTQGSYLEIKKQMDKLDPLAHPLLQWII..SS....NRSHI..................VKL.............. 39
Gallus_gallus_XP_001232753 1 ...EMTQGSYLEIKKQMDKLDPLAHPLLQWII..SS....NRSHI..................VKL.............. 39
Homo_sapiens_PARP8 1 ...EMTQAPYLEIKKQMDKQDPLAHPLLQWVI..SS....NRSHI..................VKL.............. 39
Danio_rerio_Q08CN1 1 ...EMTQAPYLEIKKQMDRHDPLAHPLLQWVI..SS....NRSHI..................VKL.............. 39
Trichomonas_vaginalis_94489 1 ..........................LVKWII..LS....NRAQI..................YCL.............. 16
Trichomonas_vaginalis_A2DLU6 1 ...........................VKWII..LS....NKSHF..................IHL.............. 15
Trichomonas_vaginalis_87871 1 ..........................LLNWII..LS....NKSQL..................YEL.............. 16
Trichomonas_vaginalis_86141 1 ..........................LLRWIL..LT....NRSQF..................MSL.............. 16
Trichomonas_vaginalis_96758 1 ..........................LIRWIL..FT....NKSHF..................MAL.............. 16
Trichomonas_vaginalis_90528 1 .......................AYNLIRWIL..LT....NKSHF..................LAL.............. 19
Trichomonas_vaginalis_88521 1 .......................AYELIKWIL..LT....NKSHF..................LAL.............. 19
Nectria_haematococca_83215 1 ..........................LLSWLC..LS....FRGFL..................RSV.............. 16
Physcomitrella_patens_A9TVE2 1 ..................KVANLAYDLMRFIL..TT....NRGSI..................CQVFGEDEVLKVERS.. 36
Homo Sapiens PARP8 Structure
α-helix
α-helix
β-sheet
α-1
α-2

```
                                                  90        100       110       120       130       140       150       160
Laccaria_bicolor_B0DPJ7                   35  .........SGQELIKNLD.PNWR.QFRLSVGA.PDAEAK........FKTAIEEAVNSDKHAQKFPVLY..........   84
Phanerochaete_chrysosporium_8174          35  .........DPEDQVTGFG.TGFR.HFRLTVGA.PDAEAK........FYKAQEQAKLEDANAAQYPSIY..........   84
Magnaporthe_grisea_A4R2D2                 53  VQP......LASLKIPEMS.DYIQ..FRFAQGS.PDKEER........FRTELNQMGVV...GSKYPTIF..........  101
Botryotinia_fuckeliana_A6S9M7             45  KI.......RPDQKVSQIGENWMQ..FRFAQGS.PDKEQR........FLNALKEQQANL..DPKYPTLF..........   94
Cryphonectria_parasitica_38809            39  .........RDQEAIPSMRAGHVQ..FRFAQGT.PDKELR........FHRALKEQE.....SHEYPTLF..........   83
Penicillium_marneffei_B6Q3H5              32  .........PCHNLVTGMN.GYLQ..FRFAQGS.PDKEEK........FVSAVSDNCS....NSKHPTIF..........   76
Talaromyces_stipitatus_B8M6M3             34  .........PRHNLVTGMD.GYLQ..FRFAQGS.PDKEEK........FVSAVSKNSF....NSKYPTIF..........   78
Mycosphaerella_fijiensis_16474            41  PVTVFS...KSQERCYGMK.DYMQ..FRFAMGA.PDKEQR........FINEVRATTDRL..GLQYPTIF..........   93
Phaeosphaeria_nodorum_Q0UPJ2              37  .........KAEDALYGMT.GWTQ..FRFAMGA.PDKERR........FISAVRDTSARL..SLKYPTLF..........   83
Pyrenophora_tritici-repentis_B2VYK4       36  .........KSEERLYGMS.GWAQ..FRFAMGA.PDKERR........FIQAVQQTTSRL..GLQYPTLF..........   82
Alternaria_brassicicola_AB0502            45  .........SPEERLYGMA.GWSQ..FRFAMGA.PDKERR........FIQAVKETTERL..SLKYPTLF..........   91
Cochliobolus_heterostrophus_30785         36  .........KAEGRLYGMG.GWAQ..FRFAMGA.PDKERR........FIEAVRQTKERL..HLKYPTIF..........   82
Aspergillus_terreus_Q0CPK4                44  .........DPEHMVSGMS.GYLQ..FRFAQGA.PDKEAR........FVQAVNAVSSTKDGNSQHPTLF..........   92
Neosartorya_fischeri_A1CVV6               29  .........HSDYRVTGMG.SYKQ..FRLVQGA.PDKEQR........FRAAVAANAAMT..KTDYPTIF..........   75
Aspergillus_fumigatus_Q4WQT4              29  .........HSDHRVSGMG.SYKQ..FRLVQGA.PDKEQR........FRAAVAANVAMT..KTDYPTIF..........   75
Aspergillus_clavatus_A1CIW1               29  .........NLEHRVSGMD.SYIQ..FRLVQGA.PDKEQR........FINAVNAKAA....KSNHPTLF..........   73
Microsporum_canis_C5FD62                  53  .........ANNDRVRGVA.GYMQ..FRFAQGA.PDKEER........FSQAVMAAKERL..QKPHATLF..........   99
Ajellomyces_dermatitidis_C5K0M            33  .........NDSDRVSGME.NYMQ..FRFAQGA.PDKEQR........FVQAVAAVTERL..NLKYPTIF..........   79

Chlorella_sp_NC64A_142217                 37  .........RCTLRAVQEQ.MPLLTGWMVDVGRNPGLRPHAVLQLSELPRDLV.........DGSGQRVL..........   87
Chlamydomonas_reinhardtii_408636          57  GVAAAGGGGPMVWQVPAFA.GHNSPTFVLRAHD....SHT........QEPF...........AHGAV...........  100
Volvox_carteri_107792                     41  GGGGSSSGSDGSWQLPSLS.GPNSPAYILRVHHHNLPQDQ........QQGFSISTTAAAATANGTGSSFQCDGSGGGVI  111

Daphnia_pulex_187911                      32  .........VGGQIELQIA.PRWI..FQIDHVS..RSQQT........WERR..........KASSSSFY..........   69
Trichoplax_adhaerens_B3RSW6               43  .........TATGFRNGTR.PSLI..FDIIYKG.KSSSSR........FDEL..........KSQYGSFY..........   81
Ixodes_scapularis_B7Q2N5                  16  .........RSKHDVPELR.GSHV.DLVFEVEHSAQTERR........FASA..........RGERDVFF..........   56
Ciona_intestinalis_376237                 29  .........EFTELTADID.PPHAPDYLFKVVYNDKKSEK........FQQL..........AEEHSLMY..........   70
Helobdella_robusta_67096                  39  .........IREKACYTFETSPPT..YIFSVEHSALSNER........FETL..........RNNRNVFY..........   79
Nematostella_vectensis_A7SF57             21  .........TGHSSYNSTE.PDYI..FEIQYHENNALNAR........FLAL..........SEECEVLY..........   60
Danio_rerio_Q6NX05                        20  .........SQLAQREGVSAPAPD..FLFELQYCDLLNSK........FERT..........RAGRDLIY..........   60
Homo_sapiens_PAR16                        36  .........TGAPHTPVPA.PDFL..FEIEYFD.PANAK.........FYET..........KGERDLIY..........   73
Xenopus_laevis_A1A620                     25  .........SPSHALPTPD.......FLFELEYCEKLSAK........FQET..........RGDRDLIY..........   60

Naegleria_gruberi_81181                   24  .........PEKKRIQEMQ.TQYQ..YIMMMDN.PEKAAT........FSQN..........RKKYGSYW..........   62
Naegleria_gruberi_80022                   32  .........VGKEQLSEMK.TDHQ..YILLSST.PDKERR........FQEL..........KKKYGSIL..........   70
Lottia_gigantea_53025                     39  .........SPNKIISSMA.TPHQ..FLLRSSP.PAKEAK........FCDL..........RKQYGSTF..........   77
Branchiostoma_floridae_C3YQ85             13  .........PESRRIKFMH.TPHQ..FLLLSSP.PAKEQA........FREA..........KRQHGSLF..........   51
Xenopus_tropicalis_A4IGR1                 40  .........PLSRQLKFMH.TSHQ..FLLLSSP.PAKEAR........FRTA..........KKLYGSTF..........   78
Homo_sapiens_PARP6                        40  .........PLS.RLKFMH.TSHQ..FLLLSSP.PAKEAR........FRTA..........KKLYGSTF..........   77
Gallus_gallus_XP_001232753                40  .........PLSRQLKFMH.TSHQ..FLLLSSP.PAKEAR........FRTA..........KKLYGSTF..........   78
Homo_sapiens_PARP8                        40  .........PVNRQLKFMH.TPHQ..FLLLSSP.PAKESN........FRAA..........KKLFGSTF..........   78
Danio_rerio_Q08CN1                        40  .........TVTRQLKFMH.TPHQ..FLLLSSP.PAKESN........FRAA..........KGLFGSTF..........   78

Trichomonas_vaginalis_94489               17  .........PNTLKPSIFN.KDCI.MFMTFLSS.PQKDEN........FNKL..........KSSYGSTF..........   56
Trichomonas_vaginalis_A2DLU6              16  .........PQQMMLQQIR.SPDH..FLAIISS.PEREYR........FRAL..........KARYGSMF..........   54
Trichomonas_vaginalis_87871               17  .........SPELRLKQFP.TRHQ..FLTLMAS.EERENE........FKAL..........KAKYGSFF..........   55
Trichomonas_vaginalis_86141               17  .........NGSLELAELK.GGTAKMFLALSST.PQKEQR........FREL..........QQQYGSLF..........   57
Trichomonas_vaginalis_96758               17  .........PPELRIKEIN.CKYQ..FLTLISN.FEKEAI........FKEY..........KKKFGSFY..........   55
Trichomonas_vaginalis_90528               20  .........PEGLRIREIN.AQFQ..FLTLISS.TEREEE........FKKL..........KDQYGSFY..........   58
Trichomonas_vaginalis_88521               20  .........PNELKINEIN.ARFQ..FLTLISS.VQKEEE........FKKL..........KQKYGSIY..........   58
Nectria_haematococca_83215                17  .........PSGFAIPSMP.QTQQ..FLLMNSH.HEREKA........FETHLGA.......TASAGSGP..........   58

Physcomitrella_patens_A9TVE2              37  .........PKSKDAPGES.GIYQ..FVVLHDS.PERAAD........FDHRR.........RDAGGSYF..........   76
```

Homo Sapiens PARP8 Structure: β-sheet | α-helix (α-3) | β-sheet (Core β-1)

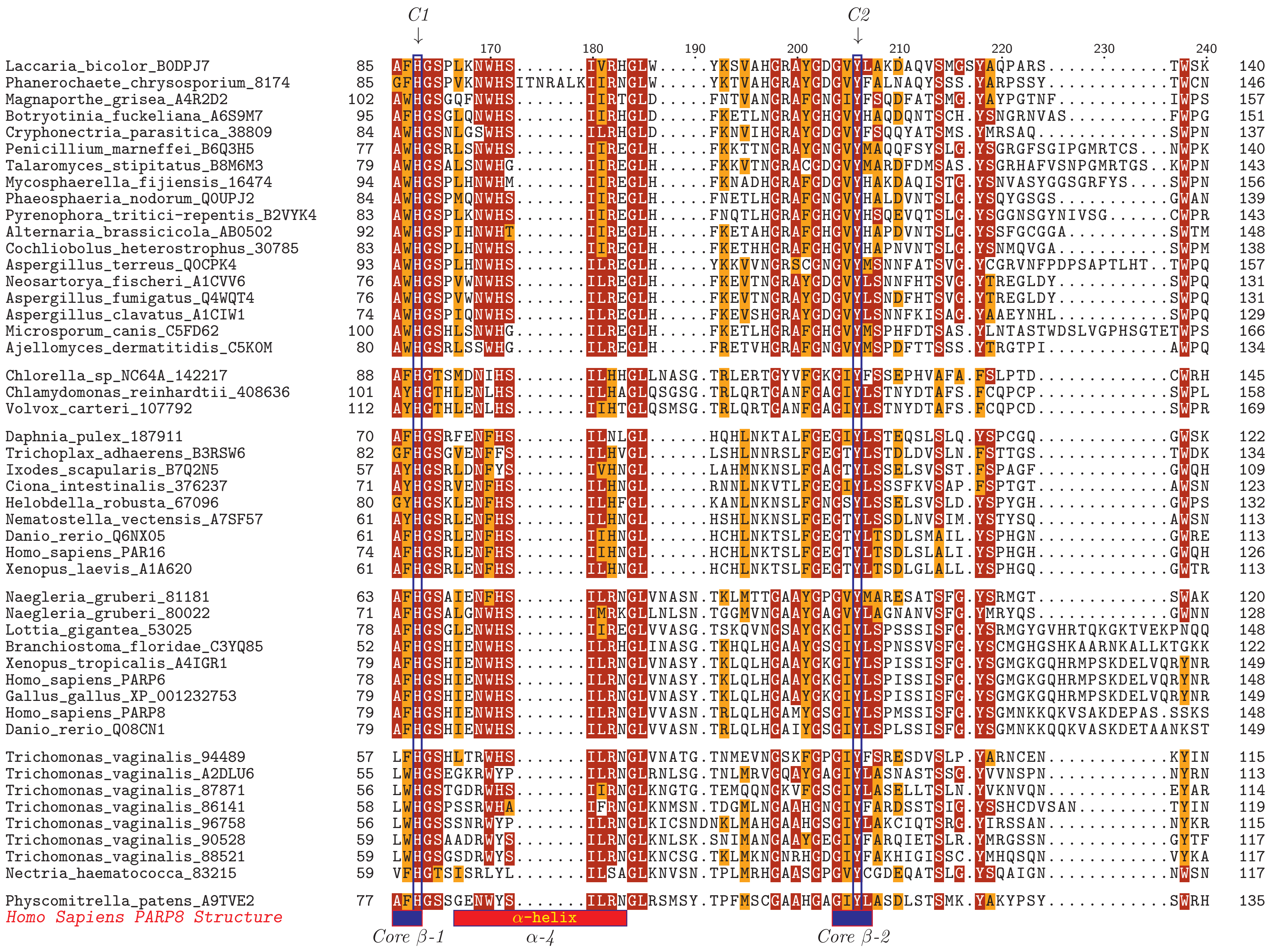
C1
C2
170 180 190 200 210 220 230 240
Laccaria_bicolor_B0DPJ7 85 AFHGSPLKNWHS.......IVRHGLW.....YKSVAHGRAYGDGVYLAKDAQVSMGSYAQPARS...........TWSK 140
Phanerochaete_chrysosporium_8174 85 GFHGSPVKNWHSITNRALKIIRNGLW.....YKTVAHGRAYGDGVYFALNAQYSSS.YARPSSY...........TWCN 146
Magnaporthe_grisea_A4R2D2 102 AWHGSGQFNWHS.......IIRTGLD.....FNTVANGRAFGNGIYFSQDFATSMG.YAYPGTNF..........IWPS 157
Botryotinia_fuckeliana_A6S9M7 95 AFHGSGLQNWHS.......IIRHGLD.....FKETLNGRAYGHGVYHAQDQNTSCH.YSNGRNVAS.........FWPG 151
Cryphonectria_parasitica_38809 84 AWHGSNLGSWHS.......ILRHGLD.....FKNVIHGRAYGDGVYFSQQYATSMS.YMRSAQ............SWPN 137
Penicillium_marneffei_B6Q3H5 77 AWHGSRLSNWHS.......IIREGLH.....FKKTTNGRAYGNGVYMAQQFSYSLG.YSGRGFSGIPGMRTCS...NWPK 140
Talaromyces_stipitatus_B8M6M3 79 AWHGSALSNWHG.......IIREGLH.....FKKVTNGRACGDGVYMARDFDMSAS.YSGRHAFVSNPGMRTGS..KWPN 143
Mycosphaerella_fijiensis_16474 94 AWHGSPLHNWHM.......IIREGLH.....FKNADHGRAFGDGVYHAKDAQISTG.YSNVASYGGSGRFYS....SWPN 156
Phaeosphaeria_nodorum_Q0UPJ2 84 AWHGSPMQNWHS.......IIREGLH.....FNETLHGRAFGNGVYHALDVNTSLG.YSQYGSGS..........GWAN 139
Pyrenophora_tritici-repentis_B2VYK4 83 AWHGSPLKNWHS.......IIREGLH.....FNQTLHGRAFGHGVYHSQEVQTSLG.YSGGNSGYNIVSG.....CWPR 143
Alternaria_brassicicola_AB0502 92 AWHGSPIHNWHT.......IIREGLH.....FKETAHGRAFGHGVYHAPDVNTSLG.YSSFGCGGA.........SWTM 148
Cochliobolus_heterostrophus_30785 83 AWHGSPLHNWHS.......IIREGLH.....FKETHHGRAFGHGVYHAPNVNTSLG.YSNMQVGA..........SWPM 138
Aspergillus_terreus_Q0CPK4 93 AWHGSPLHNWHS.......ILREGLH.....YKKVVNGRSCGNGVYMSNNFATSVG.YCGRVNFPDPSAPTLHT..TWPQ 157
Neosartorya_fischeri_A1CVV6 76 AWHGSPVWNWHS.......ILREGLH.....FKEVTNGRAYGDGVYLSNNFHTSVG.YTREGLDY..........SWPQ 131
Aspergillus_fumigatus_Q4WQT4 76 AWHGSPVWNWHS.......ILREGLH.....FKEVTNGRAYGDGVYLSNDFHTSVG.YTREGLDY..........SWPQ 131
Aspergillus_clavatus_A1CIW1 74 AWHGSPIQNWHS.......ILREGLH.....FKEVSHGRAYGDGVYLSNNFKISAG.YAAEYNHL..........SWPQ 129
Microsporum_canis_C5FD62 100 AWHGSHLSNWHG.......ILREGLH.....FKETLHGRAFGHGVYMSPHFDTSAS.YLNTASTWDSLVGPHSGTETWPS 166
Ajellomyces_dermatitidis_C5K0M 80 AWHGSRLSSWHG.......ILREGLH.....FRETVHGRAFGNGVYMSPDFTTSSS.YTRGTPI...........AWPQ 134
Chlorella_sp_NC64A_142217 88 AFHGTSMDNIHS.......ILHHGLLNASG.TRLERTGYVFGKGIYFSSEPHVAFA.FSLPTD............CWRH 145
Chlamydomonas_reinhardtii_408636 101 AYHGTHLENLHS.......ILHAGLQSGSG.TRLQRTGANFGAGIYLSTNYDTAFS.FCQPCP............SWPL 158
Volvox_carteri_107792 112 AYHGTHLENLHS.......IIHTGLQSMSG.TRLQRTGANFGAGIYLSTNYDTAFS.FCQPCD............SWPR 169
Daphnia_pulex_187911 70 AFHGSRFENFHS.......ILNLGL......HQHLNKTALFGEGIYLSTEQSLSLQ.YSPCGQ............GWSK 122
Trichoplax_adhaerens_B3RSW6 82 GFHGSGVENFFS.......ILHVGL......LSHLNNRSLFGEGTYLSTDLDVSLN.FSTTGS............TWDK 134
Ixodes_scapularis_B7Q2N5 57 AYHGSRLDNFYS.......IVHNGL......LAHMNKNSLFGAGTYLSSELSVSST.FSPAGF............GWQH 109
Ciona_intestinalis_376237 71 AYHGSRVENFHS.......ILHNGL......RNNLNKVTLFGEGIYLSSSFKVSAP.FSPTGT............AWSN 123
Helobdella_robusta_67096 80 GYHGSKLENFHS.......ILHFGL......KANLNKNSLFGNGSYLSSELSVSLD.YSPYGH............GWPS 132
Nematostella_vectensis_A7SF57 61 AYHGSRLENFHS.......ILHNGL......HSHLNKNSLFGEGTYLSSDLNVSIM.YSTYSQ............AWSN 113
Danio_rerio_Q6NX05 61 AFHGSRLENFHS.......IIHNGL......HCHLNKTSLFGEGTYLTSDLSMAIL.YSPHGN............GWRE 113
Homo_sapiens_PAR16 74 AFHGSRLENFHS.......IIHNGL......HCHLNKTSLFGEGTYLTSDLSLALI.YSPHGH............GWQH 126
Xenopus_laevis_A1A620 61 AFHGSRLENFHS.......ILHNGL......HCHLNKTSLFGEGTYLTSDLGLALL.YSPHGQ............GWTR 113
Naegleria_gruberi_81181 63 AFHGSAIENFHS.......ILRNGLVNASN.TKLMTTGAAYGPGVYMARESATSFG.YSRMGT............SWAK 120
Naegleria_gruberi_80022 71 AFHGSALGNWHS.......IMRKGLLNLSN.TGGMVNGAAYGAGVYLAGNANVSFG.YMRYQS............GWNN 128
Lottia_gigantea_53025 78 AFHGSGLENWHS.......IIREGLVVASG.TSKQVNGSAYGKGIYLSPSSSISFG.YSRMGYGVHRTQKGKTVEKPNQQ 148
Branchiostoma_floridae_C3YQ85 52 AFHGSHIENWHS.......ILRHGLINASG.TKHQLHGAAYGSGIYLSPNSSVSFG.YSCMGHGSHKAARNKALLKTGKK 122
Xenopus_tropicalis_A4IGR1 79 AFHGSHIENWHS.......ILRNGLVNASY.TKLQLHGAAYGKGIYLSPISSISFG.YSGMGKGQHRMPSKDELVQRYNR 149
Homo_sapiens_PARP6 78 AFHGSHIENWHS.......ILRNGLVNASY.TKLQLHGAAYGKGIYLSPISSISFG.YSGMGKGQHRMPSKDELVQRYNR 148
Gallus_gallus_XP_001232753 79 AFHGSHIENWHS.......ILRNGLVNASY.TKLQLHGAAYGKGIYLSPISSISFG.YSGMGKGQHRMPSKDELVQRYNR 149
Homo_sapiens_PARP8 79 AFHGSHIENWHS.......ILRNGLVVASN.TRLQLHGAMYGSGIYLSPMSSISFG.YSGMNKKQKVSAKDEPAS.SSKS 148
Danio_rerio_Q08CN1 79 AFHGSHIENWHS.......ILRNGLVVASN.TRLQLHGAIYGSGIYLSPLSSISFG.YSGMNKKQQKVASKDETAANKST 149
Trichomonas_vaginalis_94489 57 LFHGSHLTRWHS.......ILRNGLVNATG.TNMEVNGSKFGPGIYFSRESDVSLP.YARNCEN...........KYIN 115
Trichomonas_vaginalis_A2DLU6 55 LWHGSEGKRWYP.......ILRNGLRNLSG.TNLMRVGQAYGAGIYLASNASTSSG.YVVNSPN...........NYRN 113
Trichomonas_vaginalis_87871 56 LWHGSTGDRWHS.......IIRNGLKNGTG.TEMQQNGKVFGSGIYLASELLTSLN.YVKNVQN...........EYAR 114
Trichomonas_vaginalis_86141 58 LWHGSPSSRWHA.......IFRNGLKNMSN.TDGMLNGAAHGNGIYFARDSSTSIG.YSSHCDVSAN........TYIN 119
Trichomonas_vaginalis_96758 56 LWHGSSSNRWYP.......ILRNGLKICSNDNKLMAHGAAHGSGIYLAKCIQTSRG.YIRSSAN...........NYKR 115
Trichomonas_vaginalis_90528 59 LWHGSAADRWYS.......ILRNGLKNLSK.SNIMANGAAYGEGIYFARQIETSLR.YMRGSSN...........GYTF 117
Trichomonas_vaginalis_88521 59 LWHGSGSDRWYS.......ILRNGLKNCSG.TKLMKNGNRHGDGIYFAKHIGISSC.YMHQSQN...........VYKA 117
Nectria_haematococca_83215 59 VFHGTSISRLYL.......ILSAGLKNVSN.TPLMRHGAASGPGVYCGDEQATSLG.YSQAIGN...........NWSN 117
Physcomitrella_patens_A9TVE2 77 AFHGSSGENWYS.......ILRNGLRSMSY.TPFMSCGAAHGAGIYLASDLSTSMK.YAKYPSY...........SWRH 135
Homo Sapiens PARP8 Structure
α-helix
Core β-1
α-4
Core β-2

```
                                                           250       260       270       280       290       300       310       320
                                                             .         .         .         .         .         .         .         .
Laccaria_bicolor_B0DPJ7                         141   SQTSP...............TNCVALAEVVN.LPAKF...................................VSSNP...   166
Phanerochaete_chrysosporium_8174                147   SDIRV...............RNCVALAEIVN.LPHHFRHGGGNRRT..........................NGRTP.TA   183
Magnaporthe_grisea_A4R2D2                       158   SAFQS...............NITIVLSELIN.IPNRF...................................VSRSP...   183
Botryotinia_fuckeliana_A6S9M7                   152   SQLKA...............TAVMSINEIIN.CPKQF...................................VSSSP...   177
Cryphonectria_parasitica_38809                  138   SALCA...............TGAIALCEIIN.RPDQF...................................VNTIP...   163
Penicillium_marneffei_B6Q3H5                    141   SVLDM...............NVAVSLNEVVN.RPDDFV..................................HGSRG...   167
Talaromyces_stipitatus_B8M6M3                   144   SLLNM...............TCAIALNEVVN.KPSEF...................................VHHAN...   169
Mycosphaerella_fijiensis_16474                  157   SSLRI...............SSALALNEIVN.APAEF...................................TSSNP...   182
Phaeosphaeria_nodorum_Q0UPJ2                    140   SELKM...............SQALALNEIVN.APSEF...................................TSKTP...   165
Pyrenophora_tritici-repentis_B2VYK4             144   SELKV...............QQALALNEIVN.ATSEY...................................VSHSP...   169
Alternaria_brassicicola_AB0502                  149   SELKV...............QQTLALNEIVN.APIEF...................................VSQTP...   174
Cochliobolus_heterostrophus_30785               139   SELKI...............QQALALNEIVN.APGEY...................................VSQNP...   164
Aspergillus_terreus_Q0CPK4                      158   SKLNI...............KCAISLNEVVN.APNQF...................................VHNTA...   183
Neosartorya_fischeri_A1CVV6                     132   SNLKI...............RMLVSLNELVN.APAQF...................................KHTNP...   157
Aspergillus_fumigatus_Q4WQT4                    132   SNLKI...............RMLVSLNELVN.APAQF...................................KHTNP...   157
Aspergillus_clavatus_A1CIW1                     130   SRLGI...............QACISLNEVVN.VPAEF...................................KSRSP...   155
Microsporum_canis_C5FD62                        167   SKLGV..............ISAISLNEVVN.SPRDF...................................VSTTP...   192
Ajellomyces_dermatitidis_C5K0M                  135   SQLKI...............QSAISLNEVVN.APNEF...................................VSKSP...   160

Chlorella_sp_NC64A_142217                       146   SALGQ..............RLRCLLACSVQH.EHAL....................................GTHNT..D   172
Chlamydomonas_reinhardtii_408636                159   SRFGT..............KLRVLLVCEV...DLDL....................................CSSGPLIP   185
Volvox_carteri_107792                           170   SRFGS..............KLRALLVCEVDR.DKYTLGRG................................PGSGP..S   200

Daphnia_pulex_187911                            123   SQLGP..............ELSLLAICEVID.HPDVKRRGSR..............................PSTGN..L   155
Trichoplax_adhaerens_B3RSW6                     135   SMIAK..............SISCVAVCEVIK.HPRIL...................................ANQNK...   161
Ixodes_scapularis_B7Q2N5                        110   SVISS..............EFSLVAMCELID.HPDVKCQDRSKSH.....................PSRAYAQDSIG..G   151
Ciona_intestinalis_376237                       124   SFFGD..............SISCVALCELVN.HPDVKCSVEAVKEDSKDSNLRNRSSDPCSSQKSSKHRSYIKGSEG..G   186
Helobdella_robusta_67096                        133   SCMGS..............RLSCVAVCEIID.DPSVKCKTQTE.....D.................KSRSHIDGSIG..G   173
Nematostella_vectensis_A7SF57                   114   SGLGE..............KLSCVAVCEVLN.HPDVKCSLEDPRAAT.S.................SVRARAKGSEG..G   158
Danio_rerio_Q6NX05                              114   SVLGP..............FISCVALCEIID.HPDVKCQVKKKDSESID.................RQRLRARNSEG..G   159
Homo_sapiens_PAR16                              127   SLLGP..............ILSCVAVCEVID.HPDVKCQTKKKDSKEID.................RRRARIKHSEG..G   172
Xenopus_laevis_A1A620                           114   SMLGP..............VLSCVAMCEMID.HPDVKCQAKKKDSSEID.................RKRARARNSEG..G   159

Naegleria_gruberi_81181                         121   SQFGN............VSNLRCVMICEVNK.APGVP...................................TTASP...   149
Naegleria_gruberi_80022                         129   SITFK............SNQIGCLALCEILK.APELI...................................QKYGD..K   158
Lottia_gigantea_53025                           149   PCYTIYNVFQKSTRFLNSNIINCIAICEVIT.SPEL....................................KKNND...   188
Branchiostoma_floridae_C3YQ85                   123   ENTKR.........FLESDNLTCIALCEVIH.SKEL....................................RKHNN...   153
Xenopus_tropicalis_A4IGR1                       150   MNTIPQTRCLQS.RFLQSRNLNCIALCEVIT.SKDL....................................QKHGN...   188
Homo_sapiens_PARP6                              149   MNTIPQTRSIQS.RFLQSRNLNCIALCEVIT.SKDL....................................QKHGN...   187
Gallus_gallus_XP_001232753                      150   MNTIPQTRSIQS.RFLQSRNLNCIALCEVIT.SKDL....................................QKHGN...   188
Homo_sapiens_PARP8                              149   SNTSQSQKKGQQSQFLQSRNLKCIALCEVIT.SSDL....................................HKHGE...   188
Danio_rerio_Q08CN1                              150   VHLQSQKKGQNP.QFLQSRNLKCIALCEVIT.SPDL....................................HKHGD...   188

Trichomonas_vaginalis_94489                     116   SALGR..............VISLMSLCEVAK.TPDL....................................KDQGR...   141
Trichomonas_vaginalis_A2DLU6                    114   SSFGQ..............NLTLIALCEVAK.VPAL....................................KDHGN...   139
Trichomonas_vaginalis_87871                     115   STLGN..............SLQAVGLCEVAK.VPDL....................................EDHGR...   140
Trichomonas_vaginalis_86141                     120   SRFKI...............MTIVALCEIAK.VPAL....................................KDHGW...   144
Trichomonas_vaginalis_96758                     116   SMYGD...............LNMIALCEVIKDFKHL....................................TEVQD...   141
Trichomonas_vaginalis_90528                     118   SSLDR..............QFNMIALCEVIKDFSNL....................................KEHDN...   144
Trichomonas_vaginalis_88521                     118   SKLSV............NKNFNMIALCEVINDEKYL....................................TEHSN...   146
Nectria_haematococca_83215                      118   SAIDQ..............QMRVMLGCELAK.YP......................................TANTP...   141

Physcomitrella_patens_A9TVE2                    136   GMLKH..............GFQCVAICEVVKGSISRQK..................................IISTQ..F   165
```

Homo Sapiens PARP8 Structure — β-sheet

Core β-3

```
                                        C3
                                        ↓
                                                  330       340       350       360       370       380       390       400
Laccaria_bicolor_B0DPJ7                 167  .....YLVVKDTHWIMCRYLLLKGLNDSDGLT.......................IAPEPKSKHKAKKDQIPVVKLDPTH  218
Phanerochaete_chrysosporium_8174        184  AQPANILVVPQTEWILCRYLIVAGISQNGSEP.....................VAPNSTSPVPRVTLDPRYQIKI.....  237
Magnaporthe_grisea_A4R2D2               184  .....HLVVQNIDWQQCRYLFIKKQMPSEQDP.....................PLSAVAPPASLRKPSPIMFIDQDP...  234
Botryotinia_fuckeliana_A6S9M7           178  .....FLVVQHIDWIQCRYL............................................................  192
Cryphonectria_parasitica_38809          164  .....HLVVSQVDWIQCRYL............................................................  178
Penicillium_marneffei_B6Q3H5            168  .....IYVVNRIEWIQTRYLFVGRHTPAGNFN................................................  194
Talaromyces_stipitatus_B8M6M3           170  ....GVYVVDRIEWIQTRYLFVDCQKSPVNQN.......................LVGP.....................  201
Mycosphaerella_fijiensis_16474          183  .....YYVVKQLDWIQTRYL............................................................  197
Phaeosphaeria_nodorum_Q0UPJ2            166  .....HLVVPQLDWIQTRYL............................................................  180
Pyrenophora_tritici-repentis_B2VYK4     170  .....HLVVAQLDWIQTRYLFV..........................................................  186
Alternaria_brassicicola_AB0502          175  .....HLVVAQLDWIQTRYLFV..........................................................  191
Cochliobolus_heterostrophus_30785       165  .....HLVVAQLDWIQTRYLFVRKRADVSSTAAPNP............................................  195
Aspergillus_terreus_Q0CPK4              183  .....CYVVQHLDWIQPRYLFVGPTPTDAK.........................VCQGNPSRIYTQDPK..........  223
Neosartorya_fischeri_A1CVV6             158  .....HYVVTQLDWIQPRYLFVKCQGAGPGDG.......................TANAKPSAMYTQDPK..........  199
Aspergillus_fumigatus_Q4WQT4            158  .....HYVVTQLDWIQPRYLFVKCQGAGLGDG.......................TANAKPSAIYNQDPK..........  199
Aspergillus_clavatus_A1CIW1             156  .....HYVVPQLDWIQPRYLF...........................................................  171
Microsporum_canis_C5FD62                193  .....HLVVSQLDWIQTRYLFV..........................................................  209
Ajellomyces_dermatitidis_C5K0M          161  .....HLVVAQLDWIQPRYLFVSCQSLDLCNK........................DRVEQGSSSACHLQD..........  202

Chlorella_sp_NC64A_142217               173  LVPDKYLLVERMDAVQIHFLFVYVD.......................................................  197
Chlamydomonas_reinhardtii_408636        186  GLPDTYLLVQRSDAVRILFVAIYTDAAAPPAGAAGAGAGAAGAGGGPGARRAGPLARVNWCTVMVVLYAGFLVGKALLAA  265
Volvox_carteri_107792                   201  QVPETYLVVQRADAVRLLYIMLYCDAPRTARP.......................ARVNWCTVMVVLYAAFLLGKALLAA  257

Daphnia_pulex_187911                    156  AIPDKYILVTNNELVRPRYFLVYTRPKGK...................................................  184
Trichoplax_adhaerens_B3RSW6             162  ................................................................................  161
Ixodes_scapularis_B7Q2N5                152  KVPEKYFLVQNNSVVRVRYLLVYTAVHPRSPS................................................  183
Ciona_intestinalis_376237               187  QIPDKYFVVRNDELVRVKYIALF.........................................................  209
Helobdella_robusta_67096                174  EIPEKYYIVTDDEFVRVKYLLVYASRDKYLKL.......................VN.......................  207
Nematostella_vectensis_A7SF57           159  DVPDRYYVVQNNQLLRVKYLLVYAE.......................................................  183
Danio_rerio_Q6NX05                      160  EVPEKYFVVTNNELLRVKYLLVYSQ.......................................................  184
Homo_sapiens_PAR16                      173  DIPPKYFVVTNNQLLRVKYLLVYSQKPPKRAS................................................  204
Xenopus_laevis_A1A620                   160  DVPQKYFVVTNNQLLRVKYLMVYSQ.......................................................  184

Naegleria_gruberi_81181                 150  .....YYVVPQAEHITTRFFCFYPKSDTNVN.................................................  175
Naegleria_gruberi_80022                 159  MTPNPYFVIPNEEHLQTRAFFIYTTNQ.....................................................  185
Lottia_gigantea_53025                   189  .....IWVAANSDHVCTRFFFVYEDGQVGDSN.......................INTQVTKYRNEI.............  227
Branchiostoma_floridae_C3YQ85           154  .....IWVVPNPDHVCTRFFFVYEDGQVGDSM.......................VDTTQEKYQKEIL............  193
Xenopus_tropicalis_A4IGR1               189  .....IWVCPVSDHVCTRFFFVYEDGQVGDAN.......................INTQDPKIQKEIM............  228
Homo_sapiens_PARP6                      188  .....IWVCPVSDHVCTRFFFVYEDGQVGDAN.......................INTQDPKIQKEIM............  227
Gallus_gallus_XP_001232753              189  .....IWVCPVSDHVCTRFFFVYEDGQVGDAN.......................INTQDPKIQKEIM............  228
Homo_sapiens_PARP8                      189  .....IWVVPNTDHVCTRFFFVYEDGQVGDAN.......................INTQEGGIHKEIL............  228
Danio_rerio_Q08CN1                      189  .....IWVVPNTDHVCTRFFFVYEDGQVGDTS.......................INTQEAGIHREIL............  229

Trichomonas_vaginalis_94489             142  .....VHTLQDANAVIVRFILVNVKG......................................................  162
Trichomonas_vaginalis_A2DLU6            140  ....GILTCANEDAVNIRFVFTNPM.......................................................  160
Trichomonas_vaginalis_87871             141  .....AYTLMNEKAIVVRFLFV..........................................................  157
Trichomonas_vaginalis_86141             145  .....CHTLMQEEACIVRFLMLNLSRSIDIIN.......................QQPQNIPTLRDVL............  184
Trichomonas_vaginalis_96758             142  ....FCHVLRDESECIVRFLFI..........................................................  159
Trichomonas_vaginalis_90528             145  ....FCHTLKNEKECIVRFLFI..........................................................  162
Trichomonas_vaginalis_88521             147  ....FCHTLTNEKLCIVRFLFINVKTAEFNT.................................................  173
Nectria_haematococca_83215              142  .....THVVTDETRVLVR..............................................................  154

Physcomitrella_patens_A9TVE2            166  TREANVFVVSRECDVAIRYLLVFK........................................................  189
```

Homo Sapiens PARP8 Structure: β-sheet

Core β-4   Core β-5

Laccaria_bicolor_B0DPJ7	219	......	218
Phanerochaete_chrysosporium_8174	238	......	237
Magnaporthe_grisea_A4R2D2	235	......	234
Botryotinia_fuckeliana_A6S9M7	193	......	192
Cryphonectria_parasitica_38809	179	......	178
Penicillium_marneffei_B6Q3H5	195	......	194
Talaromyces_stipitatus_B8M6M3	202	......	201
Mycosphaerella_fijiensis_16474	198	......	197
Phaeosphaeria_nodorum_Q0UPJ2	181	......	180
Pyrenophora_tritici-repentis_B2VYK4	187	......	186
Alternaria_brassicicola_AB0502	192	......	191
Cochliobolus_heterostrophus_30785	196	......	195
Aspergillus_terreus_Q0CPK4	224	......	223
Neosartorya_fischeri_A1CVV6	200	......	199
Aspergillus_fumigatus_Q4WQT4	200	......	199
Aspergillus_clavatus_A1CIW1	172	......	171
Microsporum_canis_C5FD62	210	......	209
Ajellomyces_dermatitidis_C5K0M	203	......	202
Chlorella_sp_NC64A_142217	198	......	197
Chlamydomonas_reinhardtii_408636	266	LKQHRY	271
Volvox_carteri_107792	258	LQQHRY	263
Daphnia_pulex_187911	185	......	184
Trichoplax_adhaerens_B3RSW6	162	......	161
Ixodes_scapularis_B7Q2N5	184	......	183
Ciona_intestinalis_376237	210	......	209
Helobdella_robusta_67096	208	......	207
Nematostella_vectensis_A7SF57	184	......	183
Danio_rerio_Q6NX05	185	......	184
Homo_sapiens_PAR16	205	......	204
Xenopus_laevis_A1A620	185	......	184
Naegleria_gruberi_81181	176	......	175
Naegleria_gruberi_80022	186	......	185
Lottia_gigantea_53025	228	......	227
Branchiostoma_floridae_C3YQ85	194	......	193
Xenopus_tropicalis_A4IGR1	229	......	228
Homo_sapiens_PARP6	228	......	227
Gallus_gallus_XP_001232753	229	......	228
Homo_sapiens_PARP8	229	......	228
Danio_rerio_Q08CN1	230	......	229
Trichomonas_vaginalis_94489	163	......	162
Trichomonas_vaginalis_A2DLU6	161	......	160
Trichomonas_vaginalis_87871	158	......	157
Trichomonas_vaginalis_86141	185	......	184
Trichomonas_vaginalis_96758	160	......	159
Trichomonas_vaginalis_90528	163	......	162
Trichomonas_vaginalis_88521	174	......	173
Nectria_haematococca_83215	155	......	154
Physcomitrella_patens_A9TVE2	190	......	189
