# Supplementary material for: Evolutionary history of the poly(ADP-ribose) polymerase gene family in eukaryotes
Source: BMC Evol Biol. 2010 Oct 13;10:308. doi: 10.1186/1471-2148-10-308 (PMC2964712; doi:10.1186/1471-2148-10-308)
Supplement: Additional file 12 — Multiple alignment of the UBCc domain of Clade 6A PARPs. The entire UBCc domains as defined by Pfam from Clade 6A proteins are shown. [file 1471-2148-10-308-S12.PDF]

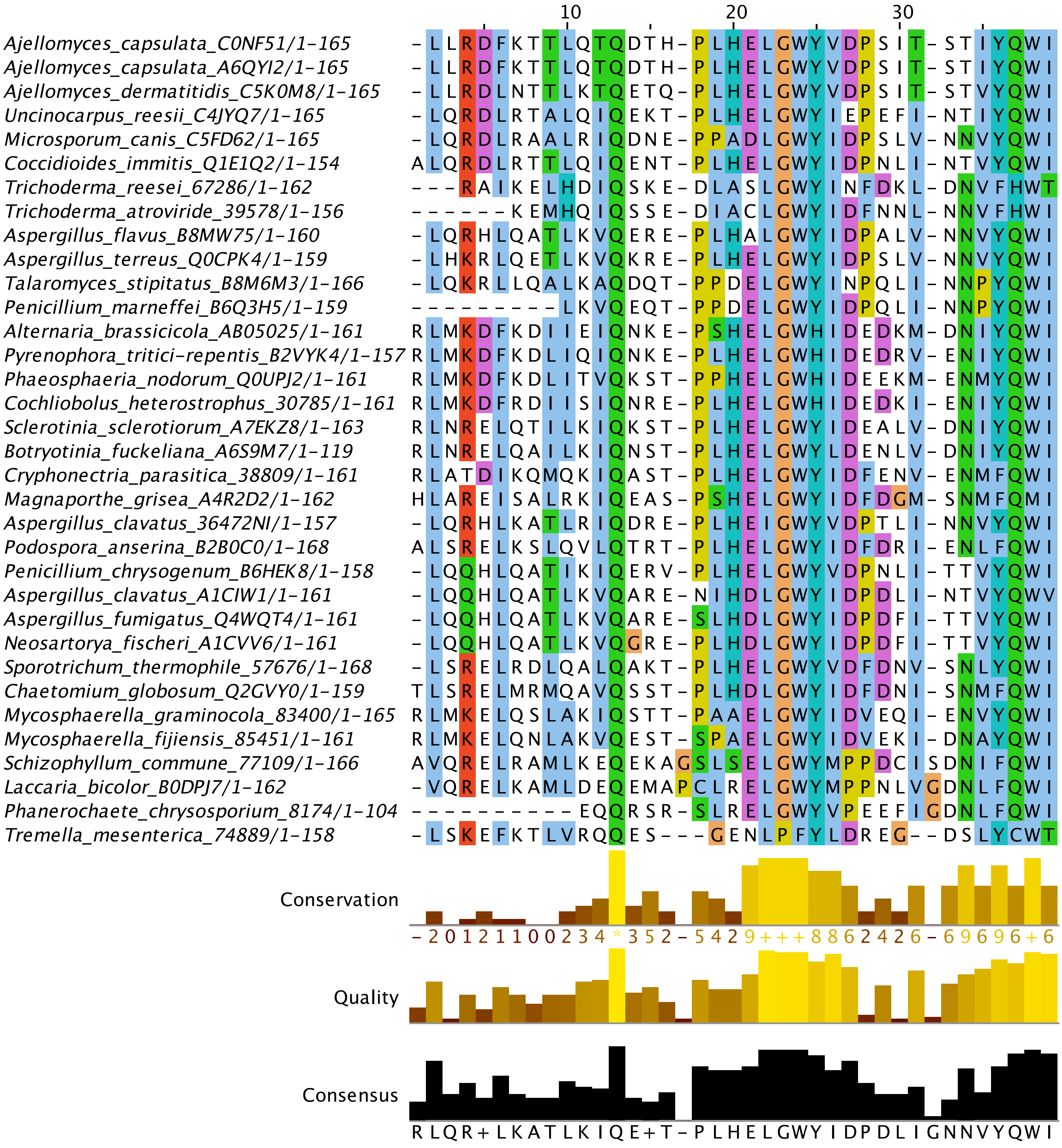
10
20
30
Ajellomyces_capsulata_C0NF51/1-165 -LLRDFKTTLQTQDTH-PLHELGWYVDPSIT-STIYQWI
Ajellomyces_capsulata_A6QYI2/1-165 -LLRDFKTTLQTQDTH-PLHELGWYVDPSIT-STIYQWI
Ajellomyces_dermatitidis_C5K0M8/1-165 -LLRDLNTTLKTQETQ-PLHELGWYVDPSIT-STVYQWI
Uncinocarpus_reesii_C4JYQ7/1-165 -LQRDLRTALQIQEKT-PLHELGWYIEPEFI-NTIYQWI
Microsporum_canis_C5FD62/1-165 -LQRDLRAALRIQDNE-PPADLGWYIDPSLV-NNVYQWI
Coccidioides_immitis_Q1E1Q2/1-154 ALQRDLRTTLQIQENT-PLHELGWYIDPNLI-NTVYQWI
Trichoderma_reesei_67286/1-162 ---RAIKELHDIQSKE-DLASLGWYINFDKL-DNVFHWT
Trichoderma_atroviride_39578/1-156 ------KEMHQIQSSE-DIACLGWYIDFNNL-NNVFHWI
Aspergillus_flavus_B8MW75/1-160 -LQRHLQATLKVQERE-PLHALGWYIDPALV-NNVYQWI
Aspergillus_terreus_Q0CPK4/1-159 -LHKRLQETLKVQKRE-PLHELGWYIDPSLV-NNVYQWI
Talaromyces_stipitatus_B8M6M3/1-166 -LQKRLLQALKAQDQT-PPDELGWYINPQLI-NNPYQWI
Penicillium_marneffei_B6Q3H5/1-159 ---------LKVQEQT-PPDELGWYIDPQLI-NNPYQWI
Alternaria_brassicicola_AB05025/1-161 RLMKDFKDIIEIQNKE-PSHELGWHIDEDKM-DNIYQWI
Pyrenophora_tritici-repentis_B2VYK4/1-157 RLMKDFKDLIQIQNKE-PLHELGWHIDEDRV-ENIYQWI
Phaeosphaeria_nodorum_Q0UPJ2/1-161 RLMKDFKDLITVQKST-PPHELGWHIDEEKM-ENMYQWI
Cochliobolus_heterostrophus_30785/1-161 RLMKDFRDIISIQNRE-PLHELGWHIDEDKI-ENIYQWI
Sclerotinia_sclerotiorum_A7EKZ8/1-163 RLNRELQTILKIQKST-PLHELGWYIDEALV-DNIYQWI
Botryotinia_fuckeliana_A6S9M7/1-119 RLNRELQAILKIQNST-PLHELGWYLDENLV-DNIYQWI
Cryphonectria_parasitica_38809/1-161 RLATDIKQMQKIQAST-PLHELGWYIDFENV-ENMFQWI
Magnaporthe_grisea_A4R2D2/1-162 HLAREISALRKIQEAS-PSHELGWYIDFDGM-SNMFQMI
Aspergillus_clavatus_36472NI/1-157 -LQRHLKATLRIQDRE-PLHEIGWYVDPTLI-NNVYQWI
Podospora_anserina_B2B0C0/1-168 ALSRELKSLQVLQTRT-PLHELGWYIDFDRI-ENLFQWI
Penicillium_chrysogenum_B6HEK8/1-158 -LQQHLQATIKIQERV-PLHELGWYVDPNLI-TTVYQWI
Aspergillus_clavatus_A1CIW1/1-161 -LQQHLQATLKVQARE-NIHDLGWYIDPDLI-NTVYQWV
Aspergillus_fumigatus_Q4WQT4/1-161 -LQQHLQATLKVQARE-SLHDLGWYIDPDFI-TTVYQWI
Neosartorya_fischeri_A1CVV6/1-161 -LQQHLQATLKVQGRE-PLHDLGWYIDPDFI-TTVYQWI
Sporotrichum_thermophile_57676/1-168 -LSRELRDLQALQAKT-PLHELGWYVDFDNV-SNLYQWI
Chaetomium_globosum_Q2GVY0/1-159 TLSRELMRMQAVQSST-PLHDLGWYIDFDNI-SNMFQWI
Mycosphaerella_graminocola_83400/1-165 RLMKELQSLAKIQSTT-PAAELGWYIDVEQI-ENVYQWI
Mycosphaerella_fijiensis_85451/1-161 RLMKELQNLAKVQEST-SPAELGWYIDVEKI-DNAYQWI
Schizophyllum_commune_77109/1-166 AVQRELRAMLKEQEKAGSLSELGWYMPPDCISDNIFQWI
Laccaria_bicolor_B0DPJ7/1-162 -VQRELKAMLDEQEMAPCLRELGWYMPPNLVGDNLFQWI
Phanerochaete_chrysosporium_8174/1-104 ----------EQQRSR-SLRELGWYVPEEFIGDNLFQWI
Tremella_mesenterica_74889/1-158 -LSKEFKTLVRQQES---GENLPFYLDREG--DSLYCWT
Conservation
- 2 0 1 2 1 1 0 0 2 3 4 * 3 5 2 - 5 4 2 9 + + + 8 8 6 2 4 2 6 - 6 9 6 9 6 + 6
Quality
Consensus
RLQR+LKATLKIQE+T-PLHELGWYIDPDLIGNNVYQWI

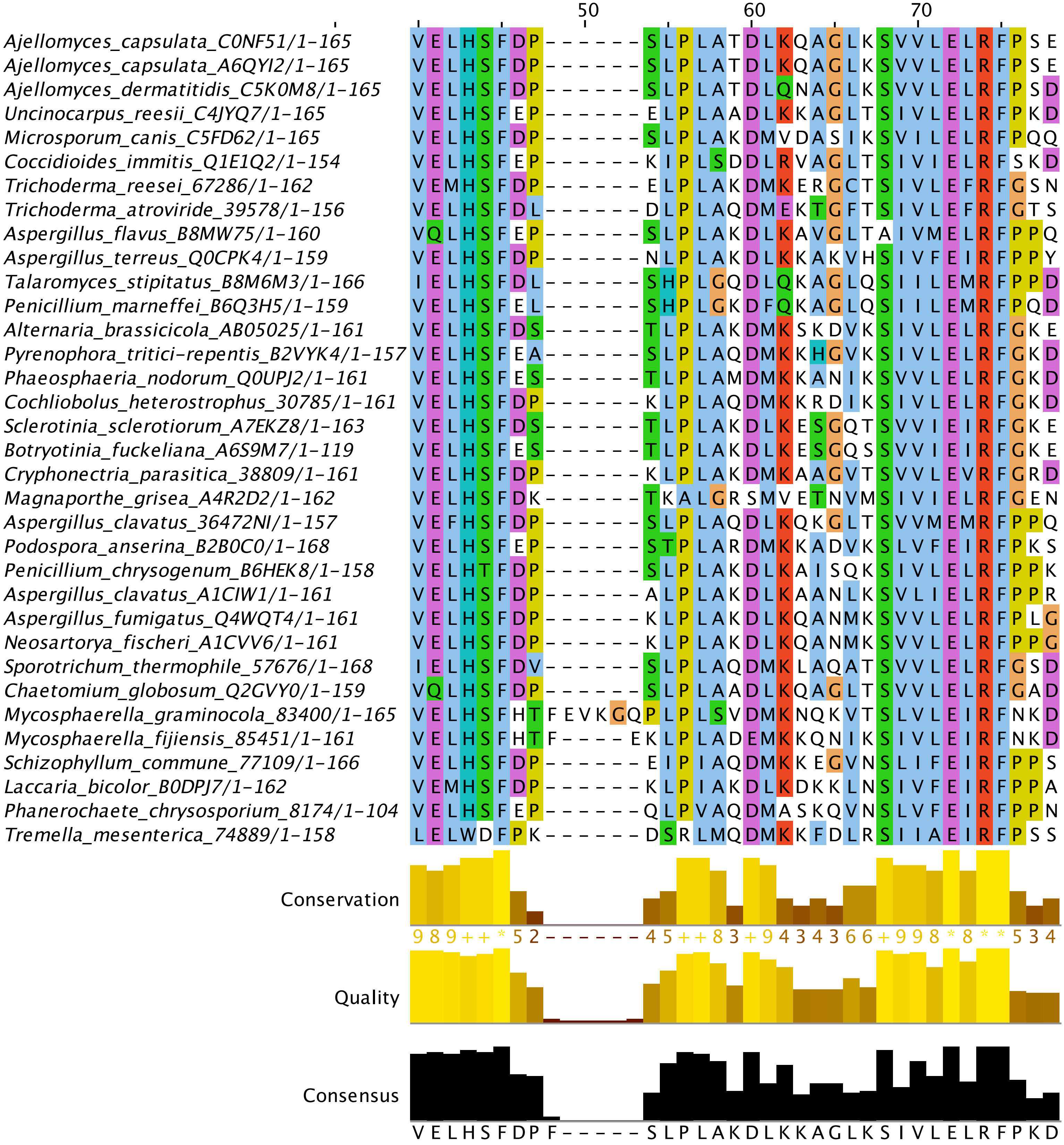

50
60
70
Ajellomyces_capsulata_C0NF51/1-165 VELHSFDP------SLPLATDLKQAGLKSVVLELRFPSE
Ajellomyces_capsulata_A6QYI2/1-165 VELHSFDP------SLPLATDLKQAGLKSVVLELRFPSE
Ajellomyces_dermatitidis_C5K0M8/1-165 VELHSFDP------SLPLATDLQNAGLKSVVLELRFPSD
Uncinocarpus_reesii_C4JYQ7/1-165 VELHSFEP------ELPLAADLKKAGLTSIVLELRFPKD
Microsporum_canis_C5FD62/1-165 VELHSFDP------SLPLAKDMVDASIKSVILELRFPQQ
Coccidioides_immitis_Q1E1Q2/1-154 VELHSFEP------KIPLSDDLRVAGLTSIVIELRFSKD
Trichoderma_reesei_67286/1-162 VEMHSFDP------ELPLAKDMKERGCTSIVLEFRFGSN
Trichoderma_atroviride_39578/1-156 VELHSFDL------DLPLAQDMEKTGFTSIVLEFRFGTS
Aspergillus_flavus_B8MW75/1-160 VQLHSFEP------SLPLAKDLKAVGLTAIVMELRFPPQ
Aspergillus_terreus_Q0CPK4/1-159 VELHSFDP------NLPLAKDLKKAKVHSIVFEIRFPPY
Talaromyces_stipitatus_B8M6M3/1-166 IELHSFDL------SHPLGQDLQKAGLQSIILEMRFPPD
Penicillium_marneffei_B6Q3H5/1-159 VELHSFEL------SHPLGKDFQKAGLQSIILEMRFPQD
Alternaria_brassicicola_AB05025/1-161 VELHSFDS------TLPLAKDMKSKDVKSIVLELRFGKE
Pyrenophora_tritici-repentis_B2VYK4/1-157 VELHSFEA------SLPLAQDMKKHGVKSIVLELRFGKD
Phaeosphaeria_nodorum_Q0UPJ2/1-161 VELHSFES------TLPLAMDMKKANIKSVVLELRFGKD
Cochliobolus_heterostrophus_30785/1-161 VELHSFDP------KLPLAQDMKKRDIKSIVLELRFGKD
Sclerotinia_sclerotiorum_A7EKZ8/1-163 VELHSFDS------TLPLAKDLKESGQTSVVIEIRFGKE
Botryotinia_fuckeliana_A6S9M7/1-119 VELHSFES------TLPLAKDLKESGQSSVVIEIRFGKE
Cryphonectria_parasitica_38809/1-161 VELHSFDP------KLPLAKDMKAAGVTSVVLEVRFGRD
Magnaporthe_grisea_A4R2D2/1-162 VELHSFDK------TKALGRSMVETNVMSIVIELRFGEN
Aspergillus_clavatus_36472NI/1-157 VEFHSFDP------SLPLAQDLKQKGLTSVVMEMRFPPQ
Podospora_anserina_B2B0C0/1-168 VELHSFEP------STPLARDMKKADVKSLVFEIRFPKS
Penicillium_chrysogenum_B6HEK8/1-158 VELHTFDP------SLPLAKDLKAISQKSIVLELRFPPK
Aspergillus_clavatus_A1CIW1/1-161 VELHSFDP------ALPLAKDLKAANLKSVLIELRFPPR
Aspergillus_fumigatus_Q4WQT4/1-161 VELHSFDP------KLPLAKDLKQANMKSVVLELRFPLG
Neosartorya_fischeri_A1CVV6/1-161 VELHSFDP------KLPLAKDLKQANMKSVVLELRFPPG
Sporotrichum_thermophile_57676/1-168 IELHSFDV------SLPLAQDMKLAQATSVVLELRFGSD
Chaetomium_globosum_Q2GVY0/1-159 VQLHSFDP------SLPLAADLKQAGLTSVVLELRFGAD
Mycosphaerella_graminocola_83400/1-165 VELHSFHTFEVKGQPLPLSVDMKNQKVTSLVLEIRFNKD
Mycosphaerella_fijiensis_85451/1-161 VELHSFHTF----EKLPLADEMKKQNIKSIVLEIRFNKD
Schizophyllum_commune_77109/1-166 VELHSFDP------EIPIAQDMKKEGVNSLIFEIRFPPS
Laccaria_bicolor_B0DPJ7/1-162 VEMHSFDP------KLPIAKDLKDKKLNSIVFEIRFPPA
Phanerochaete_chrysosporium_8174/1-104 VELHSFEP------QLPVAQDMASKQVNSLVFEIRFPPN
Tremella_mesenterica_74889/1-158 LELWDFPK------DSRLMQDMKKFDLRSIIAEIRFPSS
Conservation
989++*52------45++83+94343366+998*8**534
Quality
Consensus
VELHSFDPF-----SLPLAKDLKKAGLKSIVLELRFPKD

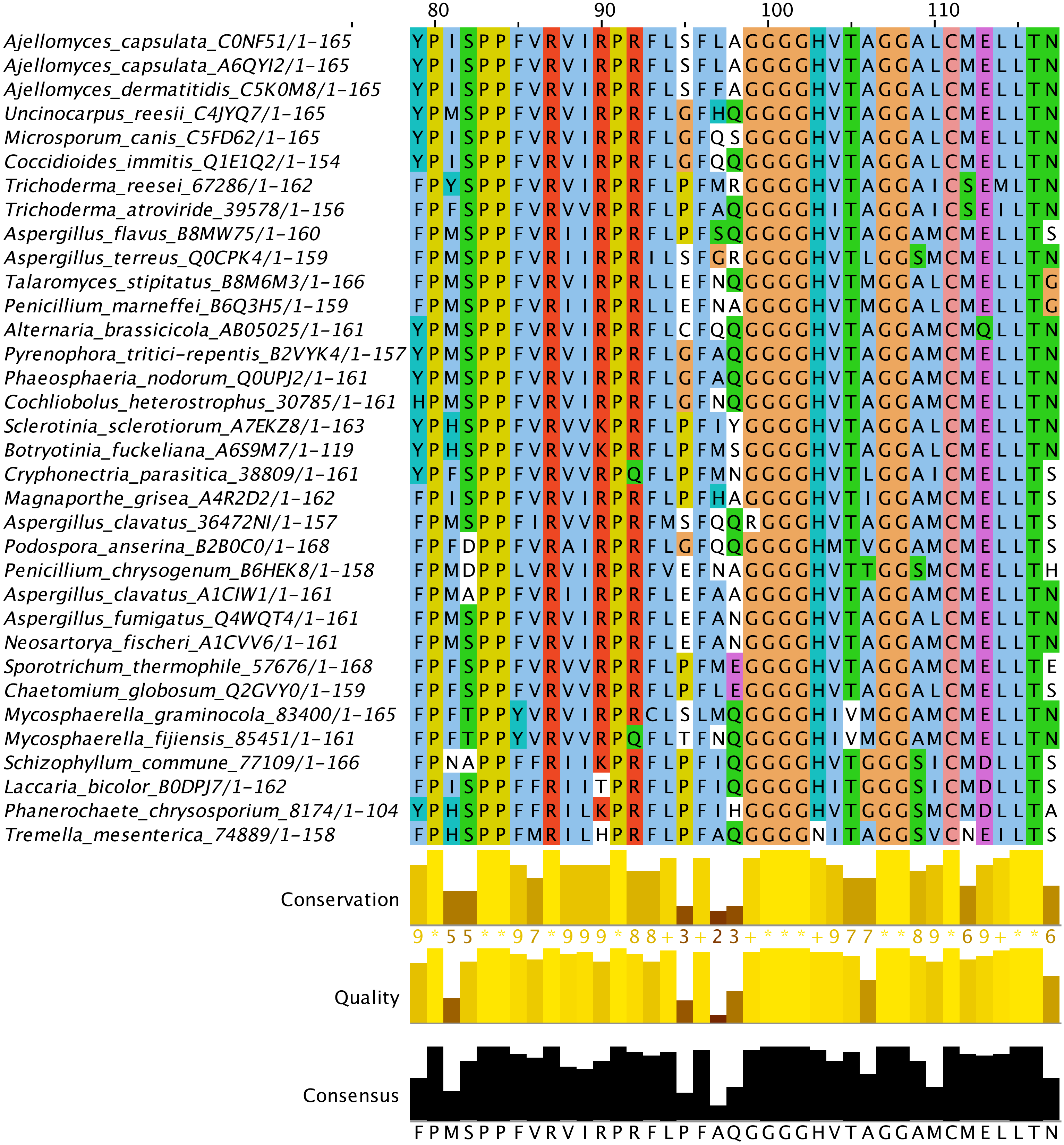
80 90 100 110
Ajellomyces_capsulata_C0NF51/1-165 YPISPPFVRVIRPRFLSFLAGGGGHVTAGGALCMELLTN
Ajellomyces_capsulata_A6QYI2/1-165 YPISPPFVRVIRPRFLSFLAGGGGHVTAGGALCMELLTN
Ajellomyces_dermatitidis_C5K0M8/1-165 YPISPPFVRVIRPRFLSFFAGGGGHVTAGGALCMELLTN
Uncinocarpus_reesii_C4JYQ7/1-165 YPMSPPFVRVIRPRFLGFHQGGGGHVTAGGALCMELLTN
Microsporum_canis_C5FD62/1-165 YPISPPFVRVIRPRFLGFQSGGGGHVTAGGALCMELLTN
Coccidioides_immitis_Q1E1Q2/1-154 YPISPPFVRVIRPRFLGFQQGGGGHVTAGGALCMELLTN
Trichoderma_reesei_67286/1-162 FPYSPPFVRVIRPRFLPFMRGGGGHVTAGGAICSEMLTN
Trichoderma_atroviride_39578/1-156 FPFSPPFVRVVRPRFLPFAQGGGGHITAGGAICSEILTN
Aspergillus_flavus_B8MW75/1-160 FPMSPPFVRIIRPRFLPFSQGGGGHVTAGGAMCMELLTS
Aspergillus_terreus_Q0CPK4/1-159 FPMSPPFVRIIRPRILSFGRGGGGHVTLGGSMCMELLTN
Talaromyces_stipitatus_B8M6M3/1-166 FPMSPPFVRVIRPRLLEFNQGGGGHVTMGGALCMELLTG
Penicillium_marneffei_B6Q3H5/1-159 FPMSPPFVRIIRPRLLEFNAGGGGHVTMGGALCMELLTG
Alternaria_brassicicola_AB05025/1-161 YPMSPPFVRVIRPRFLCFQQGGGGHVTAGGAMCMQLLTN
Pyrenophora_tritici-repentis_B2VYK4/1-157 YPMSPPFVRVIRPRFLGFAQGGGGHVTAGGAMCMELLTN
Phaeosphaeria_nodorum_Q0UPJ2/1-161 YPMSPPFVRVIRPRFLGFAQGGGGHVTAGGAMCMELLTN
Cochliobolus_heterostrophus_30785/1-161 HPMSPPFVRVIRPRFLGFNQGGGGHVTAGGAMCMELLTN
Sclerotinia_sclerotiorum_A7EKZ8/1-163 YPHSPPFVRVVKPRFLPFIYGGGGHVTAGGALCMELLTN
Botryotinia_fuckeliana_A6S9M7/1-119 YPHSPPFVRVVKPRFLPFMSGGGGHVTAGGALCMELLTN
Cryphonectria_parasitica_38809/1-161 YPFSPPFVRVVRPQFLPFMNGGGGHVTLGGAICMELLTS
Magnaporthe_grisea_A4R2D2/1-162 FPISPPFVRVIRPRFLPFHAGGGGHVTIGGAMCMELLTS
Aspergillus_clavatus_36472NI/1-157 FPMSPPFIRVVRPRFMSFQQRGGGHVTAGGAMCMELLTS
Podospora_anserina_B2B0C0/1-168 FPFDPPFVRAIRPRFLGFQQGGGGHMTVGGAMCMELLTS
Penicillium_chrysogenum_B6HEK8/1-158 FPMDPPLVRVIRPRFVEFNAGGGGHVTTGGSMCMELLTH
Aspergillus_clavatus_A1CIW1/1-161 FPMAPPFVRIIRPRFLEFAAGGGGHVTAGGAMCMELLTN
Aspergillus_fumigatus_Q4WQT4/1-161 FPMSPPFVRVIRPRFLEFANGGGGHVTAGGALCMELLTN
Neosartorya_fischeri_A1CVV6/1-161 FPMSPPFVRVIRPRFLEFANGGGGHVTAGGAMCMELLTN
Sporotrichum_thermophile_57676/1-168 FPFSPPFVRVVRPRFLPFMEGGGGHVTAGGAMCMELLTE
Chaetomium_globosum_Q2GVY0/1-159 FPFSPPFVRVVRPRFLPFLEGGGGHVTAGGALCMELLTS
Mycosphaerella_graminocola_83400/1-165 FPFTPPYVRVIRPRCLSLMQGGGGHIVMGGAMCMELLTN
Mycosphaerella_fijiensis_85451/1-161 FPFTPPYVRVVRPQFLTFNQGGGGHIVMGGAMCMELLTN
Schizophyllum_commune_77109/1-166 FPNAPPFFRIIKPRFLPFIQGGGGHVTGGGSICMDLLTS
Laccaria_bicolor_B0DPJ7/1-162 FPISPPFFRIITPRFLPFIQGGGGHITGGGSICMDLLTS
Phanerochaete_chrysosporium_8174/1-104 YPHSPPFFRILKPRFLPFIHGGGGHVTGGGSMCMDLLTA
Tremella_mesenterica_74889/1-158 FPHSPPFMRILHPRFLPFAQGGGGNITAGGSVCNEILTS
Conservation
9 * 5 5 * * 9 7 * 9 9 9 * 8 8 + 3 + 2 3 + * * * + 9 7 7 * * 8 9 * 6 9 + * * 6
Quality
Consensus
FPMSPPFVRVIRPRFLPFAQGGGGHVTAGGAMCMELLTN

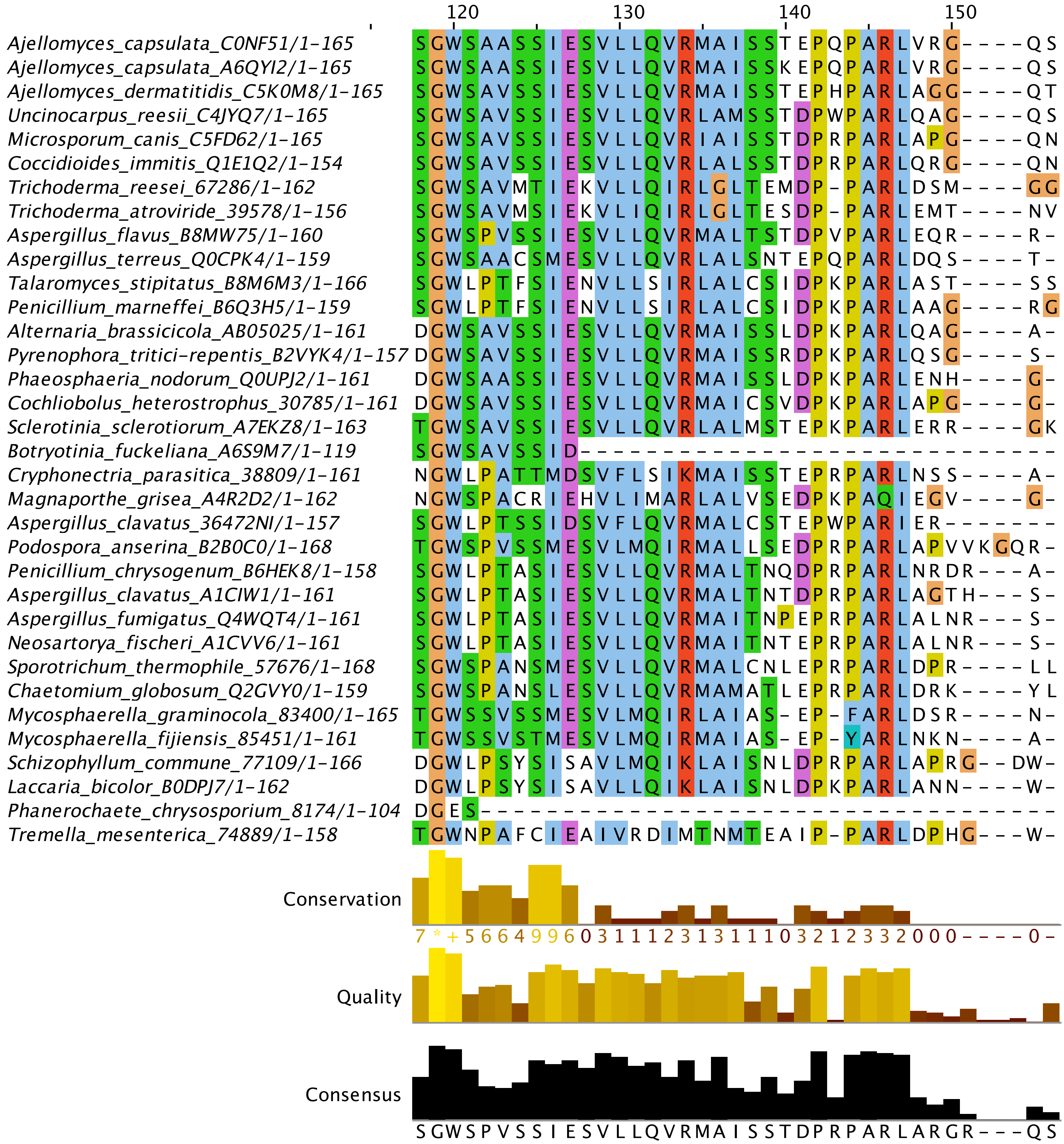

120
130
140
150
Ajellomyces_capsulata_C0NF51/1-165 SGWSAASSIESVLLQVRMAISSTEPQPARLVRG----QS
Ajellomyces_capsulata_A6QYI2/1-165 SGWSAASSIESVLLQVRMAISSKEPQPARLVRG----QS
Ajellomyces_dermatitidis_C5K0M8/1-165 SGWSAVSSIESVLLQVRMAISSTEPHPARLAGG----QT
Uncinocarpus_reesii_C4JYQ7/1-165 SGWSAVSSIESVLLQVRLAMSSTDPWPARLQAG----QS
Microsporum_canis_C5FD62/1-165 SGWSAVSSIESVLLQVRIAISSTDPRPARLAPG----QN
Coccidioides_immitis_Q1E1Q2/1-154 SGWSAVSSIESVLLQVRLALSSTDPRPARLQRG----QN
Trichoderma_reesei_67286/1-162 SGWSAVMTIEKVLLQIRLGLTEMDP-PARLDSM----GG
Trichoderma_atroviride_39578/1-156 SGWSAVMSIEKVLIQIRLGLTESDP-PARLEMT----NV
Aspergillus_flavus_B8MW75/1-160 SGWSPVSSIESVLLQVRMALTSTDPVPARLEQR----R-
Aspergillus_terreus_Q0CPK4/1-159 SGWSAACSMESVLLQVRLALSNTEPQPARLDQS----T-
Talaromyces_stipitatus_B8M6M3/1-166 SGWLPTFSIENVLLSIRLALCSIDPKPARLAST----SS
Penicillium_marneffei_B6Q3H5/1-159 SGWLPTFSIENVLLSIRLALCSIDPKPARLAAG----RG
Alternaria_brassicicola_AB05025/1-161 DGWSAVSSIESVLLQVRMAISSLDPKPARLQAG----A-
Pyrenophora_tritici-repentis_B2VYK4/1-157 DGWSAVSSIESVLLQVRMAISSRDPKPARLQSG----S-
Phaeosphaeria_nodorum_Q0UPJ2/1-161 DGWSAASSIESVLLQVRMAISSLDPKPARLENH----G-
Cochliobolus_heterostrophus_30785/1-161 DGWSAVSSIESVLLQVRMAICSVDPKPARLAPG----G-
Sclerotinia_sclerotiorum_A7EKZ8/1-163 TGWSAVSSIESVLLQVRLALMSTEPKPARLERR----GK
Botryotinia_fuckeliana_A6S9M7/1-119 SGWSAVSSID-----------------------------
Cryphonectria_parasitica_38809/1-161 NGWLPATTMDSVFLSIKMAISSTEPRPARLNSS----A-
Magnaporthe_grisea_A4R2D2/1-162 NGWSPACRIEHVLIMARLALVSEDPKPAQIEGV----G-
Aspergillus_clavatus_36472NI/1-157 SGWLPTSSIDSVFLQVRMALCSTEPWPARIER-------
Podospora_anserina_B2B0C0/1-168 TGWSPVSSMESVLMQIRMALLSEDPRPARLAPVVKGQR-
Penicillium_chrysogenum_B6HEK8/1-158 SGWLPTASIESVLLQVRMALTNQDPRPARLNRDR---A-
Aspergillus_clavatus_A1CIW1/1-161 SGWLPTASIESVLLQVRMALTNTDPRPARLAGTH---S-
Aspergillus_fumigatus_Q4WQT4/1-161 SGWLPTASIESVLLQVRMAITNPEPRPARLALNR---S-
Neosartorya_fischeri_A1CVV6/1-161 SGWLPTASIESVLLQVRMAITNTEPRPARLALNR---S-
Sporotrichum_thermophile_57676/1-168 SGWSPANSMESVLLQVRMALCNLEPRPARLDPR----LL
Chaetomium_globosum_Q2GVY0/1-159 SGWSPANSLESVLLQVRMAMATLEPRPARLDRK----YL
Mycosphaerella_graminocola_83400/1-165 TGWSSVSSMESVLMQIRLAIAS-EP-FARLDSR----N-
Mycosphaerella_fijiensis_85451/1-161 TGWSSVSTMESVLMQIRMAIAS-EP-YARLNKN----A-
Schizophyllum_commune_77109/1-166 DGWLPSYSISAVLMQIKLAISNLDPRPARLAPRG--DW-
Laccaria_bicolor_B0DPJ7/1-162 DGWLPSYSISAVLLQIKLAISNLDPKPARLANN----W-
Phanerochaete_chrysosporium_8174/1-104 DGES-----------------------------------
Tremella_mesenterica_74889/1-158 TGWNPAFCIEAIVRDIMTNMTEAIP-PARLDPHG---W-
Conservation
7*+56649996031112313111032123320000----0-
Quality
Consensus
SGWSPVSSIESVLLQVRMAISSTDPRPARLARGR---QS

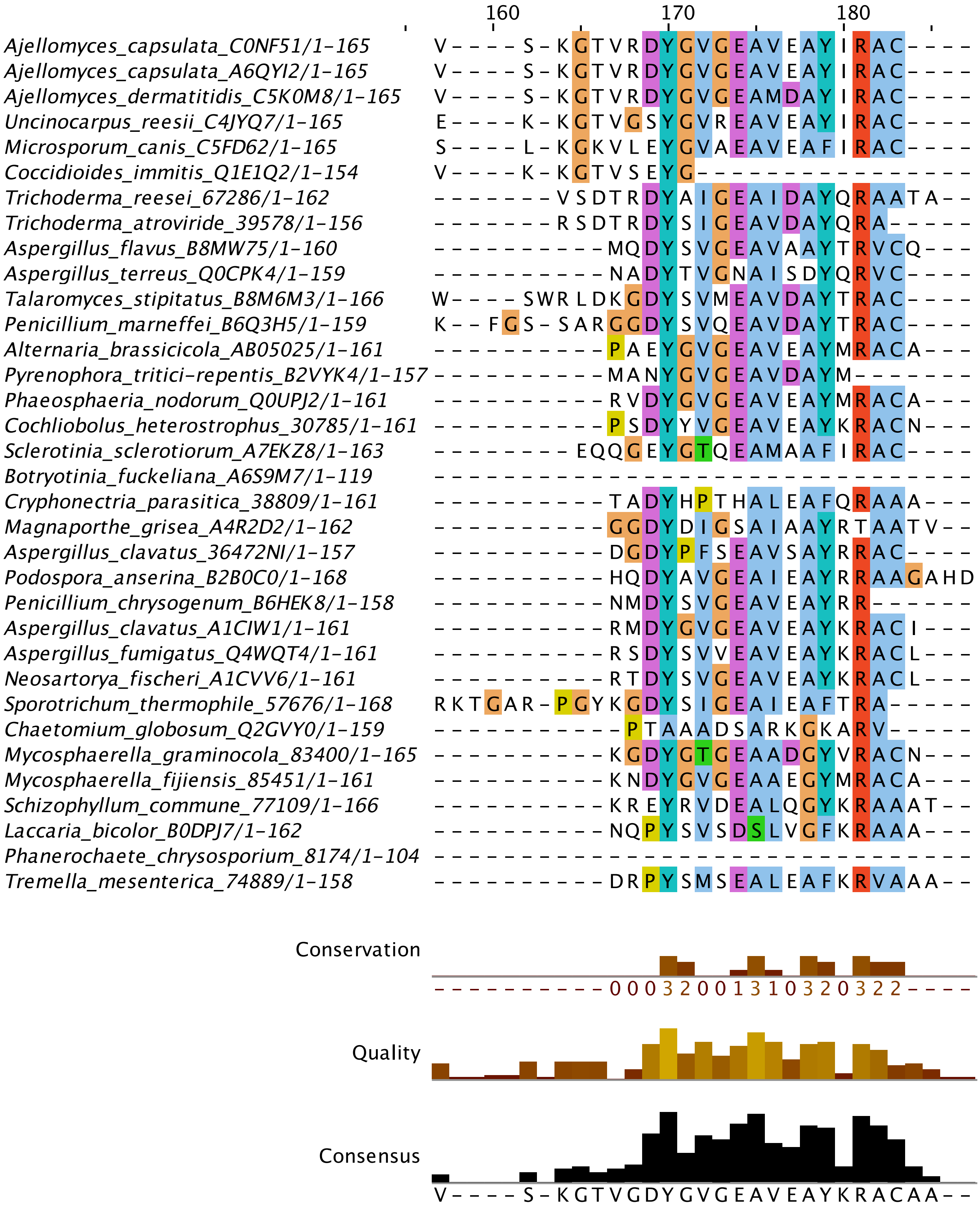

160
170
180
Ajellomyces_capsulata_C0NF51/1-165 V----S-KGTVRDYGVGEAVEAYIRAC----
Ajellomyces_capsulata_A6QYI2/1-165 V----S-KGTVRDYGVGEAVEAYIRAC----
Ajellomyces_dermatitidis_C5K0M8/1-165 V----S-KGTVRDYGVGEAMDAYIRAC----
Uncinocarpus_reesii_C4JYQ7/1-165 E----K-KGTVGSYGVREAVEAYIRAC----
Microsporum_canis_C5FD62/1-165 S----L-KGKVLEYGVAEAVEAFIRAC----
Coccidioides_immitis_Q1E1Q2/1-154 V----K-KGTVSEYG----------------
Trichoderma_reesei_67286/1-162 -------VSDTRDYAIGEAIDAYQRAATA--
Trichoderma_atroviride_39578/1-156 -------RSDTRDYSIGEAVDAYQRA-----
Aspergillus_flavus_B8MW75/1-160 ----------MQDYSVGEAVAAYTRVCQ---
Aspergillus_terreus_Q0CPK4/1-159 ----------NADYTVGNAISDYQRVC----
Talaromyces_stipitatus_B8M6M3/1-166 W----SWRLDKGDYSVMEAVDAYTRAC----
Penicillium_marneffei_B6Q3H5/1-159 K--FGS-SARGGDYSVQEAVDAYTRAC----
Alternaria_brassicicola_AB05025/1-161 ----------PAEYGVGEAVEAYMRACA---
Pyrenophora_tritici-repentis_B2VYK4/1-157 ----------MANYGVGEAVDAYM-------
Phaeosphaeria_nodorum_Q0UPJ2/1-161 ----------RVDYGVGEAVEAYMRACA---
Cochliobolus_heterostrophus_30785/1-161 ----------PSDYYVGEAVEAYKRACN---
Sclerotinia_sclerotiorum_A7EKZ8/1-163 --------EQQGEYGTQEAMAAFIRAC----
Botryotinia_fuckeliana_A6S9M7/1-119 -------------------------------
Cryphonectria_parasitica_38809/1-161 ----------TADYHPTHALEAFQRAAA---
Magnaporthe_grisea_A4R2D2/1-162 ----------GGDYDIGSAIAAYRTAATV--
Aspergillus_clavatus_36472NI/1-157 ----------DGDYPFSEAVSAYRRAC----
Podospora_anserina_B2B0C0/1-168 ----------HQDYAVGEAIEAYRRAAGAHD
Penicillium_chrysogenum_B6HEK8/1-158 ----------NMDYSVGEAVEAYRR------
Aspergillus_clavatus_A1CIW1/1-161 ----------RMDYGVGEAVEAYKRACI---
Aspergillus_fumigatus_Q4WQT4/1-161 ----------RSDYSVVEAVEAYKRACL---
Neosartorya_fischeri_A1CVV6/1-161 ----------RTDYSVGEAVEAYKRACL---
Sporotrichum_thermophile_57676/1-168 RKTGAR-PGYKGDYSIGEAIEAFTRA-----
Chaetomium_globosum_Q2GVY0/1-159 -----------PTAAADSARKGKARV-----
Mycosphaerella_graminocola_83400/1-165 ----------KGDYGTGEAADGYVRACN---
Mycosphaerella_fijiensis_85451/1-161 ----------KNDYGVGEAAEGYMRACA---
Schizophyllum_commune_77109/1-166 ----------KREYRVDEALQGYKRAAAT--
Laccaria_bicolor_B0DPJ7/1-162 ----------NQPYSVSDSLVGFKRAAA---
Phanerochaete_chrysosporium_8174/1-104 -------------------------------
Tremella_mesenterica_74889/1-158 ----------DRPYSMSEALEAFKRVAAA--
Conservation
----------00032001310320322----
Quality
Consensus
V----S-KGTVGDYGVGEAVEAYKRACAA--
