# Supplementary material for: Evolutionary history of the poly(ADP-ribose) polymerase gene family in eukaryotes
Source: BMC Evol Biol. 2010 Oct 13;10:308. doi: 10.1186/1471-2148-10-308 (PMC2964712; doi:10.1186/1471-2148-10-308)
Supplement: Additional file 13 — Clade 6A PARP proteins contain FPE and UBCc domains. A. Clade 6A PARPs contain UBCc domains in their C termini. An alignment of the HMM consensus sequence of the UBCc domain from Pfam (UBCc) and the UBCc domain from Phaeosphaeria nodorum QOUPJ2 (Pn6F). The sequence similarity between the UBCc and UBCc-like domains is shown in red (CONS). +, similar amino acids; -, gaps introduced to maximize the alignment; ., any amino acid. Residues in bold have been shown to be diagnostic of UBCc domains as discussed in the text and [85]. B. Alignment of a region of the Clade 6A PARP UBCc-like domains, containing the catalytic cysteine. The names of the proteins and the amino acid positions (within the UBCc domain) are indicated at left. The blue asterisk marks a histidine and the red asterisk marks the catalytic cysteine, both shared with typical UBCc domains. C. The FPE domain consists of alpha helices and beta strands. The sequence of the FPE domain from the Phaeosphaeria nodorum Clade 6A member (QOUPJ2) is shown. Secondary structural characteristics as detected by Phyre are shown above the sequence. h, alpha helices; e, beta strands. [file 1471-2148-10-308-S13.PDF]

A.

```
UBCc: RLqkElk.El ektelqkdpp pgisagpvdd adgpnnlfeW
CONS: RL+K++K+++     + qk+ p p      + +d+ +   + n++ W
Pn6F: RLMKDFKDLI ---TVQKSTP PHELGWHIDE EKME-NMYQW
UBCc: evtIiGPegT p.....YeG GvFk...ldieF PedYPfkPP
CONS:  v ++  e T ++    +   +     k+  l++ F + dYP++PP
Pn6F: IVELHSFEST LPLAMDMKKA NIKSVVLELRF GKDYPMSPP
UBCc: kv........ .......kFt TkiyHPNVds psGeiCLdIL
CONS:  v+  +++    +  ++++          H        + G+ C   L
Pn6F: FVRVIRPRFL GFAQGGGG-- ----HVT--- AGGAMCMELL
UBCc: kPGDDpgYEa derddevWsP altvrsvLls iqsLLL....
CONS:             +++   Ws+ a +++svLl  +      +  ++
Pn6F: T--------- -NDG---WSA ASSIESVLLQ VR---MAISS
UBCc: sePyNpesPl naeaAklyke n.reefkkrv rerv
CONS:  +P  p+    n+    +y     + +e   r+   ++
Pn6F: LDP-KPARLE NHGRV-DYGV GEAVEAYMRA --CA
```

B.

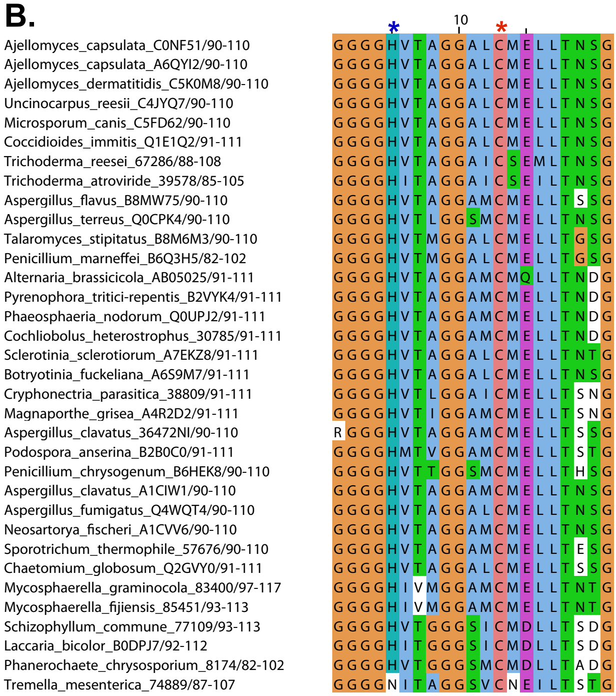

C.

```
  hhhhhhhh hhhhh          eee            eeeeeee
MPRKDFQRDL IGAKVSGRFP HLHSVGAGDH DGSITFTFAD TSTST
eeeee eeee           eeee         hhhh hhhhhhhhh
RIDFQ AIVSDAQDYPE NHTYFVFVTT SEDPPSRVVT VMENAQSRFL
    hhhhhhhh hhhhhh
GVALEDFLTYI DEIVQNALR LPASEADDDE KVGYGVDDTD FVDDD
                       hhh hhhhhhhhh hhhhh    eee
DNDDD VDWDMENEP VFGISRADEK HLLKTRRDL RAVKNAGLKVG
eee      e eeeeeeeeee     hhhhhh h       eeee eee
CLGTLTGAV IVSVSCRIGR LGISEEAMEA WNVRASEYLVL LMRYP
     hhhhhh          eeeee ee         hh hhhhhh
GTYV DFQELLALGK AKYPAIQFHV GLCDSYKPTTE DAIRAFQGN
                      hhhhhhhhhhh hhhhhhhhh      h
LSLSEEGLTG TARLRSLFIE QPLDSLLNERF LRIMELRYH LGLSW
hhhhh hhhh                                hhhhhhhhhh
TGAEL YIQQNQGRRP DYGAITDNYFE PDTWSASAP ALFQNDHIGQ
hh
GLGVD
```
