# Supplementary material for: Evolutionary history of the poly(ADP-ribose) polymerase gene family in eukaryotes
Source: BMC Evol Biol. 2010 Oct 13;10:308. doi: 10.1186/1471-2148-10-308 (PMC2964712; doi:10.1186/1471-2148-10-308)
Supplement: Additional file 14 — Multiple alignment of the FPE from Clade 6A PARP proteins. The entire PfamB_30617/FPE domains as defined by Pfam from Clade 6A proteins are shown. [file 1471-2148-10-308-S14.PDF]

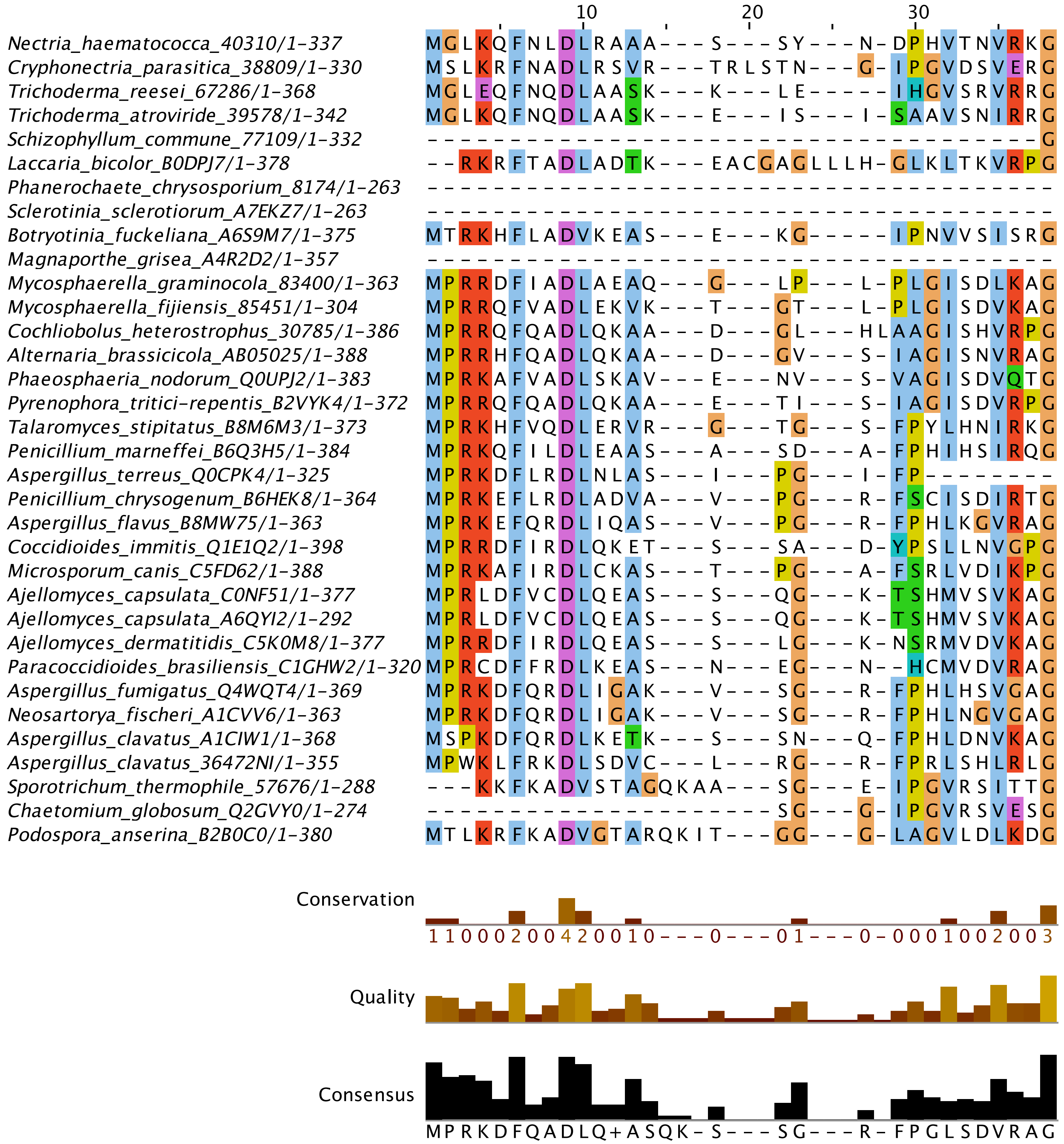
10
20
30
Nectria_haematococca_40310/1-337 MGLKQFNLDLRAAA---S---SY---N-DPHVTNVRKG
Cryphonectria_parasitica_38809/1-330 MSLKRFNADLRSVR---TRLSTN---G-IPGVDSVERG
Trichoderma_reesei_67286/1-368 MGLEQFNQDLAASK---K---LE-----IHGVSRVRRG
Trichoderma_atroviride_39578/1-342 MGLKQFNQDLAASK---E---IS---I-SAAVSNIRRG
Schizophyllum_commune_77109/1-332 -------------------------------------G
Laccaria_bicolor_B0DPJ7/1-378 --RKRFTADLADTK---EACGAGLLLH-GLKLTKVRPG
Phanerochaete_chrysosporium_8174/1-263 --------------------------------------
Sclerotinia_sclerotiorum_A7EKZ7/1-263 --------------------------------------
Botryotinia_fuckeliana_A6S9M7/1-375 MTRKHFLADVKEAS---E---KG-----IPNVVSISRG
Magnaporthe_grisea_A4R2D2/1-357 --------------------------------------
Mycosphaerella_graminocola_83400/1-363 MPRRDFIADLAEAQ---G---LP---L-PLGISDLKAG
Mycosphaerella_fijiensis_85451/1-304 MPRRQFVADLEKVK---T---GT---L-PLGISDVKAG
Cochliobolus_heterostrophus_30785/1-386 MPRRQFQADLQKAA---D---GL---HLAAGISHVRPG
Alternaria_brassicicola_AB05025/1-388 MPRRHFQADLQKAA---D---GV---S-IAGISNVRAG
Phaeosphaeria_nodorum_Q0UPJ2/1-383 MPRKAFVADLSKAV---E---NV---S-VAGISDVQTG
Pyrenophora_tritici-repentis_B2VYK4/1-372 MPRRQFQADLQKAA---E---TI---S-IAGISDVRPG
Talaromyces_stipitatus_B8M6M3/1-373 MPRKHFVQDLERVR---G---TG---S-FPYLHNIRKG
Penicillium_marneffei_B6Q3H5/1-384 MPRKQFILDLEAAS---A---SD---A-FPHIHSIRQG
Aspergillus_terreus_Q0CPK4/1-325 MPRKDFLRDLNLAS---I---PG---I-FP--------
Penicillium_chrysogenum_B6HEK8/1-364 MPRKEFLRDLADVA---V---PG---R-FSCISDIRTG
Aspergillus_flavus_B8MW75/1-363 MPRKEFQRDLIQAS---V---PG---R-FPHLKGVRAG
Coccidioides_immitis_Q1E1Q2/1-398 MPRRDFIRDLQKET---S---SA---D-YPSLLNVGPG
Microsporum_canis_C5FD62/1-388 MPRKAFIRDLCKAS---T---PG---A-FSRLVDIKPG
Ajellomyces_capsulata_C0NF51/1-377 MPRLDFVCDLQEAS---S---QG---K-TSHMVSVKAG
Ajellomyces_capsulata_A6QYI2/1-292 MPRLDFVCDLQEAS---S---QG---K-TSHMVSVKAG
Ajellomyces_dermatitidis_C5K0M8/1-377 MPRRDFIRDLQEAS---S---LG---K-NSRMVDVKAG
Paracoccidioides_brasiliensis_C1GHW2/1-320 MPRCDFFRDLKEAS---N---EG---N--HCMVDVRAG
Aspergillus_fumigatus_Q4WQT4/1-369 MPRKDFQRDLIGAK---V---SG---R-FPHLHSVGAG
Neosartorya_fischeri_A1CVV6/1-363 MPRKDFQRDLIGAK---V---SG---R-FPHLNGVGAG
Aspergillus_clavatus_A1CIW1/1-368 MSPKDFQRDLKETK---S---SN---Q-FPHLDNVKAG
Aspergillus_clavatus_36472NI/1-355 MPWKLFRKDLSDVC---L---RG---R-FPRLSHLRLG
Sporotrichum_thermophile_57676/1-288 ---KKFKADVSTAGQKAA---SG---E-IPGVRSITTG
Chaetomium_globosum_Q2GVY0/1-274 ---------------------SG---G-IPGVRSVESG
Podospora_anserina_B2B0C0/1-380 MTLKRFKADVGTARQKIT---GG---G-LAGVLDLKDG
Conservation
11000200420010---0---01---0-0001002003
Quality
Consensus
MPRKDFQADLQ+ASQK-S---SG---R-FPGLSDVRAG

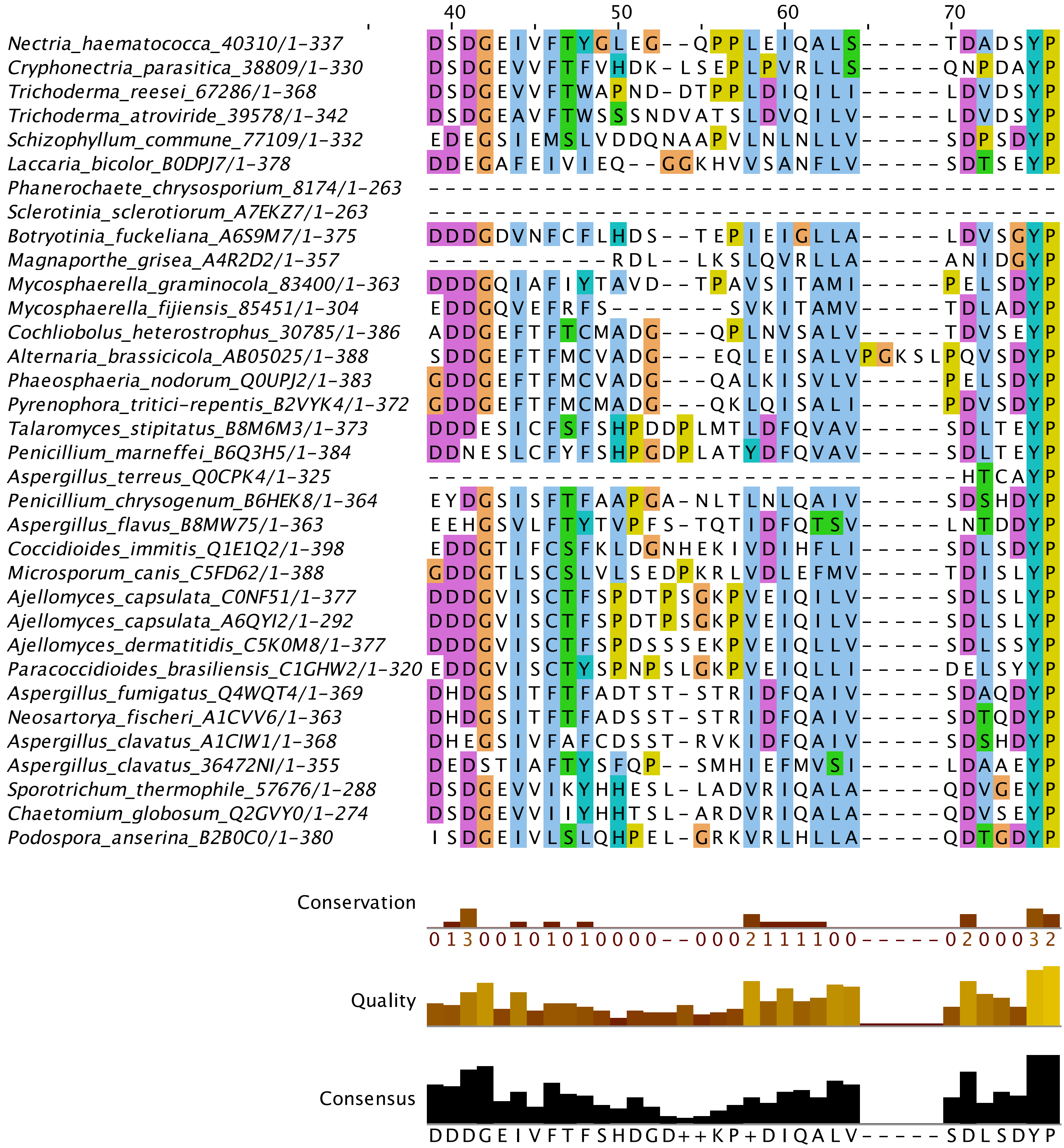
40
50
60
70
Nectria_haematococca_40310/1-337
Cryphonectria_parasitica_38809/1-330
Trichoderma_reesei_67286/1-368
Trichoderma_atroviride_39578/1-342
Schizophyllum_commune_77109/1-332
Laccaria_bicolor_B0DPJ7/1-378
Phanerochaete_chrysosporium_8174/1-263
Sclerotinia_sclerotiorum_A7EKZ7/1-263
Botryotinia_fuckeliana_A6S9M7/1-375
Magnaporthe_grisea_A4R2D2/1-357
Mycosphaerella_graminocola_83400/1-363
Mycosphaerella_fijiensis_85451/1-304
Cochliobolus_heterostrophus_30785/1-386
Alternaria_brassicicola_AB05025/1-388
Phaeosphaeria_nodorum_Q0UPJ2/1-383
Pyrenophora_tritici-repentis_B2VYK4/1-372
Talaromyces_stipitatus_B8M6M3/1-373
Penicillium_marneffei_B6Q3H5/1-384
Aspergillus_terreus_Q0CPK4/1-325
Penicillium_chrysogenum_B6HEK8/1-364
Aspergillus_flavus_B8MW75/1-363
Coccidioides_immitis_Q1E1Q2/1-398
Microsporum_canis_C5FD62/1-388
Ajellomyces_capsulata_C0NF51/1-377
Ajellomyces_capsulata_A6QYI2/1-292
Ajellomyces_dermatitidis_C5K0M8/1-377
Paracoccidioides_brasiliensis_C1GHW2/1-320
Aspergillus_fumigatus_Q4WQT4/1-369
Neosartorya_fischeri_A1CVV6/1-363
Aspergillus_clavatus_A1CIW1/1-368
Aspergillus_clavatus_36472NI/1-355
Sporotrichum_thermophile_57676/1-288
Chaetomium_globosum_Q2GVY0/1-274
Podospora_anserina_B2B0C0/1-380
Conservation
013001010100000--0002111100-----020003 2
Quality
Consensus
DDDGEIVFTFSHDGD++KP+DIQALV-----SDLSDYP

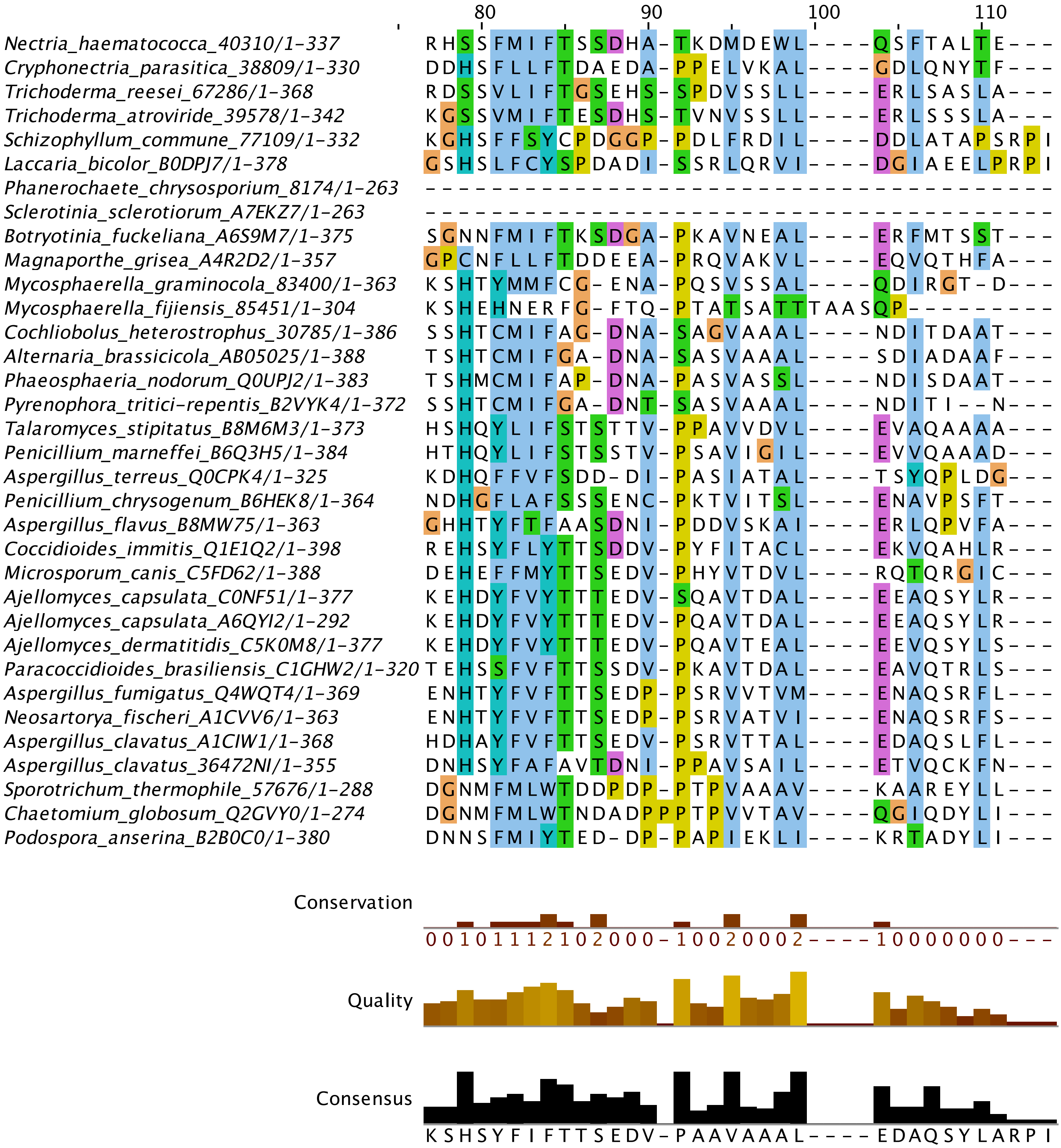
80 90 100 110
Nectria_haematococca_40310/1-337 RHSSFMIFTSSDHA-TKDMDEWL----QSFTALTE---
Cryphonectria_parasitica_38809/1-330 DDHSFLLFTDAEDA-PPELVKAL----GDLQNYTF---
Trichoderma_reesei_67286/1-368 RDSSVLIFTGSEHS-SPDVSSLL----ERLSASLA---
Trichoderma_atroviride_39578/1-342 KGSSVMIFTESDHS-TVNVSSLL----ERLSSSLA---
Schizophyllum_commune_77109/1-332 KGHSFFSYCPDGGP-PDLFRDIL----DDLATAPSRPI
Laccaria_bicolor_B0DPJ7/1-378 GSHSLFCYSPDADI-SSRLQRVI----DGIAEELPRPI
Phanerochaete_chrysosporium_8174/1-263 --------------------------------------
Sclerotinia_sclerotiorum_A7EKZ7/1-263 --------------------------------------
Botryotinia_fuckeliana_A6S9M7/1-375 SGNNFMIFTKSDGA-PKAVNEAL----ERFMTSST---
Magnaporthe_grisea_A4R2D2/1-357 GPCNFLLFTDDEEA-PRQVAKVL----EQVQTHFA---
Mycosphaerella_graminocola_83400/1-363 KSHTYMMFCG-ENA-PQSVSSAL----QDIRGT-D---
Mycosphaerella_fijiensis_85451/1-304 KSHEHNERFG-FTQ-PTATSATTTAASQP---------
Cochliobolus_heterostrophus_30785/1-386 SSHTCMIFAG-DNA-SAGVAAAL----NDITDAAT---
Alternaria_brassicicola_AB05025/1-388 TSHTCMIFGA-DNA-SASVAAAL----SDIADAAF---
Phaeosphaeria_nodorum_Q0UPJ2/1-383 TSHMCMIFAP-DNA-PASVASSL----NDISDAAT---
Pyrenophora_tritici-repentis_B2VYK4/1-372 SSHTCMIFGA-DNT-SASVAAAL----NDITI--N---
Talaromyces_stipitatus_B8M6M3/1-373 HSHQYLIFSTSTTV-PPAVVDVL----EVAQAAAA---
Penicillium_marneffei_B6Q3H5/1-384 HTHQYLIFSTSSTV-PSAVIGIL----EVVQAAAD---
Aspergillus_terreus_Q0CPK4/1-325 KDHQFFVFSDD-DI-PASIATAL----TSYQPLDG---
Penicillium_chrysogenum_B6HEK8/1-364 NDHGFLAFSSSENC-PKTVITSL----ENAVPSFT---
Aspergillus_flavus_B8MW75/1-363 GHHTYFTFAASDNI-PDDVSKAI----ERLQPVFA---
Coccidioides_immitis_Q1E1Q2/1-398 REHSYFLYTTSDDV-PYFITACL----EKVQAHLR---
Microsporum_canis_C5FD62/1-388 DEHEFFMYTTSEDV-PHYVTDVL----RQTQRGIC---
Ajellomyces_capsulata_C0NF51/1-377 KEHDYFVYTTTEDV-SQAVTDAL----EEAQSYLR---
Ajellomyces_capsulata_A6QYI2/1-292 KEHDYFVYTTTEDV-PQAVTDAL----EEAQSYLR---
Ajellomyces_dermatitidis_C5K0M8/1-377 KEHDYFVYTTTEDV-PQAVTEAL----EEVQSYLS---
Paracoccidioides_brasiliensis_C1GHW2/1-320 TEHSSFVFTTSSDV-PKAVTDAL----EAVQTRLS---
Aspergillus_fumigatus_Q4WQT4/1-369 ENHTYFVFTTSEDP-PSRVVTVM----ENAQSRFL---
Neosartorya_fischeri_A1CVV6/1-363 ENHTYFVFTTSEDP-PSRVATVI----ENAQSRFS---
Aspergillus_clavatus_A1CIW1/1-368 HDHAYFVFTTSEDV-PSRVTTAL----EDAQSLFL---
Aspergillus_clavatus_36472NI/1-355 DNHSYFAFAVTDNI-PPAVSAIL----ETVQCKFN---
Sporotrichum_thermophile_57676/1-288 DGNMFMLWTDDPDP-PTPVAAAV----KAAREYLL---
Chaetomium_globosum_Q2GVY0/1-274 DGNMFMLWTNDADPPPTPVVTAV----QGIQDYLI---
Podospora_anserina_B2B0C0/1-380 DNNSFMIYTED-DP-PAPIEKLI----KRTADYLI---
Conservation
00101112102000-10020002----10000000---
Quality
Consensus
KSHSYFIFTTSEDV-PAAVAAAL----EDAQSYLARPI

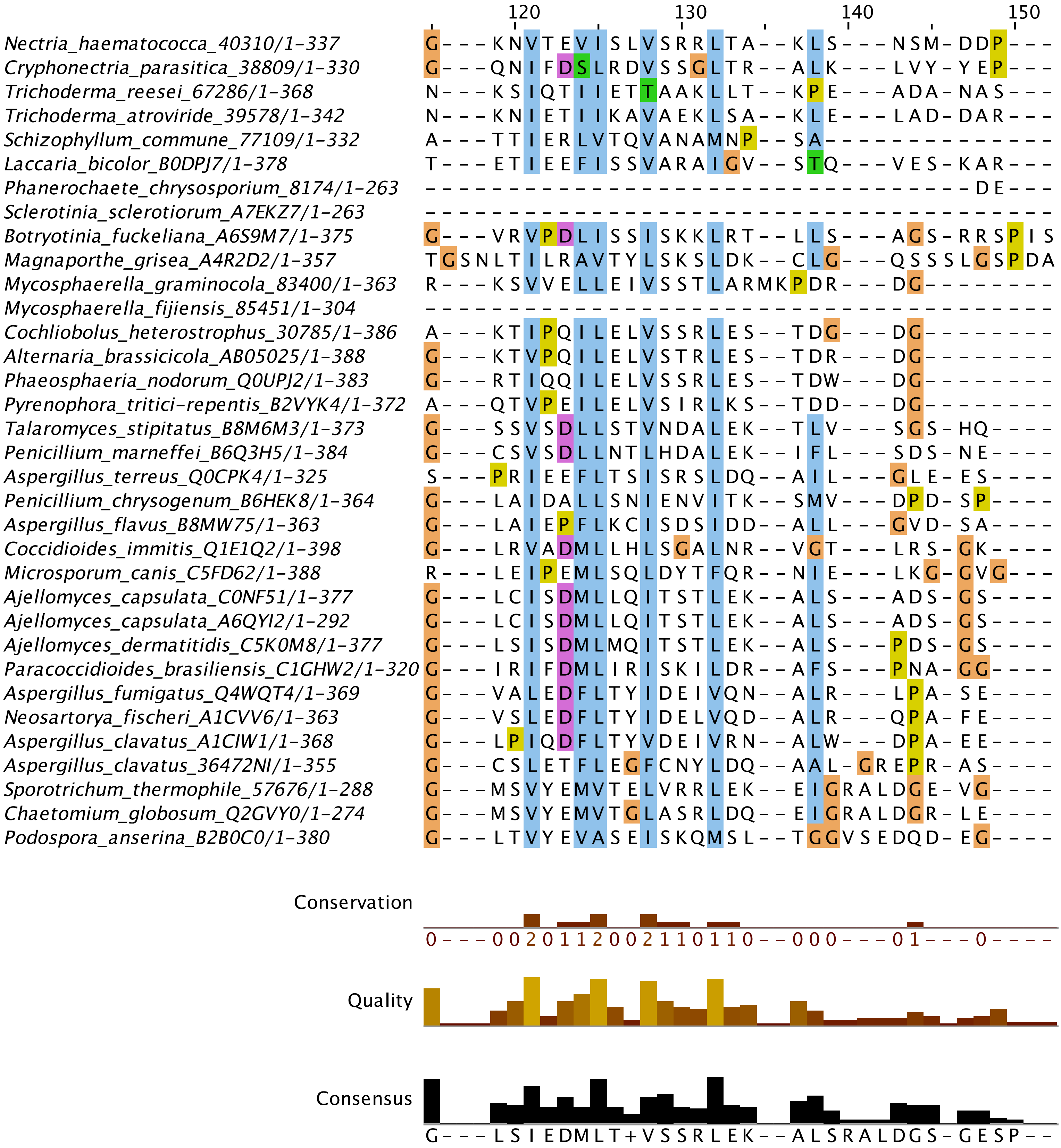
120
130
140
150
Nectria_haematococca_40310/1-337
Cryphonectria_parasitica_38809/1-330
Trichoderma_reesei_67286/1-368
Trichoderma_atroviride_39578/1-342
Schizophyllum_commune_77109/1-332
Laccaria_bicolor_B0DPJ7/1-378
Phanerochaete_chrysosporium_8174/1-263
Sclerotinia_sclerotiorum_A7EKZ7/1-263
Botryotinia_fuckeliana_A6S9M7/1-375
Magnaporthe_grisea_A4R2D2/1-357
Mycosphaerella_graminocola_83400/1-363
Mycosphaerella_fijiensis_85451/1-304
Cochliobolus_heterostrophus_30785/1-386
Alternaria_brassicicola_AB05025/1-388
Phaeosphaeria_nodorum_Q0UPJ2/1-383
Pyrenophora_tritici-repentis_B2VYK4/1-372
Talaromyces_stipitatus_B8M6M3/1-373
Penicillium_marneffei_B6Q3H5/1-384
Aspergillus_terreus_Q0CPK4/1-325
Penicillium_chrysogenum_B6HEK8/1-364
Aspergillus_flavus_B8MW75/1-363
Coccidioides_immitis_Q1E1Q2/1-398
Microsporum_canis_C5FD62/1-388
Ajellomyces_capsulata_C0NF51/1-377
Ajellomyces_capsulata_A6QYI2/1-292
Ajellomyces_dermatitidis_C5K0M8/1-377
Paracoccidioides_brasiliensis_C1GHW2/1-320
Aspergillus_fumigatus_Q4WQT4/1-369
Neosartorya_fischeri_A1CVV6/1-363
Aspergillus_clavatus_A1CIW1/1-368
Aspergillus_clavatus_36472NI/1-355
Sporotrichum_thermophile_57676/1-288
Chaetomium_globosum_Q2GVY0/1-274
Podospora_anserina_B2B0C0/1-380
Conservation
0---0020112002110110--000---01---0----
Quality
Consensus
G---LSIEDMLT+VSSRLEK--ALSRALDGS-GESP--

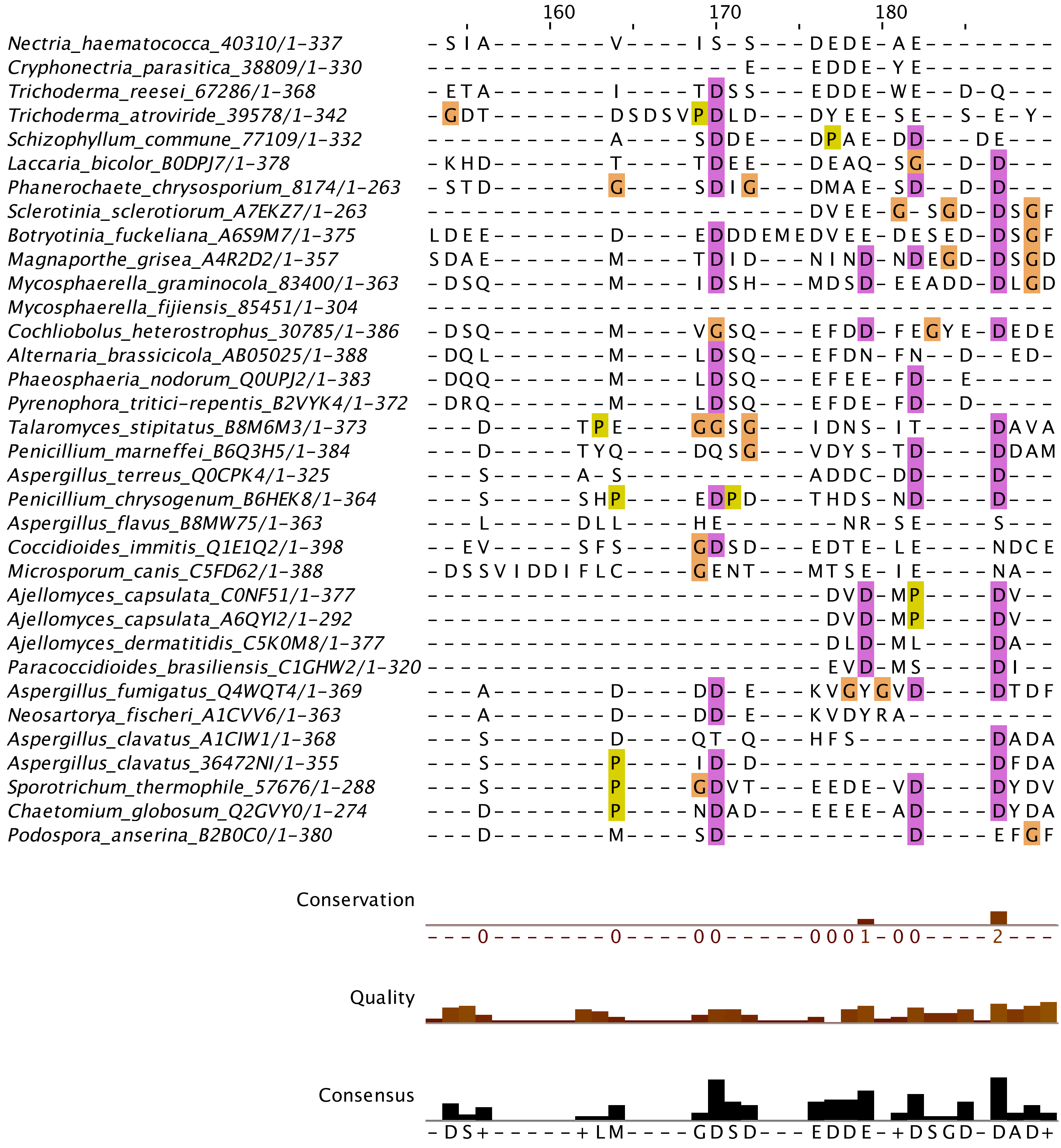
160 170 180
Nectria_haematococca_40310/1-337 -SIA--------V----IS-S---DEDE-AE--------
Cryphonectria_parasitica_38809/1-330 -------------------E---EDDE-YE--------
Trichoderma_reesei_67286/1-368 -ETA--------I----TDSS---EDDE-WE--D-Q---
Trichoderma_atroviride_39578/1-342 -GDT-------DSDSVPDLD---DYEE-SE--S-E-Y-
Schizophyllum_commune_77109/1-332 -----------A----SDDE---DPAE-DD---DE---
Laccaria_bicolor_B0DPJ7/1-378 -KHD--------T----TDEE---DEAQ-SG--D-D---
Phanerochaete_chrysosporium_8174/1-263 -STD--------G----SDIG--DMAE-SD--D-D---
Sclerotinia_sclerotiorum_A7EKZ7/1-263 ------------------------DVEE-G-SGD-DSGF
Botryotinia_fuckeliana_A6S9M7/1-375 LDEE--------D----EDDDEMEDVEE-DESED-DSGF
Magnaporthe_grisea_A4R2D2/1-357 SDAE--------M----TDID--NIND-NDEGD-DSGD
Mycosphaerella_graminocola_83400/1-363 -DSQ--------M----IDSH--MDSD-EEADD-DLGD
Mycosphaerella_fijiensis_85451/1-304 --------------------------------------
Cochliobolus_heterostrophus_30785/1-386 -DSQ--------M----VGSQ---EFDD-FEGYE-DEDE
Alternaria_brassicicola_AB05025/1-388 -DQL--------M----LDSQ---EFDN-FN--D--ED-
Phaeosphaeria_nodorum_Q0UPJ2/1-383 -DQQ--------M----LDSQ---EFEE-FD--E-----
Pyrenophora_tritici-repentis_B2VYK4/1-372 -DRQ--------M----LDSQ---EFDE-FD--D-----
Talaromyces_stipitatus_B8M6M3/1-373 ---D-----TPE----GGSG---IDNS-IT----DAVA
Penicillium_marneffei_B6Q3H5/1-384 ---D-----TYQ----DQSG---VDYS-TD----DDAM
Aspergillus_terreus_Q0CPK4/1-325 ---S-----A-S-----------ADDC-DD----D---
Penicillium_chrysogenum_B6HEK8/1-364 ---S-----SHP----EDPD---THDS-ND----D---
Aspergillus_flavus_B8MW75/1-363 ---L-----DLL----HE--------NR-SE----S---
Coccidioides_immitis_Q1E1Q2/1-398 --EV-----SFS----GDSD---EDTE-LE----NDCE
Microsporum_canis_C5FD62/1-388 -DSSVIDDIFLC----GENT---MTSE-IE----NA--
Ajellomyces_capsulata_C0NF51/1-377 -----------------------DVD-MP----DV--
Ajellomyces_capsulata_A6QYI2/1-292 -----------------------DVD-MP----DV--
Ajellomyces_dermatitidis_C5K0M8/1-377 -----------------------DLD-ML----DA--
Paracoccidioides_brasiliensis_C1GHW2/1-320 -----------------------EVD-MS----DI--
Aspergillus_fumigatus_Q4WQT4/1-369 ---A--------D----DD-E---KVGYGVD----DTDF
Neosartorya_fischeri_A1CVV6/1-363 ---A--------D----DD-E---KVDYRA---------
Aspergillus_clavatus_A1CIW1/1-368 ---S--------D----QT-Q---HFS--------DADA
Aspergillus_clavatus_36472NI/1-355 ---S--------P----ID-D-------------DFDA
Sporotrichum_thermophile_57676/1-288 ---S--------P----GDVT---EEDE-VD----DYDV
Chaetomium_globosum_Q2GVY0/1-274 ---D--------P----NDAD---EEEE-AD----DYDA
Podospora_anserina_B2B0C0/1-380 ---D--------M----SD-----------D----EFGF
Conservation
---0--------0----00-----0001-00----2---
Quality
Consensus
-DS+-----+LM----GDSD---EDDE-+DSGD-DAD+

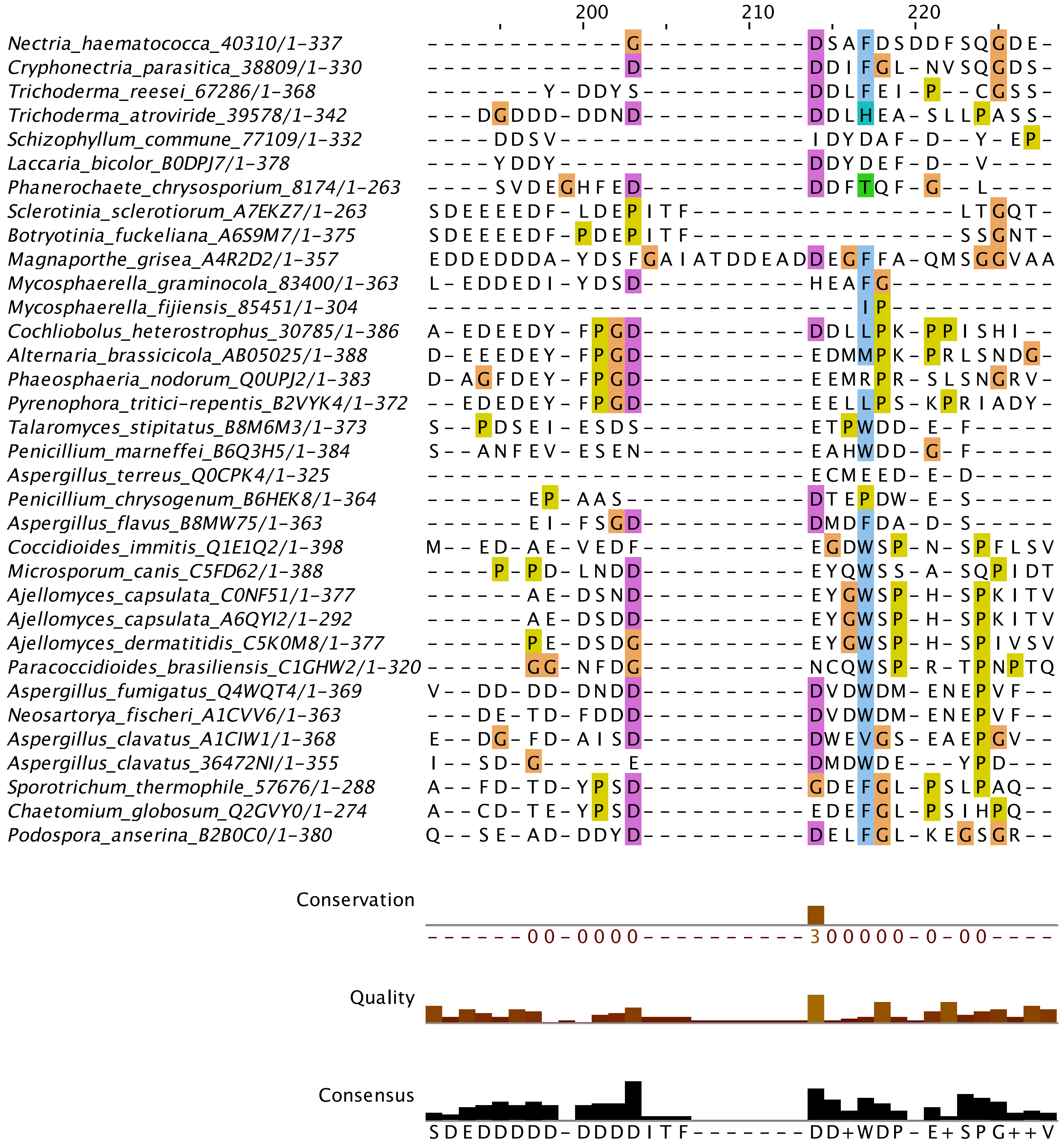
200
210
220
Nectria_haematococca_40310/1-337
Cryphonectria_parasitica_38809/1-330
Trichoderma_reesei_67286/1-368
Trichoderma_atroviride_39578/1-342
Schizophyllum_commune_77109/1-332
Laccaria_bicolor_B0DPJ7/1-378
Phanerochaete_chrysosporium_8174/1-263
Sclerotinia_sclerotiorum_A7EKZ7/1-263
Botryotinia_fuckeliana_A6S9M7/1-375
Magnaporthe_grisea_A4R2D2/1-357
Mycosphaerella_graminocola_83400/1-363
Mycosphaerella_fijiensis_85451/1-304
Cochliobolus_heterostrophus_30785/1-386
Alternaria_brassicicola_AB05025/1-388
Phaeosphaeria_nodorum_Q0UPJ2/1-383
Pyrenophora_tritici-repentis_B2VYK4/1-372
Talaromyces_stipitatus_B8M6M3/1-373
Penicillium_marneffei_B6Q3H5/1-384
Aspergillus_terreus_Q0CPK4/1-325
Penicillium_chrysogenum_B6HEK8/1-364
Aspergillus_flavus_B8MW75/1-363
Coccidioides_immitis_Q1E1Q2/1-398
Microsporum_canis_C5FD62/1-388
Ajellomyces_capsulata_C0NF51/1-377
Ajellomyces_capsulata_A6QYI2/1-292
Ajellomyces_dermatitidis_C5K0M8/1-377
Paracoccidioides_brasiliensis_C1GHW2/1-320
Aspergillus_fumigatus_Q4WQT4/1-369
Neosartorya_fischeri_A1CVV6/1-363
Aspergillus_clavatus_A1CIW1/1-368
Aspergillus_clavatus_36472NI/1-355
Sporotrichum_thermophile_57676/1-288
Chaetomium_globosum_Q2GVY0/1-274
Podospora_anserina_B2B0C0/1-380
Conservation
Quality
Consensus
SDEDDDDD-DDDDITF-------DD+WDP-E+SPG++V

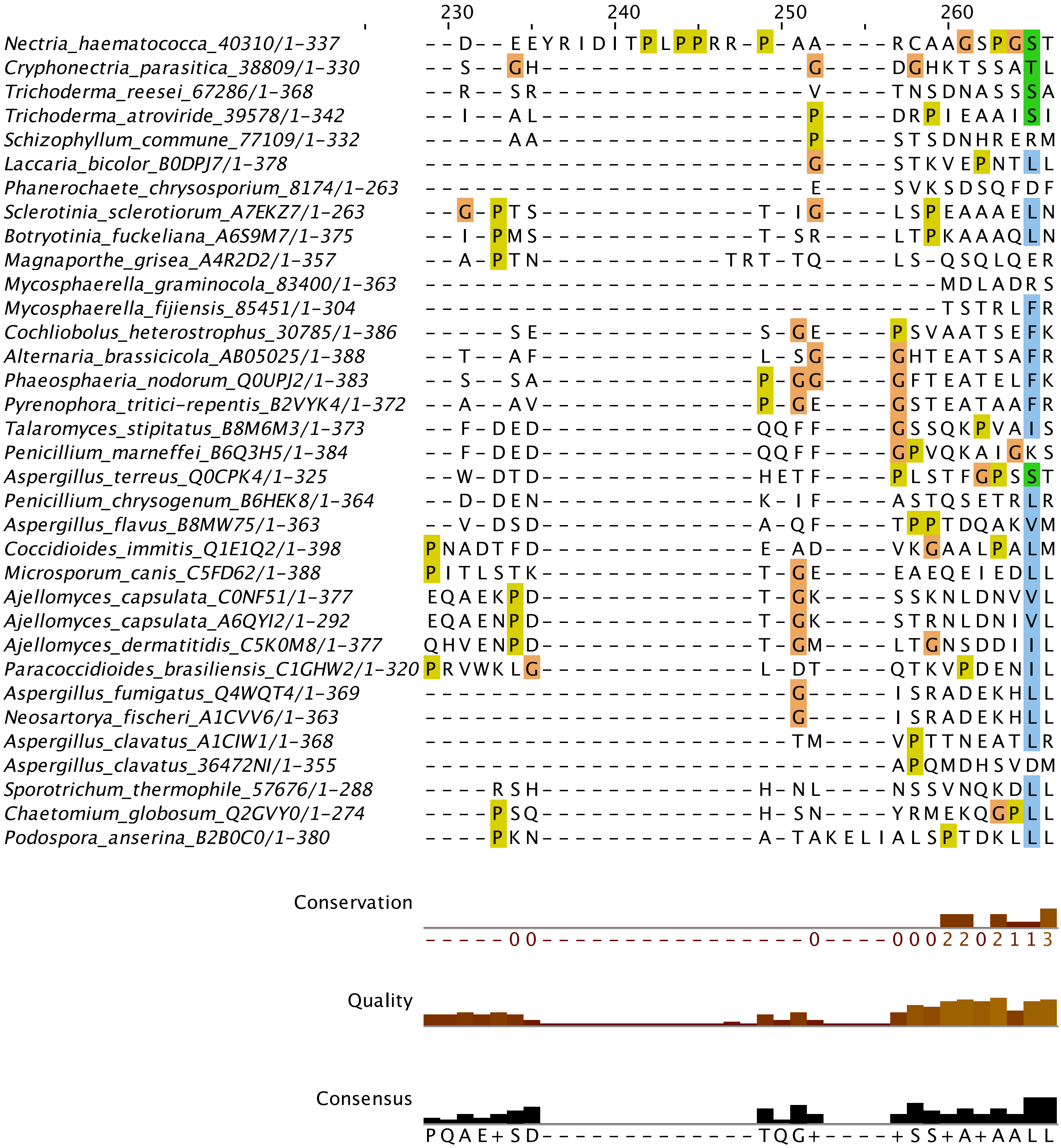

230
240
250
260
Nectria_haematococca_40310/1-337
Cryphonectria_parasitica_38809/1-330
Trichoderma_reesei_67286/1-368
Trichoderma_atroviride_39578/1-342
Schizophyllum_commune_77109/1-332
Laccaria_bicolor_B0DPJ7/1-378
Phanerochaete_chrysosporium_8174/1-263
Sclerotinia_sclerotiorum_A7EKZ7/1-263
Botryotinia_fuckeliana_A6S9M7/1-375
Magnaporthe_grisea_A4R2D2/1-357
Mycosphaerella_graminocola_83400/1-363
Mycosphaerella_fijiensis_85451/1-304
Cochliobolus_heterostrophus_30785/1-386
Alternaria_brassicicola_AB05025/1-388
Phaeosphaeria_nodorum_Q0UPJ2/1-383
Pyrenophora_tritici-repentis_B2VYK4/1-372
Talaromyces_stipitatus_B8M6M3/1-373
Penicillium_marneffei_B6Q3H5/1-384
Aspergillus_terreus_Q0CPK4/1-325
Penicillium_chrysogenum_B6HEK8/1-364
Aspergillus_flavus_B8MW75/1-363
Coccidioides_immitis_Q1E1Q2/1-398
Microsporum_canis_C5FD62/1-388
Ajellomyces_capsulata_C0NF51/1-377
Ajellomyces_capsulata_A6QYI2/1-292
Ajellomyces_dermatitidis_C5K0M8/1-377
Paracoccidioides_brasiliensis_C1GHW2/1-320
Aspergillus_fumigatus_Q4WQT4/1-369
Neosartorya_fischeri_A1CVV6/1-363
Aspergillus_clavatus_A1CIW1/1-368
Aspergillus_clavatus_36472NI/1-355
Sporotrichum_thermophile_57676/1-288
Chaetomium_globosum_Q2GVY0/1-274
Podospora_anserina_B2B0C0/1-380
Conservation
Quality
Consensus

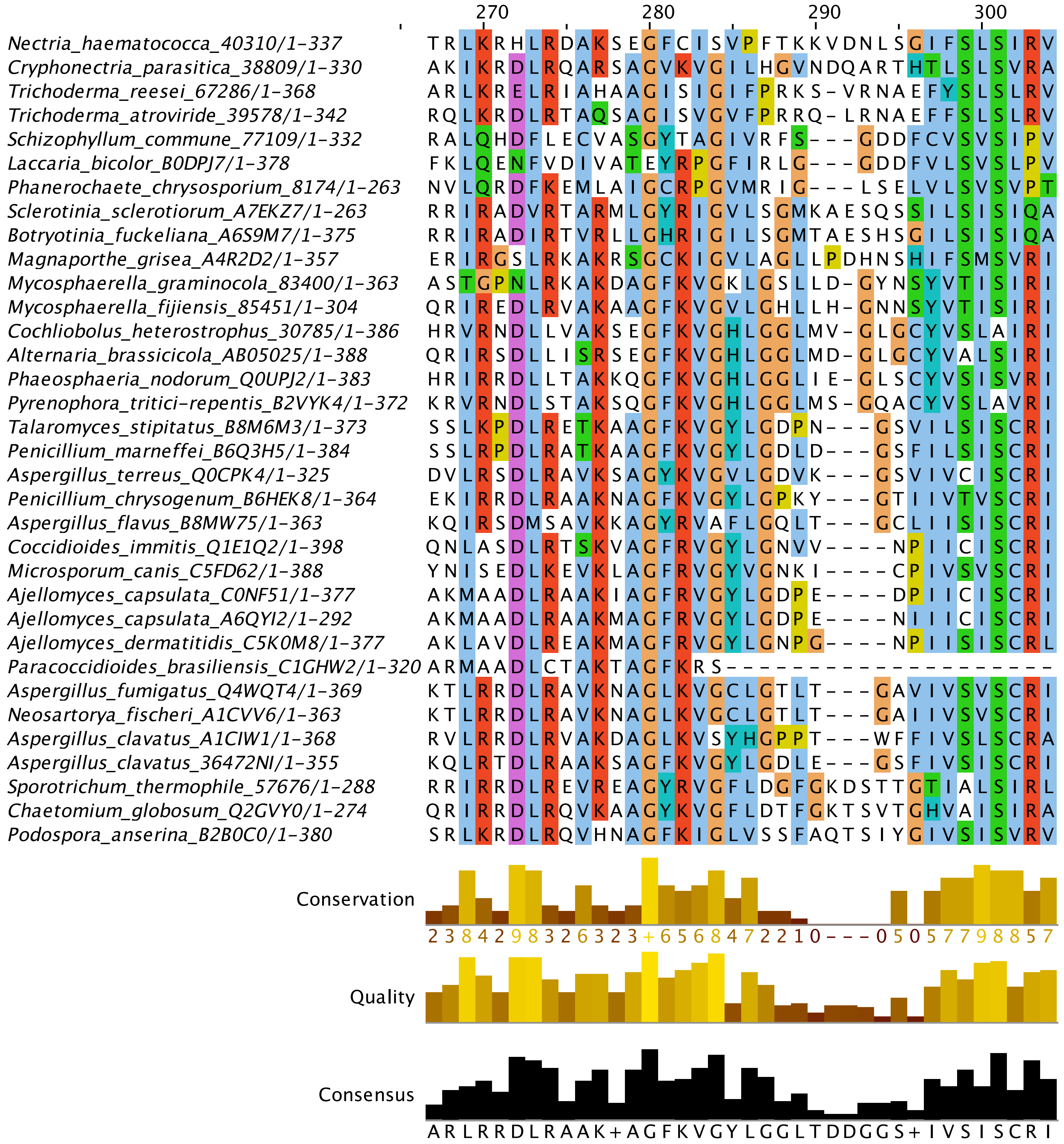
270 280 290 300
Nectria_haematococca_40310/1-337 TRLKRHLRDAKSEGFCISVPFTKKVDNLSGIFSLSIRV
Cryphonectria_parasitica_38809/1-330 AKIKRDLRQARSAGVKVGILHGVNDQARTHTLSLSVRA
Trichoderma_reesei_67286/1-368 ARLKRELRIAHAAGISIGIFPRKS-VRNAEFYSLSLRV
Trichoderma_atroviride_39578/1-342 RQLKRDLRTAQSAGISVGVFPRRQ-LRNAEFFSLSLRV
Schizophyllum_commune_77109/1-332 RALQHDFLECVASGYTAGIVRFS---GDDFCVSVSIPV
Laccaria_bicolor_B0DPJ7/1-378 FKLQENFVDIVATEYRPGFIRLG---GDDFVLSVSLPV
Phanerochaete_chrysosporium_8174/1-263 NVLQRDFKEMLAIGCRPGVMRIG---LSELVLSVSVPT
Sclerotinia_sclerotiorum_A7EKZ7/1-263 RRIRADVRTARMLGYRIGVLSGMKAESQSSILSISIQA
Botryotinia_fuckeliana_A6S9M7/1-375 RRIRADIRTVRLLGHRIGILSGMTAESHSGILSISIQA
Magnaporthe_grisea_A4R2D2/1-357 ERIRGSLRKAKRSGCKIGVLAGLLPDHNSHIFSMSVRI
Mycosphaerella_graminocola_83400/1-363 ASTGPNLRKAKDAGFKVGKLGSLLD-GYNSYVTISIRI
Mycosphaerella_fijiensis_85451/1-304 QRIREDLRVAKAAGFKVGVLGHLLH-GNNSYVTISIRI
Cochliobolus_heterostrophus_30785/1-386 HRVRNDLLVAKSEGFKVGHLGGLMV-GLGCYVSLAIRI
Alternaria_brassicicola_AB05025/1-388 QRIRSDLLISRSEGFKVGHLGGLMD-GLGCYVALSIRI
Phaeosphaeria_nodorum_Q0UPJ2/1-383 HRIRRDLLTAKKQGFKVGHLGGLIE-GLSCYVSISVRI
Pyrenophora_tritici-repentis_B2VYK4/1-372 KRVRNDLSTAKSQGFKVGHLGGLMS-GQACYVSLAVRI
Talaromyces_stipitatus_B8M6M3/1-373 SSLKPDLRETKAAGFKVGYLGDPN---GSVILSISCRI
Penicillium_marneffei_B6Q3H5/1-384 SSLRPDLRATKAAGFKVGYLGDLD---GSFILSISCRI
Aspergillus_terreus_Q0CPK4/1-325 DVLRSDLRAVKSAGYKVGVLGDVK---GSVIVCISCRI
Penicillium_chrysogenum_B6HEK8/1-364 EKIRRDLRAAKNAGFKVGYLGPKY---GTIIVTVSCRI
Aspergillus_flavus_B8MW75/1-363 KQIRSDMSAVKKAGYRVAFLGQLT---GCLIISISCRI
Coccidioides_immitis_Q1E1Q2/1-398 QNLASDLRTSKVAGFRVGYLGNVV----NPIICISCRI
Microsporum_canis_C5FD62/1-388 YNISEDLKEVKLAGFRVGYVGNKI----CPIVSVSCRI
Ajellomyces_capsulata_C0NF51/1-377 AKMAADLRAAKIAGFRVGYLGDPE----DPIICISCRI
Ajellomyces_capsulata_A6QYI2/1-292 AKMAADLRAAKMAGFRVGYLGDPE----NIIICISCRI
Ajellomyces_dermatitidis_C5K0M8/1-377 AKLAVDLREAKMAGFRVGYLGNPG----NPIISISCRL
Paracoccidioides_brasiliensis_C1GHW2/1-320 ARMAADLCTAKTAGFKRS--------------------
Aspergillus_fumigatus_Q4WQT4/1-369 KTLRRDLRAVKNAGLKVGCLGTLT---GAVIVSVSCRI
Neosartorya_fischeri_A1CVV6/1-363 KTLRRDLRAVKNAGLKVGCLGTLT---GAIIVSVSCRI
Aspergillus_clavatus_A1CIW1/1-368 RVLRRDLRVAKDAGLKVSYHGPPT---WFFIVSLSCRA
Aspergillus_clavatus_36472NI/1-355 KQLRTDLRAAKSAGFKVGYLGDLE---GSFIVSISCRI
Sporotrichum_thermophile_57676/1-288 RRIRRDLREVREAGYRVGFLDGFGKDSTTGTIALSIRL
Chaetomium_globosum_Q2GVY0/1-274 QRIRRDLRQVKAAGYKVGFLDTFGKTSVTGHVAISIRA
Podospora_anserina_B2B0C0/1-380 SRLKRDLRQVHNAGFKIGLVSSFAQTSIYGIVSISVRV
Conservation
2 3 8 4 2 9 8 3 2 6 3 2 3 + 6 5 6 8 4 7 2 2 1 0 - - - 0 5 0 5 7 7 9 8 8 5 7
Quality
Consensus
ARLRRDLRAAK+AGFKVGYLGGLTDDGGS+IVSISCRI

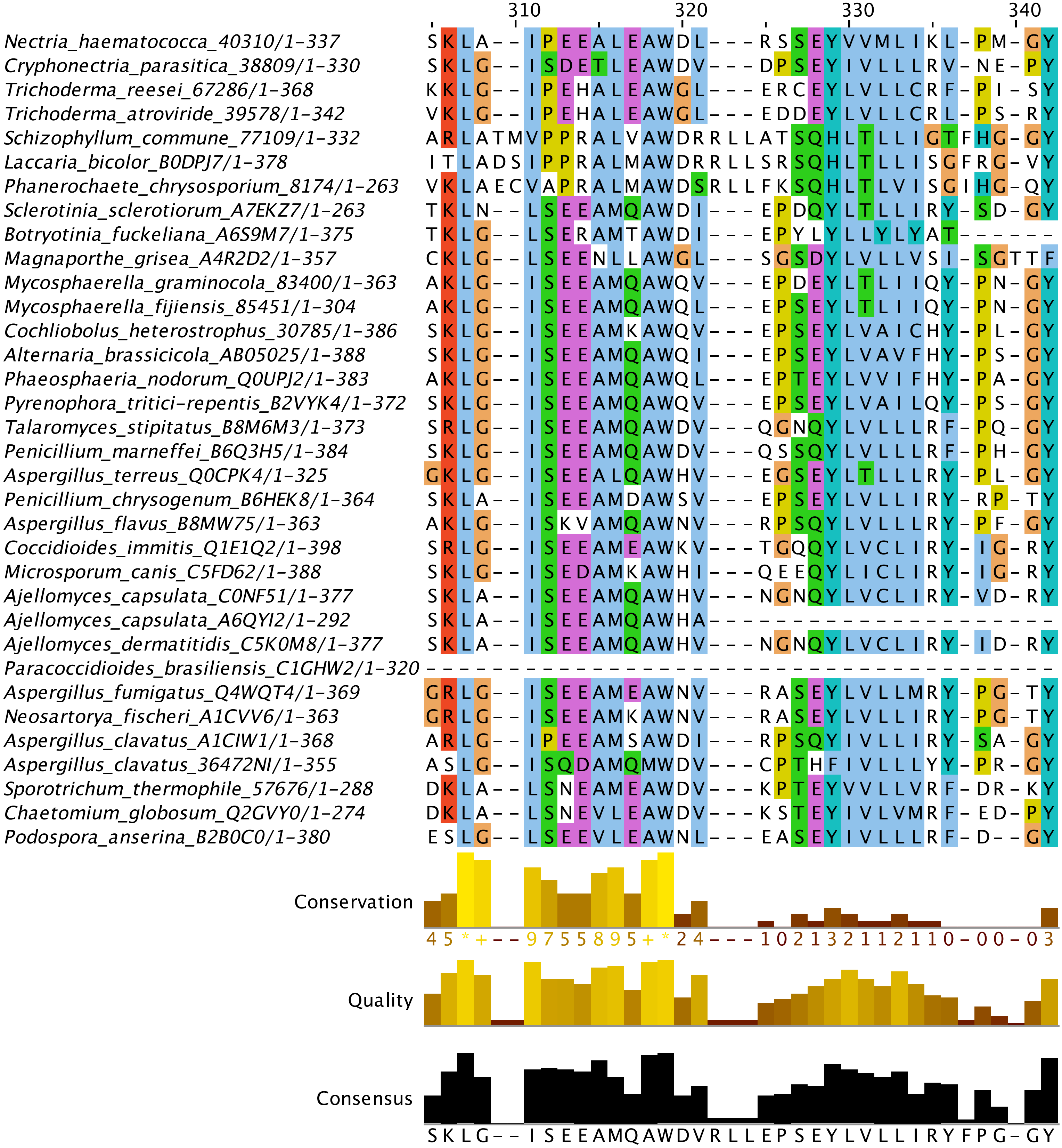
310
320
330
340
Nectria_haematococca_40310/1-337 SKLA--IPEEALEAWDL---RSSEYVVMLIKL-PM-GY
Cryphonectria_parasitica_38809/1-330 SKLG--ISDETLEAWDV---DPSEYIVLLLRV-NE-PY
Trichoderma_reesei_67286/1-368 KKLG--IPEHALEAWGL---ERCEYLVLLCRF-PI-SY
Trichoderma_atroviride_39578/1-342 VKLG--IPEHALEAWGL---EDDEYLVLLCRL-PS-RY
Schizophyllum_commune_77109/1-332 ARLATMVPPRALVAWDRRLLATSQHLTLLIGTFHG-GY
Laccaria_bicolor_B0DPJ7/1-378 ITLADSIPPRALMAWDRRLLSRSQHLTLLISGFRG-VY
Phanerochaete_chrysosporium_8174/1-263 VKLAECVAPRALMAWDSRLLFKSQHLTLVISGIHG-QY
Sclerotinia_sclerotiorum_A7EKZ7/1-263 TKLN--LSEEAMQAWDI---EPDQYLTLLIRY-SD-GY
Botryotinia_fuckeliana_A6S9M7/1-375 TKLG--LSERAMTAWDI---EPYLYLLYLYAT------
Magnaporthe_grisea_A4R2D2/1-357 CKLG--LSEENLLAWGL---SGSDYLVLLVSI-SGTTF
Mycosphaerella_graminocola_83400/1-363 AKLG--ISEEAMQAWQV---EPDEYLTLIIQY-PN-GY
Mycosphaerella_fijiensis_85451/1-304 AKLG--ISEEAMQAWQL---EPSEYLTLIIQY-PN-GY
Cochliobolus_heterostrophus_30785/1-386 SKLG--ISEEAMKAWQV---EPSEYLVAICHY-PL-GY
Alternaria_brassicicola_AB05025/1-388 SKLG--ISEEAMQAWQI---EPSEYLVAVFHY-PS-GY
Phaeosphaeria_nodorum_Q0UPJ2/1-383 AKLG--ISEEAMQAWQL---EPTEYLVVIFHY-PA-GY
Pyrenophora_tritici-repentis_B2VYK4/1-372 SKLG--ISEEAMQAWQV---EPSEYLVAILQY-PS-GY
Talaromyces_stipitatus_B8M6M3/1-373 SRLG--ISEEAMQAWDV---QGNQYLVLLLRF-PQ-GY
Penicillium_marneffei_B6Q3H5/1-384 SKLG--ISEEAMQAWDV---QSSQYLVLLLRF-PH-GY
Aspergillus_terreus_Q0CPK4/1-325 GKLG--ISEEALQAWHV---EGSEYLTLLLRY-PL-GY
Penicillium_chrysogenum_B6HEK8/1-364 SKLA--ISEEAMDAWSV---EPSEYLVLLIRY-RP-TY
Aspergillus_flavus_B8MW75/1-363 AKLG--ISKVAMQAWNV---RPSQYLVLLLRY-PF-GY
Coccidioides_immitis_Q1E1Q2/1-398 SRLG--ISEEAMEAWKV---TGQQYLVCLIRY-IG-RY
Microsporum_canis_C5FD62/1-388 SKLG--ISEDAMKAWHI---QEEQYLICLIRY-IG-RY
Ajellomyces_capsulata_C0NF51/1-377 SKLA--ISEEAMQAWHV---NGNQYLVCLIRY-VD-RY
Ajellomyces_capsulata_A6QYI2/1-292 SKLA--ISEEAMQAWHA---------------------
Ajellomyces_dermatitidis_C5K0M8/1-377 SKLA--ISEEAMQAWHV---NGNQYLVCLIRY-ID-RY
Paracoccidioides_brasiliensis_C1GHW2/1-320 --------------------------------------
Aspergillus_fumigatus_Q4WQT4/1-369 GRLG--ISEEAMEAWNV---RASEYLVLLMRY-PG-TY
Neosartorya_fischeri_A1CVV6/1-363 GRLG--ISEEAMKAWNV---RASEYLVLLIRY-PG-TY
Aspergillus_clavatus_A1CIW1/1-368 ARLG--IPEEAMSAWDI---RPSQYIVLLIRY-SA-GY
Aspergillus_clavatus_36472NI/1-355 ASLG--ISQDAMQMWDV---CPTHFIVLLLYY-PR-GY
Sporotrichum_thermophile_57676/1-288 DKLA--LSNEAMEAWDV---KPTEYVVLLVRF-DR-KY
Chaetomium_globosum_Q2GVY0/1-274 DKLA--LSNEVLEAWDV---KSTEYIVLVMRF-ED-PY
Podospora_anserina_B2B0C0/1-380 ESLG--LSEEVLEAWNL---EASEYIVLLLRF-D--GY
Conservation
45*+--9755895+*24---10213211211 0-00-03
Quality
Consensus
SKLG--ISEEAMQAWDVRLLEPSEYLVLLIRYFPG-GY

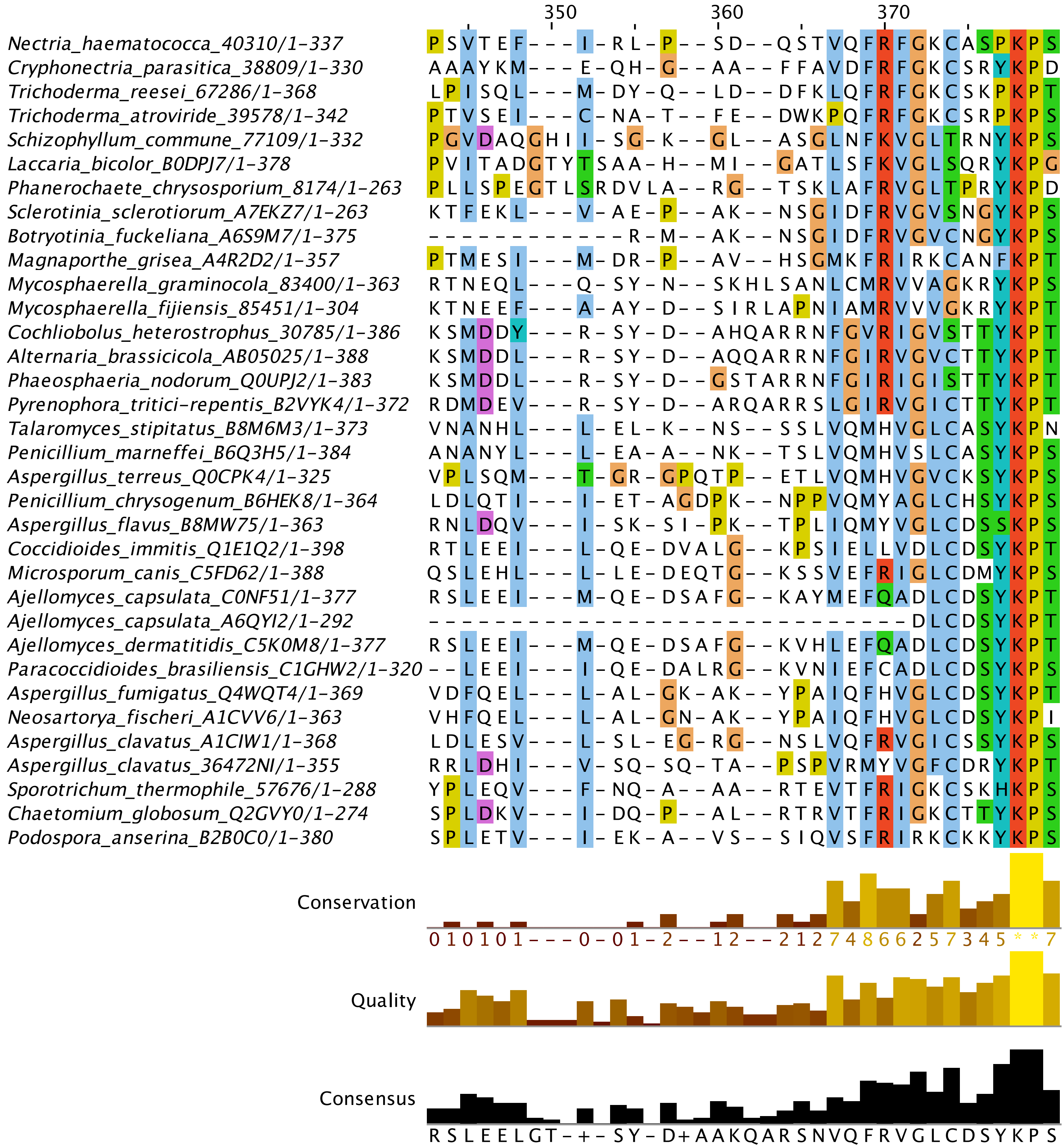

350
360
370
Nectria_haematococca_40310/1-337 PSVTEF---I-RL-P--SD--QSTVQFRFGKCASPKPS
Cryphonectria_parasitica_38809/1-330 AAAYKM---E-QH-G--AA--FFAVDFRFGKCSRYKPD
Trichoderma_reesei_67286/1-368 LPISQL---M-DY-Q--LD--DFKLQFRFGKCSKPKPT
Trichoderma_atroviride_39578/1-342 PTVSEI---C-NA-T--FE--DWKPQFRFGKCSRPKPS
Schizophyllum_commune_77109/1-332 PGVDAQGHII-SG-K--GL--ASGLNFKVGLTRNYKPS
Laccaria_bicolor_B0DPJ7/1-378 PVITADGTYTSAA-H--MI--GATLSFKVGLSQRYKPG
Phanerochaete_chrysosporium_8174/1-263 PLLSPEGTLSRDVLA--RG--TSKLAFRVGLTPRYKPD
Sclerotinia_sclerotiorum_A7EKZ7/1-263 KTFEKL---V-AE-P--AK--NSGIDFRVGVSNGYKPS
Botryotinia_fuckeliana_A6S9M7/1-375 ------------R-M--AK--NSGIDFRVGVCNGYKPS
Magnaporthe_grisea_A4R2D2/1-357 PTMESI---M-DR-P--AV--HSGMKFRIRKCANFKPT
Mycosphaerella_graminocola_83400/1-363 RTNEQL---Q-SY-N--SKHLSANLCMRVVAGKRYKPS
Mycosphaerella_fijiensis_85451/1-304 KTNEEF---A-AY-D--SIRLAPNIAMRVVVGKRYKPT
Cochliobolus_heterostrophus_30785/1-386 KSMDDY---R-SY-D--AHQARRNFGVRIGVSTTYKPT
Alternaria_brassicicola_AB05025/1-388 KSMDDL---R-SY-D--AQQARRNFGIRVGVCTTYKPT
Phaeosphaeria_nodorum_Q0UPJ2/1-383 KSMDDL---R-SY-D--GSTARRNFGIRIGISTTYKPT
Pyrenophora_tritici-repentis_B2VYK4/1-372 RDMDEV---R-SY-D--ARQARRSLGIRVGICTTYKPT
Talaromyces_stipitatus_B8M6M3/1-373 VNANHL---L-EL-K--NS--SSLVQMHVGLCASYKPN
Penicillium_marneffei_B6Q3H5/1-384 ANANYL---L-EA-A--NK--TSLVQMHVSLCASYKPS
Aspergillus_terreus_Q0CPK4/1-325 VPLSQM---T-GR-GPQTP--ETLVQMHVGVCKSYKPS
Penicillium_chrysogenum_B6HEK8/1-364 LDLQTI---I-ET-AGDPK--NPPVQMYAGLCHSYKPS
Aspergillus_flavus_B8MW75/1-363 RNLDQV---I-SK-SI-PK--TPLIQMYVGLCDSSKPS
Coccidioides_immitis_Q1E1Q2/1-398 RTLEEI---L-QE-DVALG--KPSIELLVDLCDSYKPT
Microsporum_canis_C5FD62/1-388 QSLEHL---L-LE-DEQTG--KSSVEFRIGLCDMYKPS
Ajellomyces_capsulata_C0NF51/1-377 RSLEEI---M-QE-DSAFG--KAYMEFQADLCDSYKPT
Ajellomyces_capsulata_A6QYI2/1-292 -----------------------------DLCDSYKPT
Ajellomyces_dermatitidis_C5K0M8/1-377 RSLEEI---M-QE-DSAFG--KVHLEFQADLCDSYKPT
Paracoccidioides_brasiliensis_C1GHW2/1-320 --LEEI---I-QE-DALRG--KVNIEFCADLCDSYKPS
Aspergillus_fumigatus_Q4WQT4/1-369 VDFQEL---L-AL-GK-AK--YPAIQFHVGLCDSYKPT
Neosartorya_fischeri_A1CVV6/1-363 VHFQEL---L-AL-GN-AK--YPAIQFHVGLCDSYKPI
Aspergillus_clavatus_A1CIW1/1-368 LDLESV---L-SL-EG-RG--NSLVQFRVGICSSYKPS
Aspergillus_clavatus_36472NI/1-355 RRLDHI---V-SQ-SQ-TA--PSPVRMYVGFCDRYKPT
Sporotrichum_thermophile_57676/1-288 YPLEQV---F-NQ-A--AA--RTEVTFRIGKCSKHKPS
Chaetomium_globosum_Q2GVY0/1-274 SPLDKV---I-DQ-P--AL--RTRVTFRIGKCTTYKPS
Podospora_anserina_B2B0C0/1-380 SPLETV---I-EK-A--VS--SIQVSFRIRKCKKYKPS
Conservation
010101---0-01-2--12--21274866257345**7
Quality
Consensus
RSLEELGT-+-SY-D+AAKQARSNVQFRVGLCDSYKPS

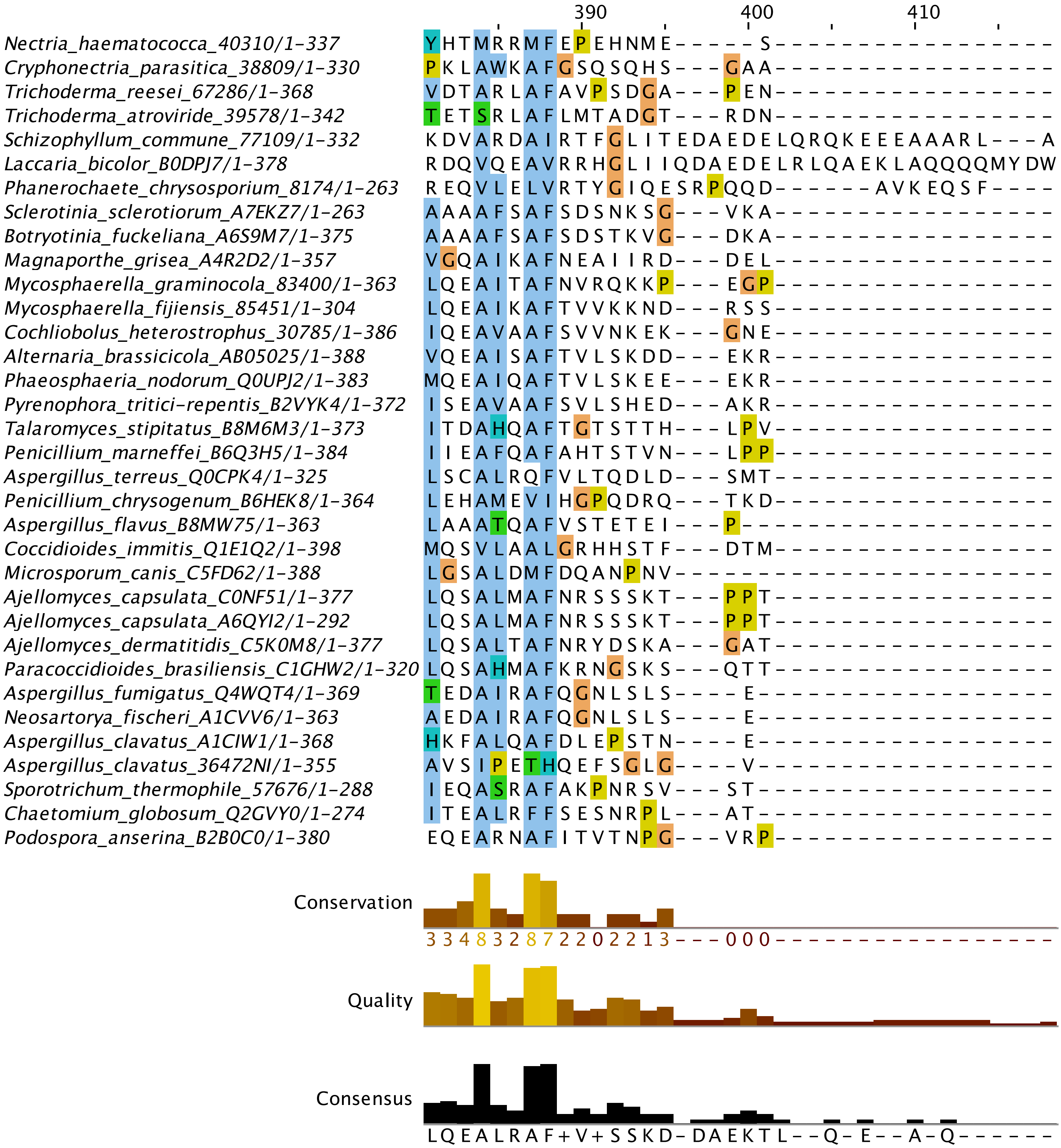

390
400
410
Nectria_haematococca_40310/1-337 YHTMRRMFEPEHNME-----S-----------------
Cryphonectria_parasitica_38809/1-330 PKLAWKAFGSQSQHS---GAA-----------------
Trichoderma_reesei_67286/1-368 VDTARLAFAVPSDGA---PEN-----------------
Trichoderma_atroviride_39578/1-342 TETSRLAFLMTADGT---RDN-----------------
Schizophyllum_commune_77109/1-332 KDVARDAIRTFGLITEDAEDELQRQKEEEAAARL---A
Laccaria_bicolor_B0DPJ7/1-378 RDQVQEAVRRHGLIIQDAEDELRLQAEKLAQQQQMYDW
Phanerochaete_chrysosporium_8174/1-263 REQVLELVRTYGIQESRPQQD------AVKEQSF----
Sclerotinia_sclerotiorum_A7EKZ7/1-263 AAAAFSAFSDSNKSG---VKA-----------------
Botryotinia_fuckeliana_A6S9M7/1-375 AAAAFSAFSDSTKVG---DKA-----------------
Magnaporthe_grisea_A4R2D2/1-357 VGQAIKAFNEAIIRD---DEL-----------------
Mycosphaerella_graminocola_83400/1-363 LQEAITAFNVRQKKP---EGP-----------------
Mycosphaerella_fijiensis_85451/1-304 LQEAIKAFTVVKKND---RSS-----------------
Cochliobolus_heterostrophus_30785/1-386 IQEAVAAFSVVNKEK---GNE-----------------
Alternaria_brassicicola_AB05025/1-388 VQEAISAFTVLSKDD---EKR-----------------
Phaeosphaeria_nodorum_Q0UPJ2/1-383 MQEAIQAFTVLSKEE---EKR-----------------
Pyrenophora_tritici-repentis_B2VYK4/1-372 ISEAVAAFSVLSHED---AKR-----------------
Talaromyces_stipitatus_B8M6M3/1-373 ITDAHQAFTGTSTTH---LPV-----------------
Penicillium_marneffei_B6Q3H5/1-384 IIEAFQAFAHTSTVN---LPP-----------------
Aspergillus_terreus_Q0CPK4/1-325 LSCALRQFVLTQDLD---SMT-----------------
Penicillium_chrysogenum_B6HEK8/1-364 LEHAMEVIHGPQDRQ---TKD-----------------
Aspergillus_flavus_B8MW75/1-363 LAAATQAFVSTETEI---P-------------------
Coccidioides_immitis_Q1E1Q2/1-398 MQSVLAALGRHHSTF---DTM-----------------
Microsporum_canis_C5FD62/1-388 LGSALDMFDQANPNV-----------------------
Ajellomyces_capsulata_C0NF51/1-377 LQSALMAFNRSSSKT---PPT-----------------
Ajellomyces_capsulata_A6QYI2/1-292 LQSALMAFNRSSSKT---PPT-----------------
Ajellomyces_dermatitidis_C5K0M8/1-377 LQSALTAFNRYDSKA---GAT-----------------
Paracoccidioides_brasiliensis_C1GHW2/1-320 LQSAHMAFKRNGSKS---QTT-----------------
Aspergillus_fumigatus_Q4WQT4/1-369 TEDAIRAFQGNLSLS----E------------------
Neosartorya_fischeri_A1CVV6/1-363 AEDAIRAFQGNLSLS----E------------------
Aspergillus_clavatus_A1CIW1/1-368 HKFALQAFDLEPSTN----E------------------
Aspergillus_clavatus_36472NI/1-355 AVSIPETHQEFSGLG----V------------------
Sporotrichum_thermophile_57676/1-288 IEQASRAFAKPNRSV---ST------------------
Chaetomium_globosum_Q2GVY0/1-274 ITEALRFFSESNRPL---AT------------------
Podospora_anserina_B2B0C0/1-380 EQEARNAFITVTNPG---VRP-----------------
Conservation
334832872202213---000-----------------
Quality
Consensus
LQEALRAF+V+SSKD-DAEKTL--Q-E--A-Q------

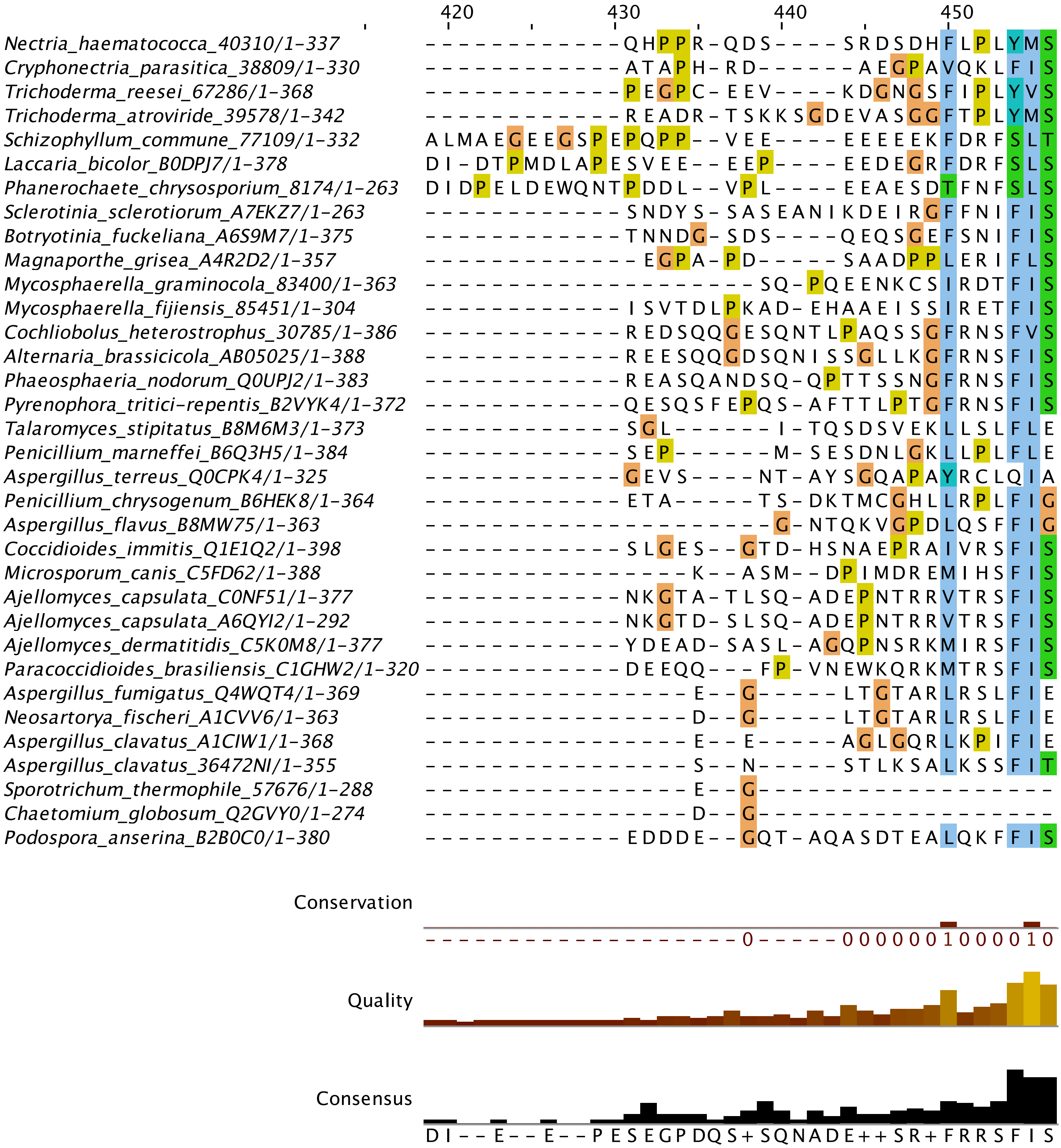
420
430
440
450
Nectria_haematococca_40310/1-337
Cryphonectria_parasitica_38809/1-330
Trichoderma_reesei_67286/1-368
Trichoderma_atroviride_39578/1-342
Schizophyllum_commune_77109/1-332
Laccaria_bicolor_B0DPJ7/1-378
Phanerochaete_chrysosporium_8174/1-263
Sclerotinia_sclerotiorum_A7EKZ7/1-263
Botryotinia_fuckeliana_A6S9M7/1-375
Magnaporthe_grisea_A4R2D2/1-357
Mycosphaerella_graminocola_83400/1-363
Mycosphaerella_fijiensis_85451/1-304
Cochliobolus_heterostrophus_30785/1-386
Alternaria_brassicicola_AB05025/1-388
Phaeosphaeria_nodorum_Q0UPJ2/1-383
Pyrenophora_tritici-repentis_B2VYK4/1-372
Talaromyces_stipitatus_B8M6M3/1-373
Penicillium_marneffei_B6Q3H5/1-384
Aspergillus_terreus_Q0CPK4/1-325
Penicillium_chrysogenum_B6HEK8/1-364
Aspergillus_flavus_B8MW75/1-363
Coccidioides_immitis_Q1E1Q2/1-398
Microsporum_canis_C5FD62/1-388
Ajellomyces_capsulata_C0NF51/1-377
Ajellomyces_capsulata_A6QYI2/1-292
Ajellomyces_dermatitidis_C5K0M8/1-377
Paracoccidioides_brasiliensis_C1GHW2/1-320
Aspergillus_fumigatus_Q4WQT4/1-369
Neosartorya_fischeri_A1CVV6/1-363
Aspergillus_clavatus_A1CIW1/1-368
Aspergillus_clavatus_36472NI/1-355
Sporotrichum_thermophile_57676/1-288
Chaetomium_globosum_Q2GVY0/1-274
Podospora_anserina_B2B0C0/1-380
Conservation
Quality
Consensus

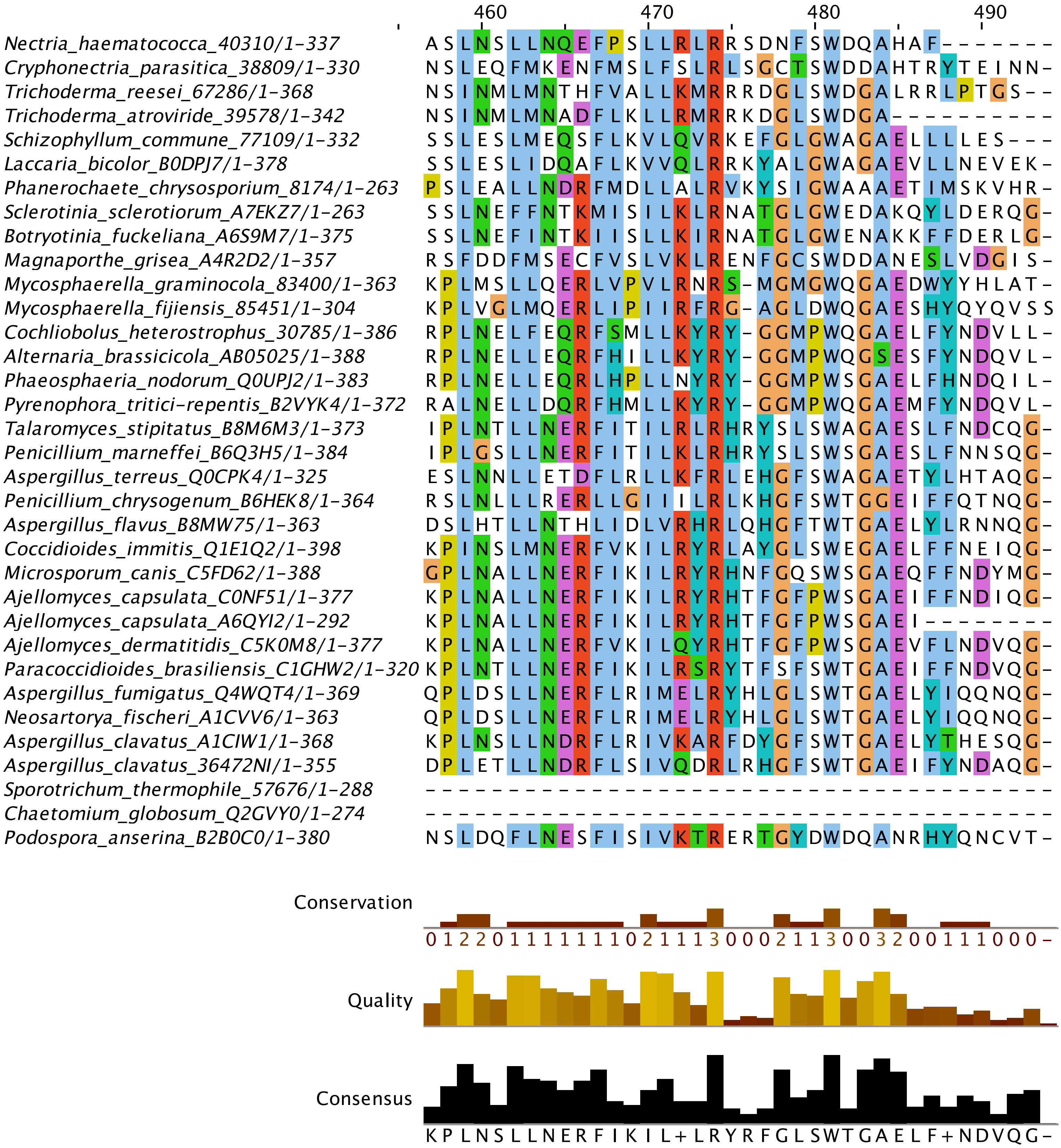

460
470
480
490
Nectria_haematococca_40310/1-337
Cryphonectria_parasitica_38809/1-330
Trichoderma_reesei_67286/1-368
Trichoderma_atroviride_39578/1-342
Schizophyllum_commune_77109/1-332
Laccaria_bicolor_B0DPJ7/1-378
Phanerochaete_chrysosporium_8174/1-263
Sclerotinia_sclerotiorum_A7EKZ7/1-263
Botryotinia_fuckeliana_A6S9M7/1-375
Magnaporthe_grisea_A4R2D2/1-357
Mycosphaerella_graminocola_83400/1-363
Mycosphaerella_fijiensis_85451/1-304
Cochliobolus_heterostrophus_30785/1-386
Alternaria_brassicicola_AB05025/1-388
Phaeosphaeria_nodorum_Q0UPJ2/1-383
Pyrenophora_tritici-repentis_B2VYK4/1-372
Talaromyces_stipitatus_B8M6M3/1-373
Penicillium_marneffei_B6Q3H5/1-384
Aspergillus_terreus_Q0CPK4/1-325
Penicillium_chrysogenum_B6HEK8/1-364
Aspergillus_flavus_B8MW75/1-363
Coccidioides_immitis_Q1E1Q2/1-398
Microsporum_canis_C5FD62/1-388
Ajellomyces_capsulata_C0NF51/1-377
Ajellomyces_capsulata_A6QYI2/1-292
Ajellomyces_dermatitidis_C5K0M8/1-377
Paracoccidioides_brasiliensis_C1GHW2/1-320
Aspergillus_fumigatus_Q4WQT4/1-369
Neosartorya_fischeri_A1CVV6/1-363
Aspergillus_clavatus_A1CIW1/1-368
Aspergillus_clavatus_36472NI/1-355
Sporotrichum_thermophile_57676/1-288
Chaetomium_globosum_Q2GVY0/1-274
Podospora_anserina_B2B0C0/1-380
Conservation
0 1 2 2 0 1 1 1 1 1 1 1 0 2 1 1 1 3 0 0 0 2 1 1 3 0 0 3 2 0 0 1 1 1 0 0 0 -
Quality
Consensus
K P L N S L L N E R F I K I L + L R Y R F G L S W T G A E L F + N D V Q G -

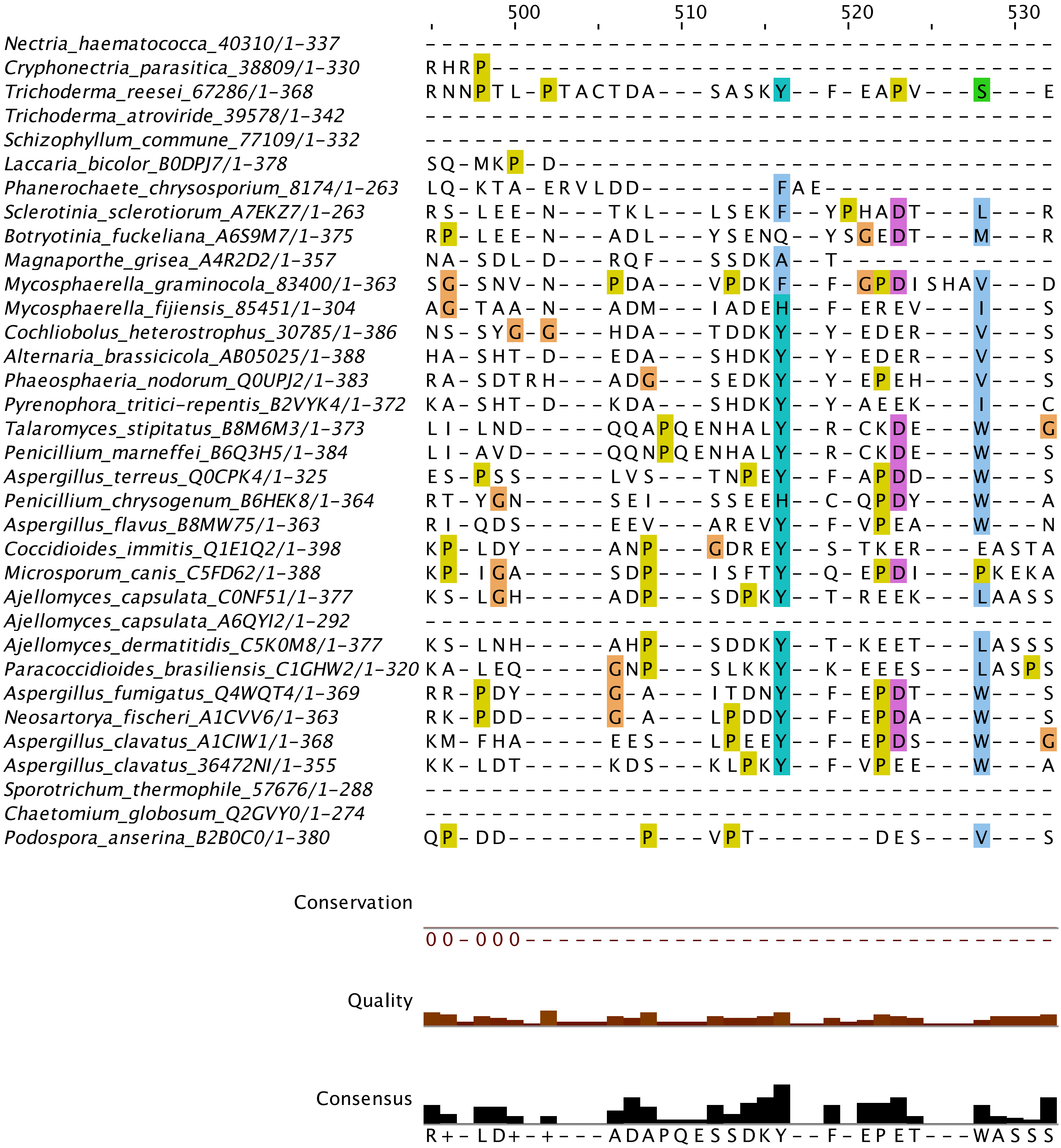
500
510
520
530
Nectria_haematococca_40310/1-337
Cryphonectria_parasitica_38809/1-330
Trichoderma_reesei_67286/1-368
Trichoderma_atroviride_39578/1-342
Schizophyllum_commune_77109/1-332
Laccaria_bicolor_B0DPJ7/1-378
Phanerochaete_chrysosporium_8174/1-263
Sclerotinia_sclerotiorum_A7EKZ7/1-263
Botryotinia_fuckeliana_A6S9M7/1-375
Magnaporthe_grisea_A4R2D2/1-357
Mycosphaerella_graminocola_83400/1-363
Mycosphaerella_fijiensis_85451/1-304
Cochliobolus_heterostrophus_30785/1-386
Alternaria_brassicicola_AB05025/1-388
Phaeosphaeria_nodorum_Q0UPJ2/1-383
Pyrenophora_tritici-repentis_B2VYK4/1-372
Talaromyces_stipitatus_B8M6M3/1-373
Penicillium_marneffei_B6Q3H5/1-384
Aspergillus_terreus_Q0CPK4/1-325
Penicillium_chrysogenum_B6HEK8/1-364
Aspergillus_flavus_B8MW75/1-363
Coccidioides_immitis_Q1E1Q2/1-398
Microsporum_canis_C5FD62/1-388
Ajellomyces_capsulata_C0NF51/1-377
Ajellomyces_capsulata_A6QYI2/1-292
Ajellomyces_dermatitidis_C5K0M8/1-377
Paracoccidioides_brasiliensis_C1GHW2/1-320
Aspergillus_fumigatus_Q4WQT4/1-369
Neosartorya_fischeri_A1CVV6/1-363
Aspergillus_clavatus_A1CIW1/1-368
Aspergillus_clavatus_36472NI/1-355
Sporotrichum_thermophile_57676/1-288
Chaetomium_globosum_Q2GVY0/1-274
Podospora_anserina_B2B0C0/1-380
Conservation
Quality
Consensus
R+-LD+-+---ADAPQESSDKY--F-EPET---WASSS

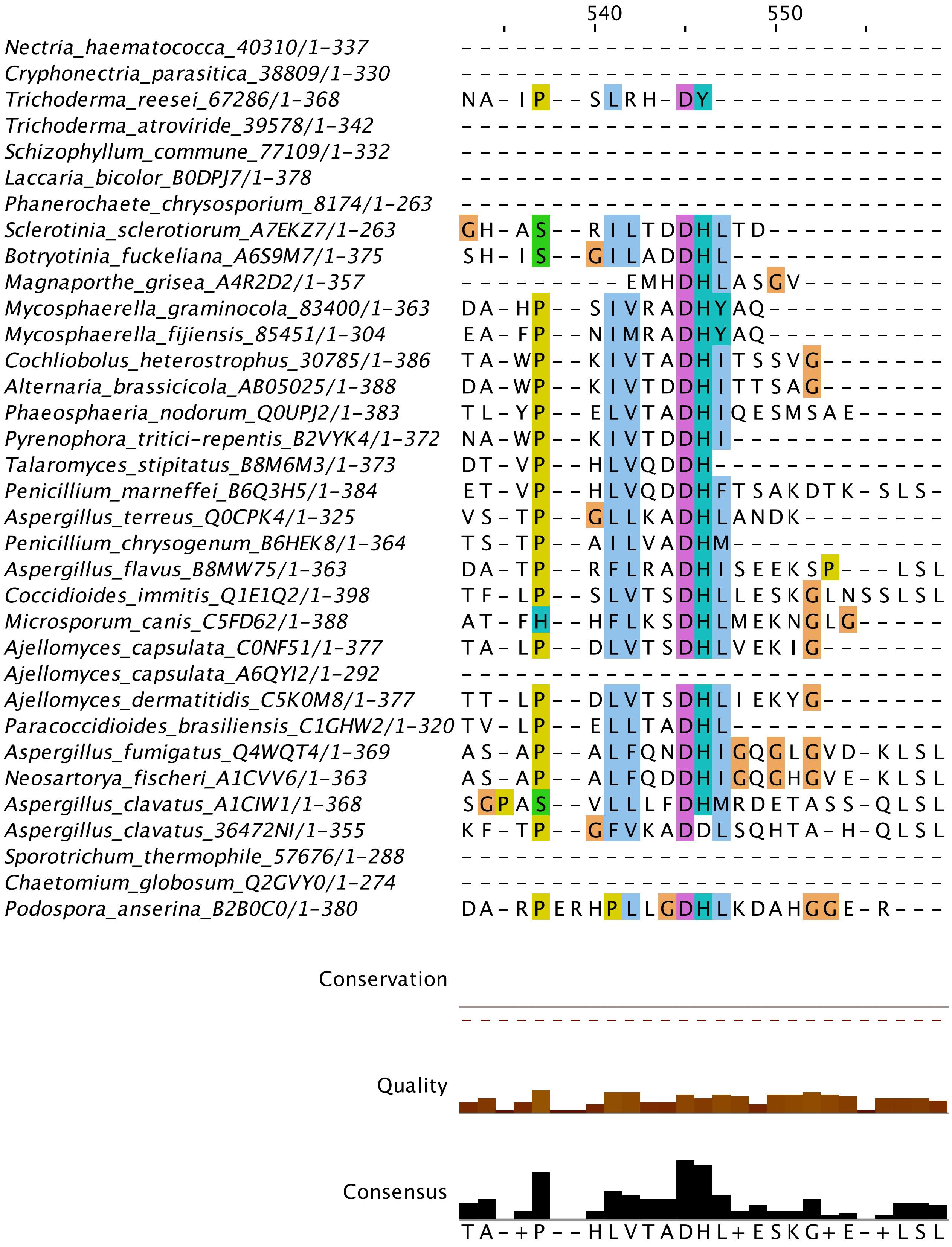
540
550
Nectria_haematococca_40310/1-337 ---------------------------
Cryphonectria_parasitica_38809/1-330 ---------------------------
Trichoderma_reesei_67286/1-368 NA-IP--SLRH-DY-------------
Trichoderma_atroviride_39578/1-342 ---------------------------
Schizophyllum_commune_77109/1-332 ---------------------------
Laccaria_bicolor_B0DPJ7/1-378 ---------------------------
Phanerochaete_chrysosporium_8174/1-263 ---------------------------
Sclerotinia_sclerotiorum_A7EKZ7/1-263 GH-AS--RILTDDHLTD----------
Botryotinia_fuckeliana_A6S9M7/1-375 SH-IS--GILADDHL------------
Magnaporthe_grisea_A4R2D2/1-357 ---------EMHDHLASGV--------
Mycosphaerella_graminocola_83400/1-363 DA-HP--SIVRADHYAQ----------
Mycosphaerella_fijiensis_85451/1-304 EA-FP--NIMRADHYAQ----------
Cochliobolus_heterostrophus_30785/1-386 TA-WP--KIVTADHITSSVG-------
Alternaria_brassicicola_AB05025/1-388 DA-WP--KIVTDDHITTSAG-------
Phaeosphaeria_nodorum_Q0UPJ2/1-383 TL-YP--ELVTADHIQESMSAE-----
Pyrenophora_tritici-repentis_B2VYK4/1-372 NA-WP--KIVTDDHI------------
Talaromyces_stipitatus_B8M6M3/1-373 DT-VP--HLVQDDH-------------
Penicillium_marneffei_B6Q3H5/1-384 ET-VP--HLVQDDHFTSAKDTK-SLS-
Aspergillus_terreus_Q0CPK4/1-325 VS-TP--GLLKADHLANDK--------
Penicillium_chrysogenum_B6HEK8/1-364 TS-TP--AILVADHM------------
Aspergillus_flavus_B8MW75/1-363 DA-TP--RFLRADHISEEKSP---LSL
Coccidioides_immitis_Q1E1Q2/1-398 TF-LP--SLVTSDHLLESKGLNSSLSL
Microsporum_canis_C5FD62/1-388 AT-FH--HFLKSDHLMEKNGLG-----
Ajellomyces_capsulata_C0NF51/1-377 TA-LP--DLVTSDHLVEKIG-------
Ajellomyces_capsulata_A6QYI2/1-292 ---------------------------
Ajellomyces_dermatitidis_C5K0M8/1-377 TT-LP--DLVTSDHLIEKYG-------
Paracoccidioides_brasiliensis_C1GHW2/1-320 TV-LP--ELLTADHL------------
Aspergillus_fumigatus_Q4WQT4/1-369 AS-AP--ALFQNDHIGQGLGVD-KLSL
Neosartorya_fischeri_A1CVV6/1-363 AS-AP--ALFQDDHIGQGHGVE-KLSL
Aspergillus_clavatus_A1CIW1/1-368 SGPAS--VLLLFDHMRDETASS-QLSL
Aspergillus_clavatus_36472NI/1-355 KF-TP--GFVKADDLSQHTA-H-QLSL
Sporotrichum_thermophile_57676/1-288 ---------------------------
Chaetomium_globosum_Q2GVY0/1-274 ---------------------------
Podospora_anserina_B2B0C0/1-380 DA-RPERHPLLGDHLKDAHGGE-R---
Conservation
Quality
Consensus
TA-+P--HLVTADHL+ESKG+E-+LSL
