# Supplementary material for: Evolutionary history of the poly(ADP-ribose) polymerase gene family in eukaryotes
Source: BMC Evol Biol. 2010 Oct 13;10:308. doi: 10.1186/1471-2148-10-308 (PMC2964712; doi:10.1186/1471-2148-10-308)
Supplement: Additional file 7 — Multiple alignment of the PARP catalytic domains of Clade 2 PARP proteins annotated with structural predictions. These alignments only show the conserved PARP catalytic domain. The structural elements predicted to be present in Arabidopsis thaliana RCD1 by Phyre are shown at the bottom of the alignment [89]. Annotations as in Additional file 5. [file 1471-2148-10-308-S7.PDF]

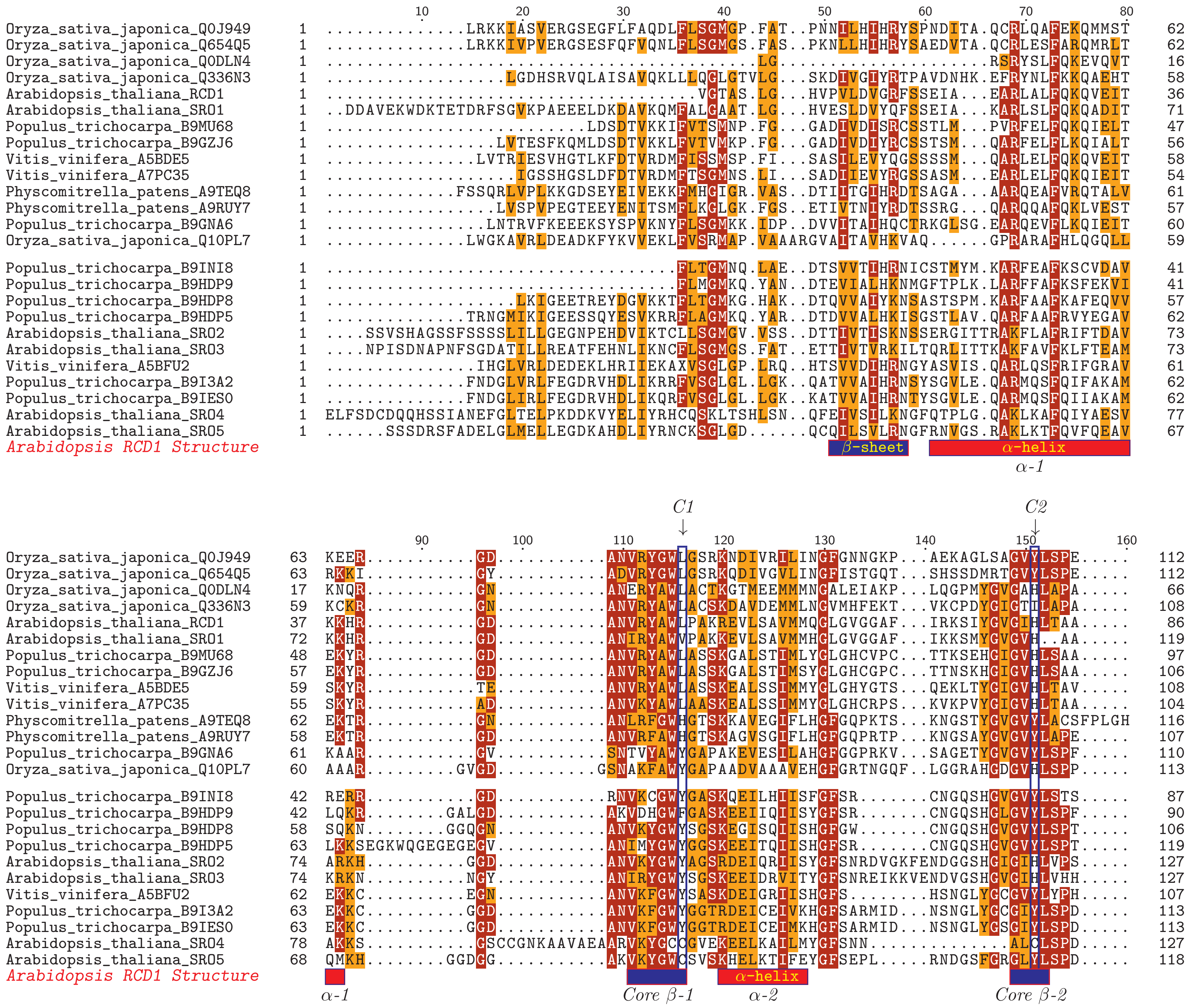
Oryza_sativa_japonica_Q0J949
Oryza_sativa_japonica_Q654Q5
Oryza_sativa_japonica_Q0DLN4
Oryza_sativa_japonica_Q336N3
Arabidopsis_thaliana_RCD1
Arabidopsis_thaliana_SRO1
Populus_trichocarpa_B9MU68
Populus_trichocarpa_B9GZJ6
Vitis_vinifera_A5BDE5
Vitis_vinifera_A7PC35
Physcomitrella_patens_A9TEQ8
Physcomitrella_patens_A9RUY7
Populus_trichocarpa_B9GNA6
Oryza_sativa_japonica_Q10PL7
Populus_trichocarpa_B9INI8
Populus_trichocarpa_B9HDP9
Populus_trichocarpa_B9HDP8
Populus_trichocarpa_B9HDP5
Arabidopsis_thaliana_SRO2
Arabidopsis_thaliana_SRO3
Vitis_vinifera_A5BFU2
Populus_trichocarpa_B9I3A2
Populus_trichocarpa_B9IES0
Arabidopsis_thaliana_SRO4
Arabidopsis_thaliana_SRO5
Arabidopsis RCD1 Structure
β-sheet
α-helix
α-1
C1
C2
Core β-1
α-2
Core β-2

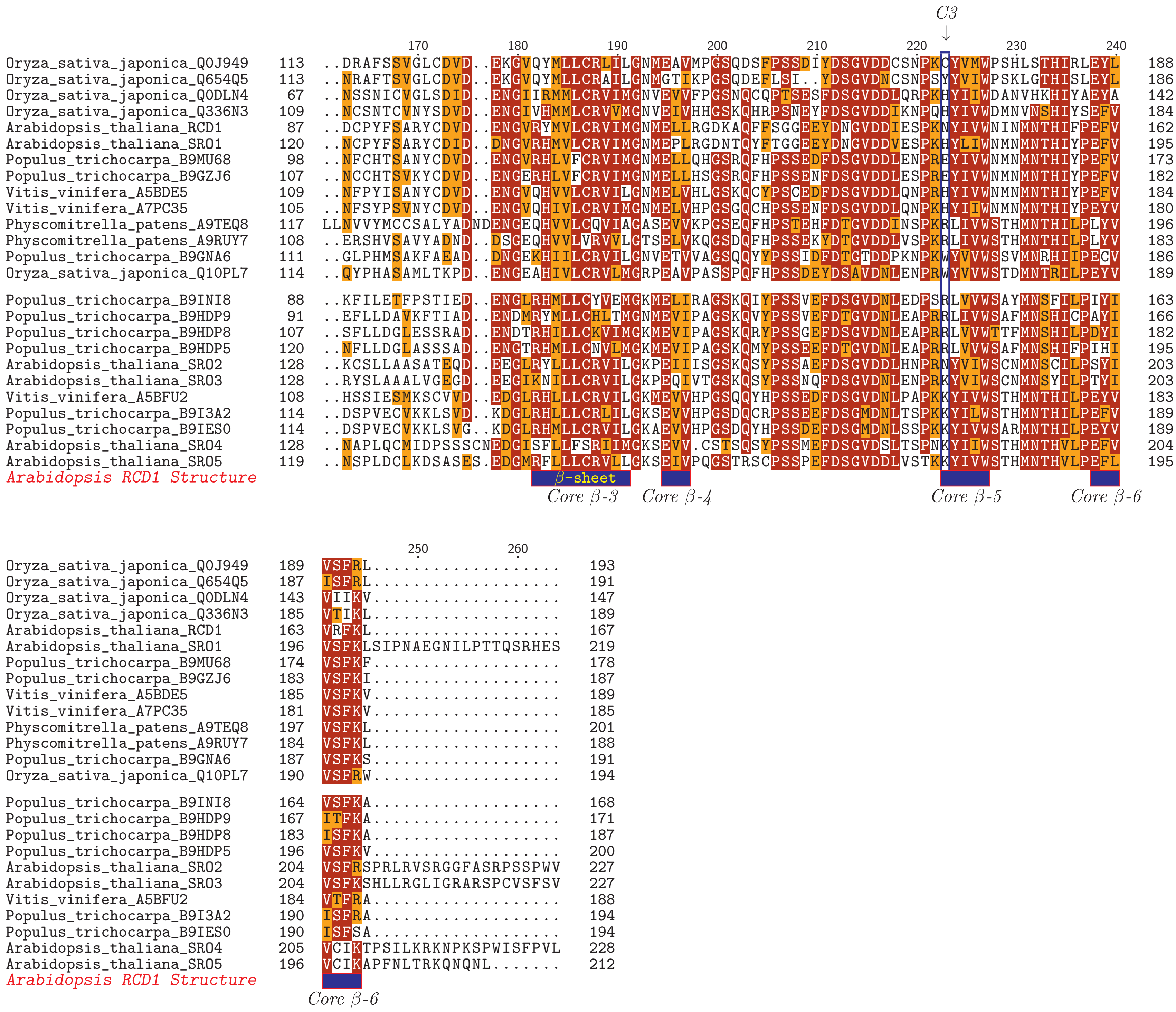
C3
170 180 190 200 210 220 230 240
Oryza_sativa_japonica_Q0J949 113 ..DRAFSSVGLCDVD..EKGVQYMLLCRLILGNMEAVMPGSQDSFPSSDIYDSGVDDCSNPKCYVMWPSHLSTHIRLEYL 188
Oryza_sativa_japonica_Q654Q5 113 ..NRAFTSVGLCDVD..EKGVQYMLLCRAILGNMGTIKPGSQDEFLSI..YDSGVDNCSNPSYYVIWPSKLGTHISLEYL 186
Oryza_sativa_japonica_Q0DLN4 67 ..NSSNICVGLSDID..ENGIIRMMLCRVIMGNVEVVFPGSNQCQPTSESFDSGVDDLQRPKHYIIWDANVHKHIYAEYA 142
Oryza_sativa_japonica_Q336N3 109 ..NCSNTCVNYSDVD..ENGIVHMMLCRVVMGNVEIVHHGSKQHRPSNEYFDSGVDDIKNPQHYIVWDMNVNSHIYSEFV 184
Arabidopsis_thaliana_RCD1 87 ..DCPYFSARYCDVD..ENGVRYMVLCRVIMGNMELLRGDKAQFFSGGEEYDNGVDDIESPKNYIVWNINMNTHIFPEFV 162
Arabidopsis_thaliana_SRO1 120 ..NCPYFSARYCDID..DNGVRHMVLCRVIMGNMEPLRGDNTQYFTGGEEYDNGVDDVESPKHYLIWNMNMNTHIYPEFV 195
Populus_trichocarpa_B9MU68 98 ..NFCHTSANYCDVD..ENGVRHLVFCRVIMGNMELLQHGSRQFHPSSEDFDSGVDDLENPREYIVWNMNMNTHIYPEFV 173
Populus_trichocarpa_B9GZJ6 107 ..NCCHTSVKYCDVD..ENGERHLVFCRVIMGNMELLHSGSRQFHPSSENFDSGVDDLESPREYIVWNMNMNTHIYPEFV 182
Vitis_vinifera_A5BDE5 109 ..NFPYISANYCDVD..ENGVQHVVLCRVILGNMELVHLGSKQCYPSCEDFDSGVDDLQNPRHYIVWNMNMNTHIYPEFV 184
Vitis_vinifera_A7PC35 105 ..NFSYPSVNYCDVD..ENGVQHIVLCRVIMGNMELVHPGSGQCHPSSENFDSGVDDLQNPKHYIIWNMNMNTHIYPEYV 180
Physcomitrella_patens_A9TEQ8 117 LLNVVYMCCSALYADNDENGEQHVVLCQVIAGASEVVKPGSEQFHPSTEHFDTGVDDINSPKRLIVWSTHMNTHILPLYV 196
Physcomitrella_patens_A9RUY7 108 ..ERSHVSAVYADND..DSGEQHVVLVRVVLGTSELVKQGSDQFHPSSEKYDTGVDDLVSPKRLIVWSTHMNTHILPLYV 183
Populus_trichocarpa_B9GNA6 111 ..GLPHMSAKFAEAD..DNGEKHIILCRVILGNVETVVAGSQQYYPSSIDFDTGTDDPKNPKWYVVWSSVMNRHIIPECV 186
Oryza_sativa_japonica_Q10PL7 114 ..QYPHASAMLTKPD..ENGEAHIVLCRVLMGRPEAVPASSPQFHPSSDEYDSAVDNLENPRWYVVWSTDMNTRILPEYV 189
Populus_trichocarpa_B9INI8 88 ..KFILETFPSTIED..ENGLRHMLLCYVEMGKMELIRAGSKQIYPSSVEFDSGVDNLEDPSRLVVWSAYMNSFILPIYI 163
Populus_trichocarpa_B9HDP9 91 ..EFLLDAVKFTIAD..ENDMRYMLLCHLTMGNMEVIPAGSKQVYPSSVEFDTGVDNLEAPRRLIVWSAFMNSHICPAYI 166
Populus_trichocarpa_B9HDP8 107 ..SFLLDGLESSRAD..ENDTRHILLCKVIMGKMEVIPAGSKQRYPSSGEFDSGVDNLEAPRRLVVWTTFMNSHILPDYI 182
Populus_trichocarpa_B9HDP5 120 ..NFLLDGLASSSAD..ENGTRHMLLCNVLMGKMEVIPAGSKQMYPSSEEFDTGVDNLEAPRRLVVWSAFMNSHIFPIHI 195
Arabidopsis_thaliana_SRO2 128 ..KCSLLAASATEQD..EEGLRYLLLCRVILGKPEIIISGSKQSYPSSAEFDSGVDDLHNPRNYVIWSCNMNSCILPSYI 203
Arabidopsis_thaliana_SRO3 128 ..RYSLAAALVGEGD..EEGIKNILLCRVILGKPEQIVTGSKQSYPSSNQFDSGVDNLENPRKYVIWSCNMNSYILPTYI 203
Vitis_vinifera_A5BFU2 108 ..HSSIESMKSCVVD..EDGLRHLLLCRVILGKMEVVHPGSQQYHPSSEDFDSGVDNLPAPKKYIVWSTHMNTHILPEYV 183
Populus_trichocarpa_B9I3A2 114 ..DSPVECVKKLSVD..KDGLRHLLLCRLILGKSEVVHPGSDQCRPSSEEFDSGMDNLTSPKKYILWSTHMNTHILPEFV 189
Populus_trichocarpa_B9IES0 114 ..DSPVECVKKLSVG..KDGLRHMLLCRVILGKAEVVHPGSDQYHPSSDEFDSGMDNLSSPKKYIVWSARMNTHILPEYV 189
Arabidopsis_thaliana_SRO4 128 ..NAPLQCMIDPSSSCNEDGISFLLFSRIIMGKSEVV.CSTSQSYPSSMEFDSGVDSLTSPNKYIIWSTHMNTHVLPEFV 204
Arabidopsis_thaliana_SRO5 119 ..NSPLDCLKDSASES.EDGMRFLLLCRVLLGKSEIVPQGSTRSCPSSPEFDSGVDDLVSTKKYIVWSTHMNTHVLPEFL 195
Arabidopsis RCD1 Structure
β-sheet
Core β-3
Core β-4
Core β-5
Core β-6
250 260
Oryza_sativa_japonica_Q0J949 189 VSFRL................... 193
Oryza_sativa_japonica_Q654Q5 187 ISFRL................... 191
Oryza_sativa_japonica_Q0DLN4 143 VIIKV................... 147
Oryza_sativa_japonica_Q336N3 185 VTIKL................... 189
Arabidopsis_thaliana_RCD1 163 VRFKL................... 167
Arabidopsis_thaliana_SRO1 196 VSFKLSIPNAEGNILPTTQSRHES 219
Populus_trichocarpa_B9MU68 174 VSFKF................... 178
Populus_trichocarpa_B9GZJ6 183 VSFKI................... 187
Vitis_vinifera_A5BDE5 185 VSFKV................... 189
Vitis_vinifera_A7PC35 181 VSFKV................... 185
Physcomitrella_patens_A9TEQ8 197 VSFKL................... 201
Physcomitrella_patens_A9RUY7 184 VSFKL................... 188
Populus_trichocarpa_B9GNA6 187 VSFKS................... 191
Oryza_sativa_japonica_Q10PL7 190 VSFRW................... 194
Populus_trichocarpa_B9INI8 164 VSFKA................... 168
Populus_trichocarpa_B9HDP9 167 ITFKA................... 171
Populus_trichocarpa_B9HDP8 183 ISFKA................... 187
Populus_trichocarpa_B9HDP5 196 VSFKV................... 200
Arabidopsis_thaliana_SRO2 204 VSFRSPRLRVSRGGFASRPSSPWV 227
Arabidopsis_thaliana_SRO3 204 VSFKSHLLRGLIGRARSPCVSFSV 227
Vitis_vinifera_A5BFU2 184 VTFRA................... 188
Populus_trichocarpa_B9I3A2 190 ISFRA................... 194
Populus_trichocarpa_B9IES0 190 ISFSA................... 194
Arabidopsis_thaliana_SRO4 205 VCIKTPSILKRKNPKSPWISFPVL 228
Arabidopsis_thaliana_SRO5 196 VCIKAPFNLTRKQNQNL....... 212
Arabidopsis RCD1 Structure
Core β-6
