# Supplementary material for: Evolutionary history of the poly(ADP-ribose) polymerase gene family in eukaryotes
Source: BMC Evol Biol. 2010 Oct 13;10:308. doi: 10.1186/1471-2148-10-308 (PMC2964712; doi:10.1186/1471-2148-10-308)
Supplement: Additional file 9 — Multiple alignment of the PARP catalytic domain from Clade 4 PARP proteins annotated with structural information. These alignments only show the conserved PARP catalytic domain. The structural elements present in Homo sapiens TNK1are shown at the bottom of the alignment [172]. Annotations as in Additional file 5. [file 1471-2148-10-308-S9.PDF]

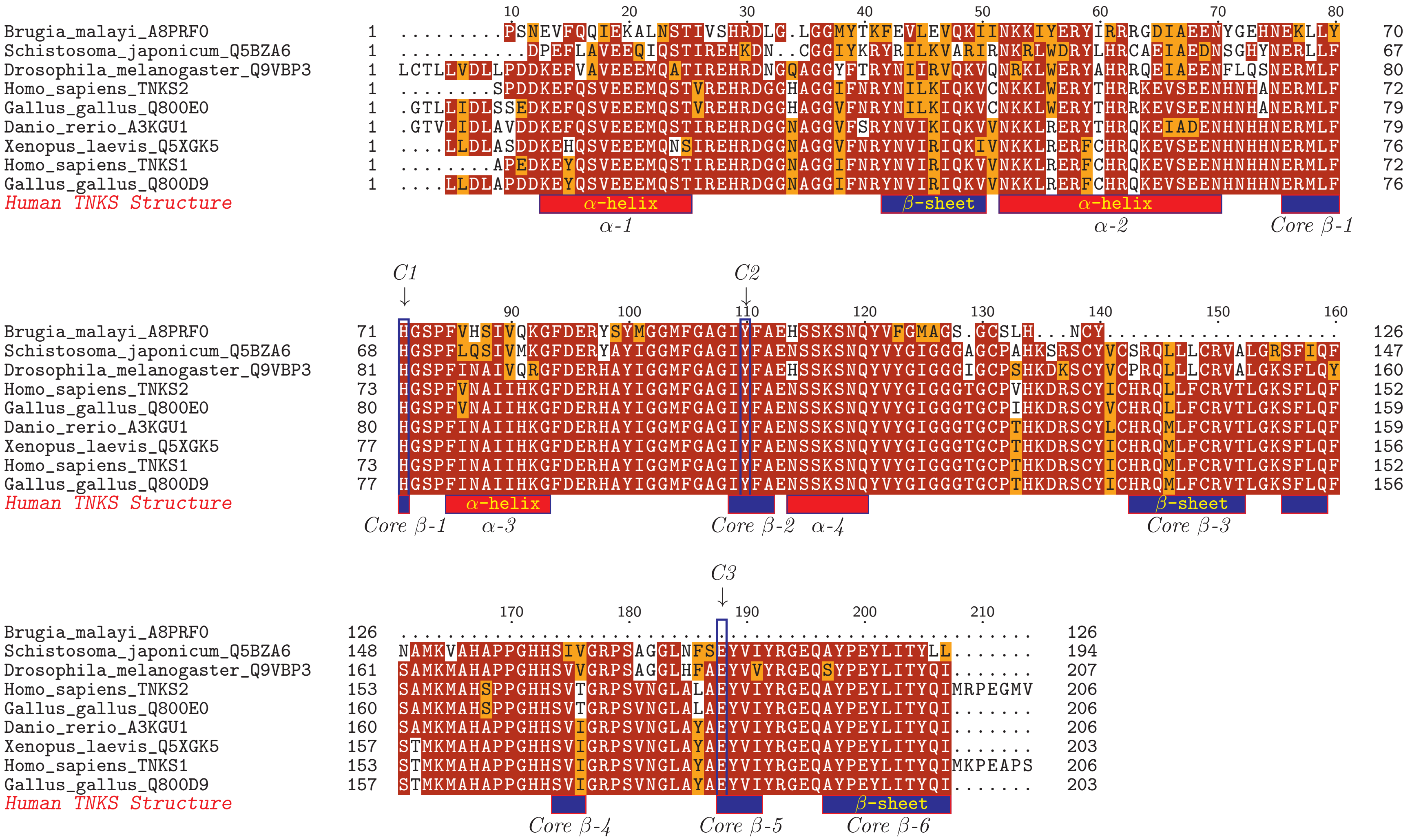

Brugia_malayi_A8PRF0 1 .........PSNEVFQQIEKALNSTIVSHRDLG.LGGMYTKFEVLEVQKIINKKIYERYIRRRGDIAEENYGEHNEKLLY 70
Schistosoma_japonicum_Q5BZA6 1 ...........DPEFLAVEEQIQSTIREHKDN..CGGIYKRYRILKVARIRNKRLWDRYLHRCAEIAEDNSGHYNERLLF 67
Drosophila_melanogaster_Q9VBP3 1 LCTLLVDLLPDDKEFVAVEEEMQATIREHRDNGQAGGYFTRYNIIRVQKVQNRKLWERYAHRRQEIAEENFLQSNERMLF 80
Homo_sapiens_TNKS2 1 ........SPDDKEFQSVEEEMQSTVREHRDGGHAGGIFNRYNILKIQKVCNKKLWERYTHRRKEVSEENHNHANERMLF 72
Gallus_gallus_Q800E0 1 .GTLLIDLSSEDKEFQSVEEEMQSTVREHRDGGHAGGVFNRYNILKIQKVCNKKLWERYTHRRKEVSEENHNHANERMLF 79
Danio_rerio_A3KGU1 1 .GTVLIDLAVDDKEFQSVEEEMQSTIREHRDGGNAGGVFSRYNVIKIQKVVNKKLRERYTHRQKEIADENHNHHNERMLF 79
Xenopus_laevis_Q5XGK5 1 ....LLDLASDDKEHQSVEEEMQNSIREHRDGGNAGGVFNRYNVIRIQKIVNKKLRERFCHRQKEVSEENHNHHNERMLF 76
Homo_sapiens_TNKS1 1 ........APEDKEYQSVEEEMQSTIREHRDGGNAGGIFNRYNVIRIQKVVNKKLRERFCHRQKEVSEENHNHHNERMLF 72
Gallus_gallus_Q800D9 1 ....LLDLAPDDKEYQSVEEEMQSTIREHRDGGNAGGIFNRYNVIRIQKVVNKKLRERFCHRQKEVSEENHNHHNERMLF 76
Human TNKS Structure
α-helix β-sheet α-helix
α-1 α-2 Core β-1
C1 C2
Brugia_malayi_A8PRF0 71 HGSPFVHSIVQKGFDERYSYMGGMFGAGIYFAEHSSKSNQYVFGMAGS.GCSLH...NCY.................... 126
Schistosoma_japonicum_Q5BZA6 68 HGSPFLQSIVMKGFDERYAYIGGMFGAGIYFAENSSKSNQYVYGIGGAGCPAHKSRSCYVCSRQLLLCRVALGRSFIQF 147
Drosophila_melanogaster_Q9VBP3 81 HGSPFINAIVQRGFDERHAYIGGMFGAGIYFAEHSSKSNQYVYGIGGIGCPSHKDKSCYVCPRQLLLCRVALGKSFLQY 160
Homo_sapiens_TNKS2 73 HGSPFVNAIIHKGFDERHAYIGGMFGAGIYFAENSSKSNQYVYGIGGTGCPVHKDRSCYICHRQLLFCRVTLGKSFLQF 152
Gallus_gallus_Q800E0 80 HGSPFVNAIIHKGFDERHAYIGGMFGAGIYFAENSSKSNQYVYGIGGTGCPIHKDRSCYVCHRQLLFCRVTLGKSFLQF 159
Danio_rerio_A3KGU1 80 HGSPFINAIIHKGFDERHAYIGGMFGAGIYFAENSSKSNQYVYGIGGTGCPTHKDRSCYLCHRQMLFCRVTLGKSFLQF 159
Xenopus_laevis_Q5XGK5 77 HGSPFINAIIHKGFDERHAYIGGMFGAGIYFAENSSKSNQYVYGIGGTGCPTHKDRSCYICHRQMLFCRVTLGKSFLQF 156
Homo_sapiens_TNKS1 73 HGSPFINAIIHKGFDERHAYIGGMFGAGIYFAENSSKSNQYVYGIGGTGCPTHKDRSCYICHRQMLFCRVTLGKSFLQF 152
Gallus_gallus_Q800D9 77 HGSPFINAIIHKGFDERHAYIGGMFGAGIYFAENSSKSNQYVYGIGGTGCPTHKDRSCYICHRQMLFCRVTLGKSFLQF 156
Human TNKS Structure
α-helix β-sheet
Core β-1 α-3 Core β-2 α-4 Core β-3
C3
Brugia_malayi_A8PRF0 126 126
Schistosoma_japonicum_Q5BZA6 148 NAMKVAHAPPGHHSIVGRPSAGGLNFSEYVIYRGEQAYPEYLITYLL....... 194
Drosophila_melanogaster_Q9VBP3 161 SAMKMAHAPPGHHSVVGRPSAGGLHFAEYVVYRGEQSYPEYLITYQI....... 207
Homo_sapiens_TNKS2 153 SAMKMAHSPPGHHSVTGRPSVNGLALAEYVIYRGEQAYPEYLITYQIMRPEGMV 206
Gallus_gallus_Q800E0 160 SAMKMAHSPPGHHSVTGRPSVNGLALAEYVIYRGEQAYPEYLITYQI....... 206
Danio_rerio_A3KGU1 160 SAMKMAHAPPGHHSVIGRPSVNGLAYAEYVIYRGEQAYPEYLITYQI....... 206
Xenopus_laevis_Q5XGK5 157 STMKMAHAPPGHHSVIGRPSVNGLAYAEYVIYRGEQAYPEYLITYQI....... 203
Homo_sapiens_TNKS1 153 STMKMAHAPPGHHSVIGRPSVNGLAYAEYVIYRGEQAYPEYLITYQIMKPEAPS 206
Gallus_gallus_Q800D9 157 STMKMAHAPPGHHSVIGRPSVNGLAYAEYVIYRGEQAYPEYLITYQI....... 203
Human TNKS Structure
β-sheet
Core β-4 Core β-5 Core β-6
